# 4th Pediatric Allergy and Asthma Meeting (PAAM)

**DOI:** 10.1186/s13601-016-0117-8

**Published:** 2016-11-21

**Authors:** S. Tolga Yavuz, Ozan Koc, Ali Gungor, Faysal Gok, Jessica Hawley, Christopher O’Brien, Matthew Thomas, Malcolm Brodlie, Louise Michaelis, Inês Mota, Ângela Gaspar, Susana Piedade, Graça Sampaio, José Geraldo Dias, Miguel Paiva, Mário Morais-Almeida, Cristina Madureira, Tânia Lopes, Susana Lopes, Filipa Almeida, Alexandra Sequeira, Fernanda Carvalho, José Oliveira, Fabienne Gay-Crosier, Ioana-Valentina Nenciu, Andreia Florina Nita, Alexandru Ulmeanu, Dumitru Oraseanu, Carmen Zapucioiu, Adrianna Machinena, Olga Domínguez Sánchez, Montserrat Alvaro Lozano, Rosa Jiménez Feijoo, Jaime Lozano Blasco, Mònica Piquer Gibert, Mª Teresa Giner Muñoz, Marcia Dias da Costa, Ana Maria Plaza Martín, Ebru Arik Yilmaz, Özlem Cavkaytar, Betul Buyuktiryaki, Ozge Soyer, Cansin Sackesen, Merryn Netting, Adaweyah El-Merhibi, Michael Gold, Patrick Quinn, Irmeli Penttila, Maria Makrides, Stavroula Giavi, Antonella Muraro, Roger Lauener, Annick Mercenier, Eugen Bersuch, Isabella M. Montagner, Maria Passioti, Nicolò Celegato, Selina Summermatter, Sophie Nutten, Tristan Bourdeau, Yvonne M. Vissers, Nikolaos G. Papadopoulos, Hanneke van der Kleij, Hans Warmenhoven, Ronald van Ree, Raymond Pieters, Dirk Jan Opstelten, Hans van Schijndel, Joost Smit, Roisin Fitzsimons, Victoria Timms, George Du Toit, Guven Kaya, Mustafa Gulec, Mehmet Saldir, Osman Sener, Nagwa Hassan, Hala Shaaban, Hazem El-Hariri, Ahmed Kamel Inas E. Mahfouz, Papp Gabor, Biro Gabor, Kovacs Csaba, Bo Chawes, Klaus Bønnelykke, Jakob Stokholm, Lene Heickendorff, Susanne Brix, Morten Rasmussen, Hans Bisgaard, Henrik Wegener Hallas, Lambang Arianto, Maike Pincus, Thomas Keil, Andreas Reich, Ulrich Wahn, Susanne Lau, Linus Grabenhenrich, Sara Fagerstedt, Helena Marell Hesla, Emelie Johansson, Helen Rosenlund, Axel Mie, Annika Scheynius, Johan Alm, Jorge Esparza-Gordillo, Anja Matanovic, Ingo Marenholz, Anja Bauerfeind, Klaus Rohde, Katja Nemat, Min-Ae Lee-Kirsch, Magnus Nordenskjöld, Marten C.G. Winge, Renate Krüger, Kirsten Beyer, Birgit Kalb, Bodo Niggemann, Norbert Hübner, Heather J. Cordell, Maria Bradley, Young-Ae Lee, Hannah Gough, Dirk Schramm, John Beschorner, Antje Schuster, Carl-Peter Bauer, Johannes Forster, Fred Zepp, Renate Bergmann, Karl Bergmann, Filipe Benito Garcia, Natacha Santos, Helena Pité, Athina Papadopoulou, Despina Mermiri, Elpida Xatziagorou, Ioannis Tsanakas, Stavroula Lampidi, Kostas Priftis, Elaine Fuertes, Iana Markevych, Gayan Bowatte, Olena Gruzieva, Ulrike Gehring, Allan Becker, Dietrich Berdel, Michael Brauer, Chris Carlsten, Barbara Hoffmann, Anita Kozyrskyj, Caroline Lodge, Göran Pershagen, Alet Wijga, Heinrich Joachim, Zorica Zivkovic, Ivana Djuric-Filipovic, Jasmina Jocić-Stevanovic, Snežana Zivanovic, Styliani Taka, Dimitra Kokkinou, Aliki Papakonstantinou, Panagiota Stefanopoulou, Anastasia Georgountzou, Paraskevi Maggina, Sofia Stamataki, Vassiliki Papaevanggelou, Evangelos Andreakos, Monica Piquer Gibert, Adriana Machinena Spera, Matea Deliu, Danielle Belgrave, Angela Simpson, Adnan Custovic, João Gaspar Marques, Pedro Carreiro-Martins, Joana Belo, Sara Serranho, Isabel Peralta, Nuno Neuparth, Paula Leiria-Pinto, Marta Vazquez-Ortiz, Mariona Pascal, Ana Maria Plaza, Manel Juan, Lorella Paparo, Rita Nocerino, Rosita Aitoro, Ilaria Langella, Antonio Amoroso, Alessia Amoroso, Carmen Di Scala, Roberto Berni Canani, Santanu Maity, Giuseppina Rotiroti, Minal Gandhi, Karin Jonsson, Annika Ljung, Bill Hesselmar, Ingegerd Adlerbert, Hilde Brekke, Susanne Johansen, Agnes Wold, Ann-Sofie Sandberg, Björn Nordlund, Cecilia Lundholm, Villhelmina Ullemar, Marianne van Hage, Anne Örtqvist, Catarina Almqvist, Anna Selby, Kate Grimshaw, Michael Clausen, Ruta Dubakiene, Alessandro Fiocchi, Marek Kowalski, Nikos Papadopoulos, Marta Reche, Sigurveig Sigurdardottir, Aline Sprikkleman, Paraskevi Xepapadaki, Clare Mills, Graham Roberts, Herberto Jose Chong Neto, Gustavo Falbo Wandalsen, Ana Carolina Dela Bianca, Carolina Aranda, Nelson Augusto Rosário, Dirceu Solé, Javier Mallol, Luis García Marcos, Ivana Banic, Matija Rijavec, Davor Plavec, Peter Korosec, Mirjana Turkalj, Alen Bozicevic, Maria De Mieri, Matthias Hamburger, Simone Holley, Ruth Morris, Frances Mitchell, Rebecca Knibb, Susan Latter, Christina Liossi, Mostafa M. M. Hassan, Malin Barman, Anna Sandin, Daniela Posa, Serena Perna, Ute Hoffmann, Kuan-Wei Chen, Yvonne Resch, Susanne Vrtala, Rudolf Valenta, Paolo Maria Matricardi, Olympia Tsilochristou, Alexander Rohrbach, Antonio Cappella, Stephanie Hofmaier, Laura Hatzler, Raffaele D’Amelio, Sophia Björkander, Maria A. Johansson, Gintare Lasaviciute, Eva Sverremark-Ekström, Franz Rüschendorf, David P. Strachan, Ben D. Spycher, Hansjörg Baurecht, Patricia Margaritte-Jeannin, Annika Sääf, Marjan Kerkhof, Markus Ege, Svetlana Baltic, Melanie C. Matheson, Jin Li, Sven Michel, Wei Q. Ang, Wendy McArdle, Andreas Arnold, Georg Homuth, Florence Demenais, Emmanuelle Bouzigon, Cilla Söderhäll, Johan C. de Jongste, Dirkje S. Postma, Charlotte Braun-Fahrländer, Elisabeth Horak, Ludmila M. Ogorodova, Valery P. Puzyrev, Elena Yu Bragina, Thomas J. Hudson, Charles Morin, David L. Duffy, Guy B. Marks, Colin F. Robertson, Grant W. Montgomery, Bill Musk, Philip J. Thompson, Nicholas G. Martin, Alan James, Patrick Sleiman, Elina Toskala, Elke Rodriguez, Regina Fölster-Holst, Andre Franke, Wolfgang Lieb, Christian Gieger, Andrea Heinzmann, Ernst Rietschel, Sven Cichon, Markus M. Nöthen, Craig E. Pennell, Peter D. Sly, Carsten O. Schmidt, Valentin Schneider, Matthias Heinig, Patrick G. Holt, Michael Kabesch, Stefan Weidinger, Hakon Hakonarson, Manuel AR Ferreira, Catherine Laprise, Maxim B Freidin, Jon Genuneit, Gerard H Koppelman, Erik Melén, Marie-Hélène Dizier, A. John Henderson, Young Ae Lee, Purificacion González-Delgado, Esther Caparrós, Fernando Clemente, Begoña Cueva, Victoria M. Moreno, Jose Luis Carretero, Javier Fernández, Kate Swan, Mudiyur Gopi, Tim Smith, Edara Ramesh, Arun Sadasivam, Cristina Arêde, Luís Miguel Borrego, Graça Pires, Cristina Santa-Marta, Stephanie Brand, Karina Stein, Holger Heine, Marion Kauth, Leif Bjarte Rolfsjord, Egil Bakkeheim, Håvard Ove Skjerven, Kai-Håkon Carlsen, Jon Olav Hunderi, Teresa Løvold Berents, Petter Mowinckel, Karin C. Lødrup Carlsen, Ullrich Munzel, William Berger, Román Valiente, the Bilastine Pediatric Safety Study Group, Valvanera Vozmediano, John C. Lukas, Mónica Rodríguez, Sebastiano Guarnaccia, Luigi Vitale, Ada Pluda, Emanuele D’Agata, Denise Colombo, Stefano Felici, Valeria Gretter, Susanna Facchetti, Gaia Pecorelli, Cristina Quecchia, George Guibas, Evangelia Spandou, Spyridon Megremis, Peter West, Nikolaos Papadopoulos, João Cavaleiro Rufo, Joana Madureira, Inês Paciência, Lívia Aguiar, Patrícia Padrão, Mariana Pinto, Luís Delgado, Pedro Moreira, João Paulo Teixeira, Eduardo Oliveira Fernandes, André Moreira, Adriana Izquierdo Dominguez, Antonio Valero, Joaquim Mullol, Alfonso Del Cuvillo, Javier Montoro, Ignacio Jauregui, Joan Bartra, Ignacio Davila, Marta Ferrer, Joaquin Sastre, Catarina Martins, Jorge Lima, Maria José Leandro, Glória Nunes, Jorge Cunha Branco, Hélder Trindade, Luis Miguel Borrego, Secil Conkar, Mehtap Kilic, Canan Aygun, Recep Sancak, Eleni Tagalaki, Lambros Banos, Anna Vlachou, Fotini Giannoula, Marina Pavlakou, Maria Kryoni, Kostas Makris, Snezhina Lazova, Guergana Petrova, Dimitrinka Miteva, Penka Perenovska, Aliya Klyucharova, Olesya Skorohodkina, Dimitra Koumaki, Alkisti Manousaki, Maria Agrapidi, Lida Iatridou, Omima Eruk, Konstantinos Myridakis, Emmanouil Manousakis, Vasiliki Koumaki, Maria Dimou, Maria Ingemansson, Gunilla Hedlin, Nitida Pastor, Delphine de Boissieu, Jon Vanderhoof, Nancy Moore, Kaitlin Maditz, Adeli Mehdi, Shaza Elhassan, Carolin Beck, Ahmed Al-Hammadi, Ioana Maris, Ronan O’Sullivan, Jonathan Hourihane, George Raptis, Audrey DunnGalvin, Matthew Greenhawt, Carina Venter, Evelyn O’Regan, Duncan Cronin, Anna O’Reilly, Foued Abdelaziz, Dounia Khelifi-Touhami, Nihad Selim, Tahar Khelifi-Touhami, Pablo Merida, Ana Mª Plaza, Juan Heber Castellanos, Jaime Lozano, Olga Dominguez, Monica Piquer, Rosa Jimenez, Mª Teresa Giner, Konstantinos Kakleas, Manohar Joishy, Wendmu Maskele, Huw R. Jenkins, Mercedes Escarrer, Agustín Madroñero, Maria Teresa Guerra, Juan Carlos Julia, Juan Carlos Cerda, Javier Contreras, Eulalia Tauler, Maria Jesus Vidorreta, Ana Rojo, Silvia Del Valle, Niamh Flynn, Gary Foley, Carol Harmon, John Fitzsimons, Krasimira Baynova, Ávila Maria Del Robledo, Labella Marina, Aaron Cortes, Alicia Sciaraffia, Angela Castillo, Nanna Juel-Berg, Kirsten Skamstrup Hansen, Lars Kærgaard Poulsen, Adina Lazar, Rita Aguiar, Anabela Lopes, Maria J. Paes, Amélia S. Santos, M. A. Pereira-Barbosa, Hatice Eke Gungor, Salih Uytun, Umit Murat Sahiner, Yasemin Altuner Torun, Mirjana Zivanovic, Marina Atanasković-Marković, Tina Vesel, Mihaela Nahtigal, Andreja Obermayer-Temlin, Eva Šoster Križnik, Mirjana Maslar, Ruben Bizjak, Marjeta Tomšič-Matic, Sonja Posega-Devetak, Maja Skerbinjek-Kavalar, Mateja Predalič, Tadej Avčin, Guillaume Pouessel, Etienne Beaudouin, Anne M. Moneret-Vautrin, Antoine Deschildre, Allergy Vigilance Network, Marta Viñas, Bartolomé Borja, Nora Hernández, Mª José Castillo, Adriana Izquierdo, Marcel Ibero, Can Naci Kocabas, Camille Heming, Emily Garrett, Adam Blackstock, Rahul Chodhari, Simona Belohlavkova, Eliska Kopelentova, Petr Visek, Ivana Setinova, Ivana Svarcova, Sigrid Sjölander, Nora Nilsson, Malin Berthold, Helena Ekoff, Magnus Borres, Caroline Nilsson, Loreto González Domínguez, Cristina Muñoz Archidona, Ana Moreira Jorge, Sergio Quevedo Teruel, Teresa Bracamonte Bermejo, Miriam Castillo Fernández, Fernando Pineda de la Losa, Luis Ángel Echeverría Zudaire, Olga Vrani, Antigone Mavroudi, Maria Fotoulaki, Maria Emporiadou, Kleomenis Spiroglou, Ioannis Xinias, Helyeh A. Sadreddini, Mia Warnes, Donna Traves, Gordana Kostić, Đorđe Filipovic, Sawapon Sittisomwong, Siripong Sittisomwong, Zygmunt Podolec, Marcin Hartel, Daria Panek, Magdalena Podolec-Rubiś, Tomasz Banasik, Elham Abbasi, Mozhgan Moghtaderi, Phani Sanneerappa, Alina Deliu, Moosa Kutty, Nagabathula Ramesh, Roya Sherkat, Mohammad Reza Sabri, Bahar Dehghan, Hamid Bigdelian, Nahid Raeesi, Mino Afshar, Hamid Rahimi, Christoph Klein, Mohemid Al-Jebouri, Oxana A. Svitich, Daria O. Zubacheva, Dmitrii A. Potemkin, Ludmila V. Gankovskaya, Vitalii V. Zverev, Elaine OB Doyle, Paul Gallagher, Sherine Dewlett, Kin Man, James Pocock, Anna Gerrardhughes, Jolanta Wasilewska, Maciej Kaczmarski, Dariusz Lebensztejn, Chandramani Thuraisingham, Davendralingam Sinniah, Yue Chen, Xiaomei Mei, Sebnem Ozdogan, Pinar Karadeniz, Durdugul Ayyildiz-Emecen, Ummuhan Oncul, Gizem Sari, Sabanur Cavdar, Niloufar Farzan, Susanne J. Vijverberg, Colin J. Palmer, Kelan G. Tantisira, Anke-Hilse Maitland-van der Zee, on behalf of the PiCA consortium, Fatma Yavuzyilmaz, Nafiye Urganci, Merve Usta, Mehmet Hoxha, Maksim Basho, Gustavo F. Wandalsen, Fernanda Monteiro, Blerta Lame, Eris Mesonjesi, Arjeta Sherri, Alkerta Ibranji, Laert Gjati, Gjustina Loloci, Ardii Bardhi, Behnam Moghtaderi, Shirin Farjadian, Dorna Eghtedari, Manuela Olaya, Laura Del Mar Vasquez, Luis Fernando Ramirez, Carlos Daniel Serrano, Belgin Usta Guc, Suna Asilsoy, Fulya Ozer, Sylvia Shopova, Vera Papochieva, Jessica Loekmanwidjaja, Márcia Mallozi, Paul Ratner, Daniel Soteres, Zoltán Novák, Anahí Yáñez, Kiss Ildikó, Piotr Kuna, Miguel Tortajada, Román Valiente, Julia Feuerhahn, Christine Blome, Meike Hadler, Efstrathios Karagiannis, Anna Langenbruch, Matthias Augustin, Michel Roux, Shinji Kakudo, Robert K. Zeldin, Anna Sokolova, Tiago Milheiro Silva, Snezana S. Zivanovic, Vesna Cvetkovic, Ivana Nikolic, Sonja J. Zivanovic, Ljiljana Saranac, Zoia Nesterenko, Snezana Radic, Branislava Milenkovic, Spomenka Smiljanic, Milka Micic-Stanijevic, Olivera Calovic, Anne Marie Bro Hofbauer, Lone Agertoft, Lucy Everson, Jessica Kearney, Jonny Coppel, Simon Braithwaite, Elisabeth S. Christiansen, Henrik Fomsgaard Kjaer, Esben Eller, Charlotte G. Mørtz, Susanne Halken, Cristina Román India, Juana Jiménez Jiménez, Luis Echeverría Zudaire, Cathal O’Connor, Varvara Kanti, Lena Lünnemann, Günther Malise, Laine Ludriksone, Andrea Stroux, Wolfgang Henrich, Michael Abu-Dakn, Ulrike Blume-Peytavi, Natalie Garcia Bartels, Marianne Schario, Thorsten Stanley, Nicolien Brandenbarg, Alia Boardman, Gary McGreevy, Emily Rodger, Katherine Knight, Trisha Taylor, Gemma Scanlan, Grüber Christoph, Margriet van Stuivenberg, Fabio Mosca, Guido Moro, Gaetano Chirico, Christian P. Braegger, Joseph Riedler, Yalcin Yavuz, Günther Boehm, Stefania Arasi, Giuseppe Crisafulli, Lucia Caminiti, Federica Porcaro, Giovanni Battista Pajno, Akane Tanaka, Yaei Togawa, Kumiko Oida, Naotomo Kambe, Peter Arkwright, Yosuke Amagai, Naoki Shimojo, Yasunori Sato, Hiroyuki Mochizuki, Hyosun Jang, Saori Ishizaka, Hiroshi Matsuda, Wisnu Barlianto, Ery Olivianto, H. M. S. Chandra Kusuma, Mariapia Mollica, Giovanna Trinchese, Elena Alfano, Francesco Amato, Claudio Pirozzi, Antonio Calignano, Rosaria Meli, Siri Rossberg, Kerstin Gerhold, Kurt Zimmermann, Mohammad Zaino, Thomas Geske, Eckard Hamelmann, Sarah Bogovic, Jochem van den Berg, Chantal Janssen, Angela Claver, Mª Flor Martin-Muñoz, C. Martorell, M. T. Belver, E. Alonso Lebrero, L. Zapatero, V. Fuentes, M. Piqué, A. Plaza, C. Muñoz, Cristina Blasco, B. Villa, C. Gómez, S. Nevot, J. M. García, L. Echeverria, Brenda DeWitt, Judith Holloway, Donald Hodge, Sian Ludman, Merhdad Jafari-Mamaghani, Rosemary Ebling, Adam T. Fox, Gideon Lack, Sofia Lovén Björkman, Natalia Ballardini, Supriyo Basu, Jenny Hallet, Jyothi Srinivas, Hazel Stringer, Nicola Jay, Paula Fonseca, Clara Vieira, Carla Mastrorilli, Carlo Caffarelli, Riccardo Asero, Salvatore Tripodi, Arianna Dondi, Gianpaolo Ricci, Carlotta Povesi Dascola, Elisabetta Calamelli, Francesca Cipriani, Andrea Di Rienzo Businco, Annamaria Bianchi, Paolo Candelotti, Tullio Frediani, Carmen Verga, Paraskevi Korovessi, Skevi Tiliakou, Evaggelia Tavoulari, Kalliopi-Maria Moraiti, Wan Jean Tee, Samir Deiratany, Raymond Seedhoo, Roisin McNamara, Ike Okafor, Ekaterina Khaleva, Gennady Novic, Natalia Bychkova, Amany Abd Al-Aziz, Amany Fatouh, Ayat Motawie, Eman El Bostany, Amr Ibrahim, Sylvia Andonova, Alexey Savov, Maria Zoto, Marialena Kyriakakou, Mariza Vassilopoulou, Athina Balaska, Stavroula Kostaridou, Jorien Wartna, Arthur M. Bohnen, Gijs Elshout, David H. J. Pols, Patrick J. E. Bindels, Sven F. Seys, Ellen Dilissen, Sarah Van der Eycken, An-Sofie Schelpe, Gudrun Marijsse, Thierry Troosters, Vincent Vanbelle, Sven Aertgeerts, Jan L. Ceuppens, Lieven J. Dupont, Koen Peers, Dominique M. Bullens, Sandra Bulat Lokas, Jelena Zivkovic, Boro Nogalo, Iva Mrkic Kobal, Georgeta Oliveira, Katharine Pike, Alda Melo, Tomás Amélia, José Carlos Cidrais Rodrigues, Cristina Serrano, José Manuel Lopes dos Santos, Carla Lopes, Uwe Schauer, Karl-Christian Bergmann, Luis Moral, Teresa Toral, Nuria Marco, Beléns García Avilés, Mª Jesús Fuentes, Jesús Garde, Cristina Montahud, Javier Perona, Mª José Forniés, Esozia Arroabarren, Marta Anda, Maria Luisa Sanz, Maria Teresa Lizaso, Candida Arregui, Sara May, Martha Hartz, Avni Joshi, Miguel A. Park, Sonja Posega Devetak, Anja Koren Jeverica, Leonor Castro, Carolina Gouveia, Ana Carvalho Marques, Antonio Jorge Cabral, Luis Amaral, Fabrícia Carolino, Eunice Castro, Madalena Passos, Josefina R. Cernadas, Luís Amaral, Eunice Dias de Castro, Fernando Pineda, Armanda Gomes, Helen Brough, Jobst Röhmel, Carsten Schwarz, Anne Mehl, Philippe Stock, Doris Staab, Christine Seib, Anita Critchlow, Alyson Barber, Belen Delavalle, Teresa Garriga, Blanca Vilá, Annalisa Astolfi, Costanza Di Chiara, Iria Neri, Annalisa Patrizi, Katerina Neskorodova, Asya Kudryavtseva, Jorge Alvarez, Miriam Palacios, Marta Martinez-Merino, Ibone Vaquero

**Affiliations:** 1 Department of Pediatric Allergy, GATA School of Medicine, Ankara, Turkey; 2 Department of Pediatrics, GATA School of Medicine, Ankara, Turkey; 3 Newcastle University, Newcastle, UK; 4 Royal Victoria Infirmary, Great North Children’s Hospital, Newcastle, UK; 5 Immunoallergy Department, CUF Descobertas Hospital, Lisbon, Portugal; 6 CHMA, Famalicão, Portugal; 7 Clinical Allergy and Clinical Immunology, Internal Medicine, Federal Specialist, Geneva, Switzerland; 8 “Grigore Alexandrescu” Emergency Hospital for Children, Bucharest, Romania; 9 Pediatric Allergy Department, Hospital Sant Joan de Déu, Barcelona, Spain; 10 Division of Pediatric Allergy, School of Medicine, Hacettepe University, Ankara, Turkey; 11 Women’s and Children’s Health Research Institute, Adelaide, Australia; 12 School of Pediatrics and Reproductive Health, University of Adelaide, Adelaide, Australia; 13 School of Medicine, The University of Adelaide, Adelaide, Australia; 14 South Australian Health Medical Research Institute, Adelaide, Australia; 15 Allergy Department, 2nd Pediatric Clinic, University of Athens, Athens, Greece; 16 Referral Centre for Food Allergy Diagnosis and Treatment, Veneto Region, Department of Women and Child Health, Padua University Hospital, Padua, Italy; 17 Children’s Hospital of Eastern Switzerland, St. Gallen, Switzerland; 18 CK-CARE, Davos, Switzerland; 19 Nestlé Research Center, Lausanne, Switzerland; 20 Department of Surgery, Oncology and Gastroenterology, Veneto Institute of Oncology IOV-IRCCS, Padua, Italy; 21 Centre for Pediatrics and Child Health, Institute of Human Development, University of Manchester, Manchester, UK; 22 HAL Allergy B.V., Haarlem, The Netherlands; 23 Experimental Immunology, Academic Medical Centre, Amsterdam, The Netherlands; 24 Institute for Risk Assessment Sciences, Immunotoxicology, Utrecht University, Utrecht, The Netherlands; 25 Guys and St Thomas’ NHS Trust, London, UK; 26 Department of Adult Immunology and Allergic Diseases, GATA School of Medicine, Ankara, Turkey; 27 National Research Centre, Cairo, Egypt; 28 Faculty of Medicine, Cairo University, Cairo, Egypt; 29 Szigetvar Hospital, Szigetvar, Hungary; 30 Danish Pediatric Asthma Center, Gentofte Hospital, University of Copenhagen, Gentofte, Denmark; 31 Department of Clinical Biochemistry, Århus University Hospital, Århus, Denmark; 32 Department of Systems Biology, Center for Biological Sequence Analysis, Technical University, Lyngby, Denmark; 33 Copenhagen Prospective Studies on Asthma in Childhood, Copenhagen University Hospital, Gentofte, Denmark; 34 Department of Pediatric Pneumology and Immunology, Charité-Universitätsmedizin Berlin, Berlin, Germany; 35 Institute of Social Medicine, Epidemiology and Health, Charité-Universitätsmedizin Berlin, Berlin, Germany; 36 Karolinska Institutet, Stockholm, Sweden; 37 Max-Delbrück-Centrum (MDC) for Molecular Medicine, Berlin, Germany; 38 Clinic for Pediatric Allergy, Experimental and Clinical Research Center, Charité Universitätsmedizin Berlin, Berlin, Germany; 39 Klinik fur Kinder- und Jugendmedizin, Technical University Dresden, Dresden, Germany; 40 Department of Molecular Medicine and Surgery, Karolinska Institutet, Stockholm, Sweden; 41 Institute for Social Medicine, Epidemiology and Health Economics, Charité Universitätsmedizin Berlin, Berlin, Germany; 42 Institute of Genetic Medicine, Newcastle University, Newcastle upon Tyne, UK; 43 Dermatology Unit, Department of Medicine, Solna Karolinska University Hospital, Stockholm, Solna, Sweden; 44 Charité-Universitätsmedizin Berlin, Berlin, Germany; 45 Allergy Pediatric Unit, KAT General Hospital, Athens, Greece; 46 Allergology and Respiratory Unit, Penteli’s Children Hospital, Athens, Greece; 47 3rd Pediatric Department, Aristoleleio University, Thessaloniki, Greece; 48 Pediatric Allergy and Pulmonology Units, 3rd Department of Pediatrics, University of Athens General Hospital “Attikon”, Athens, Greece; 49 Institute of Epidemiology I, Helmholtz Zentrum München – German Research Center for Environmental Health, Munich, Germany; 50 Division of Metabolic and Nutritional Medicine, Ludwig Maximilians University of Munich, Dr. von Hauner Children’s Hospital, Munich, Germany; 51 Allergy and Lung Health Unit, Melbourne School of Population and Global Health, The University of Melbourne, Melbourne, Australia; 52 Institute of Environmental Medicine, Karolinska Institutet, Stockholm, Sweden; 53 Institute for Risk Assessment Sciences, Utrecht University, Utrecht, The Netherlands; 54 Department of Pediatrics and Child Health, University of Manitoba, Winnipeg, MB Canada; 55 Research Institute, Department of Pediatrics, Marien-Hospital Wesel, Wesel, Germany; 56 School of Population and Public Health, University of British Columbia, Vancouver, BC Canada; 57 Department of Medicine, University of British Columbia, Vancouver, BC Canada; 58 IUF – Leibniz Research Institute for Environmental Medicine, Düsseldorf, Germany; 59 Heinrich-Heine University of Düsseldorf, Medical Faculty, Deanery of Medicine, Düsseldorf, Germany; 60 Department of Pediatrics, Faculty of Medicine and Dentistry, Women and Children’s Health Research Institute, Edmonton, AB Canada; 61 School of Public Health, University of Alberta, Edmonton, AB Canada; 62 Center for Nutrition, Prevention and Health Services, National Institute of Public Health and the Environment, Bilthoven, the Netherlands; 63 Children Hospital for Pulmonary Diseases and Tuberculosis, Belgrade, Serbia; 64 2nd Pediatric Clinic, University of Athens, Athens, Greece; 65 3rd Pediatric Clinic, University of Athens, Attikon University Hospital, Athens, Greece; 66 Biomedical Research Foundation, Academy of Athens, Athens, Greece; 67 Sant Joan de Déu Hospital, Barcelona, Spain; 68 University of Manchester, Manchester, UK; 69 Immunoallergy Department, Hospital de Dona Estefânia CHLC EPE, Lisbon, Portugal; 70 Pediatric Allergy Section, Sant Joan de Deu Hospital, Barcelona, Spain; 71 Immunology Department, Hospital Clinic, Barcelona, Spain; 72 Department of Translational Medical Science, University of Naples “Federico II”, Naples, Italy; 73 CEINGE Advanced Biotechnologies, University of Naples “Federico II”, Naples, Italy; 74 Royal Free London NHS Trust, London, UK; 75 Chalmers University of Technology, Gothenburg, Sweden; 76 University of Gothenburg, Gothenburg, Sweden; 77 University of Oslo, Oslo, Norway; 78 Skaraborg Hospital, Lidköping, Sweden; 79 University of Southampton, Southampton, UK; 80 Landspitali University Hospital, Reykjavik, Iceland; 81 Vilnius University, Vilnius, Lithuania; 82 Pediatric Hospital Bambino Gesu, Rome, Italy; 83 Medical University of Lodz, Lodz, Poland; 84 University of Athens, Athens, Greece; 85 Sofia Children’s University Hospital, Madrid, Spain; 86 Emma Children’s Hospital, Amsterdam, The Netherlands; 87 Federal University of Paraná, Curitiba, Brazil; 88 Federal University of São Paulo, São Paulo, Brazil; 89 Federal University of Pernambuco, Recife, Brazil; 90 University of Santiago de Chile, Santiago, Chile; 91 University of Murcia, Murcia, Spain; 92 Children’s Hospital Srebrnjak, Zagreb, Croatia; 93 University Clinic of Respiratory and Allergic Diseases Golnik, Golnik, Slovenia; 94 University of Basel, Basel, Switzerland; 95 University Hospital Southampton NHS Foundation Trust, Southampton, UK; 96 St Mary’s Hospital, Newport, UK; 97 University of Aston, Birmingham, UK; 98 Medical Biochemistry, Faculty of Medicine, Kasr Alainy Hospital, Cairo University, Cairo, Egypt; 99 Umeå University, Umeå, Sweden; 100 Gothenburg University, Gothenburg, Sweden; 101 Department of Pediatrics, Technical University of Munich, Munich, Germany; 102 Department of Pediatrics St. Hedwig, St. Josef’s Hospital, Freiburg, Germany; 103 Department of Pediatrics and Adolescent Medicine, Johannes Gutenberg University Medical Centre, Mainz, Germany; 104 Department of Pediatrics, Heinrich-Heine-University, Düsseldorf, Germany; 105 Institute of Social Medicine, Epidemiology and Health Economics, Charité-Universitätsmedizin Berlin, Berlin, Germany; 106 Institute of Clinical Epidemiology and Biometry, University of Würzburg, Würzburg, Germany; 107 Division of Immunopathology, Department of Pathophysiology and Allergy Research, Center of Pathophysiology, Infectiology and Immunology, Medical University of Vienna, Vienna, Austria; 108 Department of Clinical and Molecular Medicine, Sapienza University of Rome, S. Andrea University Hospital, Rome, Italy; 109 Department of Pediatrics and Adolescent Medicine, University Medicine Mainz, Mainz, Germany; 110 Department of Pediatrics, University of Düsseldorf, Düsseldorf, Germany; 111 Department of Molecular Biosciences, The Wenner-Gren Institute, Stockholm University, Stockholm, Sweden; 112 Max-Delbrück-Center for Molecular Medicine, Berlin, Germany; 113 Charité University Medical Center, Berlin, Germany; 114 St George’s, University of London, London, UK; 115 University of Bern, Bern, Switzerland; 116 University Hospital Schleswig-Holstein, Kiel, Germany; 117 Inserm, Paris, France; 118 Université Paris Diderot, Paris, France; 119 University of Groningen, Groningen, The Netherlands; 120 Ludwig Maximilians University, Munich, Germany; 121 University of Western Australia, Perth, Australia; 122 University of Melbourne, Melbourne, Australia; 123 The Children’s Hospital of Philadelphia, Philadelphia, PA USA; 124 University Children’s Hospital Regensburg, Regensburg, Germany; 125 University of Bristol, Bristol, UK; 126 University Medicine and Ernst-Moritz-Arndt-University Greifswald, Greifswald, Germany; 127 Erasmus University Medical Center, Rotterdam, The Netherlands; 128 Swiss Tropical and Public Health Institute and the University of Basel, Basel, Switzerland; 129 Medical University, Innsbruck, Austria; 130 Siberian State Medical University, Tomsk, Russia; 131 Research Institute of Medical Genetics, Tomsk, Russia; 132 Ontario Institute for Cancer Research, Toronto, Canada; 133 Centre de santé et de services sociaux de Chicoutimi, Saguenay, Canada; 134 QIMR Berghofer Medical Research Institute, Brisbane, Australia; 135 University of Sydney, Sydney, Australia; 136 Murdoch Children’s Research Institute, Melbourne, Australia; 137 Sir Charles Gairdner Hospital, Perth, Australia; 138 University of Pennsylvania, Philadelphia, PA USA; 139 Temple University, Philadelphia, PA USA; 140 Christian-Albrechts-University, Kiel, Germany; 141 Helmholtz Zentrum München, Oberschleißheim, Germany; 142 Albert Ludwigs University, Freiburg, Germany; 143 University of Cologne, Cologne, Germany; 144 University of Würzburg, Würzburg, Germany; 145 University of Bonn, Bonn, Germany; 146 Research Centre Jülich, Jülich, Germany; 147 University of Queensland, Brisbane, Australia; 148 University Medicine Greifswald, Greifswald, Germany; 149 Max Planck Institute for Molecular Genetics, Berlin, Germany; 150 Université du Québec à Chicoutimi, Saguenay, Canada; 151 Ulm University, Ulm, Germany; 152 Sachs’ Children’s Hospital, Stockholm, Sweden; 153 Allergy Section, Hospital General Universitario Alicante, Alicante, Spain; 154 Universidad Miguel Hernández, Alicante, Spain; 155 Pediatrics Service, Hospital General Universitario Alicante, Alicante, Spain; 156 Preventive Service, Hospital General Universitario Alicante, Alicante, Spain; 157 Pediatric Allergy Department, St Thomas’ Hospital, London, UK; 158 Macclesfield District General Hospital, NHS England, Altrincham, UK; 159 Protectimmun GmbH, Gelsenkirchen, Germany; 160 Division of Innate Immunity, Research Center Borstel, Borstel, Airway Research Center North, Member of the German Center for Lung Research (DZL), Gießen, Germany; 161 Innlandet Hospital Trust, Elverum, Norway; 162 Oslo University Hospital, Oslo, Norway; 163 Institute of Clinical Medicine, University of Oslo, Oslo, Norway; 164 Department of Pediatrics, Division of Pneumonology, Immunology and Intensive Care Medicine incl. Rescue Center, Charité University Hospital, Berlin, Germany; 165 Meda, Bad Homburg, Germany; 166 Allergy and Asthma Associates of Southern California, Mission Viejo, CA USA; 167 Clinical Research Department, FAES FARMA SA, Leioa, Bizkaia, Spain; 168 Drug Modeling and Consulting, Dynakin SL, Bilbao, Spain; 169 Centro “Io e l’Asma”, Spedali Civili, Brescia, Italy; 170 Center of Pediatrics and Child Health, Institute of Human Development, University of Manchester, Manchester, UK; 171 Department of Pediatric Immunology, Royal Manchester Children’s Hospital, Central Manchester University Hospitals NHS Trust, Manchester, UK; 172 Laboratory of Experimental Physiology, Aristotle University of Thessaloniki, Thessaloniki, Greece; 173 INEGI-Institute of Science and Innovation in Mechanical Engineering and Industrial Management, Porto, Portugal; 174 National Institute of Health, Porto, Portugal; 175 Epidemiology Research Unit, Institute of Public Health (EPIUnit), University of Porto, Porto, Portugal; 176 Faculty of Medicine, University of Porto, Centro Hospitalar São João, Porto, Portugal; 177 Faculty of Nutrition and Food Sciences, University of Porto, Porto, Portugal; 178 Hospital Clínic de Barcelona, Barcelona, Spain; 179 Hospital Quirón, Barcelona, Spain; 180 Unitat de Rinologia i Clinica de l’Olfacte, Servei d’Otorinolaringologia, Hospital Clínic de Barcelona, Barcelona, Spain; 181 Sección de Rinología, UGC ORL, Hospital de Jerez, Cadiz, Spain; 182 Hospital Universitario Arnau de Vilanova, Valencia, Spain; 183 Hospital de Basurto, Bilbao, Spain; 184 Hospital Clínico de Salamanca, Salamanca, Spain; 185 Clínica Universitaria de Navarra, Pamplona, Spain; 186 Fundación Jimenez Díaz, Madrid, Spain; 187 CEDOC, NOVA Medical School, UNL, Lisbon, Portugal; 188 CUF Descobertas Hospital, Lisbon, Portugal; 189 UniversityCollege London, Center for Rheumatology, London, UK; 190 Instituto Português do Sangue e da Transplantação, Lisbon, Portugal; 191 Department of Pediatric Allergy, Mayıs Unıversity, Samsun, Turkey; 192 Department of Neonatology, Mayıs Unıversity, Samsun, Turkey; 193 Allergic Pediatric Unit, KAT General Hospital, Athens, Greece; 194 Clinical Biochemistry Department, KAT General Hospital, Athens, Greece; 195 Pediatric Clinic, University Hospital “Alexandrovska”, Sofia, Bulgaria; 196 Kazan State Medical University, Kazan, Russia; 197 Second Pediatric Clinic, Aglaia Kyriakou Children’s Hospital, Athens, Greece; 198 Department of Dermatology, Salford Royal Hospital, Salford, UK; 199 Department of Dermatology, Agia Sophia, Children’s Hospital, Athens, Greece; 200 Department of Medical Microbiology, Medical School of Athens, Athens, Greece; 201 Astrid Lindgren Children’s Hospital, Karolinska University Hospital, Stockholm, Sweden; 202 Mead Johnson Pediatric Nutrition Institute, Evansville, IN USA; 203 Private Practice, Boulogne, France; 204 Boston Children’s Hospital, Boston, MA USA; 205 Pediatric Department, Hamad Hospital, Doha, Qatar; 206 Cork University Hospital, Cork, Ireland; 207 Bon Secours Hospital, Cork, Ireland; 208 University College Cork, Cork, Ireland; 209 North Cumbria University Hospitals, Whitehaven, UK; 210 Great North Children’s Hospital, Newcastle upon Tyne, UK; 211 University of Michigan, Ann Arbor MI, USA; 212 University of Portsmouth, Portsmouth, UK; 213 Pediatric Pulmonology and Allergy, Annaba, Algeria; 214 Pharmacy Department, Constantine, Algeria; 215 University Hospital, Annaba, Algeria; 216 Pediatric Allergy Department, Hospital Sant Joan de Déu, Universitat de Barcelona, Barcelona, Spain; 217 Ysbyty Gwynedd, Bangor and University Hospital of Wales, Cardiff, UK; 218 Clínica Juaneda, Palma de Mallorca, Spain; 219 Clínica Juaneda, Cadiz, Spain; 220 Clínica Juaneda, Valencia, Spain; 221 Clínica Juaneda, Madrid, Spain; 222 Clínica Juaneda, Barcelona, Spain; 223 Clínica Juaneda, Granada, Spain; 224 University of Southampton, Dublin, Ireland; 225 Pediatric Assessment Unit, Our Lady of Lourdes Hospital, Drogheda, Ireland; 226 Department of Allergy, University Hospital “Virgen del Rocío”, Seville, Spain; 227 Hospital Clinico Universidad de Chile, Santiago, Chile; 228 Hospital Carlos Van Buren, Valparaiso, Chile; 229 Allergy Clinic, Copenhagen University Hospital-Gentofte, Copenhagen, Denmark; 230 Carol Davila University of Medicine and Pharmacy, Bucharest, Romania; 231 Grigore Alexandrescu Clinical Emergency Hospital for Children, Bucharest, Romania; 232 Hospital de Santa Maria-Centro Hospitalar Lisboa Norte, Lisbon, Portugal; 233 Department of Pediatrics, Pediatric Allergy-Immunology Unit, Kayseri Education and Research Hospital, Kayseri, Turkey; 234 Special Hospital Sokobanja, Sokobanja, Serbia; 235 Faculty of Medicine, University Children Hospital, Belgrade, Serbia; 236 Department of Allergology, Rheumatology and Clinical Immunology, University Children’s Hospital, University Medical Center, Ljubljana, Slovenia; 237 General Hospital in Slovenj Gradec, Slovenj Gradec, Slovenia; 238 General Hospital in Celje, Celje, Slovenia; 239 General Hospital in Šempeter, Šempeter, Slovenia; 240 General Hospital in Trbovlje, Trbovlje, Slovenia; 241 General Hospital in Izola, Izola, Slovenia; 242 Allergologic Pediatric Ambulance, Maribor, Slovenia; 243 General Hospital in Novo Mesto, Novo Mesto, Slovenia; 244 Department of Pediatrics, Children’s Hospital, Roubaix, France; 245 Allergy Vigilance Network, Vandoeuvre les Nancy, France; 246 Pediatric Pulmonology and Allergy Department, Pôle Enfant, Hôpital Jeanne de Flandre, CHRU de Lille and Université Nord de France, Lille, France; 247 Department of Internal Medicine, Immunology and Allergology, Jean Monnet Hospital, Epinal, France; 248 Hospital de Terrassa, Terrassa, Spain; 249 Bial Arístegui, Bilbao, Spain; 250 Department of Pediatric Allergy and Immunology, Ankara Child Health and Diseases Hematology-Oncology Training and Research Hospital, Ankara, Turkey; 251 Centro Hospitalar do Médio Ave, Vila Nova de Fa, Portugal; 252 Medical School, University College London, London, UK; 253 The Royal Free NHS London Foundation Trust, London, UK; 254 Immuno-flow s.r.o., Na Homolce Hospital, Prague, Czech Republic; 255 Faculty Hospital Motol, Kolin Hospital, Prague, Czech Republic; 256 Allergology Litomysl, Litomysl, Czech Republic; 257 Immunia, Prague, Czech Republic; 258 Thermofisher Scientific, ImmunoDiagnostics, Uppsala, Sweden; 259 Karolinska Institutet and Karolinska University Hospital and Center for Allergy Research, Stockholm, Sweden; 260 Södersjukhuset, Karolinska Institutet and Sachs Children’s Hospital, Stockholm, Sweden; 261 Hospital Universitario Severo Ochoa, Madrid, Spain; 262 Hospital de Villalba, Madrid, Spain; 263 Application Department Diater, Madrid, Spain; 264 Aristotle University of Thessaloniki, Hippokrateio General Hospital, Thessaloniki, Greece; 265 Aristotle University of Thessaloniki, Papageorgiou General Hospital, Thessaloniki, Greece; 266 Aristotle University of Thessaloniki, Thessaloniki, Greece; 267 Derbyshire Children’s Hospital, Royal Derby Hospital, Nottingham, UK; 268 Faculty of Medical Science, Kragujevac, Serbia; 269 Allergy Unit, Khonkaen Ram Hospital, Khonkaen, Thailand; 270 Department of Oral and Maxillofacial Surgery, KKU, Khonkaen, Thailand; 271 Department of Psychoneuropharmacology, Centre for Research and Development MEDINET, Krakow, Poland; 272 Department and Laboratory of Magnetic Resonance, Voxel SA, Krakow, Poland; 273 Allergy Research Center, Shiraz University of Medical Sciences, Shiraz, Iran; 274 Letterkenny General Hospital, Letterkenny, Ireland; 275 Midland Regional Hospital, Portlaoise, Ireland; 276 Acquired Immunodeficiency Research Center, Isfahan University of Medical Sciences, Isfahan, Iran; 277 Pediatric Department, Isfahan University of Medical Sciences, Isfahan, Iran; 278 Heart Surgery Department, Isfahan University of Medical Sciences, Isfahan, Iran; 279 Pathology Department, Isfahan University of Medical Science, Isfahan, Iran; 280 Pediatric Department, Dr. von Hauner Children’s Hospital, Ludwig Maximilians-University, Munich, Germany; 281 College of Medicine, University of Tikrit, Tikrit, Iraq; 282 Mechnikov Research Institute of Vaccines and Sera, Moscow, Russia; 283 Russian National Research Medical University, Moscow, Russia; 284 Royal Free Hospital, London, UK; 285 Department of Pediatrics, Gastroenterology and Allergology, Medical University of Bialystok, Bialystok, Poland; 286 International Medical University Clinical School Seremban, Seremban, Malaysia; 287 School of Epidemiology, Pubic Health and Preventive Medicine, University of Ottawa, Ottawa, Canada; 288 Şişli Etfal Research and Training Hospital, Istanbul, Turkey; 289 Division of Pharmacoepidemiology & Clinical Pharmacology, Utrecht Institute for Pharmaceutical Sciences, Utrecht University, Utrecht, The Netherlands; 290 Population Pharmacogenetics Group, Biomedical Research institute, University of Dundee, Ninewells Hospital and Medical School, Dundee, UK; 291 Channing Division of Network Medicine, Department of Medicine, Brigham and Women’s Hospital and Harvard Medical School, Boston, MA USA; 292 Department of Pediatrics, Şişli Hamidiye Etfal Research and Training Hospital, Istanbul, Turkey; 293 UHC, Tirana, Albania; 294 Allergology Department, University of Medicine, Tirana, Albania; 295 Allergy and Clinical Immunology Department, “At Luigji Monti” Polyclinic, “Our Lady of Good Counsel” University, Tirana, Albania; 296 Pulmonary Disease Department, University Hospital for Lung Disease, Tirana, Albania; 297 Allergy and Clinical Immunology Department, Faculty of Medicine, Tirana University, Tirana, Albania; 298 Imagery Department, “At Luigji Monti” Polyclinic, “Our Lady of Good Counsel” University, Tirana, Albania; 299 Shiraz University of Medical Sciences, Shiraz, Iran; 300 Fundacion Valle del Lili, Cali, Colombia; 301 Department of Pediatric Allergy and Immunology, Faculty of Medicine, Adana Education and Research Hospital, Başkent University, Adana, Turkey; 302 Department of Otolaryngology, Faculty of Medicine, Adana Education and Research Hospital, Başkent University, Adana, Turkey; 303 Department of Head and Neck Surgery, Faculty of Medicine, Adana Education and Research Hospital, Başkent University, Adana, Turkey; 304 Sylvana Research Associates, San Antonio, TX USA; 305 Allergy and Asthma Associates, Colorado Springs, CO USA; 306 Aranyklinika Egészségügyi és Innovációs Kft., Szeged, Hungary; 307 INAER-Investigaciones en Alergia y Enfermedades Respiratorias, Buenos Aires, Argentina; 308 Children Department, Zala Megyei Kórház, Zalaegerszeg, Hungary; 309 Allergy and Lung Diseases, SPZOZ Uniwersytecki Szpital, Lodz, Poland; 310 Departamento de Pediatría, Hospital Universitario Dr. Peset, Valencia, Spain; 311 Clinical Research Department, FAES FARMA S. A., Leioa, Bizkaia, Spain; 312 University Medical Center Hamburg-Eppendorf, Hamburg, Germany; 313 Stallergenes GmbH, Kamp-Lintfort, Germany; 314 Stallergenes, Antony, France; 315 Shionogi & Co. Ltd, Osaka, Japan; 316 Stallergenes Germany GmbH, Kamp-Lintfort, Germany; 317 PediatricsDepartment, Hospital Professor Doutor Fernando Fonseca, Amadora, Portugal; 318 Pediatrics Department, Hospital de Dona Estefania, Lisbon, Portugal; 319 Children’s Hospital, Clinical Center Nis, Faculty of Medicine, University of Nis, Nis, Serbia; 320 Children’s Hospital for Lung Diseases and Tuberculosis, Medical Center, Nis, Serbia; 321 Saint-Petersburg State Medical University, Saint-Petersburg, Russia; 322 KBC Dr. Dragisa Misovic, Children’s Hospital for Respiratory Diseases, Belgrade, Serbia; 323 Clinic for Pulmonary Diseases, Clinical Centre of Serbia, Belgrade, Serbia; 324 Hans Christian Andersen Children’s Hospital, Odense University Hospital, Odense, Denmark; 325 University College London, London, UK; 326 Department of Dermatology and Allergy Center, Odense University Hospital, University of Southern Denmark, Odense, Denmark; 327 Hans Christian Andersen Children’s Hospital, Odense University Hospital, University of Southern Denmark, Odense, Denmark; 328 Department for Dermatology and Allergy, Clinical Research Center for Hair and Skin Science, Charité-Universitätsmedizin Berlin, Berlin, Germany; 329 Department of Medical Statistics and Clinical Epidemiology, Charité-Universitätsmedizin Berlin, Berlin, Germany; 330 Department of Obstetrics, Charité Universitätsmedizin Berlin, Berlin, Germany; 331 Department of Obstetrics, St. Joseph Clinic Berlin Tempelhof, Berlin, Germany; 332 University of Otago Wellington, Wellington, New Zealand; 333 GSTT, London, UK; 334 UMC, Beatrix Children’s Hospital, Groningen, The Netherlands; 335 Fondazione IRCCS ‘‘Ca’Granda’’ Ospedale Maggiore, Milan, Italy; 336 University of Milan, Milan, Italy; 337 Spedali Civili, Brescia, Italy; 338 University Children’s Hospital, Zurich, Switzerland; 339 Schwarzach Hospital, Salzburg, Austria; 340 Danone Research, Utrecht, The Netherlands; 341 Private Practice, Leipzig, Germany; 342 Department of Pediatrics, Allergy Unit, University of Messina, Messina, Italy; 343 Tokyo University of Agriculture and Technology, Tokyo, Japan; 344 Graduate School of Medicine, Chiba University, Chiba, Japan; 345 Chiba University Hospital, Chiba, Japan; 346 School of Medicine, Tokai University, Tokyo, Japan; 347 Department of Pediatrics, Faculty of Medicine, University of Brawijaya, Saiful Anwar Hospital, Malang, Indonesia; 348 Department of Biology, University of Naples “Federico II”, Naples, Italy; 349 Department of Pharmacy, University of Naples “Federico II”, Naples, Italy; 350 Charité University Hospital, Berlin, Germany; 351 SymbioPharm GmbH, Herborn, Germany; 352 Biostatistics, Leipzig, Germany; 353 TG Medical Services, Berlin, Germany; 354 Children’s Hospital, Children’s Center Bethel, EvKB, Bielefeld, Germany; 355 Allergy Center Ruhr, Ruhr-University Bochum, Bochum, Germany; 356 Atrium Medisch Centrum, Heerlen, The Netherlands; 357 Servicio Alergia, Hospital Universitario Quirón Dexeus, Barcelona, Spain; 358 Hospital La Paz, Madrid, Spain; 359 General Hospital, Valencia, Spain; 360 Hospital Gregorio Marañón, Madrid, Spain; 361 Hospital San Juan de Dios, Barcelona, Spain; 362 Hospital Carlos Haya, Málaga, Spain; 363 Hospital Vall Hebrón, Barcelona, Spain; 364 Hospital Fundación Althaia San Juan de Dios, Barcelona, Spain; 365 Hospital de Cruces, Bilbao, Spain; 366 Hospital Severo Ochoa Leganés, Madrid, Spain; 367 Leeds Children’s Hospital, Leeds, UK; 368 St. Mary’s Hospital, London, UK; 369 ThermoFisher Scientific, Uppsala, Sweden; 370 Immunology Laboratory, King’s College Hospital, London, UK; 371 St. Thomas’ Hospital, London, UK; 372 Sachs Children and Youth Hospital, Stockholm, Sweden; 373 Milton Keynes University Hospital NHS Foundation Trust, Milton Keynes, UK; 374 Sheffield Children’s Hospital, Sheffield, UK; 375 Centro Hospitalar do Médio Ave, Santo Tirso, Portugal; 376 Pediatric Department, Unit of Allergy and Immunology in Evolutive Age, Clinical and Experimental Medicine, University of Parma, Parma, Italy; 377 Allergology Service, San Carlo Clinic, Paderno Dugnano, Italy; 378 Pediatric Department and Pediatric Allergology Unit, Sandro Pertini Hospital, Rome, Italy; 379 Pediatric Unit, Department for Mother and Child, Ramazzini Hospital, Carpi, Italy; 380 Pediatric Unit, Department of Medical and Surgical Sciences, University of Bologna, Bologna, Italy; 381 Centro per la Prevenzione, Diagnosi e Cura delle Malattie Allergiche e Otorinolaringoiatriche, Rome, Italy; 382 Operative Complex Unit of Pediatrics and Neonatal Patology, Mazzoni Hospital, Ascoli Piceno, Italy; 383 Pediatric Unit, Mazzoni Hospital, Ascoli Piceno, Italy; 384 Pediatric Department, La Sapienza University, Rome, Italy; 385 Azienda Sanitaria Locale Salerno, Salerno, Italy; 386 Department of Pediatric Pneumology and Immunology, Charité Medical University, Berlin, Germany; 387 Children University Hospital, Temple Street, Dublin, Ireland; 388 Saint Petersburg State Pediatric Medical University, St. Petersburg, Russia; 389 Nikiforov Russian Center of Emergency and Radiation Medicine, St. Petersburg, Russia; 390 Pediatric Cilinic, University Hospital, Sofia, Bulgaria; 391 National Genetic Laboratory, Obstetric and Genecology Hospital “Maichin dom”, Sofia, Bulgaria; 392 Hygeia Hospital, Tirana, Albania; 393 Allergy Unit, 2nd Pediatric Clinic, University of Athens, Athens, Greece; 394 Intensive Care Unit, Penteli’s Children Hospital, Athens, Greece; 395 Pediatric Department, Penteli’s Children Hospital, Athens, Greece; 396 Erasmus MC, Rotterdam, The Netherlands; 397 Laboratory of Clinical Immunology, KU Leuven, Leuven, Belgium; 398 Laboratory of Pneumology, KU Leuven, Leuven, Belgium; 399 Flemish Swimming Federation, Merelbeke, Belgium; 400 Academic Centre for General Practitioners, KU Leuven, Leuven, Belgium; 401 Sport Medical Advice Centre, UZ Leuven, Leuven, Belgium; 402 Laboratory of Pediatric Immunology, KU Leuven, Leuven, Belgium; 403 Department of Women, Child and Youth, Local Health Unit of Matosinhos, Matosinhos, Portugal; 404 Respiratory, Critical Care and Anaesthesia Section, University College London, Institute of Child Health, London, UK; 405 Grouping of Matosinhos Health Centers, Local Health Unit of Matosinhos, Matosinhos, Portugal; 406 Department of Clinical Epidemiology, Predictive Medicine and Public Health, School of Medicine, University of Porto, Porto, Portugal; 407 Children’s Hospital, St. Joseph Hospital, Bochum, Germany; 408 Allergy Center Charité, Charité University Medicine, Berlin, Germany; 409 Hospital General Universitario de Alicante, Alicante, Spain; 410 Complejo Hospitalario de Navarra, Pamplona, Spain; 411 University Clinic of Navarra, Pamplona, Spain; 412 Mayo Clinic, Rochester, NY USA; 413 General and Teaching Hospital Izola, Izola, Slovenia; 414 University Children’s Hospital, Ljubljana, Slovenia; 415 Hospital Central do Funchal, Funchal, Portugal; 416 Serviço de Imunoalergologia, Centro Hospitalar de São João E.P.E., Porto, Portugal; 417 Serviço de Anestesiologia, Centro Hospitalar de São João E.P.E., Porto, Portugal; 418 Application Department, DIATER, Madrid, Spain; 419 St. Thomas’ Hospital, London, UK; 420 Department of Pneumology, Allergy and Cystic Fibrosis, Pediatric Allergy Unit, Vall d’Hebron University Hospital, Barcelona, Spain; 421 “Giorgio Prodi” Cancer Research Center, University of Bologna, Bologna, Italy; 422 Dermatology Unit, Department of Experimental, Diagnostic and Specialty Medicine, University of Bologna, Bologna, Italy; 423 I.M. Sechenov First Moscow State Medical University, Moscow, Russia

## Abstract

WORKSHOP 4: Challenging clinical scenarios (CS01–CS06)

CS01 Bullous lesions in two children: solitary mastocytoma

S. Tolga Yavuz, Ozan Koc, Ali Gungor, Faysal Gok

CS02 Multi-System Allergy (MSA) of cystic fibrosis: our institutional experience

Jessica Hawley, Christopher O’Brien, Matthew Thomas, Malcolm Brodlie, Louise Michaelis

CS03 Cold urticaria in pediatric age: an invisible cause for severe reactions

Inês Mota, Ângela Gaspar, Susana Piedade, Graça Sampaio, José Geraldo Dias, Miguel Paiva, Mário Morais-Almeida

CS04 Angioedema with C1 inhibitor deficiency in a girl: a challenge diagnosis

Cristina Madureira, Tânia Lopes, Susana Lopes, Filipa Almeida, Alexandra Sequeira, Fernanda Carvalho, José Oliveira

CS05 A child with unusual multiple organ allergy disease: what is the primer?

Fabienne Gay-Crosier

CS06 A case of uncontrolled asthma in a 6-year-old patient

Ioana-Valentina Nenciu, Andreia Florina Nita, Alexandru Ulmeanu, Dumitru Oraseanu, Carmen Zapucioiu

ORAL ABSTRACT SESSION 1: Food allergy (OP01–OP06)

OP01 Food protein-induced enterocolitis syndrome: oral food challenge outcomes for tolerance evaluation in a Pediatric Hospital

Adrianna Machinena, Olga Domínguez Sánchez, Montserrat Alvaro Lozano, Rosa Jimenez Feijoo, Jaime Lozano Blasco, Mònica Piquer Gibert, Mª Teresa Giner Muñoz, Marcia Dias da Costa, Ana Maria Plaza Martín

OP02 Characteristics of infants with food protein-induced enterocolitis syndrome and allergic proctocolitis

Ebru Arik Yilmaz, Özlem Cavkaytar, Betul Buyuktiryaki, Ozge Soyer, Cansin Sackesen

OP03 The clinical and immunological outcomes after consumption of baked egg by 1–5 year old egg allergic children: results of a randomised controlled trial

MerrynNetting, Adaweyah El-Merhibi, Michael Gold, PatrickQuinn, IrmeliPenttila, Maria Makrides

OP04 Oral immunotherapy for treatment of egg allergy using low allergenic, hydrolysed egg

Stavroula Giavi, Antonella Muraro, Roger Lauener, Annick Mercenier, Eugen Bersuch, Isabella M. Montagner, Maria Passioti, Nicolò Celegato, Selina Summermatter, Sophie Nutten, Tristan Bourdeau, Yvonne M. Vissers, Nikolaos G. Papadopoulos

OP05 Chemical modification of a peanut extract results in an increased safety profile while maintaining efficacy

Hanneke van der Kleij, Hans Warmenhoven, Ronald van Ree, Raymond Pieters, Dirk Jan Opstelten, Hans van Schijndel, Joost Smit

OP06 Administration of the yellow fever vaccine in egg allergic children

Roisin Fitzsimons, Victoria Timms, George Du Toit

ORAL ABSTRACT SESSION 2: Asthma (OP07–OP12)

OP07 Previous exacerbation is the most important risk factor for future exacerbations in school-age children with asthma

S. Tolga Yavuz, Guven Kaya, Mustafa Gulec, Mehmet Saldir, Osman Sener, Faysal Gok

OP08 Comparative study of degree of severity and laboratory changes between asthmatic children using different acupuncture modalities

Nagwa Hassan, Hala Shaaban, Hazem El-Hariri, Ahmed Kamel Inas E. Mahfouz

OP09 The concentration of exhaled carbon monoxide in asthmatic children with different controlled stadium

Papp Gabor, Biro Gabor, Kovacs Csaba

OP10 Effect of vitamin D3 supplementation during pregnancy on risk of persistent wheeze in the offspring: a randomised clinical trial

Bo Chawes, Klaus Bønnelykke, Jakob Stokholm, Lene Heickendorff, Susanne Brix, Morten Rasmussen, Hans Bisgaard

OP11 Lung function development in childhood

Henrik Wegener Hallas, Bo Chawes, Lambang Arianto, Hans Bisgaard

OP12 Is the effect of maternal and paternal asthma different in female and male children before puberty?

Maike Pincus, Thomas Keil, Andreas Reich, Ulrich Wahn, Susanne Lau, Linus Grabenhenrich

ORAL ABSTRACT SESSION 3: Epidemiology—genetics (OP13–OP18)

OP13 Lifestyle is associated with incidence and category of allergen sensitisation: the ALADDIN birth cohort

Sara Fagerstedt, Helena Marell Hesla, Emelie Johansson, Helen Rosenlund, Axel Mie, Annika Scheynius, Johan Alm

OP15 Maternal filaggrin mutations increase the risk of atopic dermatitis in children: an effect independent of mutation inheritance

Jorge Esparza-Gordillo, Anja Matanovic, Ingo Marenholz, Anja Bauerfeind, Klaus Rohde, Katja Nemat, Min-Ae Lee-Kirsch, Magnus Nordenskjöld, Marten C. G. Winge, Thomas Keil, Renate Krüger, Susanne Lau, Kirsten Beyer, Birgit Kalb, Bodo Niggemann, Norbert Hübner, Heather J. Cordell, Maria Bradley, Young-Ae Lee

OP16 Allergic multimorbidity of asthma, rhinitis and eczema in the first 2 decades of the German MAS birth cohort

Thomas Keil, Hannah Gough, Linus Grabenhenrich, Dirk Schramm, Andreas Reich, John Beschorner, Antje Schuster, Carl-Peter Bauer, Johannes Forster, Fred Zepp, Young-Ae Lee, Renate Bergmann, Karl Bergmann, Ulrich Wahn, Susanne Lau

OP17 Childhood anaphylaxis: a growing concern

Filipe Benito Garcia, Inês Mota, Susana Piedade, Ângela Gaspar, Natacha Santos, Helena Pité, Mário Morais-Almeida

OP18 Indoor exposure to molds and dampness in infancy and its association to persistent atopic dermatitis in school age. Results from the Greek ISAAC II study

Athina Papadopoulou, Despina Mermiri, Elpida Xatziagorou, Ioannis Tsanakas, Stavroula Lampidi, Kostas Priftis

ORAL ABSTRACT SESSION 4: Pediatric rhinitis—immunotherapy (OP19–OP24)

OP19 Associations between residential greenness and childhood allergic rhinitis and aeroallergen sensitisation in seven birth cohorts

Elaine Fuertes, Iana Markevych, Gayan Bowatte, Olena Gruzieva, Ulrike Gehring, Allan Becker, Dietrich Berdel, Michael Brauer, Chris Carlsten, Barbara Hoffmann, Anita Kozyrskyj, Caroline Lodge, Göran Pershagen, Alet Wijga, Heinrich Joachim

OP20 Full symptom control in pediatric patients with allergic rhinitis and asthma: results of a 2-year sublingual allergen immunotherapy study

Zorica Zivkovic, Ivana Djuric-Filipovic, Jasmina Jocić-Stevanovic, Snežana Zivanovic

OP21 Nasal epithelium of different ages of atopic subjects present increased levels of oxidative stress and increased cell cytotoxicity upon rhinovirus infection

Styliani Taka, Dimitra Kokkinou, Aliki Papakonstantinou, Panagiota Stefanopoulou, Anastasia Georgountzou, Paraskevi Maggina, Sofia Stamataki, Vassiliki Papaevanggelou, Evangelos Andreakos, Nikolaos G. Papadopoulos

OP22 Cluster subcutaneous immunotherapy schedule: tolerability profile in children

Monica Piquer Gibert, Montserrat Alvaro Lozano, Jaime Lozano Blasco, Olga Domínguez Sánchez, Rosa Jiménez Feijoo, Marcia Dias da Costa, Mª Teresa Giner Muñoz, Adriana Machinena Spera, Ana Maria Plaza Martín

OP23 Rhinitis as a risk factor for asthma severity in 11-year old children: population-based cohort study

Matea Deliu, Danielle Belgrave, Angela Simpson, Adnan Custovic

OP24 The Global Lung Function Initiative equations in airway obstruction evaluation of asthmatic children

João Gaspar Marques, Pedro Carreiro-Martins, Joana Belo, Sara Serranho, Isabel Peralta, Nuno Neuparth, Paula Leiria-Pinto

POSTER DISCUSSION SESSION 1: Food allergy (PD01–PD05)

PD01 Allergen-specific humoral and cellular responses in children who fail egg oral immunotherapy due to allergic reactions

Marta Vazquez-Ortiz, Mariona Pascal, Ana Maria Plaza, Manel Juan

PD02 FoxP3 epigenetic features in children with cow milk allergy

Lorella Paparo, Rita Nocerino, Rosita Aitoro, Ilaria Langella, Antonio Amoroso, Alessia Amoroso, Carmen Di Scala, Roberto Berni Canani

PD04 Combined milk and egg allergy in early childhood: let them eat cake?

Santanu Maity, Giuseppina Rotiroti, Minal Gandhi

PD05 Introduction of complementary foods in relation to allergy and gut microbiota in farm and non-farm children

Karin Jonsson, Annika Ljung, Bill Hesselmar, Ingegerd Adlerbert, Hilde Brekke, Susanne Johansen, Agnes Wold, Ann-Sofie Sandberg

POSTER DISCUSSION SESSION 2: Asthma and wheeze (PD06–PD16)

PD06 The association between asthma and exhaled nitric oxide is influenced by genetics and sensitisation

Björn Nordlund, Cecilia Lundholm, Villhelmina Ullemar, Marianne van Hage, Anne Örtqvist, Catarina Almqvist

PD09 Prevalence patterns of infant wheeze across Europe

Anna Selby, Kate Grimshaw, Thomas Keil, Linus Grabenhenrich, Michael Clausen, Ruta Dubakiene, Alessandro Fiocchi, Marek Kowalski, Nikos Papadopoulos, Marta Reche, Sigurveig Sigurdardottir, Aline Sprikkleman, Paraskevi Xepapadaki, Clare Mills, Kirsten Beyer, Graham Roberts

PD10 Epidemiologic changes in recurrent wheezing infants

Herberto Jose Chong Neto, Gustavo Falbo Wandalsen, Ana Carolina Dela Bianca, Carolina Aranda, Nelson Augusto Rosário, Dirceu Solé, Javier Mallol, Luis García Marcos

PD13 A single nucleotide polymorphism in the GLCCI1 gene is associated with response to asthma treatment in children

IvanaBanic, Matija Rijavec, Davor Plavec, Peter Korosec, Mirjana Turkalj

PD14 Pollen induced asthma: Could small molecules in pollen exacerbate the protein-mediated allergic response?

Alen Bozicevic, Maria De Mieri, Matthias Hamburger

PD15 A qualitative study to understand how we can empower teenagers to better self-manage their asthma

Simone Holley, Ruth Morris, Frances Mitchell, Rebecca Knibb, Susan Latter, Christina Liossi, Graham Roberts

PD16 Polymorphism of endothelial nitric oxide synthase (eNOS) gene among Egyptian children with bronchial asthma

Mostafa M. M. Hassan

POSTER DISCUSSION SESSION 3: Mechanisms—Epidemiology (PD17–PD21)

PD17 Pregnancy outcomes in relation to development of allergy in a Swedish birth cohort

Malin Barman, Anna Sandin, Agnes Wold, Ann-Sofie Sandberg

PD18 Evolution of the IgE response to house dust mite molecules in childhood

Daniela Posa, Serena Perna, Carl-Peter Bauer, Ute Hoffmann, Johannes Forster, Fred Zepp, Antje Schuster, Ulrich Wahn, Thomas Keil, Susanne Lau, Kuan-Wei Chen, Yvonne Resch, Susanne Vrtala, Rudolf Valenta, Paolo Maria Matricardi

PD19 Antibody recognition of nsLTP-molecules as antigens but not as allergens in the German-MAS birth cohort

Olympia Tsilochristou, Alexander Rohrbach, Antonio Cappella, Stephanie Hofmaier, Laura Hatzler, Carl-Peter Bauer, Ute Hoffmann, Johannes Forster, Fred Zepp, Antje Schuster, RaffaeleD’Amelio, Ulrich Wahn, Thomas Keil, Susanne Lau, Paolo Maria Matricardi

PD20 Early life colonization with Lactobacilli and Staphylococcus aureus oppositely associates with the maturation and activation of FOXP3+ CD4 T-cells

Sophia Björkander, Maria A. Johansson, Gintare Lasaviciute, Eva Sverremark-Ekström

PD21 Genome-wide meta-analysis identifies 7 susceptibility loci involved in the atopic march

Ingo Marenholz, Jorge Esparza-Gordillo, Franz Rüschendorf, Anja Bauerfeind, David P. Strachan, Ben D. Spycher, Hansjörg Baurecht, Patricia Margaritte-Jeannin, Annika Sääf, Marjan Kerkhof, Markus Ege, Svetlana Baltic, Melanie C Matheson, Jin Li, Sven Michel, Wei Q. Ang, Wendy McArdle, Andreas Arnold, Georg Homuth, Florence Demenais, Emmanuelle Bouzigon, Cilla Söderhäll, Göran Pershagen, Johan C. de Jongste, Dirkje S Postma, Charlotte Braun-Fahrländer, Elisabeth Horak, Ludmila M. Ogorodova, Valery P. Puzyrev, Elena Yu Bragina, Thomas J Hudson, Charles Morin, David L Duffy, Guy B Marks, Colin F Robertson, Grant W Montgomery, Bill Musk, Philip J Thompson, Nicholas G. Martin, Alan James, Patrick Sleiman, Elina Toskala, Elke Rodriguez, Regina Fölster-Holst, Andre Franke, Wolfgang Lieb, Christian Gieger, Andrea Heinzmann, Ernst Rietschel, Thomas Keil, Sven Cichon, Markus M Nöthen, Craig E Pennell, Peter D Sly, Carsten O Schmidt, Anja Matanovic, Valentin Schneider, Matthias Heinig, Norbert Hübner, Patrick G. Holt, Susanne Lau, Michael Kabesch, Stefan Weidinger, Hakon Hakonarson, Manuel AR Ferreira, Catherine Laprise, Maxim B. Freidin, Jon Genuneit, Gerard H Koppelman, Erik Melén, Marie-Hélène Dizier, A. John Henderson, Young Ae Lee

POSTER DISCUSSION SESSION 4: Food allergy—Anaphylaxis (PD22–PD26)

PD22 Atopy patch test in food protein induced enterocolitis caused by solid food

Purificacion González-Delgado, Esther Caparrós, Fernando Clemente, Begoña Cueva, Victoria M. Moreno, Jose Luis Carretero, Javier Fernández

PD23 Watermelon allergy: a novel presentation

Kate Swan, George Du Toit

PD24 A pilot study evaluating the usefulness of a guideline template for managing milk allergy in primary care

Mudiyur Gopi, Tim Smith, Edara Ramesh, Arun Sadasivam

PD26 Efficacy and safety of cow’s milk oral immunotherapy protocol

Inês Mota, Filipe Benito Garcia, Susana Piedade, Angela Gaspar, Graça Sampaio, Cristina Arêde, Luís Miguel Borrego, Graça Pires, Cristina Santa-Marta, Mário Morais-Almeida

POSTER DISCUSSION SESSION 5: Prevention and treatment—Allergy (PD27–PD36)

PD27 Allergy-protection by the lactic acid bacterium Lactococcus lactis G121: mode-of-action as revealed in a murine model of experimental allergy

Stephanie Brand, Karina Stein, Holger Heine, Marion Kauth

PD29 The relationship between quality of life and morning salivary cortisol after acute bronchiolitis in infancy

Leif Bjarte Rolfsjord, Egil Bakkeheim, Johan Alm, Håvard Ove Skjerven, Kai-Håkon Carlsen, Jon Olav Hunderi, Teresa Løvold Berents, Petter Mowinckel, Karin C. Lødrup Carlsen

PD30 Randomised trial of the efficacy of MP29-02* compared with fluticasone propionate nasal spray in children aged ≥6 years to <12 years with allergic rhinitis

Ulrich Wahn, Ullrich Munzel, William Berger

PD31 10 mg of oral bilastine in 2 to 11 years old children has similar exposure to the adult therapeutic dose (20 mg)

Ulrich Wahn, Román Valiente, Valvanera Vozmediano, John C. Lukas, Mónica Rodríguez

PD33 Daily symptoms, nocturnal symptoms, activity limitations and reliever therapies during the three steps of IOEASMA programme: a comparison

Sebastiano Guarnaccia, Luigi Vitale, Ada Pluda, Emanuele D’Agata, Denise Colombo, Stefano Felici, Valeria Gretter, Susanna Facchetti, Gaia Pecorelli, Cristina Quecchia

PD34 Sensitisation to an inert aeroallergen in weaning rats and longstanding disease, in a sensitisation-tolerant and easily tolerisable rodent strain

George Guibas, Evangelia Spandou, Spyridon Megremis, Peter West, Nikolaos Papadopoulos

PD35 Bacterial and fungi exposure in school and allergic sensitisation in children

João Cavaleiro Rufo, Joana Madureira, Inês Paciência, Lívia Aguiar, Patrícia Padrão, Mariana Pinto, Luís Delgado, Pedro Moreira, João Paulo Teixeira, Eduardo Oliveira Fernandes, André Moreira

PD36 Comparative study of allergy rhinitis between two populations: children vs. adults

Adriana Izquierdo Dominguez, Antonio Valero, Joaquim Mullol, Alfonso Del Cuvillo, Javier Montoro, Ignacio Jauregui, Joan Bartra, Ignacio Davila, Marta Ferrer, Joaquin Sastre

POSTER VIEWING SESSION 1: Inflammation—Genetics—Immunology—Dermatology (PP01–PP09)

PP01 Immune profile in late pregnancy: immunological markers in atopic asthmaticwomen as risk factors for atopy in the progeny

Catarina Martins, Jorge Lima, Maria José Leandro, Glória Nunes, Jorge Cunha Branco, Hélder Trindade, Luis Miguel Borrego

PP02 The impact of neonatal sepsis on development of allergic diseases

Secil Conkar, Mehtap Kilic, Canan Aygun, Recep Sancak

PP03 Clinical overview of selective IgE deficiency in childhood

Athina Papadopoulou, Eleni Tagalaki, Lambros Banos, Anna Vlachou, Fotini Giannoula, Despina Mermiri

PP04 Inverse relationship between serum 25(ΟΗ) vitamin D3 and total IgE in children and adolescence

Athina Papadopoulou, Stavroula Lampidi, Marina Pavlakou, Maria Kryoni, Kostas Makris

PP05

PP06

PP07 Asthma control questionnaire and specific IgE in children

Snezhina Lazova, Guergana Petrova, Dimitrinka Miteva, Penka Perenovska

PP08 Features of chronic urticaria of adolescents

Aliya Klyucharova, Olesya Skorohodkina

PP09 Cutaneous mastocytosis in children: a clinical analysis of 8 cases in Greece

Dimitra Koumaki, Alkisti Manousaki, Maria Agrapidi, Lida Iatridou, Omima Eruk, Konstantinos Myridakis, Emmanouil Manousakis, Vasiliki Koumaki

POSTER VIEWING SESSION 2: Food allergy—Anaphylaxis (PP10–PP47)

PP10 Prognostic factors in egg allergy

Maria Dimou, Maria Ingemansson, Gunilla Hedlin

PP11 Evaluation of the efficacy of an amino acid-based formula in infants who are intolerant to extensively hydrolysed protein formula

Nitida Pastor, Delphine de Boissieu, Jon Vanderhoof, Nancy Moore, Kaitlin Maditz

PP12 Anaphylaxis and epinephrine auto-injector use: a survey of pediatric trainees

Adeli Mehdi, Shaza Elhassan, Carolin Beck, Ahmed Al-Hammadi

PP13 Anaphylaxis in children: acute management in the Emergency Department

Ioana Maris, Ronan O’Sullivan, Jonathan Hourihane,

PP14 Understanding Cumbrian schools preparedness in managing children at risk of anaphylaxis in order to provide training and support which will create healthy and safe environments for children with allergies

George Raptis, Louise Michaelis

PP15 A new valid and reliable parent and child questionnaire to measure the impact of food protein enterocolitis syndrome on children: the FPIES Quality of Life Questionnaire (FPIESQL), Parent and Child Short Form

Audrey DunnGalvin, Matthew Greenhawt, Carina Venter, Jonathan Hourihane

PP16 An in-depth case study investigation of the experiences of teenagers and young adults in growing up and living with food allergy with emphasis on coping, management and risk, support, and social and self-identity

Evelyn O’Regan, Duncan Cronin, Jonathan Hourihane, Anna O’Reilly, Audrey DunnGalvin

PP17 Cow’s milk protein allergy in Constantine. A retrospective study of 62 cases between 1996 and 2013

Foued Abdelaziz, Dounia Khelifi-Touhami, Nihad Selim, Tahar Khelifi-Touhami

PP18

PP19 Cow’s milk and egg oral immunotherapy in children older than 5 years

Pablo Merida, Ana Mª Plaza, Juan Heber Castellanos, Adrianna Machinena, Montserrat Alvaro Lozano, Jaime Lozano, Olga Dominguez, Monica Piquer, Rosa Jimenez, Mª Teresa Giner

PP20 Professionals’ awareness of management of Cow’s Milk Protein Allergy (CMPA) in North Wales Hospitals

Konstantinos Kakleas, Manohar Joishy, Wendmu Maskele, Huw R. Jenkins

PP21

PP22 Anaphylaxis: the great unknown for teachers. Presentation of a protocol for schools

Mercedes Escarrer, Agustín Madroñero, Maria Teresa Guerra, Juan Carlos Julia, Juan Carlos Cerda, Javier Contreras, Eulalia Tauler, Maria Jesus Vidorreta, Ana Rojo, Silvia Del Valle

PP23 Challenges facing children with food allergies and their parents in out of school activity sectors

Niamh Flynn

PP24 A review of food challenges at a Regional Irish Centre

Gary Foley, Carol Harmon, John Fitzsimons

PP25 The use of epinephrine in infants with anaphylaxis

Krasimira Baynova, Ávila Maria Del Robledo, Labella Marina

PP26

PP27

PP28 Mother’s psychological state predicts the expression of symptoms in food allergic children

Aaron Cortes, Alicia Sciaraffia, Angela Castillo

PP29 The correlation between sIgE towards tree nuts and birch pollen in a Danish Pediatric Allergy Clinic

Nanna Juel-Berg, Kirsten Skamstrup Hansen, Lars Kærgaard Poulsen

PP30 Food allergy in children: evaluation of parents’ use of online social media

Andreia Florina Nita, Ioana Valentina Nenciu, Adina Lazar, Dumitru Oraseanu

PP31 The impact of food allergy on quality of life: FAQLQ questionnaire

Rita Aguiar, Anabela Lopes, Maria J. Paes, Amélia S. Santos, M. A. Pereira-Barbosa

PP32 An unexpected cause of anaphylaxis: potato

Hatice Eke Gungor, Salih Uytun, Umit Murat Sahiner, Yasemin Altuner Torun

PP33 Is it clinical phenotype of allergic diseases determined by sensitisation to food?

Mirjana Zivanovic, Marina Atanasković-Marković

PP34

PP35 Prescribing adrenaline auto-injectors in children in 2014: the data from regional pediatricians

Tina Vesel, Mihaela Nahtigal, Andreja Obermayer-Temlin, Eva Šoster Križnik, Mirjana Maslar, Ruben Bizjak, Marjeta Tomšič-Matic, Sonja Posega-Devetak, Maja Skerbinjek-Kavalar, Mateja Predalič, Tadej Avčin

PP36 Who should have an adrenaline autoinjector? Adherence to the European and French guidelines among 121 allergists from the Allergy Vigilance Network

Guillaume Pouessel, Etienne Beaudouin, Anne M. Moneret-Vautrin, Antoine Deschildre, Allergy Vigilance Network

PP37 Anaphylaxis by Anacardium Occidentale

Marta Viñas, Bartolomé Borja, Nora Hernández, Mª José Castillo, Adriana Izquierdo, Marcel Ibero

PP38 Anaphylaxis with honey in a child

S. Tolga Yavuz, Ali Gungor, Betul Buyuktiryaki, Ozan Koc, Can Naci Kocabas, Faysal Gok

PP39 Evaluation of courses adopted to children on prevention, recognition and management of anaphylaxis

Tina Vesel, Mihaela Nahtigal

PP40 Symptomatic dust mites and shrimp allergy: three pediatric case reports

Filipa Almeida, Susana Lopes, Cristina Madureira, Tânia Lopes, Fernanda Carvalho

PP41 Poor identification rates of nuts by high risk individuals: a call for improved education and support for families

Camille Heming, Emily Garrett, Adam Blackstock, Santanu Maity, Rahul Chodhari

PP42 DAFALL: database of food allergies in the Czech Republic

Simona Belohlavkova, Eliska Kopelentova, Petr Visek, Ivana Setinova, Ivana Svarcova

PP43 Serological cross-reactivity between grass and wheat is not only caused by profilins and CCDs

Sigrid Sjölander, Nora Nilsson, Malin Berthold, Helena Ekoff, Gunilla Hedlin, Magnus Borres, Caroline Nilsson

PP44 Oil body associated proteins in children with nuts allergy. Allergens to consider in IgE-mediated nuts allergy

Loreto González Domínguez, Cristina Muñoz Archidona, Ana Moreira Jorge, Sergio Quevedo Teruel, Teresa Bracamonte Bermejo, Miriam Castillo Fernández, Fernando Pineda de la Losa, Luis Ángel Echeverría Zudaire

PP45

PP46 Protective effect of helicobacter pylori infection against food allergy in children

Olga Vrani, Antigone Mavroudi, Maria Fotoulaki, Maria Emporiadou, Kleomenis Spiroglou, Ioannis Xinias

PP47 Anaphylaxis pathway: A road tryp-tase to success?

Helyeh A. Sadreddini, Mia Warnes, Donna Traves

POSTER VIEWING SESSION 3: Miscellaneous (PP48–PP58)

PP48 Surveillance study on safety of SLIT in pediatric population

Ivana Djuric-Filipovic, Zorica Zivkovic, Snežana Zivanovic, Gordana Kostić, Đorđe Filipovic

PP49 Efficacy and safety of mixed mite subcutaneous immunotherapy among allergic rhinitis patients in the Northeastern Thailand

Sawapon Sittisomwong, Siripong Sittisomwong

PP50 Effect of inhaled beclomethasone or placebo on brain stem activity in a patient chronically treated with steroids: preliminary report

Zygmunt Podolec, Marcin Hartel, Daria Panek, Magdalena Podolec-Rubiś, Tomasz Banasik

PP51 Sensitisation to aeroallergens in patients with allergic rhinitis, asthma and atopic dermatitis in Shiraz, Southwestern Iran

Elham Abbasi, Mozhgan Moghtaderi

PP52 Referring a child for allergy test: how appropriate are we?

Phani Sanneerappa, Alina Deliu, Moosa Kutty, Nagabathula Ramesh

PP53 EBV lymphoproliferative disease and cardiac lymphoma in a STK4 deficient patient

Roya Sherkat, Mohammad Reza Sabri, Bahar Dehghan, Hamid Bigdelian, Nahid Raeesi, Mino Afshar, Hamid Rahimi, Christoph Klein

PP54 A case study: the effect of massive honeybees attack on various body parameters atopic girl including allergy

Mohemid Al-Jebouri

PP55 The role of TLR9, NLRP3 and proIL-1β in activation of antiviral innate immunity

Oxana A. Svitich, Daria O. Zubacheva, Dmitrii A. Potemkin, Ludmila V. Gankovskaya, Vitalii V. Zverev

PP56 Overnight pulse oximetry, as a screening tool to diagnose obstructive sleep apnoea. How effective is it?

Phani Sanneerappa, Elaine OB Doyle, Paul Gallagher, Nagabathula Ramesh

PP57 The presentation and management of acute urticaria and allergic reactions in children in a multi-ethnic, inner city Emergency Department (ED)

Sherine Dewlett, Kin Man, Minal Gandhi, James Pocock, Anna Gerrardhughes

PP58 Food allergens responsible for delayed-type sensitisation in atopy patch test in children diagnosed with autism spectrum disorder

Jolanta Wasilewska, Maciej Kaczmarski, Dariusz Lebensztejn

POSTER VIEWING SESSION 4: Asthma—Rhinitis (PP59–PP87)

PP59 Systematic review of incense as a trigger factor for asthma

Chandramani Thuraisingham, Davendralingam Sinniah

PP60 Increased risks of mood and anxiety disorders in children with asthma

Yue Chen, Xiaomei Mei

PP61

PP62 Asthma Control Test (ACT) and Pediatric Asthma Quality of Life Questionnaire (PAQLQ) association in children

Sebnem Ozdogan, Pinar Karadeniz, Durdugul Ayyildiz-Emecen, Ummuhan Oncul

PP63 Seasonal and gender variations in vitamin D levels in children with asthma and its association with pulmonary function tests

Sebnem Ozdogan, Gizem Sari, Sabanur Cavdar

PP64 Defining treatment response in childhood asthma: rationale and design of the Pharmacogenomics in the Childhood Asthma (PiCA) consortium

Niloufar Farzan, Susanne J. Vijverberg, Colin J. Palmer, Kelan G. Tantisira, Anke-Hilseon Maitland-van der Zee behalf of the PiCA consortium

PP65 Prevalence of asthma and allergic disease in patients with inflammatory disease compared to celiac disease

Fatma Yavuzyilmaz, Sebnem Ozdogan, Nafiye Urganci, Merve Usta

PP66 A severe case with cystic fibrosis (CF) asthma

Mehmet Hoxha, Maksim Basho

PP67 Severe asthma exacerbation complicated with pneumothorax in a child with uncontrolled asthma due to poor treatment compliance

Ioana Valentina Nenciu, Andreia Florina Nita, Adina Lazar, Alexandru Ulmeanu, Carmen Zapucioiu, Dumitru Oraseanu

PP68 Evaluation of the Pediatric Quality of Life inventory (PedsQL) asthma module among low income asthmatic children and adolescents in Sao Paolo, Brazil

Gustavo F. Wandalsen, Fernanda Monteiro, Dirceu Solé

PP69 Early initiation of specific immunotherapy in asthma patients leads to higher benefits

Blerta Lame, Eris Mesonjesi, Arjeta Sherri

PP70 Treatment resistant asthma and rhinosinusitis with recurrent pulmonary infections. Is it primary ciliary dyskinesia?

Alkerta Ibranji, Laert Gjati, Gjustina Loloci, Ardii Bardhi

PP71 The comparison of sensitisation to animal allergens in children- and adult- onset patients with asthma

Behnam Moghtaderi, Shirin Farjadian, Dorna Eghtedari

PP72 Characterisation of children less than five years with wheezing episodes in Cali, Colombia

Manuela Olaya, Laura Del Mar Vasquez, Luis Fernando Ramirez, Carlos Daniel Serrano

PP73 Evaluation of the patients with recurrent croup

Belgin Usta Guc, Suna Asilsoy, Fulya Ozer

PP74 Obesity in adolescence compromising the asthma control

Guergana Petrova, Sylvia Shopova, Vera Papochieva, Snezhina Lazova, Dimitrinka Miteva, Penka Perenovska

PP75 Sleep behavior in children with persistent allergic rhinitis

Gustavo F. Wandalsen, Jessica Loekmanwidjaja, Márcia Mallozi, Dirceu Solé

PP76 Randomised trial of the safety of MP29-02* compared with fluticasone propionate nasal spray in children aged ≥4 years to <12 years with allergic rhinitis

William Berger, Ulrich Wahn, Paul Ratner, Daniel Soteres

PP77 Safety and tolerability evaluation of bilastine 10 mg in children from 2 to 11 years of age with allergic rhinoconjunctivitis or urticaria

Zoltán Novák, Anahí Yáñez, Kiss Ildikó, Piotr Kuna, Miguel Tortajada, Román Valiente, the Bilastine Pediatric Safety Study Group

PP78 Sensitisation to Alternaria alternata: Is it a risk factor for severe rhinitis?

Susana Lopes, Filipa Almeida, Tânia Lopes, Cristina Madureira, José Oliveira, Fernanda Carvalho

PP79 Validation of the Patient Benefit Index (PBI) for the assessment of patient-related outcomes in allergic rhinitis in children

Julia Feuerhahn, Christine Blome, Meike Hadler, Efstrathios Karagiannis, Anna Langenbruch, Matthias Augustin

PP80 Efficacy of sublingual tablet of house dust mite allergen extracts in adolescents with house dust mite-associated allergic rhinitis

Michel Roux, Shinji Kakudo, Efstrathios Karagiannis, Robert K. Zeldin

PP81 Lung function improvement in a child treated with omalizumab for bronchial asthma

Anna Sokolova, Tiago Milheiro Silva

PP82 How to treat a child suffering from asthma, allergic rhinitis, allergy to peanuts and diabetes at the same time?

Snezana S. Zivanovic, Vesna Cvetkovic, Ivana Nikolic, Sonja J. Zivanovic

PP83 Nitric oxide in exhaled air in the relationship of the degree of sensitisation to aeroallergens

Snezana S. Zivanovic, Ljiljana Saranac, Ivana Nikolic, Sonja J. Zivanovic, Zorica Zivkovic

PP84 Clinical basis of diagnostic errors in pediatric asthma

Zoia Nesterenko

PP85

PP86 Childhood asthma control in Serbia and organised Asthma Educational Intervention (AEI)

Snezana Radic, Branislava Milenkovic, Spomenka Smiljanic, Milka Micic-Stanijevic, Olivera Calovic

PP87 Experience from a group of adolescents with severe allergic asthma treated with Omalizumab

Anne Marie Bro Hofbauer, Lone Agertoft

THEMATIC POSTER SESSION 1: Prevention and Treatment—Epidemiology (TP01–TP18)

TP01 A cost effective primary school asthma education program: pilot study from inner London schools

Lucy Everson, Jessica Kearney, Jonny Coppel, Simon Braithwaite, Rahul Chodhari

TP02 The prevalence of allergic diseases among 14–15 years old adolescents in two Danish birth cohorts 14 years apart

Elisabeth S. Christiansen, Henrik Fomsgaard Kjaer, Esben Eller, Charlotte G. Mørtz, Susanne Halken

TP03 Does pattern of sensitisation to phleum pratense change with age? Is it different in children with allergic rhinitis or asthma?

Cristina Román India, Ana Moreira Jorge, Loreto González Domínguez, Cristina Muñoz Archidona, Sergio Quevedo Teruel, Teresa Bracamonte Bermejo, Juana Jiménez Jiménez, Luis Echeverría Zudaire

TP04 Practicalities of prevention of peanut allergy: modelling a national response to LEAP

Cathal O’Connor, Jonathan Hourihane

TP05 Comparison of the influence of sunflower seed oil and skin care lotion on the skin barrier function of newborns: a randomised controlled trial

Varvara Kanti, Lena Lünnemann, Günther Malise, Laine Ludriksone, Andrea Stroux, Wolfgang Henrich, Michael Abu-Dakn, Ulrike Blume-Peytavi, Natalie Garcia Bartels

TP06 The effect of daily skin care on skin barrier properties in infants with dry skin and risk for atopic dermatitis

Varvara Kanti, Lena Lünnemann, Laine Ludriksone, Marianne Schario, Andrea Stroux, Ulrike Blume-Peytavi, Natalie Garcia Bartels

TP07 Change in sum total aeroallergen skin prick test wheal diameters at 6 months predicts which children will respond to subcutaneous immunotherapy by three years

Thorsten Stanley, Nicolien Brandenbarg

TP08 Are mobile apps regarding adrenaline auto-injectors accessed by adolescents for support and education in the community?

Alia Boardman, Gary McGreevy, Emily Rodger, Katherine Knight, Victoria Timms, Trisha Taylor, Gemma Scanlan, Roisin Fitzsimons

TP09

TP10 Prevention of early atopic dermatitis among low-atopy-risk infants by immunoactive prebiotics is not sustained after the first year of life

Grüber Christoph, Ulrich Wahn, Margriet van Stuivenberg, Fabio Mosca, Guido Moro, Gaetano Chirico, Christian P. Braegger, Joseph Riedler, Yalcin Yavuz, Günther Boehm

TP11

TP12

TP13 Treatment with Omalizumab in a 16-year-old Caucasian girl with refractory solar urticaria

Stefania Arasi, Giuseppe Crisafulli, Lucia Caminiti, Federica Porcaro, Giovanni Battista Pajno

TP14 Ultra-pure soft water ameliorates skin conditions of adult and child patients with atopic dermatitis

Akane Tanaka, Yaei Togawa, Kumiko Oida, Naotomo Kambe, Peter Arkwright, Yosuke Amagai, Naoki Shimojo, Yasunori Sato, Hiroyuki Mochizuki, Hyosun Jang, Saori Ishizaka, Hiroshi Matsuda

TP15 Potential adjuvant effect of immunomodulator to improve specific immunotherapy in asthmatic child

Wisnu Barlianto, Ery Olivianto, H. M. S. Chandra Kusuma

TP16 How can Component Resolved Diagnosis (CRD) influence in Specific Immunotherapy (SIT) prescription, in a Spanish children population

Ana Moreira Jorge, Cristina Román India, Loreto González Domínguez, Cristina Muñoz Archidona, Juana Jiménez Jiménez, Teresa Bracamonte Bermejo, Sergio Quevedo Teruel, Luis Echeverría Zudaire

TP17 Mitochondrial dysfunction in food allergy: effects of *L. rhamnosus* GG in a mice model of peanut allergy

Rosita Aitoro, Mariapia Mollica, Roberto Berni Canani, Giovanna Trinchese, Elena Alfano, Antonio Amoroso, Lorella Paparo, Francesco Amato, Claudio Pirozzi, Antonio Calignano, Rosaria Meli

TP18 Prediction of atopic diseases in childhood: elevated blood eosinophils in infancy in a high risk birth cohort

Siri Rossberg, Kerstin Gerhold, Kurt Zimmermann, Mohammad Zaino, Thomas Geske, Eckard Hamelmann, Susanne Lau

THEMATIC POSTER SESSION 2: Food allergy—Anaphylaxis (TP19–TP38)

TP19

TP20

TP21 Double-blind provocation tests in non-IgE mediated cow’s milk allergy and the occurrence of placebo reactions

Sarah Bogovic, Jochem van den Berg, Chantal Janssen

TP22 Gradual introduction of baked egg (BE) in egg allergic patients under 2 years old

Angela Claver

TP23 Randomised controlled trial of SOTI with raw hen’s egg in children with persistent egg allergy I: safety and efficacy of daily vs. weekly protocols of induction

Mª Flor Martin-Muñoz, C. Martorell, M. T. Belver, E. Alonso Lebrero, L. Zapatero, V. Fuentes, M. Piqué, A. Plaza, C. Muñoz, A. Martorell, Cristina Blasco, B. Villa, C. Gómez, S. Nevot, J. M. García, L. Echeverria

TP24 Randomised controlled trial of SOTI with raw hen’s egg in children with persistent egg allergy II: a randomised controlled trial to study a safer, more effective and easy to perform maintenance (daily vs. every two days) pattern of egg SOTI

Mª Flor Martin-Muñoz, C. Martorell, M. T. Belver, E. Alonso Lebrero, L. Zapatero, V. Fuentes, M. Piqué, A. Plaza, C. Muñoz, A. Martorell, Cristina Blasco, B. Villa, C. Gómez, S. Nevot, J. M. García, L. Echeverria

TP25 Determining the safety of baked egg home reintroduction for children with mild egg allergy

Brenda DeWitt, Judith Holloway, Donald Hodge

TP26 Demographics, investigations and patterns of sensitisation in children with oral allergy syndrome in a London Teaching Hospital

Sian Ludman, Merhdad Jafari-Mamaghani, Rosemary Ebling, Adam T. Fox, Gideon Lack, George Du Toit

TP27 Airborne peanut challenge in children: allergic reactions are rare

Sofia Lovén Björkman, Caroline Nilsson, Natalia Ballardini

TP28 The nutty question on Pediatric Wards: to be or “nut” to be?

Supriyo Basu, Jenny Hallet, Jyothi Srinivas

TP29

TP30

TP31 Allergy education in nursery schools

Hazel Stringer, Nicola Jay

TP32 Food allergy in the first year of life

Tânia Lopes, Cristina Madureira, Filipa Almeida, Susana Lopes, Paula Fonseca, Clara Vieira, Fernanda Carvalho

TP33 Prevalence and geographic distribution of oral allergy syndrome in Italian children: a multicenter study

Carla Mastrorilli, Carlo Caffarelli, Riccardo Asero, Salvatore Tripodi, Arianna Dondi, Gianpaolo Ricci, Carlotta Povesi Dascola, Elisabetta Calamelli, Francesca Cipriani, Andrea Di Rienzo Businco, Annamaria Bianchi, Paolo Candelotti, Tullio Frediani, Carmen Verga, Paolo Maria Matricardi

TP34 Are common standardised allergen extracts used in skin test enough in the diagnosis of nuts allergy?

Cristina Muñoz Archidona, Loreto González Domínguez, Ana Moreira Jorge, Sergio Quevedo Teruel, Teresa Bracamonte Bermejo, Miriam Castillo Fernández, Fernando Pineda de la Losa, Luis Ángel Echeverría Zudaire

TP35 Evaluation of IgE sensitisation in children with allergic proctocolitis and its relationship to atopic dermatitis

Despina Mermiri, Paraskevi Korovessi, Skevi Tiliakou, Evaggelia Tavoulari, Kalliopi-Maria Moraiti, Fotini Giannoula, Athina Papadopoulou

TP36 Food allergy in children: are we managing them appropriately in the Emergency Department?

Wan Jean Tee, Samir Deiratany, Raymond Seedhoo, Roisin McNamara, Ike Okafor

TP37 Importance of oil body associated allergenic proteins in nuts suspected allergy children

Loreto González Domínguez, Ana Moreira Jorge, Cristina Muñoz Archidona, Teresa Bracamonte Bermejo, Sergio Quevedo Teruel, Fernando Pineda de la Losa, Miriam Castillo Fernández, Luis Ángel Echeverría Zudaire

TP38 Practical application of basophil activation test in children with food allergy

Ekaterina Khaleva, Gennady Novic, Natalia Bychkova

THEMATIC POSTER SESSION 3: Asthma (TP39–TP57)

TP39 Effect of corticosteroid therapy upon serum magnesium level in chronic asthmatic children

Amany Abd Al-Aziz, Amany Fatouh, Ayat Motawie, Eman El Bostany, Amr Ibrahim

TP40 ADAM33 in Bulgarian children with asthma

Guergana Petrova, Dimitrinka Miteva, Snezhina Lazova, Penka Perenovska, Sylvia Andonova, Alexey Savov

TP41

TP42 The impact of vitamin D serum levels in asthma and allergic rhinitis

Maria Zoto, Marialena Kyriakakou, Paraskevi Xepapadaki, Nikolaos G. Papadopoulos

TP43 Life-threatening, first reported, paradoxical bronchospasm after nebulised Salbutamol in a 10 year old child

Paraskevi Korovessi, Mariza Vassilopoulou, Athina Balaska, Lambros Banos, Stavroula Kostaridou, Despina Mermiri

TP44

TP45 Asthma symptoms in children with treatment for allergic rhinoconjunctivitis

Jorien Wartna, Arthur M. Bohnen, Gijs Elshout, David H. J. Pols, Patrick J. E. Bindels

Erasmus MC, Rotterdam, The Netherlands

TP46 Atopy increased the risk of developing exercise-induced bronchoconstriction in young athletes

Sven F. Seys; Ellen Dilissen, Sarah Van der Eycken, An-Sofie Schelpe, Gudrun Marijsse, Thierry Troosters, Vincent Vanbelle, Sven Aertgeerts, Jan L. Ceuppens, Lieven J. Dupont, Koen Peers, Dominique M. Bullens

TP47 The effect of higher BMI on risk for asthma and treatment outcome in overweight and obese children

Ivana Banic, Sandra Bulat Lokas, Jelena Zivkovic, Boro Nogalo, Iva Mrkic Kobal, Davor Plavec, Mirjana Turkalj

TP48

TP49

TP50

TP51

TP52 The impact of a multidisciplinary project intended to change the culture of nebulisers towards pressurised metered dose inhalers

Georgeta Oliveira, Katharine Pike, Alda Melo, Tomás Amélia, José Carlos Cidrais Rodrigues, Cristina Serrano, José Manuel Lopes dos Santos, Carla Lopes

TP53

TP54

TP55

TP56 Increased asthma control in patients with severe persistent allergic asthma after 12 month of nightly temperature controlled laminar airflow (TLA)

Eckard Hamelmann, Uwe Schauer, Karl-Christian Bergmann

TP57

THEMATIC POSTER SESSION 4: Drug allergy—Dermatology (TP58–TP77)

TP58 Should we proceed directly to provocation challenges to diagnose drug allergy? Our experience says yes

Luis Moral, Teresa Toral, Nuria Marco, Beléns García Avilés, Mª Jesús Fuentes, Jesús Garde, Cristina Montahud, Javier Perona, Mª José Forniés

TP59 Anaphylaxis to 13-valent pneumococcal vaccine

Esozia Arroabarren, Marta Anda, Maria Luisa Sanz, Maria Teresa Lizaso, Candida Arregui

TP60 Intrapartum antibiotic exposure for treatment of group B streptococcus was not associated with the development of penicillin allergy in children

Sara May, Martha Hartz, Avni Joshi, Miguel A. Park

TP61 Evaluation of suspected drug hypersensitivity reactions in 169 children referred to the General Hospital

Sonja Posega Devetak, Tina Vesel, Anja Koren Jeverica, Tadej Avčin

TP62 Drug provocation testing: experience of a tertiary hospital

Leonor Castro, Carolina Gouveia, Ana Carvalho Marques, Antonio Jorge Cabral

TP63 Perioperative anaphylaxis: a growing concern in pediatric population

Luis Amaral, Fabrícia Carolino, Eunice Castro, Madalena Passos, Josefina R. Cernadas

TP64 Raising awareness of hypersensitivity to non-steroidal anti-inflammatory drugs in the pediatric age

Fabrícia Carolino, Luís Amaral, Eunice Dias de Castro, Josefina R. Cernadas

TP65 Perioperative anaphylaxis in young children: how to confirm the suspicion

Josefina R. Cernadas, Fabrícia Carolino, Luís Amaral, Fernando Pineda, Armanda Gomes

TP66 A case study of a child suspected to be penicillin allergic-digging deeper

Katherine Knight, Roisin Fitzsimons, Helen Brough

TP67 Prevalence, characteristics and risk factors of hypersensitivity reactions to antibiotics in patients with cystic fibrosis

Jobst Röhmel, Carsten Schwarz, Anne Mehl, Philippe Stock, Doris Staab

TP68 Antibiotic drug hypersensitivity in cystic fibrosis: A pilot study using cellular allergy tests for diagnostics

Jobst Röhmel, Carsten Schwarz, Christine Seib, Doris Staab, Philippe Stock

TP69 Oral antibiotics challenges in children

Anita Critchlow, Alyson Barber, Nicola Jay

TP70 Hypersensitivity reaction to vancomycin: a new successful desensitization protocol

Belen Delavalle, Teresa Garriga, Blanca Vilá, Cristina Blasco

TP71

TP72 Clinical phenotypes according to FLG gene loss of function mutations in children with atopic dermatitis

Francesca Cipriani, Annalisa Astolfi, Costanza Di Chiara, Elisabetta Calamelli, Iria Neri, Annalisa Patrizi, Gianpaolo Ricci

TP73

TP74 Urticaria in children: clinical and epidemiological features

Katerina Neskorodova, Asya Kudryavtseva

TP75

TP76 Acute urticaria at the Pediatrics Emergency Department: is it allergy?

Esozia Arroabarren, Jorge Alvarez, Marta Anda, Miriam Palacios, Marta Martinez-Merino, Ibone Vaquero

TP77

## WORKSHOP 4: Challenging clinical scenarios (CS01–CS06)

### CS01 Bullous lesions in two children: solitary mastocytoma

#### S. Tolga Yavuz^1^, Ozan Koc^2^, Ali Gungor^2^, Faysal Gok^2^

##### ^1^Department of Pediatric Allergy, GATA School of Medicine, Ankara, Turkey; ^2^Department of Pediatrics, GATA School of Medicine, Ankara, Turkey

###### **Correspondence:** S. Tolga Yavuz

*Clinical and Translational Allergy* 2016, **6(Suppl 1)**:CS01

**Introduction:** Bullous lesions are common skin lesions particularly in childhood. Drug reactions, burns, insect bites, mosquito bites, skin diseases, autoimmune bullous dermatoses and bacterial infections are the most common etiologies. Herein, we report two children who have solitary mastocytoma presented with variable bullous lesions.

**Case 1:** A 3-year old boy admitted to our outpatient department suffering from red, periodically vesicular and bullous lesions on his back of neck since birth. His medical history revealed that he had four attacks characterized by flushing, perioral paleness, and hypotension. The lesion became swollen and itchy when it was rubbed vigorously (positive Darier’s sign). Physical examination revealed a bullous lesion (2 × 3 cm) in his dorsal neck region. Laboratory investigations including CBC, liver and kidney function tests and serum electrolytes were within normal limits. Histopathologic examination of a punch biopsy specimen revealed solitary mastocytoma and the patient is under regular antihistamine treatment.

**Case 2:** A 3-month old boy admitted with complaints of oval, erythematous and periodically changing bullous lesions in his proximal of right ankle since birth. His medical history revealed that the lesion became bullous and itchy when his mother rubbed it vigorously. Physical examination revealed a bullous lesion (1 × 1 cm) in his left foot. Laboratory investigations for possible etiologic factors were within normal limits. Histopathologic examination of a punch biopsy specimen revealed solitary mastocytoma. The patient is uneventfully under regular follow up.

**Conclusion:** Solitary mastocytomas should be considered in the differential diagnosis of periodically varying bullous reactions in children.

**Consent to publish**

Written informed consent for publication of this clinical details and/or clinical images was obtained from the patient/parent/guardian/relative of the patient. A copy of the consent form is available for review by the Editor of this journal.

### CS02 Multi-System Allergy (MSA) of cystic fibrosis: our institutional experience

#### Jessica Hawley^1^, Christopher O’Brien^2^, Matthew Thomas^2^, Malcolm Brodlie^2^, Louise Michaelis^2^

##### ^1^Newcastle University, Newcastle, UK; ^2^Royal Victoria Infirmary, Great North Children’s Hospital, Newcastle, UK

###### **Correspondence:** Jessica Hawley

*Clinical and Translational Allergy* 2016, **6(Suppl 1)**:CS02

The published version of this abstract can be found at [1].

**Reference**

1. Clin Exp Allergy. 2015;45(12):1876–913. http://onlinelibrary.wiley.com/doi/10.1111/cea.12656/abstract.

### CS03 Cold urticaria in pediatric age: an invisible cause for severe reactions

#### Inês Mota, Ângela Gaspar, Susana Piedade, Graça Sampaio, José Geraldo Dias, Miguel Paiva, Mário Morais-Almeida

##### Immunoallergy Department, CUF Descobertas Hospital, Lisbon, Portugal

###### **Correspondence:** Inês Mota

*Clinical and Translational Allergy* 2016, **6(Suppl 1)**:CS03

**Background:** Cold urticaria (CU) is a subtype of inducible urticaria, which can be responsible for severe reactions namely during aquatic activities and winter season. The prevalence and clinical course in pediatric age is barely known. The authors describe the clinical features and the evolution of CU in pediatric patients.

**Methods:** Retrospective characterization of 15 pediatric patients with CU followed at our Immunoallergy Department, including clinical presentation, ice cube challenge test (ICCT) result, laboratory testing, allergen sensitization and outcome.

**Results:** The mean age of onset was 8.9 ± 4.2 years old, 73 % were female. Most patients had other allergic diseases (87 %), 40 % were atopic, two had also cholinergic urticaria and one case had family history of CU. Five patients (33 %) had systemic reactions with hemodynamic collapse after cold exposure. Symptoms occurred few minutes after skin exposure to cold (median of 7 min; ranging from immediate reactions to 20 min later); 73 % had several episodes (>5). Found one case secondary to cryoglobulinemia and the remaining cases were considered idiopathic. Aquatic activities (swimming, sea bathing) and cold air exposure were the main triggers. Some children developed reactions when touching cold objects (3), with cold beverages (1) and intraoperative (1). Positive ICCT with ≤3 min of stimulation in 5 patients (3 of 5 who had type III reactions), 5 min in 1, 10 min in 5 and 20 min in 3 of them. Patients were successfully controlled with prophylactic antihistamines and avoidance measures; adrenaline was prescribed when indicated. Five patients (33 %) overcame the symptoms in less than 5 years (mean: 3.6 years). Those who remain susceptible have so far a follow-up period of 1–13 years.

**Conclusions:** In pediatric age, CU seems to be a persistent disorder. ICCT is a convenient tool to confirm the diagnosis, to assess the risk for severe reactions and follow-up. Severe reactions can be prevented with prophylactic treatment and cold exposure avoidance.

### CS04 Angioedema with C1 inhibitor deficiency in a girl: a challenge diagnosis

#### Cristina Madureira, Tânia Lopes, Susana Lopes, Filipa Almeida, Alexandra Sequeira, Fernanda Carvalho, José Oliveira

##### CHMA, Famalicão, Portugal

###### **Correspondence:** Cristina Madureira

*Clinical and Translational Allergy* 2016, **6(Suppl 1)**:CS04

**Introduction:** Angioedema is defined as localised and self-limiting nonpitting recurrent edema of the skin and mucous membranes caused by the release of several vasoactive mediators.

Seven forms of angioedema were identified based on specific characteristics and are classified as non-hereditary and hereditary forms.

Only Hereditary Angioedema with C1 inhibitor (C1-INH) deficiency had approved treatment.

The authors present a case of angioedema with C1 deficiency without family history of angioedema.

**Fig. 1:**
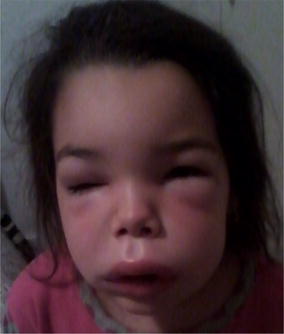
Angioedema of the face

**Clinical case:** Five years old girl attended in emergency unit with angioedema of the face (Fig. 1) after left periorbital trauma. There was improvement of the clinical picture after administration of epinephrine, corticosteroids and antihistamine. She had a history of two previous similar episodes both triggered by trauma. Edema disappears completely after 5 days. Family history of angioedema is unknown.

The study carried out in outpatient pediatric allergy revealed: C4 very low (<5 mg/dl) and low esterase C1-INH (30 mg/L) with decreased function (10 %). She waits for genetic study of C1-INH gene (SERPING1).

Currently the child is medicated with an antihistamine without new episodes of angioedema.

**Conclusion:** Angioedema without urticaria in children is an infrequent condition and requires an exhaustive diagnostic investigation. Dosing C4 and C1-INH esterase becomes fundamental when no etiologic factor is identified. Deficit of C1-INH esterase with a decrease in C4 points to an acquired or a hereditary form of angioedema. Hereditary angioedema with C1-INH deficiency is a rare disease with an estimated prevalence of 1/10,000–1/100,000 inhabitants and is due to a mutation in one of the two alleles of the C1-INH gene (SERPING1). In the absence of family history, the genetic study becomes important to define possible prophylaxis.

**Consent to publish**

Written informed consent for publication of this clinical details and/or clinical images was obtained from the patient/parent/guardian/relative of the patient. A copy of the consent form is available for review by the Editor of this journal.

### CS05 A child with unusual multiple organ allergy disease: what is the primer?

#### Fabienne Gay-Crosier

##### Clinical Allergy and Clinical Immunology, Internal Medicine, Federal Specialist, Geneva, Switzerland

###### **Correspondence:** Fabienne Gay-Crosier

*Clinical and Translational Allergy* 2016, **6(Suppl 1)**:CS05

**Introduction**: An immediate contact urticaria to food rich in palm date oil was developed by Julia at 2 years old. At 6 years old, she developed rhinoconjunctivitis and a severe onset of asthma on the 15th April 2014.

**Method and results:** SPT for panallergens profilin and polcalcin were positive which led to conduct to multiple false positive result for usual pollens’ SPT extracts [1].

Pollen calendar issued from the Swiss centre for meteorology gave this year for betulaceae like birch, a highest pollen count of 798 grains/m^3^ on the 2.04.2014. No pollen count was really pertinent on the 15 April 2014, date of the asthma crisis. Three days before, the highest pollen count of plane tree (400 grains/m^3^) and oak (360 grains/m^3^) were recorded. The ash highest pollen count was 62 grains/m^3^ on the 1st of April; the cupressaceae highest pollen count was 182 grains/m^3^ on the 17th of March.

**Conclusion:** According to SPT and biology, profilin is probably the primer to explain contact food urticaria to palm oil pastries, Phod2 [2] being the major allergen contained in palm oil in accordance to SPT profilin.

According to molecular biology [1,3], the pathogenesis of accumulated panallergens [4] due to multiple pollen expositions before the 15th April might explain the severe pollinosis [5].

To my knowledge, this is the first case report of profilin contact dermatitis in childhood. Recently, panallergens have been related to severe clinical pollinosis [6] and the role of enzyme activity of particular allergenic pollens has been described [7,8]. These mechanisms are probably involved predominantly in the delayed phase reaction of allergic asthma [9,10].

This observation should lead clinicians to improve their diagnosis and understanding of different panallergen and/or enzyme activity implications in allergy disease’ clinical onset and severity [11], particularly in multiple pollens exposed area and multi-ethnic areas [11,12].

**Consent to publish**

Written informed consent for publication of this clinical details and/or clinical images was obtained from the patient/parent/guardian/relative of the patient. A copy of the consent form is available for review by the Editor of this journal.

**References**

1. Barber D, de la Torre F, Lombardero M, Antépara I, Colas C, Dávila I, Tabar AI, Vidal C, Villalba M, Salcedo G, Rodríguez R. Component-resolved diagnosis of pollen allergy based on skin testing with profilin, polcalcin and lipid transfer protein pan-allergens. Clin Exp Allergy. 2009;39(11):1764–73.
2. Asturias JA, Ibarrola I, Fernández J, Arilla MC, González-Rioja R, Martínez A. Pho d 2, a major allergen from date palm pollen is a profilin: cloning, sequencing and immunoglobulin E cross-reactivity with other profilins. Clin Exp Allergy. 2005;35:374–81.
3. Barber D, de la Torre F, Feo F, Florido F, Guardia P, et al. Understanding patient sensitization profiles in complex pollens areas: a molecular epidemiological study. Allergy. 2008;63:1550–1558.
4. Hauser M, Roulias A, Ferreira F, Egger M: Panallergens and their impact on the allergic patient. Allergy Asthma Clin Immunol 2010;6:1–14.
5. Ribeiro H, Abreu I, et al. Pollen allergenic potential nature of some trees species: a multidisciplinary approach using aerobiological, immunochemical and hospital admissions data. Environ Res. 2009;109(3):328–33.
6. Patelis A, et al. Population-based study of multiplexed IgE sensitization in relation to asthma, exhaled nitric oxide, and bronchial responsiveness. J Allergy Clin Immunol 2011;130(2):397–402.
7. Barderas R, Villalba M, Pascual CY, Batanero E, Rodríguez R. Profilin (Che a 2) and polcalcin (Che a 3) are relevant allergens of Chenopodium album pollen: isolation, amino acid sequences and immunologic properties. J Allergy Clin Immunol. 2004;113:1192–8.
8. Mas S, Garrido-Arandia M, Batanero E, Purohit A, Pauli G. Characterization of profilin and polcalcin panallergens from ash pollen. J Investig Allergol Clin Immunol. 2014;24(4):257–66.
9. Smole U, Balazs N, Hoffmann-Sommergruber K, Radauer C, Hafner C, Wallner M, Ferreira F, Grossinger R, De Jong EC, Wagner S, Breiteneder H. Differential T cell responses and allergen uptake after exposure of dendritic cells to the birch pollen allergens Bet v 1.0101, Bet v 1.0401 and Bet v 1.1001. Immunobiology 2010;215(11):903–9.
10. Schulten V, Sette A, Peters B, et al. Previously undescribed grass pollen antigens are the major inducers of T helper 2 cytokine-producing T cells in allergic individuals. Proc Natl Acad Sci USA 2013;110(issue 9):3459–64.
11. Barber D, Díaz-Perales A, Villalba M, Chivato T. Challenges for allergy diagnosis in regions with complex pollen exposures in press. In: Current Allergy and Asthma Reports, vol. 15, 2015. p. 496.
12. Gay-Crosier F, Barber D, Bienvenu J. Allergy diagnosis in Geneva area: a complex multi-ethnic community with high pan-allergen prevalence. Clin Transl Allergy. 2014;4(Suppl 2):P49.

### CS06 A case of uncontrolled asthma in a 6-year-old patient

#### Ioana-Valentina Nenciu, Andreia Florina Nita, Alexandru Ulmeanu, Dumitru Oraseanu, Carmen Zapucioiu

##### “Grigore Alexandrescu” Emergency Hospital for Children, Bucharest, Romania

###### **Correspondence:** Ioana-Valentina Nenciu

*Clinical and Translational Allergy* 2016, **6(Suppl 1)**:CS06

**Introduction:** Poorly controlled asthma involves higher costs related to emergency department visits, hospitalizations and scholar absenteeism. A survey of pediatric patients visiting their primary care providers revealed a 46 % prevalence of uncontrolled asthma (Liu AH et al. 2010).

**Case report:** We report a case of a 6-year-old male patient with uncontrolled asthma, receiving treatment with fluticasone and montelukast, who referred to our hospital for chronic cough and repeated asthma exacerbations. The patient was diagnosed with asthma at 4 years and with gastro-esophageal reflux at 5 years. He was also diagnosed with hypogammaglobulinemia and immunoglobulin A deficiency. The child’s mother is smoker. The parents were advised about the therapy and the measures they should take in order to improve their child’s outcome. Giving the repeated asthma exacerbations despite the indication of correct treatment, differential diagnosis and comorbidities were taken into account. The spirometry revealed obstructive syndrome reversible after 200 μg salbutamol administration. Thoracic CT scan showed bronchiectasis located in the middle lobe and lingula. Bronchoscopy revealed severe inflammation of traheobronhic mucosa and bronchial dyskinesia. FENO was 1 ppb and the sweat test was normal. Chest X-ray revealed increased interstitial markings and the X-ray for the sinuses was normal. Allergy testing and diamine oxidase activity were normal. All the tests revealed lack of control both for the asthma and for the gastro-esophageal reflux, despite the treatment, leading to important complications such as bronchiectasis. Repeated history revealed that the child did not receive the treatment correctly, daily, ant the mother continued to smoke close to the child.

**Acknowledgements:** This paper was co-financed from the European Social Fund, through the Sectorial Operational Programme Human Resources Development 2007–2013, contract POSDRU/187/1.5/S/155463 “Supporting excellence in scientific interdisciplinary doctoral research in the economic, medical and social fields”, coordinator The Bucharest University of Economic Studies.

**Consent to publish**

Written informed consent for publication of this clinical details and/or clinical images was obtained from the patient/parent/guardian/relative of the patient. A copy of the consent form is available for review by the Editor of this journal.

## ORAL ABSTRACT SESSION 1: Food allergy (OP01–OP06)

### OP01 Food protein-induced enterocolitis syndrome: oral food challenge outcomes for tolerance evaluation in a Pediatric Hospital

#### Adrianna Machinena, Olga Domínguez Sánchez, Montserrat Alvaro Lozano, Rosa Jimenez Feijoo, Jaime Lozano Blasco, Mònica Piquer Gibert, M^a^ Teresa Giner Muñoz, Marcia Dias da Costa, Ana Maria Plaza Martín

##### Pediatric Allergy Department, Hospital Sant Joan de Déu, Barcelona, Spain

###### **Correspondence:** Adrianna Machinena

*Clinical and Translational Allergy* 2016, **6(Suppl 1)**:OP01

**Aim:** To report clinical features, food allergens, reaction threshold doses and age of tolerance within a pediatric population with food protein-induced enterocolitis syndrome (FPIES).

**Methods:** Retrospective study of children with suspected FPIES who have undergone an oral food challenge (OFC) for tolerance evaluation from June 2008 to December 2013. Variables registered: age at OFC, gender, family and personal atopic history, symptoms, food involved, skin prick test (SPT), total and specific IgE and OFC outcomes.

**Results:** 82 patients with FPIES were included, 52.4 % boys, 42.7 % with atopic family history and 18.3 % had atopic dermatitis. Vomiting, lethargy, pallor and diarrhea were reported in 95.1, 67.1, 48.8 and 41.5 %, respectively, with an average time of reaction of 2 h post-ingestion (1.5–6). Food allergens implicated: fish (53.7 %), cow’s milk (25.6 %), egg (11 %), rice and corn (9.8 %). Mean age at OFC: 38.7 months (6–106 months) and 58.5 % (n 48) resulted tolerant.

The mean age at which tolerance was achieved differed according to the trigger food with statistical significance: 51.2 months for fish, 29.9 months for cow’s milk, 45.1 months for egg and 33.4 months for cereals. Of the 20 children (n 44) who tolerated one fish at OFC, 16 also tested negative with other fish families.

The reaction threshold doses were: 1.6 g for cow’s milk, about 2 g for egg and 0.5 g of protein for rice. Regarding fish, the reaction threshold dose was different depending on the fish family involved: half of the positive OFC had tested hake (n 13) and 69 % (n 9) reacted with 3.6 g of protein.

**Conclusion:** This study shows high prevalence of FPIES for fish in our environment. Fish tolerance resolves later than other foods and the outcome can usually be applied to other fish families. We detect low reaction threshold doses especially for cereals and fish.

### OP02 Characteristics of infants with food protein-induced enterocolitis syndrome and allergic proctocolitis

#### Ebru Arik Yilmaz, Özlem Cavkaytar, Betul Buyuktiryaki, Ozge Soyer, Cansin Sackesen

##### Division of Pediatric Allergy, School of Medicine, Hacettepe University, Ankara, Turkey

###### **Correspondence:** Ebru Arik Yilmaz

*Clinical and Translational Allergy* 2016, **6(Suppl 1)**:OP02

**Background:** Food protein-induced enterocolitis syndrome (FPIES) and allergic proctocolitis (AP) are rare non-IgE-mediated food allergies in early childhood. We aimed to determine the clinical and laboratory features of FPIES and AP in infants.

**Method:** FPIES was diagnosed in the presence of gastrointestinal symptoms within 24 h after the ingestion of incriminated foods, without any other cause for the symptoms; or a positive open food challenge result with causative food or removal of causative food from the diet resulting in the resolution of symptoms in infants. AP was diagnosed in the presence of bloody stool after ingesting incriminated food and disappearance of blood with elimination of incriminated food or with positive challenge test.

**Results:** We analyzed 52 patients (23 FPIES and 29 AP) between 2010 and 2014. The age at admission was significantly lower in patients with AP than FPIES [4.2 (3.1–7.2) vs. 8.9 (4.4–12.7) month, p = 0.008] and age of initial symptoms was slightly lower in AP than FPIES [2 (1–3 vs. 4.5 (1–6) month, p > 0.05]. Cow’s milk was determined as the most frequent trigger with 90.4 %. The other incriminated foods are hen’s egg (n = 7), rice (n = 2), fish (n = 2), potato (n = 1), lentil (n = 1), wheat (n = 1), soy (n = 1) and banana (n = 1). Three patients had positive skin prick test with the suspected food, 7 (13.5 %) had concomitant IgE-mediated food allergy (egg, milk walnut), and 15 (28.8 %) had atopic dermatitis. Oral food challenge test was performed in 40 patients and 13 of those (32.5 %) resulted positive. The age of recovery of diseases was similar in FPIES and AP [12 (10.5–15.3) and 13 (12–20.3) months, respectively].

**Conclusion:** Our results denoted that cow’s milk is the most common trigger of FPIES and AP. Although the age of onset for initial symptoms seems to be earlier in AP compared with FPIES, resolution age was similar.

### OP03 The clinical and immunological outcomes after consumption of baked egg by 1–5 year old egg allergic children: results of a randomised controlled trial

#### Merryn Netting^1^, Adaweyah El-Merhibi^1^, Michael Gold^2^, Patrick Quinn^2^, Irmeli Penttila^3^, Maria Makrides^4^

##### ^1^Women’s and Children’s Health Research Institute, Adelaide, Australia; ^2^School of Pediatrics and Reproductive Health, University of Adelaide, Adelaide, Australia; ^3^School of Medicine, The University of Adelaide, Adelaide, Australia; ^4^South Australian Health Medical Research Institute, Adelaide, Australia

###### **Correspondence:** Merryn Netting

*Clinical and Translational Allergy* 2016, **6(Suppl 1)**:OP03

**Background:** This RCT aimed to compare clinical and immunological outcomes after 6 months consumption of baked egg (BE) with an egg free diet in 1–5 year old BE tolerant, but raw egg allergic children.

**Methods:** Children were recruited at the Women’s and Children’s Hospital, Adelaide, Australia Allergy Clinic and randomised into two groups according to a protocol approved by the Institutional Human Research Ethics Committee, (REC2400/9/14; ACTRN 12612000173897). The intervention group consumed 10 g BE per serve of the provided muffins, biscuits or cake, two to three times per week for 6 months. The control group consumed identical egg free products. Both groups maintained egg free diets during the trial. The final assessment was a medically supervised raw egg oral food challenge (OFC) at 7 months. Immune markers, including skin prick testing (SPT), egg specific IgE and IgG4, Th1/Th2 cytokines and T cell phenotype were assessed at baseline and 7 months.

**Results:** 43 children were randomised into the study (intervention group n = 21; control group n = 22). The final analysis included 35 children (intervention group n = 17; control group n = 18) who had raw egg OFCs. Ten children (4/17 intervention group and 6/18 control group) tolerated raw egg at the end of the intervention. Tolerance was independent of age and amount of BE consumed. Both groups demonstrated decreased SPT weal sizes and whole egg, egg white, ovalbumin specific serum IgE titre and increased whole egg IgG4. No difference between the groups was observed in the percentage of naive (CD4^+^ CD45RA^+^), central (CCR7^−^CD45RA^−^) or effector (CCR7^+^CD45RA^−^) memory T-cells or cytokine excretion after culture of cells with egg allergens.

**Conclusion:** The results suggest that BE tolerant 1–5 year old egg allergic children are evolving tolerance to raw egg which is not hastened by short term, regular inclusion of BE. Further trials of larger sample size, including children of different age groups are required.

### OP04 Oral immunotherapy for treatment of egg allergy using low allergenic, hydrolysed egg

#### Stavroula Giavi^1^, Antonella Muraro^2^, Roger Lauener^3,4^, Annick Mercenier^5^, Eugen Bersuch^3,4^, Isabella M. Montagner^6^, Maria Passioti^1^, Nicolò Celegato^2^, Selina Summermatter^3,4^, Sophie Nutten^5^, Tristan Bourdeau^5^, Yvonne M. Vissers^5^, Nikolaos G. Papadopoulos^1,7^

##### ^1^Allergy Department, 2nd Pediatric Clinic, University of Athens, Athens, Greece; ^2^ Referral Centre for Food Allergy Diagnosis and Treatment, Veneto Region, Department of Women and Child Health, Padua University Hospital, Padua, Italy; ^3^Children’s Hospital of Eastern Switzerland, St. Gallen, Switzerland; ^4^CK-CARE, Davos, Switzerland; ^5^Nestlé Research Center, Lausanne, Switzerland; ^6^ Department of Surgery, Oncology and Gastroenterology, Veneto Institute of Oncology IOV-IRCCS, Padua, Italy; ^7^Centre for Pediatrics and Child Health, Institute of Human Development, University of Manchester, Manchester, UK

###### **Correspondence:** Yvonne M. Vissers

*Clinical and Translational Allergy* 2016, **6(Suppl 1)**:OP04

**Background:** There is considerable interest in oral immunotherapy (OIT) to treat food allergy. Because the major drawback of OIT is severe side effects, we designed a low allergenic hydrolysed egg (HE) product and tested its efficacy for desensitisation.

**Method:** In a double-blind placebo control multicentre pilot study (Athens, Davos, Padua), 29 patients (aged 1–5.5 years), diagnosed with IgE-mediated egg allergy were included. A tolerance assessment was performed at day 1 and afterwards 9 ± 1 g HE (n = 15) or placebo (n = 14) was administered daily for 6 months. Primary outcome was the result for OFC to boiled egg performed at the end of the study. Basophil activation and specific IgE and IgG4 were assessed at the start and end of the study.

**Results:** All egg allergic patients randomized to HE (n = 15) tolerated the full dose at day 1. No significant difference was observed on the primary outcome (36 % and 21 % had a negative OFC in the treatment and placebo group, respectively). While no significant difference was observed regarding egg specific IgE levels, IgG4 to egg white, egg yolk and ovalbumin increased significantly more over time in the treatment than in the placebo group (p = 0.07, p = 0.01 and p = 0.04, respectively). A higher increase over time in the treatment group compared to the placebo group was also observed for specific IgG4/specific IgE. In the basophil activation test, a significant decrease in both CD203c+ (p = 0.04) and CD63+ (p = 0.07) was observed after stimulation with 0.01 µg/ml ovalbumin in the treatment group, as compared to an increase over time in the placebo group.

**Conclusion:** HE given for 6 months did not change significantly the proportion of patients becoming tolerant to egg. However, HE induced a modulation of the immune response towards better tolerance. A larger study considering a longer treatment period and/or a higher dose could improve the clinical outcome.

**Conflicts of interest** AM, SN, TB and YMV are employees of Nestec Ltd. The study was sponsored by Nestec Ltd.

### OP05 Chemical modification of a peanut extract results in an increased safety profile while maintaining efficacy

#### Hanneke van der Kleij^1^, Hans Warmenhoven^1^, Ronald van Ree^2^, Raymond Pieters^3^, Dirk Jan Opstelten^1^, Hans van Schijndel^1^, Joost Smit^1^

##### ^1^HAL Allergy B.V., Haarlem, The Netherlands; ^2^Experimental Immunology, Academic Medical Centre, Amsterdam, The Netherlands; ^3^Institute for Risk Assessment Sciences, Immunotoxicology, Utrecht University, Utrecht, The Netherlands

###### **Correspondence:** Hanneke van der Kleij

*Clinical and Translational Allergy* 2016, **6(Suppl 1)**:OP05

**Background:** Peanuts are responsible for the induction of the majority of food related anaphylactic reactions. A curative treatment is not yet available for peanut-allergic patients. A chemically modified peanut extract is being investigated for its potential use in immunotherapy.

**Methods:** Peanut extract (PE) was reduced and subsequent alkylated resulting in modified PE (MPE) followed by adsorption to aluminium hydroxide (AlOH_3_). IgE-binding assays using a set of sera from peanut-allergic patients and mediator release assays (MRA) using human basophils were performed. The immunogenicity of PE and MPE was evaluated by the induction of PE-specific IgG after i.p. injections of PE and MPE in mice. In addition, mice were sensitized intra-gastrically for PE and either (1) s.c. challenged with (M) PE ± Al(OH)_3_ to assess safety, or (2) de-sensitized by s.c. injections of PE or MPE ± Al(OH)_3_ for 3–6 weeks, followed by oral and i.p. challenges to assess efficacy. Body temperature was measured after challenge as an objective parameter for an anaphylactic shock response. During the course of immunotherapy, blood samples were taken for analysis of antibody responses.

**Results:** The IgE-binding of MPE was decreased (mean remaining potency: 7.2 ± 5 %) when tested in all patient sera. The potency of MPE in MRA was also reduced for all patients (mean reduction: 10- to 100-fold, range 3- to >10,000-fold). PE and MPE were equally potent in inducing PE-specific IgG antibodies in mice. Mice sensitized for PE experienced severe anaphylactic symptoms upon s.c. challenge with PE. These effects were aborted after modification of PE or after complete binding of PE to Al(OH)_3_. Immunotherapy with both PE and MPE (±alum) resulted in a dose-dependent reduction of the anaphylactic response upon systemic challenge. In addition, both PE and MPE (±alum) were able to induce strong increases in the levels of PE-specific IgG1 and IgG2a compared to non-desensitized mice.

**Conclusions:** Various in vitro and in vivo model systems have shown that MPE adsorbed to Al(OH)_3_ has a significantly improved safety profile compared to PE while retaining its efficacy profile.

### OP06 Administration of the yellow fever vaccine in egg allergic children

#### Roisin Fitzsimons, Victoria Timms, George Du Toit

##### Guys and St Thomas’ NHS Trust, London, UK

###### **Correspondence:** Roisin Fitzsimons

*Clinical and Translational Allergy* 2016, **6(Suppl 1)**:OP06

**Objectives:** Yellow Fever Vaccine (YFV) was developed in the 1930s for the prevention of YF. In the UK it is administered to those travelling to countries where there is a risk of contracting the disease. Adverse reactions are possible following administration of YFV: mild localised erythema, malaise or more severe, but rare, neurotropic or viscerotropic disease. YFV is propagated on hen’s egg and as such poses a risk of allergic reaction to those children with an egg allergy.

Children in the UK with an egg allergy were unable to receive the YFV until GSTT Children’s Allergy Service was awarded a licence to be an YFV centre in 2013. There are few studies looking at the administration of YFV in patients with an egg allergy, suggesting this can be administered in divided doses and protective immunity achieved following intradermal administration 1/5 of the dose.

**Methods:** A prospective evaluation of patients receiving the YFV between 2013 and 2015 was performed. The administration protocol includes: counselling regarding geographical area to which travel is planned, risks and benefits of receiving the YFV, SPT, and YFV administered as a subcutaneous injection in divided doses—1/10 dose and 30 min later 9/10 dose. The patient is observed for one hour post administration.

**Results:** Fifteen patients attended the service for administration of the YFV with an age range of 10 months–20 years. Two (13 %) children had positive SPT and did not receive the vaccine. Two (13 %) children had previously had anaphylaxis to egg, both tolerated the YFV. Three (20 %) children tolerated baked egg in their diet—they all tolerated administration of the vaccine, which was administered in divided doses.

**Conclusions:** YFV can be safely administered to children who are allergic to egg. We should explore the possibility of administering 1/5 of the dose intradermally to those children who are positive on SPT.

## ORAL ABSTRACT SESSION 2: Asthma (OP07–OP12)

### OP07 Previous exacerbation is the most important risk factor for future exacerbations in school-age children with asthma

#### S. Tolga Yavuz^1^, Guven Kaya^2^, Mustafa Gulec^3^, Mehmet Saldir^2^, Osman Sener^3^, Faysal Gok^2^

##### ^1^Department of Pediatric Allergy, Gata School of Medicine, Ankara, Turkey; ^2^Department of Pediatrics, Gata School of Medicine, Ankara, Turkey; ^3^Department of Adult Immunology and Allergic Diseases, Gata School of Medicine, Ankara, Turkey

###### **Correspondence:** S. Tolga Yavuz

*Clinical and Translational Allergy* 2016, **6(Suppl 1)**:OP07

**Background:** Acute asthma exacerbation is one of the most frequent emergencies in childhood. We aimed to investigate the risk factors associated with exacerbations in school-age children with asthma.

**Method:** Children who attended to a tertiary outpatient pediatric allergy and asthma department and diagnosed with asthma were enrolled in the study. A questionnaire including demographic features and parameters to determine socioeconomic status along with previous disease history were applied in the admission visit. Recent GINA guidelines were used to determine the asthma control status of patients and for the diagnosis of asthma exacerbation. Laboratory investigations including complete blood counts with differential, total IgE levels, skin prick tests and pulmonary function tests were also performed.

**Results:** A total of 431 children (288 male (66.8 %); with a median age [interquartile range] of 8.1 [6.3–11.2] years were included. Asthma was controlled in 154 children (35.7 %), whereas partially controlled and uncontrolled in 53 (12.3 %) and 143 (33.2 %) patients, respectively. 81 patients (18.8 %) presented with an asthma exacerbation. Multivariate logistic regression analysis revealed that history of asthma exacerbation in the last year (Odds Ratio [Confidence Interval]) (16.51 [6.97–39.11]; p < 0.001), lack of previous asthma diagnosis (3.02 [1.53–5.96]; p = 0.001), history of ER admission in the last year (2.34 [1.18–4.66]; p = 0.015) and lack of regular controller therapy (2.80 [1.03–7.61]; p = 0.044) were related with asthma exacerbation whereas presence of rhinitis emerged as a “protective” factor (0.43 [0.24–0.79]; p = 0.006) in school-age children with asthma.

**Conclusion:** Awareness of risk factors related with asthma exacerbation may alert physicians who deal with school-age children with asthma and may help prompt and rational interventions in order to prevent asthma exacerbations.

### OP08 Comparative study of degree of severity and laboratory changes between asthmatic children using different acupuncture modalities

#### Nagwa Hassan^1^, Hala Shaaban^2^, Hazem El-Hariri^1^, Ahmed Kamel Inas E. Mahfouz^1^

##### ^1^National Research Centre, Cairo, Egypt; ^2^Faculty of Medicine, Cairo University, Cairo, Egypt

###### **Correspondence:** Ahmed Kamel Inas E. Mahfouz

*Clinical and Translational Allergy* 2016, **6(Suppl 1)**:OP08

**Background:** Asthma is a chronic airway inflammation characterized by being a heterogenous disease. Acupuncture is one of traditional Chinese medicine (TCM) modalities and it is considered the backbone of complementary and alternative medicine Laser acupuncture specially low-level laser therapy (LLLT) is a noninvasive form of phototherapy which being acceptable for treating children instead of needle acupuncture.

**Methodology:** Our study formed on sixty asthmatic children under ordinary medical treatment for bronchial asthma and they were divided randomly into 3 groups. Group A: Twenty children were given laser Acupuncture session three times per week for 1 month group B: twenty patients given needle acupuncture sessions by a rate same as laser group and group C (control group) under their asthmatic medications only. Assessing of the clinical condition of the patient (frequency of attack and severity of asthma) and laboratory (IgE level and Eosinophil count) before and after the study.

**Results:** Frequency of asthmatic attack diminished in group A (p < 0.001) more than in group B (p = 0.002) and least in group C (p = 0.147) at the end of the study, Clinical severity of group A significantly improved (p < 0.001) than group B and C, IgE level were significantly improved in both groups A and B (p < 0.001) better than changes occurred in group C (p = 0.057). Eosinophilic count showed more significantly improvement in group A (p < 0.001) than group B (p = 0.206) and group C (p = 0.784).

**Conclusions:** Application of laser acupuncture sessions beside medical treatment results in more significantly improvement of the asthma frequency of attack, the degree of asthma severity, IgE level and eosinophilic count in asthmatic children than the use of needle acupuncture or medications used only.

### OP09 The concentration of exhaled carbon monoxide in asthmatic children with different controlled stadium

#### Papp Gabor, Biro Gabor, Kovacs Csaba

##### Szigetvar Hospital, Szigetvar, Hungary

###### **Correspondence:** Papp Gabor

*Clinical and Translational Allergy* 2016, **6(Suppl 1)**:OP09

**Introduction:** The exhaled carbon monoxide is an important biomarker of the oxidative stress and the airways inflammation. Most scientific publications account of increase values in asthma. However our known literature does not have data on the relationship between different stadiums of asthma control and the exhaled carbon monoxide values. The aim of this study was to access this correlation.

**Method:** Our patients are well controlled, partly and uncontrolled asthma bronchial children, who are treated in outpatient clinic. Each of them has treatment according to GINA protocol. Before spirometry, patients were made deep inhalation, then they were made slow exhalation to the carbon monoxide measure equipment. (PiCO + Smokerlyzer) The exhaled carbon monoxide values were only known by the assistant, so that these values wouldn’t influence when putting in a category. We made the statistic by InStat softver. We used non-parametric procedures.

**Results:** We found significant differences between the groups of well controlled and group of partly or uncontrolled in concentration of exhaled carbon monoxide, but significant difference was not demonstrable between the group of partly controlled and group of uncontrolled (Table 1).

**Table 1:** Summary

	Well controlled	Partly controlled	Uncontrolled	Significance
Number of patients	99 (37 %)	102 (38 %)	68 (25 %)	
eCO (ppm)	3.21 (SD: 1.15)	4.88 (SD: 2.60)	5.55 (SD: 2.49)	P < 0.0001

**Conclusion:** According to our investigation/examinations the exhaled carbon monoxide is significantly higher in partly or uncontrolled stadium of asthma bronchial than in well controlled stadium of asthma. This higher amount of exhaled carbon monoxide values show the raising of airways inflammation and the finish of well controlled stadium. The exhaled carbon monoxide suggests the inflammation aspect of asthma bronchial. Detailed analysis of our results shows that the eCO can be used to estimate the compliance of patients (Table 2).

**Table 2:** Summary

	Controlled	Partly controlled	Uncontrolled	Significant relation
Patients number (male/female)	99 (37 %) (51/48)	102 (38 %) (62/40)	68 (25 %) (36/32)	–
Age	11.13	11.23	10.97	p > 0.05
FVC%	94.53	94.06	94.74	p > 0.05
FEV1%	95.29	92.24	91.57	p > 0.05
FEV1/FVC	88.24	85.68	85.41	P < 0.05 (p = 0.0186)
FEF25–75 %	94.82	85.95	86.65	P < 0.05 (p = 0.0163)
eCO/ppm/	3.21	4.88	5.55	P < 0.05 (p < 0.0001)

### OP10 Effect of vitamin D3 supplementation during pregnancy on risk of persistent wheeze in the offspring: a randomised clinical trial

#### Bo Chawes^1^, Klaus Bønnelykke^1^, Jakob Stokholm^1^, Lene Heickendorff^2^, Susanne Brix^3^, Morten Rasmussen^1^, Hans Bisgaard^1^

##### ^1^Danish Pediatric Asthma Center, Gentofte Hospital, University of Copenhagen, Gentofte, Denmark; ^2^Department of Clinical Biochemistry, Århus University Hospital, Århus, Denmark; ^3^Department of Systems Biology, Center for Biological Sequence Analysis, Technical University, Lyngby, Denmark

###### **Correspondence:** Bo Chawes

*Clinical and Translational Allergy* 2016, **6(Suppl 1)**:OP10

**Importance:** Observational studies have suggested that increased dietary Vitamin D intake during pregnancy may protect the offspring against preschool wheezing, which is the most common disorder in young children.

**Objective:** To determine whether supplementation of Vitamin D_3_ during third trimester of pregnancy reduces the risk of persistent wheeze in the offspring.

**Design, setting and participants:** The study was a double-blinded, single-center, randomized controlled trial conducted within the Copenhagen Prospective Study on Asthma in Childhood (COPSAC_2010_) unselected mother–child cohort. A total of 627 women were recruited for the Vitamin D trial at 24 weeks of pregnancy, between March 4th 2009 and November 17th 2010. Clinical follow-up of the children (n = 581) was completed when the youngest child turned 3 years and unblinded on March 28th 2014.

**Intervention:** Vitamin D_3_ (2400 IU/day) supplementation or matching placebo tablets from pregnancy week 24 to 1 week postpartum.

**Main outcome measure:** Persistent wheeze at age 0–5 years diagnosed solely by the intervention-blinded study pediatricians strictly adherent to a predefined algorithm based on 11 scheduled and additional acute clinic visits and a day-to-day symptom diary filled by the parents from birth. Secondary outcomes were number of wheezy episodes, asthma, neonatal airway immunology, respiratory infections, allergic sensitization and eczema.

**Results:** Occurrence of persistent wheeze did not differ between the Vitamin D_3_ supplement and control group (incidence, 18 % vs. 21 %; hazard ratio, 0.79; 95 % CI, 0.54–1.14, *P* = 0.21). The number of wheezy episodes was reduced by the Vitamin D_3_ intervention (mean 5.9 vs. 7.2 episodes; incidence risk ratio, 0.83; 95 % CI, 0.71–0.97, *P* = 0.02) and the airway immune profile at age one month was up-regulated (principle component analysis, *P* = 0.04). There was no effect on additional end-points.

**Conclusion:** The use of Vitamin D_3_ supplementation during pregnancy did not reduce the risk of persistent wheeze in the offspring.

### OP11 Lung function development in childhood

#### Henrik Wegener Hallas, Bo Chawes, Lambang Arianto, Hans Bisgaard

##### Copenhagen Prospective Studies on Asthma in Childhood, Copenhagen University Hospital, Gentofte, Denmark

###### **Correspondence:** Henrik Wegener Hallas

*Clinical and Translational Allergy* 2016, **6(Suppl 1)**:OP11

**Background:** We have previously shown that children who develop asthma in childhood have reduced lung function already at birth. The objective of this study was to examine if the inborn lung function deficit in asthmatic children is sustained until adolescence, even if symptoms cease.

**Methods:** This study included 366 children of the Copenhagen Prospective Studies on Asthma in Childhood (COPSAC_2000_) high-risk birth cohort. Lung function was measured repetitively from 0 to 13 years by spirometry (FEV_x_) and plethysmography (sRaw). Asthma was diagnosed solely by the COPSAC pediatricians according to a strict predefined algorithm based on symptom load and response to treatment. Age at onset and remission was monitored by daily diary cards.

Association analysis was performed using generalized estimating equation models.

**Results:** Children developing asthma during childhood compared to children never experiencing asthma symptoms had reduced forced expiratory flow: FEV_x_ (z-score): −0.31 [−0.47; −0.16], p < 0.0001, and increased airway resistance: sRaw (z-score): 0.41 [0.24;0.59], p < 0.0001. The lung function was reduced in the asthmatics already before onset of symptoms (FEV_x_ (z-score): −0.32 [−0.49; −0.14], p = 0.0004 and sRaw (z-score): 0.47 [0.21;0.72], p = 0.0003) and not improving after remission of symptoms (FEV_x_ (z-score): 0.03 [−0.03;0.07], p = 0.31 and sRaw (z-score): 0.02 [−0.03;0.07], p = 0.39).

The level of lung function was not correlated with the duration of symptoms (FEV_x_ (z-score): −0.03 [−0.10;0.04], p = 0.44 and sRaw (z-score): 0.01 [−0.05;0.07], p = 0.68).

**Conclusion:** The reduced lung function characterizing newborn children developing asthma later in childhood is sustained till adolescence independent of remission of symptoms and not further deteriorated by increased disease duration. This suggests that the lung function deficit accompanying asthma propensity is primarily an inherited trait.

### OP12 Is the effect of maternal and paternal asthma different in female and male children before puberty?

#### Maike Pincus^1^, Thomas Keil^2^, Andreas Reich^2^, Ulrich Wahn^1^, Susanne Lau^1^, Linus Grabenhenrich^2^

##### ^1^Department of Pediatric Pneumology and Immunology, Charité-Universitätsmedizin Berlin, Berlin, Germany; ^2^Institute of Social Medicine, Epidemiology and Health, Charité-Universitätsmedizin Berlin, Berlin, Germany

###### **Correspondence:** Maike Pincus

*Clinical and Translational Allergy* 2016, **6(Suppl 1)**:OP12

**Background:** Parental allergy is one of the strongest risk factors for asthma during childhood, but data on sex/gender-specific effects by maternal and paternal asthma separately are lacking. Our aim was to evaluate the prevalence of asthma in males and females before puberty by maternal and paternal asthma separately.

**Methods:** We analysed the prospective follow-up data from two decades of the urban multicenter birth cohort study MAS which recruited 1314 children in 5 German cities in 1990. Definition of current asthma was based on the presence of at least 2 of the following criteria: (i) wheeze in the last 12 months; (ii) asthma medication last 12 months; (iii) parent-/self-reported doctor diagnoses asthma ever.

**Results:** Among girls at age 9, 22 % (n = 11, 95 % CI 11–35 %) with an asthmatic mother had asthma themselves compared to 18 % (n = 6, 95 % CI 7–35 %) with an asthmatic father and 67 % (n = 4, 95 % CI 22–96 %) where both parents had asthma. The corresponding results in boys aged 9 were 17 % (n = 6, 95 % CI 7–33 %), 22 % (n = 8, 95 % CI 10–38 %) and none. In children without an asthmatic parent 4 % (n = 12, 95 % CI 2–6 %) of girls and 8 % (n = 30, 95 % CI 6–11 %) of boys had asthma at 9 years of age. Analyses of assessments in puberty and adolescence are currently ongoing.

**Conclusion:** The tendency of different sex-specific paternal and maternal effects on asthma at age 9 needs to be interpreted with caution (wide confidence intervals). More information will be gained by considering the MAS data from puberty and during adolescence up to age 20.

## ORAL ABSTRACT SESSION 3: Epidemiology—genetics (OP13–OP18)

### OP13 Lifestyle is associated with incidence and category of allergen sensitisation: the ALADDIN birth cohort

#### Sara Fagerstedt, Helena Marell Hesla, Emelie Johansson, Helen Rosenlund, Axel Mie, Annika Scheynius, Johan Alm

##### Karolinska Institutet, Stockholm, Sweden

###### **Correspondence:** Sara Fagerstedt

*Clinical and Translational Allergy* 2016, **6(Suppl 1)**:OP13

**Background:** Environmental and lifestyle factors are considered to contribute to the global increase in allergen sensitization. Pre- and post-natal periods are important time windows for immune system development. Lifestyle is associated with lower prevalence of allergic sensitization. We aimed to determine if the age at onset, and category of allergic sensitization, is associated with lifestyle.

**Methods:** Children (n = 474) from the prospective cohort study ALADDIN were followed from birth to 5 years. Families were divided into 3 lifestyle groups: anthroposophic (n = 100), partly anthroposophic (n = 209) and non-anthroposophic (n = 165). IgE-sensitization to food- (hen’s egg, cow’s milk and peanut), animal- (cat and dog) and pollen allergen (birch and timothy) was analyzed in blood samples from the children at 6, 12, 24 and 60 months of age.

**Results:** 118 developed an allergen sensitization up to 5 years of age. Out of these, 18 were from the anthroposophic-, 44 from the partly- and 56 from the non-anthroposophic group. The incidence rate in the children from families with an anthroposophic and partly anthroposophic lifestyle was significantly lower compared to the non-anthroposophic group up to 6 months. Up to 12 months, the incidence rate remained significantly lower in the anthroposophic group compared to the non-anthroposophic group. The differences were explained by the sensitization to food allergens in early infancy.

**Conclusions:** Anthroposophic lifestyle is associated with reduced risk of allergic sensitization in early childhood. This seems largely explained by the absence of food sensitization before one year of age. We propose that this lifestyle affects the pattern of the allergic march.

### OP15 Maternal filaggrin mutations increase the risk of atopic dermatitis in children: an effect independent of mutation inheritance

#### Jorge Esparza-Gordillo^1,2^, Anja Matanovic^1,2^, Ingo Marenholz^1,2^, Anja Bauerfeind^1^, Klaus Rohde^1^, Katja Nemat^3^, Min-Ae Lee-Kirsch^3^, Magnus Nordenskjöld^4^, Marten C.G. Winge^4^, Thomas Keil^5^, Renate Krüger^6^, Susanne Lau^6^, Kirsten Beyer^6^, Birgit Kalb^6^, Bodo Niggemann^6^, Norbert Hübner^1^, Heather J. Cordell^7^, Maria Bradley^4,8^, Young-Ae Lee^1,2^

##### ^1^Max-Delbrück-Centrum (MDC) for Molecular Medicine, Berlin, Germany; ^2^Clinic for Pediatric Allergy, Experimental and Clinical Research Center, Charité Universitätsmedizin Berlin, Berlin, Germany; ^3^Klinik fur Kinder- und Jugendmedizin, Technical University Dresden, Dresden, Germany; ^4^Department of Molecular Medicine and Surgery, Karolinska Institutet, Stockholm, Sweden; ^5^Institute for Social Medicine, Epidemiology and Health Economics, Charité Universitätsmedizin Berlin, Berlin, Germany; ^6^Pediatric Pneumology and Immunology, Charité Universitätsmedizin Berlin, Berlin, Germany; ^7^Institute of Genetic Medicine, Newcastle University, Newcastle upon Tyne, UK; ^8^Dermatology Unit, Department of Medicine, Solna Karolinska University Hospital, Stockholm, Solna, Sweden

###### **Correspondence:** Jorge Esparza-Gordillo

*Clinical and Translational Allergy* 2016, **6(Suppl 1)**:OP15

Epidemiological studies have shown that maternal allergy is a stronger risk factor for the offspring than paternal allergy, suggesting a preferential maternal transmission of disease risk. However, the molecular mechanism underlying this observation is currently unknown. Loss-of-function mutations in the filaggrin gene (*FLG*) cause skin barrier deficiency and strongly predispose to atopic dermatitis (AD). We analyzed the 4 most prevalent European *FLG* mutations (c.2282del4, p.R501X, p.R2447X, and p.S3247X) in two family-based samples (759 and 450 AD families) and applied the PREMIM/EMIM tool to test for parent-of-origin effects. As expected from the known role of these mutations on AD, children carrying a *FLG* mutation had a 2.4 fold increased disease risk (R1meta-analysis = 2.4, P = 1.0 × 10^−36^). Strikingly, we also observed a strong maternal *FLG* genotype effect indicating that children of *FLG*-carrier mothers had a 1.5-fold increased AD risk (S1 = 1.50, Pmeta-analysis = 8.4 × 10^−8^). This maternal effect that was consistent in both sets of families and for all 4 mutations analyzed. Our results point to two independent scenarios where *FLG* mutations increase AD risk: (i) carrying a mutation and (ii) having a mutation carrier mother. This maternal effect was independent of mutation inheritance and can be seen as a non-genetic transmission of a genetic effect. Interestingly, the *FLG* maternal effect was observed only when mothers had allergic sensitization, suggesting that *FLG*-induced changes in the maternal immune response shape the child’s immune system through feto-maternal cross-talk and increase the child’s risk for AD. Overall, our study indicates that maternal *FLG* mutations act as strong environmental risk factors for the child and highlights the potential of family-based studies in uncovering novel disease mechanisms in the field of allergy susceptibility.

### OP16 Allergic multimorbidity of asthma, rhinitis and eczema in the first 2 decades of the German MAS birth cohort

#### Thomas Keil, Hannah Gough, Linus Grabenhenrich, Dirk Schramm, Andreas Reich, John Beschorner, Antje Schuster, Carl-Peter Bauer, Johannes Forster, Fred Zepp, Young-Ae Lee, Renate Bergmann, Karl Bergmann, Ulrich Wahn, Susanne Lau

##### Charité-Universitätsmedizin Berlin, Berlin, Germany

###### **Correspondence:** Thomas Keil

*Clinical and Translational Allergy* 2016, **6(Suppl 1)**:OP16

The published version of this abstract can be found at [1].

**Reference**

1. Pediatr Allergy Immunol. 2015;26(5):431–7. http://onlinelibrary.wiley.com/doi/10.1111/pai.12410/abstract.

### OP17 Childhood anaphylaxis: a growing concern

#### Filipe Benito Garcia, Inês Mota, Susana Piedade, Ângela Gaspar, Natacha Santos, Helena Pité, Mário Morais-Almeida

##### Immunoallergy Department, CUF Descobertas Hospital, Lisbon, Portugal

###### **Correspondence:** Filipe Benito Garcia

*Clinical and Translational Allergy* 2016, **6(Suppl 1)**:OP17

**Background:** Anaphylaxis is a severe life-threatening condition, frequently underdiagnosed and undertreated. The incidence of anaphylaxis is increasing, especially among children. We aimed to examine the changes in anaphylaxis frequency and characteristics over a 5 year period.

**Methods:** Comparative analysis report on data from two cross-sectional independent samples of children with anaphylaxis, collected 5 years apart in 2006 (sample A) and 2011 (sample B). In both samples, we included patients <18 years with history of anaphylaxis, observed in an Immunoallergy Department in Lisbon, Portugal, during 12 months.

**Results:** The frequency of anaphylaxis was 0.98 % (A, n = 56/5734) and 1.76 % (B, n = 64/3646), respectively (p = 0.001). Median age was 7 years (A, 1–17 years; B, 18 days–17 years); 71 % (A) and 61 % (B) male. Median age of first anaphylactic episode was 2 years (A, 29 days–16 years) and 3 years (B, 4 days–17 years); 57 % (A) and 44 % (B) had history of asthma. Food-induced anaphylaxis occurred in 70 % (A) and 84 % (B) of children and drug-induced anaphylaxis was reported in 9 % (A) and 8 % (B). Less common triggers were cold exposure, latex and insect sting. Most children had no previous etiologic diagnosis [70 % (A); 73 % (B)]; anaphylaxis triggers were identified in all patients after proper clinical investigation. Clinical manifestations of anaphylaxis were similar in both samples: mucocutaneous [100 % (A); 94 % (B)] and respiratory [75 % (A); 84 % (B)] symptoms were more common than gastrointestinal [39 % (A); 42 % (B)] and cardiovascular [21 % (A); 25 % (B)] manifestations. In 89 % (A) and 88 % (B), the anaphylactic reaction occurred in the first 30 min after trigger exposure. Only about one-third of children with anaphylaxis had been treated with adrenaline [32 % (A); 33 % (B)].

**Conclusions:** The frequency of anaphylaxis was significantly higher in 2011; food allergy was the leading cause. The percentages of children with anaphylaxis with previous unknown etiologic diagnosis and treated with adrenaline were fairly similar in both time points. This data suggests the need for improved awareness and educational programs for the recognition and management of children with anaphylaxis.

### OP18 Indoor exposure to molds and dampness in infancy and its association to persistent atopic dermatitis in school age. Results from the Greek ISAAC II study

#### Athina Papadopoulou^1^, Despina Mermiri^2^, Elpida Xatziagorou^3^, Ioannis Tsanakas^3^, Stavroula Lampidi^1^, Kostas Priftis^4^

##### ^1^Allergy Pediatric Unit, KAT General Hospital, Athens, Greece; ^2^Allergology and Respiratory Unit, Penteli’s Children Hospital, Athens, Greece; ^3^3rd Pediatric Department, Aristoleleio University, Thessaloniki, Greece; ^4^Pediatric Allergy and Pulmonology Units, 3rd Department of Pediatrics, University of Athens General Hospital “Attikon”, Athens, Greece

###### **Correspondence:** Athina Papadopoulou

*Clinical and Translational Allergy* 2016, **6(Suppl 1)**:OP18

The presence of molds as a source of perennial allergens and multiple bacteria has been related to the appearance of respiratory symptoms in several studies. Yet, its role in eczema has not been elucidated.

**Aim:** The aim of this study was to investigate the association between exposure to indoor visible molds/dampness and the manifestation of eczema in children.

**Materials and methods:** The study is part of the Greek contribution to ISAAC IΙ that includes 2023 students of randomly selected public primary schools in Athens and Thessaloniki, aged 9–10 years. The children represented a general population sample and were evaluated according to ISAAC II questionnaire, validated for Greek language. Additionally, skin prick tests (SPT), to aero-allergens were performed and children were examined for active lesions.

**Results:** 13 % had suffered from eczema in the past, 9 % had current and 2 % atopic eczema (positive SPT). Out of the children examined, half reported that eczema first appeared after the age of 5 years whereas 68 % mentioned persistence of eczema since infancy. 10.8 and 6.4 % of children were currently exposed to indoor dampness and visible mold respectively, while 77, 5 % of them confirmed exposure since birth. 10 % of the sensitized children were positive to house dust mites and Alternaria. In logistic regression analysis evaluating 20 environmental risk factors, a significant association was noted between the presence of indoor visible mold and dampness in infancy, and the presence of current eczema OR 1, 89 (95 % CI 1.25–2.85). This association remained significant irrespective of the family history of atopy.

**Conclusions:** Frequently eczema first appears at early school age. The presence of visible mold and dampness at home during infancy appears to be an initial enhancing risk factor for the development but also for the persistence of the disease throughout school age.

## ORAL ABSTRACT SESSION 4: Pediatric rhinitis—immunotherapy (OP19–OP24)

### OP19 Associations between residential greenness and childhood allergic rhinitis and aeroallergen sensitisation in seven birth cohorts

#### Elaine Fuertes^1^, Iana Markevych^1,2^, Gayan Bowatte^3^, Olena Gruzieva^4^, Ulrike Gehring^5^, Allan Becker^6^, Dietrich Berdel^7^, Michael Brauer^8,9^, Chris Carlsten^8,9^, Barbara Hoffmann^10,11^, Anita Kozyrskyj^12,13^, Caroline Lodge^3^, Göran Pershagen^4^, Alet Wijga^14^, Heinrich Joachim^1^

##### ^1^Institute of Epidemiology I, Helmholtz Zentrum München – German Research Center for Environmental Health, Munich, Germany; ^2^Division of Metabolic and Nutritional Medicine, Ludwig Maximilians University of Munich, Dr. von Hauner Children’s Hospital, Munich, Germany; ^3^Allergy and Lung Health Unit, Melbourne School of Population and Global Health, The University of Melbourne, Melbourne, Australia; ^4^Institute of Environmental Medicine, Karolinska Institutet, Stockholm, Sweden; ^5^Institute for Risk Assessment Sciences, Utrecht University, Utrecht, The Netherlands; ^6^Department of Pediatrics and Child Health, University of Manitoba, Winnipeg MB, Canada; ^7^Research Institute, Department of Pediatrics, Marien-Hospital Wesel, Wesel, Germany; ^8^School of Population and Public Health, University of British Columbia, Vancouver BC, Canada; ^9^Department of Medicine, University of British Columbia, Vancouver BC, Canada; ^10^IUF – Leibniz Research Institute for Environmental Medicine, Düsseldorf, Germany; ^11^Heinrich-Heine University of Düsseldorf, Medical Faculty, Deanery of Medicine, Düsseldorf, Germany; ^12^Department of Pediatrics, Faculty of Medicine and Dentistry, Women and Children’s Health Research Institute, Edmonton AB, Canada; ^13^School of Public Health, University of Alberta, Edmonton AB, Canada; ^14^Center for Nutrition, Prevention and Health Services, National Institute of Public Health and the Environment, Bilthoven, the Netherlands

###### Correspondence: Iana Markevych

Clinical and Translational Allergy 2016, **6(Suppl 1)**:OP19

**Introduction:** Inconsistent associations have been reported between the green environment and childhood asthma and allergic health outcomes. We conducted a meta-analysis on residential greenness and allergic rhinitis and aeroallergen sensitization based on data from Swedish (BAMSE), Australian (MACS), Dutch (PIAMA), Canadian (CAPPS and SAGE) and German (GINIplus and LISAplus) birth cohorts (N = 13,016).

**Methods:** Allergic rhinitis (doctor diagnosis or symptoms) and aeroallergen sensitization were assessed in children aged 6–8 years in six cohorts and 10–12 years in five cohorts. Residential greenness was defined as the mean Normalized Difference Vegetation Index (NDVI) in 500 m and 1000 m buffers around the home address at the time of health assessment. Cohort-specific associations between NDVI (per 0.2 unit increase) and allergic rhinitis and aeroallergen sensitization were assessed using logistic regression models adjusted for host and environmental confounders. The findings were combined in a random-effects meta-analysis.

**Results:** Heterogeneous associations for a range of outcomes were observed across cohorts. Greenness in a 500 buffer was positively associated with allergic rhinitis at 6–8 years in BAMSE (odds ratio = 1.42, 95 % confidence interval [1.13, 1.79]) and GINI/LISA South (1.69, [1.19, 2.41]) but negatively associated in GINI/LISA North (0.61 [0.36, 1.01]) and PIAMA (0.67 [0.47, 0.95]). The direction of the effect estimates in the Canadian cohorts were also conflicting but not significant (0.63 [0.32, 1.23] and 1.22 [0.76, 1.95] for CAPPS and SAGE, respectively). All meta-analytic estimates were null. Results were similar for aeroallergen sensitization at 6–8 years and both outcomes at 10–12 years, and were independent of buffer size. Stratification by four urbanization markers (particulate matter smaller than 2.5 µm concentrations, nitrogen dioxide concentrations, population density and urban versus rural surroundings) did not reveal consistent trends within subgroups.

**Conclusions:** Although residential greenness appears to be associated with childhood allergic rhinitis and aeroallergen sensitization, the direction of the effect varies by location.

### OP20 Full symptom control in pediatric patients with allergic rhinitis and asthma: results of a 2-year sublingual allergen immunotherapy study

#### Zorica Zivkovic, Ivana Djuric-Filipovic, Jasmina Jocić-Stevanovic, Snežana Zivanovic

##### Children Hospital for Pulmonary Diseases and Tuberculosis, Belgrade, Serbia

###### **Correspondence:** Ivana Djuric-Filipovic

*Clinical and Translational Allergy* 2016, **6(Suppl 1)**:OP20

**Introduction:** Our study was designed to assess the efficacy of SLIT in allergic rhinitis and asthma in pediatric population. Here we report interim analysis done after 2 years. The aim of this study was to document the clinical implication of allergen specific drops on symptom severity and medication in children with allergic rhinitis and asthma.

**Methods:** In this observational case control study we have collected information from 59 children with AR or/and asthma. 34 children received SLIT drops plus symptomatic treatment whereas 25 of patients received only symptomatic therapy. During the follow up period the patients rate their symptoms (rhinitis, conjunctivitis, asthma and atopic dermatitis) as combined score of severity—traditional symptom score assessment (graded from 0 to 3) plus recording of doses of symptomatic medications. The beneficial effects of SLIT on asthma and rhinitis scores and medication use have been observed for two consecutive years of treatment.

**Results:** Both asthma and rhinitis scores were decreased during the first year of treatment, with the tendency of further decrease in the second year of follow up period. The most important effects of SLIT were observed for the symptom of wheezing and night cough (Χ^2^ = 56,790; p < 0.001, Χ^2^ = 56,142; p < 0.001) only in the experimental group. On the other side the biggest success of SLIT for rhinitis symptoms were detected for nasal congestion and rhinorrhea (Χ^2^ = 43,607; p < 0.001, Χ^2^ = 41,809; p < 0.001), without any significant changes in the control group. No differences have been detected for ocular and symptoms of atopic dermatitis. Additional significant improvements were also observed for symptomatic medication: antihistaminic: Χ^2^ = 32,774; p < 0.001, inhalation corticosteroids: Χ^2^ = 30,022; p < 0.001, intranasal corticosteroids: Χ^2^ = 30,785; p < 0.001, beta2agonist Q = 28,783; p < 0.001, LTRA: Q = 12,000; p = 0.002, but only in the experimental group.

**Conclusion:** The results of this study indicate favorable clinical efficacy of SLIT drops in children with asthma and allergic rhinitis. These findings also suggest that SLIT drops may have potential to alter the natural history of atopic disease with the convenience of home administration and the benefit of oral rather than subcutaneous administration.

### OP21 Nasal epithelium of different ages of atopic subjects present increased levels of oxidative stress and increased cell cytotoxicity upon rhinovirus infection

#### Styliani Taka^1^, Dimitra Kokkinou^1^, Aliki Papakonstantinou^1^, Panagiota Stefanopoulou^1^, Anastasia Georgountzou^1^, Paraskevi Maggina^1^, Sofia Stamataki^1^, Vassiliki Papaevanggelou^2^, Evangelos Andreakos^3^, Nikolaos G. Papadopoulos^1,4^

##### ^1^2nd Pediatric Clinic, University of Athens, Athens, Greece; ^2^3rd Pediatric Clinic, University of Athens, Attikon University Hospital, Athens, Greece; ^3^Biomedical Research Foundation, Academy of Athens, Athens, Greece; ^4^Centre for Pediatrics and Child Health, Institute of Human Development, University of Manchester, Manchester, UK

###### **Correspondence:** Styliani Taka

*Clinical and Translational Allergy* 2016, **6(Suppl 1)**:OP21

**Background:** The respiratory epithelium is critical both for the clearance of infections and the development of adaptive responses. There is still a significant gap of knowledge on the interplay between maturation of innate immunity, exposure and vulnerability to infections in the development of allergic responses. The aim of the present study was to determine the role of viral infections in the maturation of the immune response in relation to age.

**Method:** Primary nasal epithelial cells (PNECs) were derived from non atopic (n = 17) and atopic (n = 13) individuals (1–48 years). The existence of atopy was assessed by skin prick testing to common aeroallergens or/and egg white. PNECs were cultured and infected with Human Rhinovirus A1B (HRVA1B). Doubling time of PNECs was assessed using Least Squares Fitting. Reactive oxygen species (ROS) were measured at 1 h, 3 h, 24 h post infection with flow cytometry. Virus replication evaluated using titration method at 8 h, 24 h, 48 h and 72 h. CCL5 release was examined at 48 h using ELISA. Cytotoxicity levels were evaluated at 24 h, 48 h and 72 h with crystal violet staining. Statistic analysis was performed using Linear regression analysis and oneway ANOVA.

**Results:** Epithelial cell growth rate was affected by age and specifically atopic children have higher doubling time than atopic adults. Also atopic individuals seem to have increased production of ROS at baseline and CCL5 production. PNECs from atopic individuals have increased induction of ROS at 3 h and 24 h (p < 0.05) post infection and higher virus replication at 72 h (p < 0.1). Age is a factor that influence ROS production, CCL5 release and cytotoxicity after HRA1VB infection (p < 0.05). Males produce higher levels of ROS in combination with increased cytotoxicity (p < 0.05).

**Conclusion:** This is the first study investigating the development of cell growth and ROS generation comparing atopic and non-atopic individuals. Age, atopy and gender are important factors to rhinovirus infection in PNECs.

### OP22 Cluster subcutaneous immunotherapy schedule: tolerability profile in children

#### Monica Piquer Gibert, Montserrat Alvaro Lozano, Jaime Lozano Blasco, Olga Domínguez Sánchez, Rosa Jiménez Feijoo, Marcia Dias da Costa, M^a^ Teresa Giner Muñoz, Adriana Machinena Spera, Ana Maria Plaza Martín

##### Sant Joan de Déu Hospital, Barcelona, Spain

###### **Correspondence:** Monica Piquer Gibert

*Clinical and Translational Allergy* 2016, **6(Suppl 1)**:OP22

**Background:** Subcutaneous immunotherapy (SCIT) treatment begins with an allergen dose increase phase over a period of several weeks, followed by a maintenance phase. The aim of our study was to test the tolerance of a cluster schedule.

**Methods:** We recruited pediatric patients diagnosed with asthma and/or rhinoconjunctivitis due to mites or *alternaria* in which immunotherapy was indicated. A SCIT with non-modified allergens with aluminium hydroxide as adjuvant was chosen. On the 1st day patients received two injections (6000 and 9000SQ+) within 30 min. Spirometry was performed baseline and 30 min after the second dose or/and at any time if respiratory symptoms appeared. A maintenance dose of 15000SQ+ was administered after 28 days, spirometry was performed baseline and if respiratory symptoms appeared. Adverse events were recorded. Demographic data were collected.

**Results:** Ninety-five children (31 females) between age 5 and 18 years were evaluated. History of allergic disease: drug allergy 1 %, food allergy 7 %, atopic dermatitis 27 %, and no other allergic diseases 65 %. 49/95 were monosensitised. 69 patients had asthma, 59 of them also rhinoconjunctivitis, and 26 only rhinoconjunctivitis. Patients were treated with a SCIT formulation with *alternaria* in 12, *D. pteronyssinus* in 18, and a mix of mites in 65. Local and systemic reactions were reported by 2.1 % (2/95) of the patients. Both local reactions were delayed reactions (injection site swelling and erythema). Systemic reactions occurred within 30 min after the second dose on the first day. One patient had a mild asthma reaction, resolved with inhaled salbutamol, and tolerated subsequent doses. The second patient had a moderated asthma reaction, treated with inhaled salbutamol and oral corticosteroids, SCIT was withdrawn as any dose above 6000SQ+ produced asthma.

**Conclusions:** This cluster schedule has a good safety profile in children. SCIT fast protocols improve compliance by reducing injections, appointments, and school/work absences.

### OP23 Rhinitis as a risk factor for asthma severity in 11-year old children: population-based cohort study

#### Matea Deliu, Danielle Belgrave, Angela Simpson, Adnan Custovic

##### University of Manchester, Manchester, UK

###### **Correspondence:** Matea Deliu

*Clinical and Translational Allergy* 2016, **6(Suppl 1)**:OP23

The published version of this abstract can be found at [1].

**Reference**

1. Allergy 2013;68 (Suppl 97):1–104. http://dspace.uevora.pt/rdpc/bitstream/10174/10128/1/all12247_OralPresent_EAACI2013.pdf.

### OP24 The Global Lung Function Initiative equations in airway obstruction evaluation of asthmatic children

#### João Gaspar Marques, Pedro Carreiro-Martins, Joana Belo, Sara Serranho, Isabel Peralta, Nuno Neuparth, Paula Leiria-Pinto

##### Immunoallergy Department, Hospital de Dona Estefânia CHLC EPE, Lisbon, Portugal

###### **Correspondence:** João Gaspar Marques

*Clinical and Translational Allergy* 2016, **6(Suppl 1)**:OP24

**Methods:** A retrospective analysis of the children (6–18 years-old) with a medical diagnosis of asthma that performed spirometry during 2014 in our lung function laboratory was made. GLI and Zapletal equations agreement was compared in three different obstruction criteria: FEV_1_ < 80 % predicted, FEV_1_ < LLN (lower limit of normal) and FEV_1_/FVC < LLN. All the agreement analyses were performed using Cohen’s kappa test.

**Results:** 391 children were evaluated (61 % boys) with a mean age of 12.4 years (standard-deviation ±3.1 years). Considering Zapletal equations the percentages of children classified as obstructed according to the different analyzed parameters were: 7.7 % for FEV_1_ < 80 % predicted, 8.7 % for FEV_1_ < LLN and 26.3 % for FEV_1_/FVC < LLN. Assuming GLI 2012 equations the correspondent percentages were 12.0 % for FEV_1_ < 80 % predicted, 11.8 % for FEV_1_ < LLN and 29.2 % for FEV_1_/FVC < LLN. Using the FEV_1_/FVC < 0.70 ratio criteria 10.2 % of the patients were classified as obstructed. Cohen’s kappa coefficients for agreement between Zapletal and GLI 2012 equations to the analyzed parameters were: 0.67 for FEV_1_ < 80 % predicted, 0.64 for FEV_1_ < LLN and 0.85 for FEV_1_/FVC < LLN.

**Conclusions:** In our study there was a good agreement between the commonly used prediction equations (Zapletal equations) and GLI 2012 equations. GLI 2012 equations adoption is a reasonable option in asthmatic children lung function evaluation, although maybe the changes in nowadays clinical practice will not be considerable.

## POSTER DISCUSSION SESSION 1: Food allergy (PD01–PD05)

### PD01 Allergen-specific humoral and cellular responses in children who fail egg oral immunotherapy due to allergic reactions

#### Marta Vazquez-Ortiz^1^, Mariona Pascal^2^, Ana Maria Plaza^1^, Manel Juan^2^

##### ^1^Pediatric Allergy Section, Sant Joan de Deu Hospital, Barcelona, Spain; ^2^Immunology Department, Hospital Clinic, Barcelona, Spain

###### **Correspondence:** Marta Vazquez-Ortiz

*Clinical and Translational Allergy* 2016, **6(Suppl 1)**:PD01

**Introduction:** Oral immunotherapy (OIT) may trigger significant allergic reactions. Our ability to identify children who are unable to undergo OIT safely is limited.

**Aim:** To characterize allergen-specific humoral and cellular responses in children who required “Early Discontinuation” (named “ED group”) due to allergic reactions, versus those who successfully completed egg-OIT (“non ED group”).

**Methods:** Children aged 5–16 years with DBPCFC-confirmed IgE-mediated egg allergy were recruited for egg-OIT. Ovalbumin (OVA) and ovomucoid (OVM)- specific serum IgE, IgG4 and IgA were determined by ImmunoCAP (Thermofisher Scientific, Sweden) at baseline. In a subset of patients, PBMCs (2 × 10^5^) were isolated and cultured (in duplicate) for 7 days at 37 °C in 5 % CO_2_ with OVA and OVM (both at 100 ug/ml, Sigma Aldrich, Germany) with phytohaemaglutinin and medium alone as positive and negative controls respectively. After centrifugation, supernatants were collected and analyzed for presence of IL-2, IL-4, IL-5, IL-13, IFNg, IL-10, IL-9, IL-17, IL-6 and TNFa by multiplex magnetic bead assay (Luminex, Invitrogen, Life technologies, USA). Comparative analysis between ED and non ED groups was performed using U-Mann-Withney test. A two-tailed p value <0.05 was considered significant.

**Results:** 50 children underwent egg-OIT, 9 of whom required “Early Discontinuation” (ED group). Specific IgE, IgG4 and IgA to OVA and OVM were higher in ED vs. non ED group (p < 0.005). IL-9 production by PBMC following OVM stimulation was higher in ED (n = 6) vs. non ED group (n = 10, p < 0.006) and a similar trend was detected after OVA. IL-5 and IL-13 responses to OVM also tended to be higher in ED vs. non ED children. No differences were observed in other cytokines.

**Conclusion:** An IL-9 predominant cellular response as well as a strong allergen-specific poly-isotypic antibody response characterizes egg-allergic children who fail OIT at early stages due to significant allergic reactions.

### PD02 FoxP3 epigenetic features in children with cow milk allergy

#### Lorella Paparo^1^, Rita Nocerino^1^, Rosita Aitoro^1^, Ilaria Langella^1^, Antonio Amoroso^1^, Alessia Amoroso^1^, Carmen Di Scala^1^, Roberto Berni Canani^1,2^

##### ^1^Department of Translational Medical Science, University of Naples “Federico II”, Naples, Italy; ^2^CEINGE Advanced Biotechnologies, University of Naples “Federico II”, Naples, Italy

###### **Correspondence:** Lorella Paparo

*Clinical and Translational Allergy* 2016, **6(Suppl 1)**: PD02

**Background and aim:** Epigenetic changes in DNA methylation have been recently demonstrated during cow milk allergy (CMA) disease course (*Berni Canani R* et al*. Clin Epigenet, 2015*). The suppressive phenotype of regulatory T (Treg) cells, characterized by stable expression of transcription factor *Forkhead box Protein 3* (FoxP3), is involved in oral tolerance acquisition. We aimed to assess whether tolerance acquisition in children with IgE-mediated CMA involves a different DNA methylation profile of FoxP3.

**Methods:** DNA methylation of CpGs in the promoter regions and respective mRNA levels of FoxP3 from peripheral blood mononuclear cells (PBMCs), were assessed in children with active IgE-mediated CMA (Group 1), CMA subjects with recent evidence of oral tolerance acquisition (Group 2) and in healthy controls (Group 3).

**Results:** 37 children (18 male, aged 3–18 months) were enrolled: 16 in Group 1, 10 in Group 2 and 11 Group 3. DNA methylation profiles of FoxP3 clearly separated active CMA patients from healthy controls. Active IgE-mediated CMA patients showed significantly higher rate of DNA methylation in FoxP3 promoter region compared to healthy controls (52 vs. 80 %; *p* < 0.0001). DNA methylation analysis of this transcription factor clearly separated CMA patients by disease-state: tolerant subjects presented a significant different DNA methylation profile compared with active CMA patients (42 vs. 80 %, *p* < 0.0001). This profile was similar but not identical to that observed in healthy controls. A strong correlation between gene promoter DNA methylation rates and respective mRNA levels was also demonstrated (R^2^ = 0.946; *p* < 0.0001).

**Conclusion:** Tolerance acquisition in children with IgE-mediated CMA involves demethylation of promoter region of Foxp3 gene leading to an increased expression in Tregs. This feature could be a new epigenetic biomarker to follow the CMA disease course.

### PD04 Combined milk and egg allergy in early childhood: let them eat cake?

#### Santanu Maity, Giuseppina Rotiroti, Minal Gandhi

##### Royal Free London NHS Trust, London, UK

###### **Correspondence:** Santanu Maity

*Clinical and Translational Allergy* 2016, **6(Suppl 1)**:PD04

**Background:** Milk and egg allergies are the commonest food allergies in early childhood. Over 75 % may tolerate baked products avoiding unnecessary dietary restrictions and accelerating tolerance.

**Objective:** To audit our use of casein and ovomucoid recombinants in patients with combined milk and egg allergy to aid early dietary introduction of baked products.

**Method:** Caesin and ovomucoid recombinant testing on combined milk and egg allergic children, not previously exposed to baked products. Challenge selections included consideration of current published predictive data for SPT and recombinant tests.

**Results:** 67 children, aged 5–74 months (median 9.5) were tested.

*Milk allergy:* 18/19 (94.7 %) children passed the baked milk challenge. The SPT milk solution was 0–10 mm (median 3.0 mm), SPT raw milk 0–23 mm (median 10 mm), casein 0–14.2 KU/l (median 0.59 KU/l, mean 2.54 KU/l). 2/18 children who passed had a milk solution SPT of 6 mm or above but with a casein of 1.46 and 2.13. *GG allergy:* 18/21 (85.7 %) children passed the baked egg challenge. The SPT egg solution was 2–18 mm (median 8.0 mm), SPT raw egg 7–22 mm (median 10 mm), ovomucoid 0–4.56 KU/l (median 0.24, mean 0.59). 14/18 children had an egg solution SPT of 5 mm or above. These children’s’ ovomucoid was 0–4.56 KU/l (median 0.36, mean 0.65). 3 children failed their baked egg challenge (SPT egg solution 11 mm, 6 mm & 4 mm, ovomucoid 28.8 KU/l, 0.0 KU/l, 0.79 KU/l). Two of these children are now tolerating baked egg. 33 children were not challenged. Their median SPT to milk solution was higher (median 6.0) as was the caesin (median 16.6). The egg solution SPT was similar (median 10) with higher ovomucoid levels (median 12).

**Conclusion:** Caesin and Ovomucoid recombinants are useful additional predictive tools to aid patient selection for early introduction of dietary baked milk and egg.

### PD05 Introduction of complementary foods in relation to allergy and gut microbiota in farm and non-farm children

#### Karin Jonsson^1^, Annika Ljung^2^, Bill Hesselmar^2^, Ingegerd Adlerbert^2^, Hilde Brekke^3^, Susanne Johansen^4^, Agnes Wold^2^, Ann-Sofie Sandberg^1^

##### ^1^Chalmers University of Technology, Gothenburg, Sweden; ^2^University of Gothenburg, Gothenburg, Sweden; ^3^University of Oslo, Oslo, Norway; ^4^Skaraborg Hospital, Lidköping, Sweden

###### **Correspondence:** Karin Jonsson

*Clinical and Translational Allergy* 2016, **6(Suppl 1)**:PD05

**Background and aim:** Evidence is emerging that postponed introduction of complementary foods might increase the risk of allergy. Likewise, low “food diversity” in early age has been associated with allergy development. Interestingly, also low diversity of the early gut microbiota has been linked to increased allergy risk. The incidence of allergy is markedly low in farm children; our aims are to investigate introduction practices in relation to: (1) farm environment, (2) allergy development, and (3) gut microbiota.

**Methods:** Subjects from the FARMFLORA birth-cohort, including 28 farm and 37 non-farm parent-children pairs, were investigated. Practices of breastfeeding, formula and complementary feeding were recorded; month of introduction was registered for potatoes, vegetables, fruits, berries, nuts, peanuts, legumes, eggs, fish, meat, milk and flour. Allergies were diagnosed by doctors. Timing of introduction of complementary foods was analyzed in farm and non-farm infants and related to allergy development. Additionally, prevalence of microbes commonly found in the infant gut microbiota was analyzed on a genus level up to 18 months of age and will be related to formula and food introduction practices.

**Preliminary results:** Nuts were introduced earlier to farm children than non-farm children and flour was more frequent introduced to farm children at 7 months. In contrast, peanuts were less frequent introduced to farm children at 17 months. When allergic (1 farmer, 10 non-farmers) and healthy children were compared, fish was more frequent introduced to healthy infants at 10 months; the association was strengthened in children of non-allergic mothers but weakened when the analysis was confined to non-farm children. Legumes were more frequent introduced to healthy infants at 8 months, although non-significantly when maternal heredity was considered. Eggs were less frequent introduced to healthy infants at 11 months; the significance disappeared when considering maternal heredity. No differences in food diversity were observed at 6 months, or in total up to 18 months, between farm and non-farm children or between healthy and allergic children. Microbial analyses are ongoing.

## POSTER DISCUSSION SESSION 2: Asthma and wheeze (PD06–PD16)

### PD06 The association between asthma and exhaled nitric oxide is influenced by genetics and sensitisation

#### Björn Nordlund, Cecilia Lundholm, Villhelmina Ullemar, Marianne van Hage, Anne Örtqvist, Catarina Almqvist

##### Karolinska Institutet, Stockholm, Sweden

###### **Correspondence:** Björn Nordlund

*Clinical and Translational Allergy* 2016, **6(Suppl 1)**:PD06

**Background:** Rationale was to enhance knowledge about fraction of exhaled nitric oxide (FeNO) as a biomarker. The aim was to analyze the association between asthma and FeNO, and to take genetics, inhaled corticosteroids (ICS), and sensitization into account.

**Methods:** The population based STOPPA study of 681 twins (53 % monozygotic (MZ) and 47 % dizygotic (DZ), average age 12.6; SD 1.5) was used. Measurements were FeNO (parts per billion), parental report of current asthma with and without ICS and airborne sensitization (allergen-specific immunoglobulin E ≥ 0.35 kU_A_/l). The association between FeNO and asthma was analyzed between and within twin pairs (DZ and MZ) in regression models, the latter giving adjustment for shared environmental and genetic factors. Regression coefficients (β) and 95 % confidence interval (CI) per unit increase of log-transformed FeNO were presented.

**Results** demonstrated an association between current asthma and FeNO in between pairs analysis; β 0.31 (0.13–0.50), which was significantly stronger within DZ; β 0.41 (0.17–0.64), compared to MZ; β 0.07 (−0.18–0.31), and DZ vs. MZ, (p = 0.049). This indication of genetic confounding remained in twins without regular treatment with ICS (p = 0.022), but not in those with regular ICS. There was no increase in FeNO in children with asthma but without sensitization in between pairs analysis; β −0.01 (−0.27–0.24), compared to children with neither asthma nor sensitization. However, sensitization only, β 0.33 (0.14–0.53), and together with asthma, β 0.62 (0.32–0.92) were associated with increased FeNO.

**Conclusions:** This study shows that genetics and sensitization are strong influences for the association between asthma and FeNO.

### PD09 Prevalence patterns of infant wheeze across Europe

#### Anna Selby^1^, Kate Grimshaw^1^, Thomas Keil^2^, Linus Grabenhenrich^2^, Michael Clausen^3^, Ruta Dubakiene^4^, Alessandro Fiocchi^5^, Marek Kowalski^6^, Nikos Papadopoulos^7^, Marta Reche^8^, Sigurveig Sigurdardottir^3^, Aline Sprikkleman^9^, Paraskevi Xepapadaki^7^, Clare Mills^10^, Kirsten Beyer^2^, Graham Roberts^1^

##### ^1^University of Southampton, Southampton, UK; ^2^Charité Universitätsmedizin, Berlin, Germany; ^3^Landspitali University Hospital, Reykjavik, Iceland; ^4^Vilnius University, Vilnius, Lithuania; ^5^Pediatric Hospital Bambino Gesu, Rome, Italy; ^6^Medical University of Lodz, Lodz, Poland; ^7^University of Athens, Athens, Greece; ^8^Sofia Children’s University Hospital, Madrid, Spain; ^9^Emma Children’s Hospital, Amsterdam, the Netherlands; ^10^University of Manchester, Manchester, UK

###### **Correspondence:** Anna Selby

*Clinical and Translational Allergy* 2016, **6(Suppl 1)**:PD09

**Aim:** Pre-school wheeze is a significant health problem worldwide. This analysis aimed to assess prevalence patterns of wheeze in the first 2 years of life and identify how risk factors for this vary across Europe focusing on food allergy, breastfeeding and smoke exposure.

**Methods:** Children from nine European countries were recruited into the EuroPrevall birth cohort between 2005 and 2010. At recruitment, data were collected on birth details, familial allergies, socio-demographic status and environmental exposures, including cigarette smoke. At 12 and 24 months, data on feeding, symptoms and signs of allergic disease, wheeze, cigarette smoke exposure, infections and day care attendance were collected. The primary outcome for this study was wheeze in the second year of life using Poisson regression to identify risk factors (STATA SE 13).

**Results:** The EuroPrevall birth cohort included 12049 infants. Data on wheeze in the second year of life was available in 8775 (72.8 %). Prevalence rates varied across Europe with a broadly north-western to south-eastern gradient (Table 3). In multivariate analysis of the whole cohort, male gender, maternal asthma, maternal smoking, day care attendance and frequent respiratory tract infections were identified as risk factors for wheeze in the second year of life. Analysis of individual country data revealed different risk factor patterns. Food allergy was only associated with wheeze in univariate analysis. Breastfeeding did not appear to protect against wheeze.

**Table 3:** Prevalence of wheeze in the second year of life by country

Nordic	Maritime		Central European			Mediterranean			All
Iceland	UK	Netherlands	Germany	Poland	Lithuania	Spain	Italy	Greece	
17.2 %	13.1 %	10.8 %	11.8 %	1.7 %	1.9 %	3.0 %	9.5 %	2.8 %	7.8 %

**Conclusions:** In this study, the prevalence of wheeze in the second year of life varied considerably across Europe. A priori risk factors for preschool wheeze did not explain this variation. Other factors may be operating within countries.

### PD10 Epidemiologic changes in recurrent wheezing infants

#### Herberto Jose Chong Neto^1^, Gustavo Falbo Wandalsen^2^, Ana Carolina Dela Bianca^3^, Carolina Aranda^2^, Nelson Augusto Rosário^1^, Dirceu Solé^2^, Javier Mallol^4^, Luis García Marcos^5^

##### ^1^Federal University of Paraná, Curitiba, Brazil;^; 2^Federal University of São Paulo, São Paulo, Brazil; ^3^Federal University of Pernambuco, Recife, Brazil; ^4^University of Santiago de Chile, Santiago, Chile; ^5^University of Murcia, Murcia, Spain

###### **Correspondence:** Herberto Jose Chong Neto

*Clinical and Translational Allergy* 2016, **6(Suppl 1)**:PD10

The published version of this abstract can be found at [1].

**Reference**

1. Allergy 2015;70(Suppl S101):409–506. http://onlinelibrary.wiley.com/doi/10.1111/all.12720/abstract;jsessionid=A4A3CDF96C0F7611D97102B65A050C49.f02t04.

### PD13 A single nucleotide polymorphism in the GLCCI1 gene is associated with response to asthma treatment in children

#### Ivana Banic^1^, Matija Rijavec^2^, Davor Plavec^1^, Peter Korosec^2^, Mirjana Turkalj^1^

##### ^1^Children’s Hospital Srebrnjak, Zagreb, Croatia; ^2^University Clinic of Respiratory and Allergic Diseases Golnik, Golnik, Slovenia

###### **Correspondence:** Ivana Banic

*Clinical and Translational Allergy* 2016, **6(Suppl 1)**:PD13

The published version of this abstract can be found at [1].

**Reference**

1. Allergy. 2015;70(Suppl S101):113–279. http://onlinelibrary.wiley.com/doi/10.1111/all.12717/abstract.

### PD14 Pollen induced asthma: Could small molecules in pollen exacerbate the protein-mediated allergic response?

#### Alen Bozicevic, Maria De Mieri, Matthias Hamburger

##### University of Basel, Basel, Switzerland

###### **Correspondence:** Alen Bozicevic

*Clinical and Translational Allergy* 2016, **6(Suppl 1)**:PD14

**Background:** Plant pollen are known to be strong airborne elicitors of asthma in humans, and the role of allergenic proteins in the allergic response is well established. To better understand the exacerbation episodes of asthma in patients, other pathophysiologic mechanisms based on airways mechanics need to be considered. Both mechanisms regulated by cation channels such as TRPA1 and by the lipid kinase PIP5Kγ, regulate the intracellular Ca^2+^ concentration, having as an effect the smooth muscle contractionand consequent airways constriction. To explore a possible contribution of non-allergenic small molecules in pollen to the clinical outcome of asthma, we analyzed and compared the phytochemical profiles of pollen originating from *Ambrosia artemisiifolia* and other 29 plant species causing pollen allergenicity of varying severity.

**Methods:** Profiling was performed with high performance liquid chromatography (HPLC) coupled with electrospray ionization mass spectrometry (ESIMS), photodiode array (PDA) and evaporative light scattering (ELSD) detectors, and supported by microprobe nuclear magnetic resonance (NMR) spectroscopy and spectrophotometric analysis.

**Results:** The presence of conjugated polyamineswas a characteristic feature of pollen from the family of Asteraceae (*Ambrosia* and *Artemisia* ssp.). Compounds with Michael acceptor properties, such as sesquiterpene lactones (STLs) were present in pollen of different families.

**Conclusion:** Polyamines, such as spermine and spermidine, activate the lipid kinase PIP5Kγ. Sesquiterpene lactones activate cation channel TRPA1. Thus, the possible contribution of these small molecules in the exacerbation of airway constriction after exposure to plant aeroallergenes should be explored in more detail. The immunologic modulation through allergens might not be the only responsible for the asthma exacerbation episodes.

### PD15 A qualitative study to understand how we can empower teenagers to better self-manage their asthma

#### Simone Holley^1^, Ruth Morris^2^, Frances Mitchell^3^, Rebecca Knibb^4^, Susan Latter^1^, Christina Liossi^1^, Graham Roberts^1,2^

##### ^1^University of Southampton, Southampton, UK; ^2^University Hospital Southampton NHS Foundation Trust, Southampton, UK; ^3^St Mary’s Hospital, Newport, UK; ^4^University of Aston, Birmingham, UK

###### **Correspondence:** Simone Holley

*Clinical and Translational Allergy* 2016, **6(Suppl 1)**:PD15

**Background:** Teenagers with asthma often find it difficult to manage their asthma and there is little robust evidence on how health care professionals can facilitate teenagers’ efforts.

**Aim:** To understand the facilitators and barriers to asthma self-management in teenagers.

**Methods:** We recruited teenagers aged 12–18 years with asthma to take part in either a focus group or one-to-one interview as preferred by the participants. Separate focus groups and interviews were also conducted with their parents and healthcare professionals (HCP-pediatrician, nurse, family doctor). A thematic data analysis was undertaken.

**Findings:** 28 teenagers (1 focus group; 22 1:1 interviews), 12 parents (2 focus groups; 4 interviews) and 13 health professionals (2 focus groups, 3 interviews) were recruited. Themes included: 1) medication: forgetting, being prepared, need for routines or reminders, device issues, insufficient time; 2) symptom management: breathing techniques, keeping calm, recognising symptoms; 3) trigger avoidance: avoiding triggers, preparing for unavoidable triggers; 4) beliefs about medication and asthma: effectiveness of medication, need for medication, lack of control, outgrowing asthma; 5) attitudes to asthma: motivation, acceptance, seriousness, embarrassment, confidence, taking responsibility; 6) knowledge: consequences of not taking medication, understanding of condition and understanding of treatment, others not understanding asthma; 7) parents: reminders, education and information, monitoring, communication with HCP, providing support, help with treatments; 8) HCP: providing treatment, education and information, support, communication, conflicting and inaccurate information, honesty, lack of action, clinic setup; 9) others: friends, schools/teachers. In general, similar issues were voiced by the adolescents, parents and HCP.

**Discussion:** The potential facilitators and barriers to asthma self-management were similar to themes that were identified in our recent narrative systematic review of the literature (Holley et al., submitted). These could be targeted to support teenagers to become empowered to self-manage their asthma through a self-efficacy model of self-management.

**Funding:** Asthma UK.

### PD16 Polymorphism of endothelial nitric oxide synthase (eNOS) gene among Egyptian children with bronchial asthma

#### Mostafa M. M. Hassan

##### Medical Biochemistry, Faculty of Medicine, Kasr Alainy Hospital, Cairo University, Cairo, Egypt

###### **Correspondence:** Mostafa M. M. Hassan

*Clinical and Translational Allergy* 2016, **6(Suppl 1)**:PD16

**Introduction:** Bronchial asthma is a pulmonary disease characterized by chronic inflammation of the airways and bronchial hyperresponsiveness. Asthma is a multifactorial disease influenced by genetic and environmental factors. Nitric oxide (NO) has recently attracted attention in the pathophysiology of bronchial asthma. NO has protective effects on airways such as muscle relaxation & attenuation of airway hyper-responsiveness to bronchoconstrictor stimuli. In contrast, adverse effects of NO include vasodilation of the bronchial circulation & increased airway secretions. NO is synthesized by the enzyme NO synthase (NOS), which exists in three distinct isoforms: neural (nNOS), inducible (iNOS) and endothelial (eNOS).

**Purpose:** To investigate the association between Glu298Asp polymorphism of eNOS gene (rs1799983) and development of bronchial asthma in children from Egypt.

**Methods:** 93 asthmatic children who were able to perform pulmonary function tests efficiently & had a history of mild persistent asthma were included in this study. In addition, ninety healthy age matched subjects who had neither clinical evidence nor personal or familial history of asthma, served as control group. All participants were subjected to thorough medical history taking, clinical examination, pulmonary function testing, and estimation of eNOS (Glu298Asp) gene polymorphism using conventional PCR.

**Results:** A statistically significant difference was observed as regards genotype distribution of the Glu298Asp polymorphism between children with and without bronchial asthma. The prevalence of the T allele was significantly higher in asthmatic groups in comparison to control group.

**Conclusion:** This study showed the presence of a strong association between Glu298Asp polymorphism of the eNOS gene and the risk of development of bronchial asthma among Egyptian children. Genetic associations are often inconsistent across ethnic barriers. The results of this study provide the rationale for further studies with larger sample sizes.

## POSTER DISCUSSION SESSION 3: Mechanisms—Epidemiology (PD17–PD21)

### PD17 Pregnancy outcomes in relation to development of allergy in a Swedish birth cohort

#### Malin Barman^1^, Anna Sandin^2^, Agnes Wold^3^, Ann-Sofie Sandberg^1^

##### ^1^Chalmers University of Technology, Gothenburg, Sweden; ^2^Umeå University, Umeå, Sweden; ^3^Gothenburg University, Gothenburg, Sweden

###### **Correspondence:** Malin Barman

*Clinical and Translational Allergy* 2016, **6(Suppl 1)**:PD17

**Background:** It is suggested that the infantile period is crucial for immune modulation. Pregnancy outcomes, such as gestational age and birth weight, have been associated with risk for developing allergic disease later in life.

**Aim:** To analyze the influence of gestational age, birth weight, birth length and head circumference at birth on later allergy development in children born at term in a Swedish birth-cohort (BAS).

**Method:** All 1231 children born during a 1-year period from February 1996 to January 1997 at the Östersund Hospital in Jämtland, Sweden, were included in the BAS cohort and were followed from pregnancy until 13 years of age, with regular assessments of allergic sensitisation by skin prick tests and allergic symptoms by questionnaires. Data regarding gestational age, birth weight, birth length, and head circumference at birth, were derived from the Swedish Medical Birth Register, and related to atopic eczema and respiratory allergy at 13 years of age in children born at term (gestational week 37–42, *n* = 1052).

**Results:** Higher gestational age was a risk factor for developing respiratory allergy at 13 years of age (OR = 1.20, *P* = 0.041). No association was found for gestational age and prevalence of atopic eczema at 13 years of age. No association was found for birth weight, birth length or head circumference at birth and risk of developing respiratory allergy or atopic eczema at 13 years of age.

**Conclusion:** Higher gestational age was a risk factor for respiratory allergy at 13 years of age for children born at term.

### PD18 Evolution of the IgE response to house dust mite molecules in childhood

#### Daniela Posa^1^, Serena Perna^1^, Carl-Peter Bauer^2^, Ute Hoffmann^2^, Johannes Forster^3^, Fred Zepp^4^, Antje Schuster^5^, Ulrich Wahn^1^, Thomas Keil^6,7^, Susanne Lau^1^, Kuan-Wei Chen^8^, Yvonne Resch^8^, Susanne Vrtala^8^, Rudolf Valenta^8^, Paolo Maria Matricardi^1^

##### ^1^Department of Pediatric Pneumology and Immunology, Charité-Universitätsmedizin Berlin, Berlin, Germany; ^2^Department of Pediatrics, Technical University of Munich, Munich, Germany; ^3^Department of Pediatrics St. Hedwig, St. Josef’s Hospital, Freiburg, Germany; ^4^Department of Pediatrics and Adolescent Medicine, Johannes Gutenberg University Medical Centre, Mainz, Germany; ^5^Department of Pediatrics, Heinrich-Heine-University, Düsseldorf, Germany; ^6^Institute for Social Medicine, Epidemiology and Health Economics, Charité-Universitätsmedizin Berlin, Berlin, Germany; ^7^Institute of Clinical Epidemiology and Biometry, University of Würzburg, Würzburg, Germany; ^8^Division of Immunopathology, Department of Pathophysiology and Allergy Research, Center of Pathophysiology, Infectiology and Immunology, Medical University of Vienna, Vienna, Austria

###### **Correspondence:** Daniela Posa

*Clinical and Translational Allergy* 2016, **6(Suppl 1)**:PD18

**Background:** The allergic IgE response to grass pollen starts as a weak monosensitisation or oligosensitisation phenomenon and increase through a “molecular spreading” process. We aimed this study to investigate the development of the IgE response to house dust mite.

**Materials and methods:** The Multicenter Allergy Study (MAS), a birth cohort study, started in 1990 and recruiting 1314 infants in 5 German cities. Blood samples were collected at 1, 2, 3, 5, 6, 7, 10, 13 and 20 years of age. Sera with IgE antibodies to an extract of *Dermatophagoides pteronyssinus* (D.pt) (ImmunoCAP, TFS) were further tested for the presence of IgE to Der p1, Der p2, Der p4, Der p5, Der p7, Der p11, Der p14, Der p15, Der p18, Der p21, Der p23, Clone 16 (in the context of a MeDALL chip). The concentration of Der p1 was measured in house dust samples collected at 6–18 months of age.

**Results:** Overall, 168 subjects produced serum-IgE to D.pt. and its molecules. Der p2, Der p1 and Der p23 were the molecules most frequently recognized by IgE, working as “initiators molecules” in 98 % of the children. IgE to Der p4, 5, 7, 11, and 21 were frequently observed (20–37 %) while those to Der p 14, 15, 18 and to clone 16 were less frequent (11–18 %). This ranking of prevalence was stable during the first two decades of life. The IgE sensitization profiles to the 12 molecules were extremely heterogeneous in the population at all ages. Twenty-one subjects remained prospectively sensitized to only one molecule (stable”monomolecular” sensitization). The remaining 147 subjects developed a molecularly complex sensitization. The complexity of the IgE response to D.pt molecules was higher in the children most exposed to Der p1 at 6–18 months of age than in the least exposed ones (highest vs. lowest exposure quartile).

**Conclusions:** In the MAS birth cohort, Der p1, Der p2 and Der p23 are the “*initiators*” of the IgE response to D. pt. High exposure to Der p1 in early childhood is associated with a stronger “*molecular spreading”* of the IgE response to D.pt.

### PD19 Antibody recognition of nsLTP-molecules as antigens but not as allergens in the German-MAS birth cohort

#### Olympia Tsilochristou^1^, Alexander Rohrbach^1^, Antonio Cappella^2^, Stephanie Hofmaier^1^, Laura Hatzler^1^, Carl-Peter Bauer^3^, Ute Hoffmann^3^, Johannes Forster^4^, Fred Zepp^5^, Antje Schuster^6^, Raffaele D’Amelio^2^, Ulrich Wahn^1^, Thomas Keil^7,8^, Susanne Lau^1^, Paolo Maria Matricardi^1^

##### ^1^Department of Pediatric Pneumology and Immunology, Charité-Universitätsmedizin Berlin, Berlin, Germany; ^2^Department of Clinical and Molecular Medicine, Sapienza University of Rome, S. Andrea University Hospital, Rome, Italy; ^3^Department of Pediatrics, Technical University of Munich, Munich, Germany; ^4^Department of Pediatrics St. Hedwig, St. Josef’s Hospital, Freiburg, Germany; ^5^Department of Pediatrics and Adolescent Medicine, University Medicine Mainz, Germany; ^6^Department of Pediatrics, University of Düsseldorf, Düsseldorf, Germany; ^7^Institute of Social Medicine, Epidemiology and Health Economics, Charité-Universitätsmedizin Berlin, Berlin, Germany; ^8^Institute of Clinical Epidemiology and Biometry, University of Würzburg, Würzburg, Germany

###### **Correspondence:** Olympia Tsilochristou

*Clinical and Translational Allergy* 2016, **6(Suppl 1)**:PD19

**Background:** IgE sensitization to non-specific lipid transfer proteins (nsLTP) molecules is very frequent in Southern Europe, but not in Northern and Central Europe. Information on the geographical distribution of IgG responses to nsLTP in Europe is limited.

**Aim:** To evaluate the longitudinal development of IgE and IgG responses to nsLTP molecules in a population of German children.

**Methods:** Children of the German Multicenter Allergy Study (MAS) were included in the present analysis if they had provided: (1) ≥1 serum sample at age 1–3 years, (2) ≥2 serum samples at ages 5–7 years, (3) a serum sample at age 10 years. IgG (cut-off ≥0.1 ISU/L) and IgE (cut-off ≥0.3 ISU/L) to four nsLTP molecules (rPar j2, nArt v3, nPru p3, rCor a8) were tested by microarray (ISAC, TFS). Sera with undetectable IgE (<0.35 kU/l) against a panel of nine common foodborne and airborne allergenic extracts (ImmunoCAP, TFS) were considered negative for IgE against the four nsLTPs and were tested only for IgG.

**Results:** Overall, 586 sera from 104 children were examined. Of these, 389 (66 %) were negative for IgE to extracts and not tested for IgE to nsLTP. Only 1/104 (1 %) child had IgE to a nsLTP (only once, to nArt v3). By contrast, all 104 children but one (99 %) developed IgG to at least one of the examined nsLTPs. IgG responses to all the nsLTPs were frequent: nPru p3 = 72 % (419/586 sera), rCor a8 = 67 %, nPar j2 = 63 %, rArt v3 = 16 %. IgG to rCor a8 showed the highest concentration [geometric mean value: 1.62 (ISU/L)], followed by nPru p3 [1.40 (ISU/L)], nArt v3 [0.92 (ISU/L)], and rPar j2 [0.87 (ISU/L)]. The prevalence of IgG responses to rPar j2, nPru p3, and rCor a8 rapidly increased until age 5 and plateaued thereafter. The average concentration of IgG antibodies against all four nsLTP molecules remained quite stable at population level until age 10 years.

**Conclusions:** Our results confirm that IgE responses to nsLTPs are rare among children living in Central-Northern Europe. On the contrary, IgG responses to nsLTPs are very frequent in our German-MAS birth cohort. The biological and clinical implications of our observations require further investigation.

### PD20 Early life colonization with Lactobacilli and Staphylococcus aureus oppositely associates with the maturation and activation of FOXP3+ CD4 T-cells

#### Sophia Björkander, Maria A. Johansson, Gintare Lasaviciute, Eva Sverremark-Ekström

##### Department of Molecular Biosciences, The Wenner-Gren Institute, Stockholm University, Sweden

###### **Correspondence:** Sophia Björkander

*Clinical and Translational Allergy* 2016, **6(Suppl 1)**:PD20

**Introduction:** The human gut microbiota influences immune maturation during early life and associates with the development of immune mediated diseases. Several studies support the idea that the gut microbiota influences the FOXP3+ T-regulatory cells, which are important for immune homeostasis and tolerance. Lactobacilli are present in the early infant gut and correlated with a lower risk of allergy later in life. In addition, lactobacilli dampen in vitro immune activation induced by *Staphylococcus (S.) aureus*, a gut bacteria associated with an increased risk for allergy.

**Aims:** Here we investigated if early life colonization with lactobacilli and *S. aureus* influences the maturation and functional responses of FOXP3+ T-cells later in life. Further, we studied how soluble products from these bacteria affect FOXP3+ T-cells in vitro.

**Materials and methods:** RT PCR was used to detect and quantify bacterial DNA in faeces from infants and associated with immune data at age two. Peripheral blood mononuclear cells from children at age two and adults were analysed basally or after stimulation with cell free supernatants (CFS) from *Lactobacillus (L.) reuteri* DSM 17938 or/and *S. aureus* 161:2. The cells were stained with antibodies and analysed by flow cytometry.

**Results:** Children colonized with *S. aureus* had a higher percentage of FOXP3+ cells that produced IL-10 and expressed CD161, a T-cell marker connected to high cytokine-producing capacity. Further, in vitro stimulation with *S. aureus*-CFS induced CD161-expression and production of IL-10 and IFN-γ in FOXP3+ cells. In opposite, lactobacilli-colonization associated with a lower percentage of IL-10-producing FOXP3+ cells after stimulation. *L. reuteri*-CFS also dampened *S. aureus*-induced activation of FOXP3+ cells in vitro.

**Conclusions:** We conclude that species in the early gut microbiota are differentially linked to the development and function of FOXP3+ cells later in life, perhaps by modulation of CD161-expression and cytokine responses.

### PD21 Genome-wide meta-analysis identifies 7 susceptibility loci involved in the atopic march

#### Ingo Marenholz^1,2^, Jorge Esparza-Gordillo^1,2^, Franz Rüschendorf^1^, Anja Bauerfeind^1^, David P. Strachan^3^, Ben D. Spycher^4^, Hansjörg Baurecht^5^, Patricia Margaritte-Jeannin^6,7^, Annika Sääf^8^, Marjan Kerkhof^9^, Markus Ege^10^, Svetlana Baltic^11^, Melanie C. Matheson^12^, Jin Li^13^, Sven Michel^14^, Wei Q. Ang^11^, Wendy McArdle^15^, Andreas Arnold^16^, Georg Homuth^17^, Florence Demenais^6,7^, Emmanuelle Bouzigon^6,7^, Cilla Söderhäll^8^, Göran Pershagen^8^, Johan C. de Jongste^18^, Dirkje S. Postma^9^, Charlotte Braun-Fahrländer^19^, Elisabeth Horak^20^, Ludmila M. Ogorodova^21^, Valery P. Puzyrev^21,22^, Elena Yu Bragina^22^, Thomas J. Hudson^23^, Charles Morin^24^, David L. Duffy^25^, Guy B. Marks^26^, Colin F. Robertson^27^, Grant W. Montgomery^25^, Bill Musk^28^, Philip J. Thompson^11^, Nicholas G. Martin^25^, Alan James^28^, Patrick Sleiman^13,29^, Elina Toskala^30^, Elke Rodriguez^5^, Regina Fölster-Holst^5^, Andre Franke^31^, Wolfgang Lieb^31^, Christian Gieger^32^, Andrea Heinzmann^33^, Ernst Rietschel^34^, Thomas Keil^2,35^, Sven Cichon^36,37,38^, Markus M Nöthen^36^, Craig E. Pennell^11^, Peter D. Sly^39^, Carsten O. Schmidt^16^, Anja Matanovic^1,2^, Valentin Schneider^1^, Matthias Heinig^1,40^, Norbert Hübner^1^, Patrick G. Holt^11,39^, Susanne Lau^2^, Michael Kabesch^14^, Stefan Weidinger^5^, Hakon Hakonarson^13,29^, Manuel A. R. Ferreira^25^, Catherine Laprise^41^, Maxim B. Freidin^22^, Jon Genuneit^42^, Gerard H Koppelman^9^, Erik Melén^8,43^, Marie-Hélène Dizier^6,7^, A. John Henderson^15^, Young Ae Lee^1,2^

##### ^1^Max-Delbrück-Center for Molecular Medicine, Berlin, Germany, ^2^Charité University Medical Center, Berlin, Germany; ^3^ St George’s, University of London, UK; ^4^ University of Bern, Switzerland; ^5^ University Hospital Schleswig–Holstein, Kiel, Germany; ^6^ Inserm, Paris, France; ^7^ Université Paris Diderot, France; ^8^ Karolinska Institutet, Stockholm, Sweden; ^9^ University of Groningen, The Netherlands; ^10^ Ludwig Maximilians University, Munich, Germany; ^11^ University of Western Australia, Perth, Australia; ^12^ University of Melbourne, Australia; ^13^ The Children’s Hospital of Philadelphia, USA; ^14^ University Children’s Hospital Regensburg, Germany; ^15^ University of Bristol, UK; ^16^University Medicine Greifswald, Germany; ^17^ University Medicine and Ernst-Moritz-Arndt-University Greifswald, Germany; ^18^ Erasmus University Medical Center, Rotterdam, The Netherlands; ^19^ Swiss Tropical and Public Health Institute and the University of Basel, Switzerland; ^20^ Medical University, Innsbruck, Austria; ^21^ Siberian State Medical University, Tomsk, Russia; ^22^ Research Institute of Medical Genetics, Tomsk, Russia; ^23^ Ontario Institute for Cancer Research, Toronto, Canada; ^24^ Centre de santé et de services sociaux de Chicoutimi, Saguenay, Canada; ^25^ QIMR Berghofer Medical Research Institute, Brisbane, Australia; ^26^ University of Sydney, Australia; ^27^ Murdoch Children’s Research Institute, Melbourne, Australia; ^28^ Sir Charles Gairdner Hospital, Perth, Australia; ^29^ University of Pennsylvania, Philadelphia, USA; ^30^ Temple University, Philadelphia, USA; ^31^ Christian-Albrechts-University, Kiel, Germany; ^32^ Helmholtz Zentrum München, Germany; ^33^ Albert Ludwigs University, Freiburg, Germany; ^34^ University of Cologne, Germany^35^ University of Würzburg, Germany; ^36^ University of Bonn, Germany; ^37^ University of Basel, Switzerland; ^38^ Research Centre Jülich, Germany; ^39^ University of Queensland, Brisbane, Australia; ^40^ Max Planck Institute for Molecular Genetics, Berlin, Germany; ^41^ Université du Québec à Chicoutimi, Saguenay, Canada; ^42^ Ulm University, Germany; ^43^ Sachs’ Children’s Hospital, Stockholm, Sweden

###### **Correspondence:** Ingo Marenholz

*Clinical and Translational Allergy* 2016, **6(Suppl 1)**:PD21

**Introduction:** The atopic march refers to the sequential development of allergic conditions in childhood and is associated with severe and persistent disease manifestations. Up to 30 % of infants with eczema develop asthma in childhood which is the most common pattern of the atopic march.

**Aim of the study:** We conducted a multi-stage genome-wide association study (GWAS) for infantile eczema followed by childhood asthma to unravel the genes underlying this characteristic pattern of allergic disease.

**Methods:** GWASs were performed in 6 study populations (discovery phase). Selected SNPs were replicated in another 6 study populations (replication phase). Our study included 2428 cases and 17,034 controls of European descent. Association was calculated by logistic regression using an additive allele-dosage model. Meta-analyses were carried out with METAL using the inverse variance fixed effects model.

**Results:** We identified 7 loci associated with the atopic march at genome-wide significance. Two chromosomal loci at 6p12.3 and 12q21.3 were specific for the combined eczema plus asthma phenotype and associated with allergic disease for the first time. Four additional loci at 1q21.3, 5q31.1, 11q13.1, and 11q13.5 were previously identified in GWASs on eczema while, at 17q21, a single asthma-specific locus was detected. By inspecting all known GWAS loci for eczema or asthma in the discovery set, we found that eczema loci were significantly more likely to be associated with the atopic march than asthma loci.

**Conclusion:** The two novel loci provide genetic support for a specific atopic march phenotype. In addition, we demonstrate that eczema loci were the main genetic determinants of the atopic march which may point to the development of eczema as a key event initiating this unfavorable disease course. We suggest that the prevention or early treatment of infantile eczema could be a promising approach in order to reduce the burden of allergic diseases associated with the atopic march.

## POSTER DISCUSSION SESSION 4: Food allergy—Anaphylaxis (PD22–PD26)

### PD22 Atopy patch test in food protein induced enterocolitis caused by solid food

#### Purificacion González-Delgado^1^, Esther Caparrós^2^, Fernando Clemente^3^, Begoña Cueva^1^, Victoria M. Moreno^2^, Jose Luis Carretero^4^, Javier Fernández^1^

##### ^1^Allergy Section, Hospital General Universitario Alicante, Alicante, Spain; ^2^Universidad Miguel Hernández, Alicante, Spain; ^3^Pediatrics Service, Hospital General Universitario Alicante, Alicante, Spain; ^4^Preventive Service, Hospital General Universitario Alicante, Alicante, Spain

###### **Correspondence:** Purificacion González-Delgado

*Clinical and Translational Allergy* 2016, **6(Suppl 1)**:PD22

**Background:** Atopy patch test (APT) has been proposed as a valuable tool in children with non-IgE mediated cow’s milk allergy, although studies are small and limited. We aimed to investigate the value of APT in solid food protein induced enterocolitis (FPIES).

**Methods:** We studied 22 children diagnosed as having FPIES caused by solid foods. Oral food challenge was performed to confirm the diagnosis, except in severe recent reactions. All children had negative skin prick test (SPT) to the offending food.

Patch tests were performed to a battery of foods implicated with the same extracts used for SPT (ALK, Abello, Denmark). They were applied into Finn chambers, placed on the back and removed after 48 h. Reactions were scored at 72 h. Positive reactions included erythema with infiltration (+), with few papules (++) several papules (+++) and vesicles (++++) following the EAACI GA^2^LEN position paper.

APT were carried out in the first and in follow up visits.

A control group of 10 non atopic children with good tolerance to foods were also tested.

**Results:** 22 patients with FPIES to solid foods were studied. Mean age at diagnosis was 19 months (range 12–28 months). Eighteen patients were diagnosed by OFC, 4 with clinical history and OFC was not performed because of a recent severe reaction.

Skin prick tests were negative in all.

At the first visit, 8 children showed positivity APT to the offending food, 14 were negative (sensitivity: 0.36, 36 %, specificity: 100 %). Children in control group showed negative APT.

In the series of positive APT children, only 4 showed persistence of positivity when they were tested 24 months later. Patients in whom APT became negative (4), underwent OFC that confirmed persistence of symptoms.

**Conclusions:** APT in FPIES induced by solid foods has some diagnostic value, but OFC remains as the gold standard.

Although in some patients APT become negative, clinical symptoms persisted, so the age of diagnosis may be important, with loss of sensitivity in older children.

### PD23 Watermelon allergy: a novel presentation

#### Kate Swan, George Du Toit

##### Pediatric Allergy Department, St Thomas’ Hospital, London, UK

###### **Correspondence:** Kate Swan

*Clinical and Translational Allergy* 2016, **6(Suppl 1)**:PD23

**Background:** Recently, there has been an increased recognition of patients who are allergic to the seeds but not the pulp of citrus fruit. A study of 100 patients with nut allergy demonstrated high rates of co-sensitisation between cashew and orange seed but the clinical relevance of this state was not investigated by challenge. Watermelon (*Citrullus lanatus*), of the Cucurbitaceae family, has a high protein content in the seeds. Watermelon allergy predominantly presents as oral allergy syndrome but we describe a different manifestation.

**Case history:** 2 patients have presented to our pediatric allergy service, both with a history of uneventfully enjoying consumption of watermelon on multiple occasions. A 6 years old boy, on one occasion of eating watermelon had swelling of his lips and widespread urticaria which resolved with anti-histamine and another young man of 7 years had anaphylaxis requiring an adrenaline autoinjector when he ate watermelon.

**Investigations:** Both tested negative to the watermelon pulp (0 mm) but had positive skin prick test to the seeds (4 mm and 25 mm). Interestingly, both were also sensitised to cashew nut (3 mm and 6 mm). Neither has ever eaten cashew nut.

**Discussion:** Watermelon seeds are often swallowed whole or removed before eating, hence patients may appear to tolerate watermelon. When the seed contents are consumed the reaction occurs. Watermelon seeds contain high levels of vicilin-like glycoprotein (seed storage protein). In cashew nuts Ana o 1, a vicilin-like protein, is a major food allergen hence the possibility of cross-reactivity. Other vicilin-like proteins such as peanut (Ara h1), soya (gly m5) and sesame (Ses i3) may also need consideration.

**Conclusions:** Allergy to fruit seeds may be common and under-recognised. One should consider seed allergy despite previous or ongoing apparent tolerance to the fruit pulp. Clinical significance of the co-sensitisation to the cashew nut is uncertain and needs further investigation.

### PD24 A pilot study evaluating the usefulness of a guideline template for managing milk allergy in primary care

#### Mudiyur Gopi, Tim Smith, Edara Ramesh, Arun Sadasivam

##### Macclesfield District General Hospital, NHS England, Altrincham, UK

###### **Correspondence:** Mudiyur Gopi

*Clinical and Translational Allergy* 2016, **6(Suppl 1)**:PD24

**Background:** With the increasing prevalence of allergic disease, the number of children presenting with cow’s milk protein allergy (CMPA) to primary care has also increased. They have little resource or access to allergy testing and may be unfamiliar with the interpretation of results. Many guidelines have surfaced in the past few years but it is felt that the knowledge, uptake and application of these guidelines have been suboptimal. This project attempted to assess if an electronic version of a modified CMPA guideline can increase the confidence of primary care clinicians in the management of children with possible CMPA. This was done by developing a template which was adapted and modified from a national guideline and was introduced into individual primary care computer systems.

**Aim:** To evaluate the usefulness of a guideline template for “managing cow’s milk allergy in primary care” based on confidence scoring from primary care clinicians.

**Methodology:** An electronic guideline template for managing CMPA was introduced for a period of two months in primary care electronic record keeping computer system. Confidence scoring was compared in GPs before and after this period to see whether use of the template resulted in changes in confidence scores.

**Results:** Nine clinicians participated in this study from five surgeries across North of England. Awareness of national guidelines was very low in this group (11 %) before the template was introduced. Following the introduction of an electronic template to manage CMAP, a significant increase in confidence scores was noted from all participants GPs (p = 0.038). When asked if the template improved their confidence levels, a significant number of respondents answered in the affirmative (p = 0.020). A significant number of respondents felt that using the templates helped them consider the diagnosis if CMPA in cases where such a diagnosis would not have been considered previously (p = 0.003). An additional finding was noted in this survey that the GPs did not refer the patients to dieticians (p = 0.007), which is a MAP guideline recommendation.

**Conclusion:** Our study, like many previous studies, has demonstrated that the uptake and use of national guidelines remains poor. Our study demonstrates that confidence levels in managing CMPA improved significantly by following an electronic template. We recommend that national and international bodies take note of the significance of these findings as these may guide future CMPA and other guideline implementation in primary care.

### PD26 Efficacy and safety of cow’s milk oral immunotherapy protocol

#### Inês Mota, Filipe Benito Garcia, Susana Piedade, Angela Gaspar, Graça Sampaio, Cristina Arêde, Luís Miguel Borrego, Graça Pires, Cristina Santa-Marta, Mário Morais-Almeida

##### Immunoallergy Department, CUF Descobertas Hospital, Lisbon, Portugal

###### **Correspondence:** Inês Mota

*Clinical and Translational Allergy* 2016, **6(Suppl 1)**:PD26

**Background:** Cow’s milk (CM) is one of the major causes of food allergy in childhood. Oral immunotherapy (OIT), a novel strategy, has been recognized as promising treatment for severe and long-lasting CM allergy. The authors describe the efficacy and safety of the CM-OIT protocol used in our Immunoallergy Department.

**Methods:** Systematic review of all children and adolescents, who underwent CM-OIT until March 2015. The protocol involves the introduction of increasing amounts of non-diluted CM, beginning with sublingual drops and gradual increases of the threshold dose at predetermined intervals. The doses increments and establishment of the dose for daily ingestion at home were always performed in hospital sessions.

**Results:** A consecutive sample of 49 children and adolescents was included: mean age at initiation of the protocol was 7 years-old, 59 % male and 96 % IgE-mediated reactions (20 % anaphylaxis). Most patients had other allergic diseases (69 % allergic rhinitis, 51 % asthma, 33 % atopic dermatitis) and 24 % multiple food allergy. The target dose (200 mL/day) was reached in 92 % of children, after a mean period of time of 5.2 months (ranging from 1.5 to 15 months). There were 4 failures: 2 due to gastrointestinal symptoms and 2 by poor adherence. During the protocol occurred mild to moderate reactions in 86 % and severe in 3 cases: milk-dependent exercise-induced anaphylaxis, accidental exposure and due to poor adherence to the recommended protocol. All reactions were controlled with rescue treatment.

**Conclusions:** CM-OIT is a safe and effective treatment for severe and long-lasting CM allergy. The protocol used allowed to achieve tolerance within a short period of time. Maintenance of 200 mL daily ingestion enables a diet without restrictions, with a clear positive impact on quality of life.

## POSTER DISCUSSION SESSION 5: Prevention and treatment—Allergy (PD27–PD36)

### PD27 Allergy-protection by the lactic acid bacterium *Lactococcus lactis* G121: mode-of-action as revealed in a murine model of experimental allergy

#### Stephanie Brand^1^, Karina Stein^2^, Holger Heine^2^, Marion Kauth^1^

##### ^1^Protectimmun GmbH, Gelsenkirchen, Germany; ^2^Division of Innate Immunity, Research Center Borstel, Borstel, Airway Research Center North, Member of the German Center for Lung Research (DZL), Germany

###### **Correspondence:** Marion Kauth

*Clinical and Translational Allergy* 2016, **6(Suppl 1)**:PD27

**Introduction:** Numerous epidemiological studies provide strong evidence that frequent contact to a traditional farm environment in early life protects children from the development of allergic airway diseases like hay fever and asthma. We have previously demonstrated that intranasal application of the cowshed-derived lactic acid bacterium *Lactococcus lactis* G121 (LL) resulted in protection from allergic disease in different murine asthma models. However, details of the underlying mode-of-action of this protective immune response are largely unknown.

**Materials and methods:** Cellular mechanisms involved in cytokine induction upon LL exposure were analysed in human monocyte derived dendritic cells (moDCs) and murine bone marrow-derived dendritic cells (BMDCs) by use of specific inhibitors for uptake and endosomal acidification. Functional relevance was assessed in vivo in a murine model of allergic airway inflammation with sensitisation via intranasal transfer of ovalbumin (OVA)-pulsed BMDCs to naïve mice.

**Results:** LL exposure of moDCs and BMDCs led to release of several cytokines including IL-12p70 and IL-10. Preincubation with the inhibitors Cytochalasin D or Bafilomycin A1 (Baf) strongly decreased the cytokine production indicating the importance of both uptake of the bacteria and endosomal acidification. *In vivo*, sensitisation of mice could be achieved by intranasal administration of OVA-pulsed BMDC. Administration of BMDC which were exposed to LL prior to pulsing with allergen (G121/OVA group) protected mice from the development of allergic airway inflammation upon allergen challenge. However, treatment of BMDC with Baf prior to LL exposure significantly reduced the protective effect resulting in a restored allergic phenotype.

**Conclusion:** We revealed uptake of LL in dendritic cells and subsequent endosomal acidification as key elements of the mode-of-action of allergy protection mediated by the cowshed-derived bacterium. These findings will greatly support the further development of a *Lactococcus*-based primary prophylaxis against hay fever and asthma for protection of children early in life.

**Funding:** KS and HH were supported by DFG, SFB/TR22, project A2.

### PD29 The relationship between quality of life and morning salivary cortisol after acute bronchiolitis in infancy

#### Leif Bjarte Rolfsjord^1^, Egil Bakkeheim^2^, Johan Alm^3^, Håvard Ove Skjerven^4^, Kai-Håkon Carlsen ^2^, Jon Olav Hunderi^2^, Teresa Løvold Berents^2^, Petter Mowinckel^2^, Karin C. Lødrup Carlsen^2^

##### ^1^Innlandet Hospital Trust, Elverum, Norway; ^2^Oslo University Hospital, Oslo, Norway; ^3^Karolinska Institutet, Stockholm, Sweden; ^4^Institute of Clinical Medicine, University of Oslo, Oslo, Norway

###### **Correspondence:** Leif Bjarte Rolfsjord

*Clinical and Translational Allergy* 2016, **6(Suppl 1)**:PD29

**Introduction:** Hospitalisation with bronchiolitis in infancy is associated with increased asthma risk and reduced quality of life (QoL). Stress may negatively impact morning cortisol and possibly asthma development. We aimed to investigate the association between cortisol and QoL in young children and the potential modifying effect of having been hospitalised for bronchiolitis.

**Method:** In children recruited during hospitalisation in their first year of life for moderate to severe bronchiolitis (n = 207), and controls (n = 152), with mean ages at follow-up 23.5 and 24.0 months, morning salivary cortisol and parentally completed QoL questionnaires, ITQOL™, were collected. 10.5 % of controls and 32.4 % of bronchiolitis children had asthma diagnosis or inhaled steroids daily. Cortisol was analysed at Karolinska Institutet by RIA. By robust regression, cortisol results were analysed as dependent of QoL quartiles, adjusting for age and gender, and subsequently also for asthma severity, graded depending of follow-up plans or daily steroid inhalations.

**Results:** Cortisol did not differ significantly between the groups, but was lower the more severe the asthma. Mean QoL scores were higher in the control group for Overall health and General Health, but higher in the bronchiolitis group for Change in health (compared to 1 year ago). Increasing QoL for 11 of 13 domains was associated with increasing morning cortisol in the bronchiolitis group but not in controls except for two domains. Controls had results for one domain pointing the opposite way. After adjustment for asthma severity, the associations remained significant for 9 of 13 domains in the bronchiolitis group. We found interaction between hospitalisation for bronchiolitis and QoL quartiles.

**Conclusion:** Increasing QoL at 2 years of age was associated with increasing morning cortisol only in the bronchiolitis group. The group differences are possibly due to different asthma frequencies and severities, but not explainable only by our measured asthma severity.

### PD30 Randomised trial of the efficacy of MP29-02* compared with fluticasone propionate nasal spray in children aged ≥6 to <12 years with allergic rhinitis

#### Ulrich Wahn^1^, Ullrich Munzel^2^, William Berger^3^

##### ^1^Department of Pediatrics, Division of Pneumonology, Immunology and Intensive Care Medicine incl. Rescue Center, Charité University Hospital, Berlin, Germany; ^2^Meda, Bad Homburg, Germany; ^3^Allergy and Asthma Associates of Southern California, Mission Viejo, CA, USA

###### **Correspondence:** Ulrich Wahn

*Clinical and Translational Allergy* 2016, **6(Suppl 1)**:PD30

**Background:** MP29-02* (a novel intranasal formulation of azelastine hydrochloride (AZE) and fluticasone propionate (FP)) is approved for use in patients aged 12 years or older with moderate/severe allergic rhinitis (AR). Superior efficacy of MP29-02* over FP has already been established in seasonal AR patients (aged ≥12 years) [1,2] and in perennial AR patients (aged 12–80 years) [3]. The objective of this analysis was to evaluate the efficacy of MP29-02* compared to FP, administered as 1 spray per nostril twice daily, in pediatric AR subjects aged ≥6 to <12 years.

**Methods:** This was a randomized, open-label, 3-month safety study in patients (≥4 to 12 years). Qualified subjects had a history of AR, were in good health, and had no evidence of nasal mucosal erosion, nasal ulceration, nasal septum perforation, or any significant nasal disease. Subjects were randomized in a 3:1 ratio to MP29-02* (n = 304) or FP (n = 101). Efficacy was also assessed by subject-reported assessment of overall allergy symptom severity, in a subset of patients (aged 6–12 years; MP29-02*: n = 264; FP: n = 89). Symptom severity was rated daily on a 4 point scale from 0 to 3 (0 = no symptoms; 1 = mild symptoms; 2 = moderate symptoms; 3 = severe symptoms).

**Results:** The total symptom score at baseline was 1.73 for patients in the MP29-02* group and 1.80 for patients in the FP group (max score: 3). Over the entire study period patients treated with MP29-02* experienced a −0.68 pt reduction in overall symptom score (corresponding to a −5.44 change from baseline in AM + PM reflective total nasal symptom score (rTNSS; max = 24), significantly greater relief than that afforded by FP (−0.54 pt reduction; Diff: −0.14; 95 % CI: −0.28, −0.01; p = 0.04).

**Conclusion:** MP29-02* provides significantly greater AR symptom relief than FP in a pediatric population (aged ≥6–12 years) and has been granted approval for use in this age group by the FDA.

*Dymista

**References**

1. Meltzer E, et al. Int Arch Allergy Immunol. 2013;161(4):369–77
2. Carr W, et al. J Allergy Clin Immunol. 2012;129(5):1282–9
3. Price D, et al. J Investig Allergol Clin Immunol. 2013;23(7):495–503

### PD31 10 mg of oral bilastine in 2 to 11 years old children has similar exposure to the adult therapeutic dose (20 mg)

#### Ulrich Wahn^1^, Román Valiente^2^, Valvanera Vozmediano^3^, John C. Lukas^3^, Mónica Rodríguez^3^

##### ^1^Department of Pediatric Pneumology and Immunology, Charité-Universitätsmedizin Berlin, Berlin, Germany; ^2^Clinical Research Department, FAES FARMA SA, Leioa, Bizkaia, Spain; ^3^Drug Modeling and Consulting, Dynakin SL, Bilbao, Spain

###### **Correspondence:** Mónica Rodríguez

*Clinical and Translational Allergy* 2016, **6(Suppl 1)**:PD31

**Background:** The H_1_ antihistamine Bilastine is approved in adults for seasonal and perennial allergic rhinoconjunctivitis (SAR/PAR) and urticaria. A model based drug development strategy, supported by a set of prior data from adults (N = 310) receiving bilastine, permitted extrapolation of the PK to children, selection of the appropriate dose for the pediatric subset, and then design of an adaptive limited sampling trial to confirm the adequacy of the proposed regimen.

**Objective:** A clinical trial was performed in children (2–11 years) with SAR/PAR or urticaria to ascertain whether the proposed dose (10 mg/day) matched the systemic exposure in adults (20 mg/day), via pharmacokinetic (PK) assessment.

**Methods:** Blood samples were drawn from each child after multiple administrations of 10 mg of bilastine as oral dispersible tablet. The PK schedule was optimally designed to minimize the number of samples and the total volume per child. Dataset (N = 31) analysis was with NONMEM^®^ VI. Final model establishment and validation was done using standard statistical and diagnostic criteria for parametric non-linear mixed effects models. Individual PK parameters were used to calculate the exposure (AUC_0→∞_), which was then applied in the statistical assessment of the suitability of the proposed dose.

**Results:** The population parameters in children, expressed as mean (SEE%) were the following: K_a_ 1.29 (22.2) h^−1^, CL/F 12.5 (5.90) L/h, Vc/F 19.7 (12.8) L, Q/F 2.01 (34.0) L/h, Vp/F 17.4 (14.8) L. Interindividual variability was estimated for CL and K_a_15 % and 67 %, respectively and also a proportional error variance (35 (22) %). The model served to characterize the PK in children and to confirm the universal validity of the proposed 10 mg dose for the whole pediatric range.

**Conclusion:** A daily dose of 10 mg bilastine in children adequately produces similar exposure (1265 ng h/mL) to that observed in adults (1105 ng h/mL) after the therapeutic dose of 20 mg.

### PD33 Daily symptoms, nocturnal symptoms, activity limitations and reliever therapies during the three steps of IOEASMA programme: a comparison

#### Sebastiano Guarnaccia, Luigi Vitale, Ada Pluda, Emanuele D’Agata, Denise Colombo, Stefano Felici, Valeria Gretter, Susanna Facchetti, Gaia Pecorelli, Cristina Quecchia

##### Centro “Io e l’Asma”, Spedali Civili, Brescia, Italy

###### **Correspondence:** Sebastiano Guarnaccia

*Clinical and Translational Allergy* 2016, **6(Suppl 1)**:PD33

**Introduction:** Asthma is a major cause of chronic morbidity and mortality throughout the world in terms of access to emergency services, number of hospitalization days, school absence.

IOEASMA program used an integrative care model, implemented international asthma guidelines, to address pediatric asthma with a multidisciplinary approach.

**Methods:** Since 2007 the center “Io e l’Asma” implemented the programme as follows:

- 2007–2009, Path Diagnostic Therapeutic (PDT): 3 visits every 4/6 weeks, with follow-up after 6 months. During the protocol, skin prick tests and spirometry were administered.
  The family physician and/or the patient’s parents communicated to specialists any symptoms and treatments used.
- 2010–2011: Path Diagnostic Therapeutic Educational (PDTE): 3 visits every 8 weeks, followed by semiannual or annual visit. After the first visit, children and parents undertook an individual therapeutic education course, conducted by the health personnel, about: environmental prevention, asthma attacks management, daily therapy use, proper use of devices, self-management disease control.
- 2012–2013, Path Diagnostic Therapeutic Educational (PDTE) reissued as ECCM, extended to lifestyles above 6 years of age. The ECCM, after the first visit, addressed the following health adversity: social interaction, diet, physical activity, smoking.

**Results:** A percentage decrease (Δ%), in “activity limitations”: −37.5 % (PDT); −39.4 % (PDTE); −40.4 % (PTDE + ECCM). “Nocturnal symptoms”: −32.1 % (PDT); −33.8 % (PDTE); −34.3 % (PTDE + ECCM); reaching the highest value in the (PDTE + ECCM). A significant percentage decrease of “daily symptoms” in each group (Δ −37.5 %); −31.7 (PDTE); −23.7 % (PTDE + ECCM). The “relievers therapies” decreased in each group with a more significant percentage change in the first period (Δ −17.3 %) compared to PDTE (Δ −9 %) and to PDTE + ECCM (Δ −14 %); initial number of patients were really different in the three groups at the beginning, so the gap is explained.

**Conclusions:** An integrated and structured diagnostic and therapeutic pathway can significantly reduce the impact of asthma and its comorbidities and improve children’s quality of life.

### PD34 Sensitisation to an inert aeroallergen in weaning rats and longstanding disease, in a sensitisation-tolerant and easily tolerisable rodent strain

#### George Guibas^1,2^, Evangelia Spandou^3^, Spyridon Megremis^1^, Peter West^1^, Nikolaos Papadopoulos^1,2,4^

##### ^1^Center of Pediatrics and Child Health, Institute of Human Development, University of Manchester, Manchester, UK; ^2^Department of Pediatric Immunology, Royal Manchester Children’s Hospital, Central Manchester University Hospitals NHS Trust, Manchester, UK; ^3^Laboratory of Experimental Physiology, Aristotle University of Thessaloniki, Thessaloniki, Greece; ^4^Allergy Department, 2nd Pediatric Clinic, University of Athens, Athens, Greece

###### **Correspondence:** George Guibas

*Clinical and Translational Allergy* 2016, **6(Suppl 1)**:PD34

**Background:** The difficulty to sensitize most strains of laboratory rodents to ovalbumin (OVA) -and especially through the airways-, without the addition of adjuvant is well-known. It is also well-known that even if sensitization does occur, it is difficult to model longstanding disease, as the rodents gradually develop tolerance to OVA and usually only one or two exposures are carried out before sacrifice.

**Aim:** We opted to design a rodent model of allergic rhinitis/asthma in which we could both sensitize the rodents to the innocuous aeroallergen OVA through the airways, but also manage to sustain longstanding disease even after several exposures.

**Methods:** Three week-old ether-anesthetized male Wistar rats (n = 20) were given ovalbumin (OVA) in saline vehicle, intranasally (i.n). Fifteen days later, awake rats were re-challenged i.n with OVA (n = 10-active group) or saline (n = 10-control group) and subsequent sneezes were counted. The rodents were re-exposed every third day for a total of 13 exposures. They were sacrificed 54 days from the start of the experiment. Inflammation of the nasal mucosa was semi-quantitatively assessed with Haematoxylin & Eosin stain.

**Results:** OVA-challenged rats demonstrated signs of allergic rhinitis as evidenced by a statistically significant increase in the number of sneezes and presence of marked nasal mucosal inflammation, as opposed to the control group.

**Conclusions:** We present an experimental model whereby we managed to sensitize to OVA, rats of a sensitization-tolerant strain. These rats were of weaning age upon first exposure and sensitization. Furthermore, these weaning rats were sensitized via the airways without the addition of adjuvant, and multiple exposures to OVA failed to tolerise them to this innocuous aeroallergen. It is likely that exposure to inert aeroallergens at a very early age and in the context of in irritant (ether), rendered these rats susceptible to sensitization and longstanding disease.

### PD35 Bacterial and fungi exposure in school and allergic sensitisation in children

#### João Cavaleiro Rufo^1^, Joana Madureira^1^, Inês Paciência^1^, Lívia Aguiar^2–3^, Patrícia Padrão^5^, Mariana Pinto^4^, Luís Delgado^4^, Pedro Moreira^5^, João Paulo Teixeira ^2,3^, Eduardo Oliveira Fernandes^1^, André Moreira^4,5^

##### ^1^INEGI-Institute of Science and Innovation in Mechanical Engineering and Industrial Management, Porto, Portugal; ^2^National Institute of Health, Porto, Portugal; ^3^Epidemiology Research Unit, Institute of Public Health (EPIUnit), University of Porto, Porto, Portugal; ^4^Faculty of Medicine, University of Porto, Centro Hospitalar São João, Porto, Portugal; ^5^Faculty of Nutrition and Food Sciences, University of Porto, Porto, Portugal

###### **Correspondence:** João Cavaleiro Rufo

*Clinical and Translational Allergy* 2016, **6(Suppl 1)**:PD35

**Background:** Development of allergic sensitization may be regulated by microbial exposure. Children spend a lot of their time in school, under an extensive diversity of biological aerosols, such as bacteria and fungi. We aimed characterize indoor air microbiological exposure as a predictor of allergic sensitization.

**Methods:** A total of 858 children, aged 8–10 years, attending 71 classrooms in 20 primary schools were submitted to skin-prick tests (SPT) to house-dust mites, mixed weed, mixed grasses, cat, dog and *Alternaria alternata*. Atopy was defined by a positive SPT to at least one of the allergens. Air samples were collected in all the participating classrooms and respective outdoor locations using a single-stage microbiological air impactor through TSA and MEA plates at a 100 L/min rate. Quantification was performed by naked eye count. Endotoxins were collected using a 2 L/min flow control pump for 4 h and analysed by limulus amebocyte lysate assay. Mann–Whitney tests and logistic regression models were used to statistically analyse the data.

**Results:** Prevalence of atopy was 34.1 %. The risk of sensitization to inhalant allergens increased with increasing endotoxin exposure in classrooms (p = 0.015). Similarly, higher concentrations of *Penicillium* spp. showed higher risk of having a positive SPT (1.682 [95 % CI 1.180–2.398]) while children in classrooms with higher concentrations of *Aspergilus fumigatus*, *Aspergilus niger* and *Chaetomium* spp. had lower risk of sensitization (0.64 [95 % CI: 0.47–0.87], 0.62 [95 % CI: 0.45–0.87] and 0.61 [95 % CI: 0.39–0.96], respectively).

**Conclusion:** Although the cross sectional nature of our study does not allow to establish causal relationships, our results further suggest that current exposure to higher levels of endotoxin is associated with increasing odds of allergic sensitization in children. However, some fungi species, such as *Aspergilus fumigatus*, *Aspergilus niger* and *Chaetomium* spp., but not *Penicillium* spp., may also have effect in protecting from allergic sensitization.

### PD36 Comparative study of allergy rhinitis between two populations: children vs. adults

#### Adriana Izquierdo Dominguez^1,2^, Antonio Valero^1^, Joaquim Mullol^3^, Alfonso Del Cuvillo^4^, Javier Montoro^5^, Ignacio Jauregui^6^, Joan Bartra^1^, Ignacio Davila^7^, Marta Ferrer^8^, Joaquin Sastre^9^

##### ^1^Hospital Clínic de Barcelona, Barcelona, Spain; ^2^Hospital Quirón, Barcelona, Spain; ^3^Unitat de Rinologia i Clinica de l’Olfacte, Servei d’Otorinolaringologia, Hospital Clínic de Barcelona, Barcelona, Spain; ^4^Sección de Rinología, UGC ORL, Hospital de Jerez, Cadiz, Spain; ^5^Hospital Universitario Arnau de Vilanova, Valencia, Spain; ^6^Hospital de Basurto, Bilbao, Spain; ^7^Hospital Clínico de Salamanca, Salamanca, Spain; ^8^Clínica Universitaria de Navarra, Pamplona, Spain; ^9^Fundación Jimenez Díaz, Madrid, Spain

###### **Correspondence:** Adriana Izquierdo Dominguez

*Clinical and Translational Allergy* 2016, **6(Suppl 1)**:PD36

**Introduction:** Allergic rhinitis (AR) is a highly prevalent disease among the worldwide population, with significant impact on quality of life and healthcare costs that affects both pediatric and adults. Numerous studies describe the characteristics of AR in adult and pediatric patients, but do not compare the characteristics between both populations.

**Objectives:** The aim was to compare the characteristic of AR between child and adults.

**Methods:** Two observational, cross-sectional, multicenter studies were performed with data collection consecutively in two phases, the study in children and study in adults. The AR was classified according to the criteria original Allergic Rhinitis and Its Impact on Asthma (o-ARIA) and a modification of this classification (m-ARIA), and comorbidities were also assessed. We compared both databases, and we was performed a database in common, for analysis by SPSS 15.0

**Results:** 5405 patients (children: 1275; adults: 4130) were included, mean age 37.5 (±13.4) years in adults and 9.05 (±1.93) years children. Distribution by gender was 41 % girls and 52 % female. According to their duration, was intermittent in 59.5 % children and 51.5 % adults (p < 0.001). According o-ARIA classification adults was 26 % mild, 74 % moderate and severe. Children was 10.3 % mild, 90 % moderate-severe (p < 0.001). Depending on m-ARIA classification, AR adults was moderate 56.7 and 14.5 % severe. In children was moderate 68.3 and 18.2 % severe (p < 0.0001). Adults T4SS (6.50 ± 2.8) were higher than children (6.25 ± 2.8; p < 0.01). Moreover, adults VAS (39.78 ± 23.60) were higher than children (36.99 ± 25.47; p < 0.0001). According classification allergen implicated adults was perennial 35 % of them and, children 39 % (p < 0.001). Regarding comorbidities, 49.5 % children had asthma and 20 % adults; 54 % children had conjunctivitis and 28 % adults (p < 0.0001).

**Conclusion:** We found significant differences between the characteristics of AR between children and adults. Rhinitis is more intermittent and severe in children, being also more prevalent comorbidities.

## POSTER VIEWING SESSION 1: Inflammation—Genetics—Immunology—Dermatology (PP01–PP09)

### PP01 Immune profile in late pregnancy: immunological markers in atopic asthmaticwomen as risk factors for atopy in the progeny

####  Catarina Martins^1^, Jorge Lima^2^, Maria José Leandro^3^, Glória Nunes^1^, Jorge Cunha Branco^2^, Hélder Trindade^4^, Luis Miguel Borrego^1,2^

##### ^1^CEDOC, NOVA Medical School, UNL, Lisboa, Portugal; ^2^CUF Descobertas Hospital, Lisboa, Portugal; ^3^UniversityCollege London, Center for Rheumatology, London, United Kingdom; ^4^Instituto Português do Sangue e da Transplantação, Lisboa, Portugal

###### **Correspondence:** Luis Miguel Borrego

*Clinical and Translational Allergy* 2016, **6(Suppl 1)**:PP01

**Background:** Asthma and rhinitis are the commonest chronic diseases in pregnancy, with possible outgrowth in progeny.

**Objective:** This study aimed to characterize the immune profile of atopic asthmatic women in late pregnancy, and to further evaluate its relation with the development of atopic signs and symptoms in newborns.

**Methods:** Third trimester asthmatic pregnant women (n = 15) were recruited between the 31st and 36th weeks of gestation. At this time point, peripheral blood samples from each woman were collected and analyzed by Flow Cytometry, in order to characterize T and B cell subsets. Six months after birth, the babies were clinically assessed and atopic manifestations were investigated (atopic dermatitis, recurrent wheeze). Statistical analysis was done using GraphPadPrism6 (statistical significance: p-value <0.05).

**Results achieved:** After separating the mothers in two groups (BW-babies with complications, n = 6, and BWO-babies without complications, n = 9), differences were observed, with BW mothers presenting increased circulating activated CD4^+^CD25^+^ T cells and decreased effector memory CD8^+^ T cells (p < 0.05) in late pregnancy. Within B cells, BW mothers had a significantly higher percentage of transitional CD38^++^IgM^++^ B cells (p < 0.001).

**Conclusion:** BW mothers do have distinguishing immune features in late pregnancy, with a more activated T cell compartment and loss of circulating effector memory CD8^+^ T cells. Interestingly, effector memory CD8^+^ T cells, highly activated, are the most important subset of T cells at the fetal–maternal interface.[1] We speculate that BW mothers have a higher migration of effector memory CD8^+^ T cells into the fetal-maternal interface, and this activation status may alter the immune response of the child. Transitional B cells point towards a more effective B cell output in BW mothers. These are preliminary data that need further confirmation, but can be useful in the future development of atopy risk markers in children from atopic mothers.

**Reference**

1. Tilburgs T, Strominger JL. CD8^+^ effector T cells at the fetal–maternal interface, balancing fetal tolerance and antiviral immunity. Am J ReprodImmunol. 2013;69(4). doi:10.1111/aji.12094.

### PP02 The impact of neonatal sepsis on development of allergic diseases

####  Secil Conkar^1^, Mehtap Kilic^1^, Canan Aygun^2^, Recep Sancak^1^

##### ^1^Department of Pediatric Allergy, Mayıs Unıversity, Samsun, Turkey; ^2^Department of Neonatology, Mayıs Unıversity, Samsun, Turkey

###### **Correspondence:** Mehtap Kilic

*Clinical and Translational Allergy* 2016, **6(Suppl 1)**:PP02

**Background:** It was suggested that bacterial infections in children at early ages can protect allergic reactions in future. Nevertheless, contact to bacterial products such as endotoxins in early childhood may protect development of allergic diseases later in childhood is still a dilemma.

**Aim:** The aim of the study is to assess the impact of neonatal sepsis on development of asthma, allergic rhinitis and atopic dermatitis in children.

**Methods:** 126 children were divided into two groups. Group 1 was consists of 63 subjects (mean age 67.2 ± 8.4 months) who have been hospitalized or have had sepsis in neonatal period. Another 63 children (mean age 91.4 ± 24.2 months) were in group 2 who were chosen among the siblings of group 1 to minimize genetical and environmental factors which affect developing allergic diseases. The prevalence of asthma, atopic dermatitis and allergic rhinitis were compared between two groups. Comprehensive examination was performed for all subjects by the same pediatric allergist. The Turkish version of International Study of Asthma and Allergies in Children (ISAAC) questionnaires were utilized for each subject. Face to face methodology was used to complete all ISAAC questionnaires. In addition, total blood count for eosinophilia, total IgE levels were measured and skin prick tests were performed for each subjects.

**Results:** Total IgE levels and sensitivity of dermatophagoides pteronyssinus, dermatophagoides farinea were significantly lower (p < 0.05) in group 1. In addition, prevalence of allergic symptoms and diagnosed asthma were also found significantly lower (p < 0.05) in group 1. However, we found no significant difference between two groups in terms of prevalence of allergic rhinitis and atopic dermatitis.

**Conclusion:** Our study has highlighted that severe infections such as sepsis in neonatal period can protect from sensitization to environmental allergens and developing asthma in later childhood.

### PP03 Clinical overview of selective IgE deficiency in childhood

####  Athina Papadopoulou, Eleni Tagalaki, Lambros Banos, Anna Vlachou, Fotini Giannoula, Despina Mermiri

##### Allergology and Respiratory Unit, Penteli’s Children Hospital, Athens, Greece

###### **Correspondence:** Athina Papadopoulou

*Clinical and Translational Allergy* 2016, **6(Suppl 1)**:PP03

Selective IgE deficiency was recently associated with immune system dysregulation leading to lung diseases, autoimmunity and chronic urticaria in adults and children. Seventy-six children with selective IgE deficiency have been studied since 2005 (44.7 % boys, mean age 55.7 ± 34, ranging from 11 to 120 months). Clinical history was taken, SPT, nasal as well as sputum cultures, lung function test and chest x-ray were performed.

**Results:** Recurrent upper and lower airway infections were reported in 54 % whereas 23 % were admitted to hospital due to serious infection or respiratory distress. Abnormal CXR was detected in 26.3 % of cases. Family atopic history was positive in 33 % whereas 58 % of cases were passive smokers. 15.7 % had active asthma and 14 % sensitization to aeroallergens but no relation was detected between asthma symptoms and allergy sensitization or parental atopic history. Six cases (8 %) suffered from chronic rhinosinusitis and one child had chronic urticaria and Hashimoto disease. 17 % had reported atopic dermatitis.

**Conclusions:** Selective IgE deficiency seems to be related to increased prevalence of lung diseases and non atopic asthma. Low serum total IgE may be used as a marker of immune dysregulation.

### PP04 Inverse relationship between serum 25(ΟΗ) vitamin D3 and total IgE in children and adolescence

#### Athina Papadopoulou^1^, Stavroula Lampidi^1^, Marina Pavlakou^1^, Maria Kryoni^1^, Kostas Makris^2^

##### ^1^Allergic Pediatric Unit, KAT General Hospital, Athens, Greece; ^2^Clinical Biochemistry Department, KAT General Hospital, Athens, Greece

###### **Correspondence:** Athina Papadopoulou

*Clinical and Translational Allergy* 2016, **6(Suppl 1)**:PP04

Non skeletal effects of vitamin D have been discussed in recent studies and many reports refer to its role in health and disease. There is also evidence that vitamin D deficiency can impair immune function, resulting in both, overactivity or suppression. However, there are no studies relating Vitamin D levels to total IgE, a basic marker of allergic diseases. The main aim of this study was to relate levels of 25(ΟΗ) Vitamin D3 to total IgE in general population. Clinical and laboratory data from 223 children and adolescence (54.3 % boys, mean age 10.8 years, 4.5–18 years) who proceeded to the pediatric unit for vaccination, were analyzed. Serums were collected during winter 17.4 %, spring 43 %, summer 19.7 % and autumn 19.7 %.

**Results:** Mean levels of 25(ΟΗ) Vitamin D3 was 25.66 ± 10.02 pg/ml whereas deficiency (<20 pg/ml) was detected in 35 % of the cases. Mean level of IgE was 203 ± 70 KU/L whereas 30, 20.5 and 5 % reported active asthma, allergic rhinitis and eczema respectively. Levels of 25(ΟΗ) Vitamin D3 showed a negative association with total IgE (p = 0.001). This association was constant when total IgE was >60 IU/ml and independent of season, sex and age.

**Conclusion:** Total IgE was inversely related to 25 (ΟΗ) Vitamin D3 in serums. Allergic diseases might influence vitamin D synthesis or vice versa, low levels of vitamin D might increase allergic markers. More studies are needed to clarify this relation and to evaluate any clinical role.

### PP05

**WITHDRAWN**

*Clinical and Translational Allergy* 2016, **6(Suppl 1)**:PP05

### PP06

**WITHDRAWN**

*Clinical and Translational Allergy* 2016, **6(Suppl 1)**:PP06

### PP07 Asthma control questionnaire and specific IgE in children

####  Snezhina Lazova, Guergana Petrova, Dimitrinka Miteva, Penka Perenovska

##### Pediatric Clinic, University Hospital “Alexandrovska”, Sofia, Bulgaria

###### **Correspondence:** Snezhina Lazova

*Clinical and Translational Allergy* 2016, **6(Suppl 1)**:PP07

**Background:** Atopy and allergy have long been associated with asthma. Usually the presence of atopy or any allergic diseases could interfere and obstacle the control of asthma.

**Method:** For a period of 6 months we evaluated medical history data of 113 children with asthma aged 3–17 years. For all children we drew blood for IgE against inhalation and food allergies antibodies detection and asked them to fill ACQ. IgE were detected with the predesigned kit EurolinePediatric.

**Results:** We found that the children claiming to have more limited physical activities to have inadequate asthma control (ACQ ≥ 1.5) Elevated titer against specific IgE (especially birch, *D. pteronyssinus* and *D. farinae*) are correlated with worse asthma control (p < 0.05).

**Conclusion:** Closer monitoring for patients sensitive to birch, *D. pteronyssinus* and *D. farinae* is needed in order to prevent bad asthma control.

**Acknowledgements:** This work was supported by a grant from the Medical University of Sofia (Council of Medical Science, project no. 23-D/2013, grant no. 35-D/2013)

### PP08 Features of chronic urticaria of adolescents

####  Aliya Klyucharova, Olesya Skorohodkina

##### Kazan State Medical University, Kazan, Russia

###### **Correspondence:** Aliya Klyucharova

*Clinical and Translational Allergy* 2016, **6(Suppl 1)**:PP08

**Objective:** To study the clinical features of the current chronic urticaria (CU) of adolescents.

**Materials and methods:** The study involved 28 patients aged 17–19 years. A comparison group was presented of 34 patients middle-aged (30–50 years) with CU. All patients had a comprehensive examination, including clinical history, physical examination, laboratory and instrumental examination, specific allergy tests.

**Results:** Among adolescents with CU dominated male (79 %), among the middle-aged patients were predominantly female (85 %). The most common form of CU of adolescents was inducible (63.8 %): dermographic (39 %), and the combination of cold contact and cholinergic forms (14.3 %). The inducible CU met among young men 2 times more often (72.1 %) than girls (32 %). Spontaneous CU prevailed among the middle-aged patients (75 %), this form met with the same frequency among men and women. It should be noted that spontaneous urticaria of adolescents was predominantly moderate. Middle-aged patients had not only moderate disease, but mild (19.6 %) and severe (9.8 %) CU also. Urticaria in half of boys and girls was observed more than a year. 70 % of men over 30 years had symptoms of CU less than 6 months, while 55.2 % of women with CU had urticaria more than 1 year. Most common comorbidities of adolescents were a disease of gastrointestinal system (57.1 %) and helminth infections and parasitosis (21.4 %). Patients of middle age had also pathology gastrointestinal system (48.4 %), parasitosis with helminths occurred in 2 times less, and 16 % of patients diagnosed with autoimmune thyroiditis.

**Conclusions:** CU of adolescents had certain features. Urticaria observed among young men mainly. The symptoms of urticaria induced mainly by physical factors. The most common forms of urticaria is inducible: dermographic and combination cold contact with cholinergic. Idiopathic CU usually has moderate forms. The most common comorbidities are diseases of the gastrointestinal system, helminth infections and parasitosis.

### PP09 Cutaneous mastocytosis in children: a clinical analysis of 8 cases in Greece

####  Dimitra Koumaki^1^, Alkisti Manousaki^1^, Maria Agrapidi^1^, Lida Iatridou^1^, Omima Eruk^2^, Konstantinos Myridakis^3^, Emmanouil Manousakis^1^, Vasiliki Koumaki^4^

##### ^1^Second Pediatric Clinic, Aglaia Kyriakou Children’s Hospital, Athens, Greece; ^2^Department of Dermatology, Salford Royal Hospital, Salford, United Kingdom; ^3^Department of Dermatology, Agia Sophia, Children’s Hospital, Athens, Greece; ^4^Department of Medical Microbiology, Medical School of Athens, Athens, Greece

###### **Correspondence:** Dimitra Koumaki

*Clinical and Translational Allergy* 2016, **6(Suppl 1)**:PP09

**Objectives:** To characterize the clinical features, response to therapy, evolution and prognosis of pediatric cutaneous mastocytosis in a children hospital in Athens, Greece.

**Methods:** We conducted a file review of children diagnosed with cutaneous mastocytosis at Children’s Hospital in Athens, Greece, over the last year. We evaluated gender, age at onset, character and distribution of lesions, associated symptoms and course of the disease.

**Results:** In total 8 children were diagnosed with mastocytosis based on clinical presentation and on histological examination of the skin using special stains. There were 62.5 % cases of urticaria pigmentosa, 12.5 % of cases of mastocytoma and 25 % of diffuse cutaneous mastocytosis. In 100 % of cases disease onset was in the first year of life. There was a male predominance 2:1. There was a male predominance 2:1. The majority of lesions were distributed over the trunk and limbs. Different kinds of associated symptoms were noticed. None of the cases was familial. Treatment did not modify the disease evolution.

**Conclusion:** Pediatric onset mastocytosis is an unusual disease with an often benign course. Most cases of pediatric mastocytosis appeared within the first year of life, especially on the trunk. The most common clinical form of mastocytosis was urticaria pigmentosa followed by diffuse cutaneous mastocytosis and mastocytoma. The disease in childhood is less likely to have a systemic component. The prognosis of pediatric mastocytosis in general is good.

## POSTER VIEWING SESSION 2: Food allergy—Anaphylaxis (PP10–PP47)

### PP10 Prognostic factors in egg allergy

####  Maria Dimou, Maria Ingemansson, Gunilla Hedlin

##### Astrid Lindgren Children’s Hospital, Karolinska University Hospital, Stockholm, Sweden

###### **Correspondence:** Maria Dimou

*Clinical and Translational Allergy* 2016, **6(Suppl 1)**:PP10

**Introduction:** Egg allergy is one of the most common food allergies in children. Even if tolerance has developed in 70 % of the children by the age of 16, there is evidence that children outgrow their allergy at older age now compared to past decades. There is a variety of symptoms from eczema to anaphylaxis. Comorbidity with other allergic diseases is common. The aim of this study was to evaluate if specific symptoms, comorbidities or biochemical markers could be used as prognostic factors in the development of tolerance.

**Methods:** 52 children (28 boys and 24 girls) with a mean age of 7.8 (±4.1) years underwent oral egg challenge in 2012–2013. Their histories included comorbidities like atopic eczema (40 %) and peanut allergy (40 %). 17 % of the participants had no comorbidities. When exposed to egg 79 % of children had a history of skin symptoms, 46 % gastrointestinal tract symptoms, 11 % airway symptoms and 4 % anaphylaxis. Using logistic regression, we have searched for associations between age, gender as well as specific clinical characteristics and biochemical markers (IgE to egg and ovomucoid) and the oral egg challenge result.

**Results:** 33 children had a positive egg challenge. Preliminary analysis not including any covariates showed nominally significant associations between IgE to egg ≥0.35 kU/l and IgE to the egg component ovomucoid ≥0.35 kU/l and positive oral egg challenge result (p-values 0.028 and 0.028 respectively). However, adjusting for age at provocation, only the association between IgE to egg and the oral challenge result remained nominally significant. There was no association between comorbidities or a specific symptom and the oral egg challenge result.

**Conclusions:** Positive IgE to egg was the only factor that could be used as a prognostic factor for the result of an oral egg challenge in children with a history of egg allergy.

### PP11 Evaluation of the efficacy of an amino acid-based formula in infants who are intolerant to extensively hydrolysed protein formula

####  Nitida Pastor^1^, Delphine de Boissieu^2^, Jon Vanderhoof^3^, Nancy Moore^1^, Kaitlin Maditz^1^

##### ^1^Mead Johnson Pediatric Nutrition Institute, Evansville IN, USA; ^2^Private Practice, Boulogne, France; ^3^Boston Children’s Hospital, Boston MA, USA

###### **Correspondence:** Nitida Pastor

*Clinical and Translational Allergy* 2016, **6(Suppl 1)**:PP11

**Background:** Nearly 2.2–2.5 % of infants and children under 3 years of age are diagnosed with cow’s milk protein allergy (CMPA), resulting in atopic dermatitis, diarrhea, urticaria, vomiting, and gastroesophageal reflux (GER) [1,2]. Current management for infants and children with CMPA includes complete avoidance of cow’s milk protein and initiation of extensively hydrolyzed protein (EHP) formula. However, in infants and children unable to tolerate EHP formulas, amino acid-based formulas are often recommended.

**Objective:** To evaluate the efficacy of an amino acid-based formula in infants between the ages of 1–12 months with CMPA who had history of weight loss of >0.5 WHO reference z-score while on an EHP formula.

**Methods:** This was an observational, prospective, multi-center study conducted in France. Infants were put on an amino acid-based formula for 12 weeks. The primary outcome for this study was infant z-score change over the 12 weeks of feeding. Formula efficacy was evaluated by body weight gain and evaluation of allergic manifestations (atopic dermatitis (AD), bloody stools, GER score, chronic diarrhea, and urticaria).

**Results:** Thirty (30) of 32 infants completed the 12-week feeding period. Mean weight gain over the 12 week feeding period was +0.43 ± 0.28 (mean ± SD). Improvement was observed for all allergic manifestations, both in terms of the number of infants presenting symptoms and the intensity of symptoms. For AD, 13 presented and 7 continued at end of study (SCORAD improvement p = 0.02). Over the 12 week feeding period, an improvement of the overall GER score was observed, −10.5 ± 1.8 (mean ± standard error, p < 0.01).

**Conclusions:** The amino acid formula supported healthy weight gain and improvement in allergic manifestations in CMPA infants who had a history of intolerance to EHP formulas.

**References**

1. Crittenden RG, Bennett LE. J Am Coll Nutr 2005;24(6 Suppl):582S–91S.
2. Vandenplas Y, et al. Acta Paediatr 2012;101(11):1105–9.

### PP12 Anaphylaxis and epinephrine auto-injector use: a survey of pediatric trainees

####  Adeli Mehdi, Shaza Elhassan, Carolin Beck, Ahmed Al-Hammadi

##### Pediatric Department, Hamad Hospital, Doha, Qatar

###### **Correspondence:** Ahmed Al-Hammadi

*Clinical and Translational Allergy* 2016, **6(Suppl 1)**:PP12

**Background:** Anaphylactic shock is a life-threatening circumstance which requires urgent and proper medical management. The delay in making an accurate diagnosis, initiating appropriate treatment and inappropriate use of epinephrine can lead to death.

**Objectives:** This study is designed to evaluate and emphasize the paramount importance of the trainee knowledge about anaphylaxis, the treatment methods, life-saving medications, the route of administration and the dosage.

**Method:** This is a cross-sectional two phase questionnaire based survey at Hamad General Hospital’s Pediatrics department, in Doha-Qatar.

**Results:** In phase 1, the questionnaires were distributed to 96 trainees. The response was 98 % (94 responses). 84 trainees (89.3 %) reported knowing how to treat and a total of 44 (50 %) claimed not being trained at all. Epinephrine was selected as a life saving drug by 89 (94 %). Correct Epinephrine concentration was known by 77 (83 %). For phase 2, questionnaires were distributed to 94 trainees who responded to the stage 1 and the response rate was 89 % (84). 84 % claimed they heard about Epinephrine Auto injector, 72 % claimed knowing when to use it .23 (27 %) did not know. Anaphylaxis was the case of using it in 71 %. Only 43 responders (51 %) know the right location and the method of injection.

**Conclusion:** Although prompt treatment with epinephrine is deemed to be critically important for survival in anaphylaxis, we have huge gap between theoretical knowledge about epinephrine concentration and site of administration of epinephrine and fundamental practice among pediatrics trainees. Analysis of these data necessitates the urgent need of a concrete program for teaching the trainees, especially the pediatrics fellows to solidify their knowledge about anaphylaxis. More important practical guidelines about the site of administration, what concentration and how to act fast when faced with anaphylaxis is needed to be taught to current and future trainees.

### PP13 Anaphylaxis in children: acute management in the Emergency Department

####  Ioana Maris^1^, Ronan O’Sullivan^2^, Jonathan Hourihane^3^

##### ^1^Cork University Hospital, Cork, Ireland; ^2^Bon Secours Hospital, Cork, Ireland; ^3^University College Cork, Cork, Ireland

###### **Correspondence:** Ioana Maris

*Clinical and Translational Allergy* 2016, **6(Suppl 1)**:PP13

**Hypothesis:** Several studies suggest an increase in both the prevalence of food allergy and in the frequency of emergency department (ED) visits for food-related allergic reactions, including anaphylaxis.

**Aim:** To describe the key features of anaphylactic reactions in children and their management when presenting to the Emergency Department.

**Study design and method:** Patients aged 0–16 years who presented to a large academic ED, and met diagnostic criteria for anaphylaxis, were consecutively included from July 2013 to February 2015. We collected data on patient characteristics, suspected triggers, signs and symptoms, ED management, and discharge recommendations.

**Results:** 48 cases presented to the ED with anaphylaxis, either directly (77 %) or referred by a General Practitioner (23 %). Anaphylaxis was the first allergic event in 81 % of the cases. Food caused 73 % of events, with egg (25 %) and cashew nut (17 %) being the most common eliciting foods. Skin (92 %) and airway (89 %) symptoms predominated, with gastro-intestinal (48 %) and cardio-vascular symptoms (46 %) also frequent. Emergency medication was given by lay person before presenting to ED in 33 % of cases. Adrenaline i.m. was given in 50 % of the cases: in ED in 8/24 cases (33 %), self administered in 5/24 cases (21 %), by GP prior to ED attendance in 9/24 cases (37 %), and in 2 cases by paramedics. 65 % were hospitalized. None of the 7 new patients (18 %) discharged directly from ED, were given adrenaline auto injectors prescription and training, but they were all referred to a specialist clinic.

**Conclusions:** Food allergens are the main triggers for anaphylaxis in Irish children. The rate of Adrenaline use in emergency setting clearly needs to improve in Ireland, and barriers to Adrenaline use must be addressed.

### PP14 Understanding Cumbrian schools preparedness in managing children at risk of anaphylaxis in order to provide training and support which will create healthy and safe environments for children with allergies

####  George Raptis^1^, Louise Michaelis^2^

##### ^1^North Cumbria University Hospitals, Whitehaven, United Kingdom; ^2^Great North Children’s Hospital, Newcastle upon Tyne, United Kingdom

###### **Correspondence:** George Raptis

*Clinical and Translational Allergy* 2016, **6(Suppl 1)**:PP14

**Background:** Allergic diseases can have detrimental effects on physical, emotional and social life especially in childhood and they can affect significantly children’s ability to flourish.

**Aim:** To develop an effective model of care for allergic children at school and ensure all staff are familiar with and able to implement it.

**Methods:** A self-completed postal questionnaire was sent to all Cumbrian schools (n = 312) to assess three primary areas of concern: existing protocols for anaphylaxis management, training offered by and to schools and preventative measures. Subsequently, training on how to deal with anaphylaxis was offered to 10 % (n = 18) of all participating schools. A follow-up questionnaire was sent to measure the preparedness and confidence by staff following the training.

**Results:** Prior to training 47 % of all participating schools felt completely confident dealing with anaphylaxis while 55 % felt prepared for anaphylaxis in children without a prior history of allergies. 78 % of the schools felt that further training in anaphylaxis would be beneficial. 82 % believed that a national school policy in managing anaphylaxis is needed and 96 % agreed with the genetic provision of adrenaline auto-injectors along with individual anaphylaxis plans to be kept at school.

45 % of the schools have a no-nut policy while 56 % have one on no food-sharing. However, only 38 % have a no eating-utensil sharing policy and 34 % one on no eating-on-transport to and from school.

The confidence level in dealing with anaphylaxis increased to 95 % post training while 100 % of them are updating their no-nut, no food and no eating-utensil sharing and no eating-on- transport to and from school policies.

**Conclusions:** Adopting a county-wide, age appropriate school protocol including training in anaphylaxis led by the school and supported by all the stake holders will ensure that all school staff can prevent, recognise and initiate safe treatment of anaphylaxis.

### PP15 A new valid and reliable parent and child questionnaire to measure the impact of food protein enterocolitis syndrome on children: the FPIES Quality of Life Questionnaire (FPIESQL), Parent and Child Short Form

####  Audrey DunnGalvin^1^, Matthew Greenhawt^2^, Carina Venter^3^, Jonathan Hourihane^2^

##### ^1^University College Cork, Cork, Ireland; ^2^University of Michigan, Ann Arbor MI, USA; ^3^University of Portsmouth, Portsmouth, United Kingdom

###### **Correspondence:** Audrey DunnGalvin

*Clinical and Translational Allergy* 2016, **6(Suppl 1)**:PP15

**Rationale:** No tool currently exists to measure health related quality of life (HRQL) in Food Protein Enterocolitis Syndrome (FPIES). We investigated the ability of the FPIES Quality of Life Questionnaire (FPIESQL), Parent and Child Short Form to accurately assess the impact of FPIES on the HRQL of children, aged 0^+^ years. FPIES are similar to severe forms of food allergy where there is the risk of death, but due to hypovolemic shock rather than anaphylaxis. FPIES are also different from classic IgE mediated food allergy, as the foods involved are often atypical foods and not validated tests exists.

**Methods:** Following initial validation with 148 Irish, UK, and US caregivers of children with FPIES of the protocol instrument, 68 Irish and UK parents completed the short online version of the questionnaire on the impact of FPIES on their child (10 items) and on the parent themselves (10 items). The measure underwent further psychometric analysis including factor analysis and reliability analysis.

**Results:** Parents of children (58 % male) aged between 6 months and 8 years completed the questionnaires. Analyses demonstrated high reliability (α = .92). Bartlett’s test of sphericity, Χ^2^(45) = 257.3, *p* ≤ .001, showed good correlation between items. Two components had eigenvalues over the KMO criterion of 1 and explained 70 % of the variance in the impact of FPIES on HRQL, demonstrating construct validity. There was a high positive correlation between independent clinical items and scores on the questionnaire.

**Conclusions:** The new questionnaire allows for a disease specific analysis of the impact of FPIES on HRQL in both parents and children. It will provide a powerful disease specific outcome and tracking measure in both research and clinical contexts.

### PP16 An in-depth case study investigation of the experiences of teenagers and young adults in growing up and living with food allergy with emphasis on coping, management and risk, support, and social and self-identity

####  Evelyn O’Regan, Duncan Cronin, Jonathan Hourihane, Anna O’Reilly, Audrey DunnGalvin

##### University College Cork, Cork, Ireland

###### **Correspondence:** Audrey DunnGalvin

*Clinical and Translational Allergy* 2016, **6(Suppl 1)**:PP16

**Rationale:** There is limited research into coping and management strategies of teenagers and young adults living with food allergy, particularly with a strong developmental focus using either qualitative or quantitative methods.

**Aim:** The current study qualitatively investigates the experiences of teenagers and young adults in growing up and living with food allergy with emphasis on coping, management and risk, support, and social and self-identity.

**Method:** In-depth case study interviews were conducted with participants aged 18–24, and analysed following the method of Interpretative Phenomenological Analysis.

**Results:** The analysis yielded five super-ordinate themes: “change as a process”, “being consumed by panic”, “using mechanisms to cope with fear”, “creating internal and external stability”, and “compromising safety for normality”, with the overarching theme “gaining control”. Findings show that coping and management in food-allergic young adults is a journey. This journey is similar for all young adults in that they all are striving for control, though their experiences are unique. All are challenged by the developmental juncture with which they are faced as young adults and are informed by previous developmental transition experience. While they move towards developing independence, young people rely on external support for their safety and to cope with the fear and anxiety around reactions.

**Conclusions:** The findings from this study add complexity to our existing knowledge of coping and management in food allergy from a developmental perspective, and can be used as a basis for interventions or further qualitative and quantitative research in the field.

### PP17 Cow’s milk protein allergy in Constantine. A retrospective study of 62 cases between 1996 and 2013

####  Foued Abdelaziz^1^, Dounia Khelifi-Touhami^2^, Nihad Selim^3^, Tahar Khelifi-Touhami^1^

##### ^1^Pediatric Pulmonology and Allergy, Annaba, Algeria; ^2^Pharmacy Department, Constantine, Algeria; ^3^University Hospital, Annaba, Algeria

###### **Correspondence:** Foued Abdelaziz

*Clinical and Translational Allergy* 2016, **6(Suppl 1)**:PP17

**Background:** The epidemiology of cow’s milk protein allergy (CMPA) remains poorly defined in North Africa. The aim of this study is to report the epidemiological, clinical and evolutionary characteristics of CMPA in children living in Constantine.

**Method:** This is a retrospective study that included all children with CMPA seen in a pediatric allergy clinic from 1996 to 2013 and all available data were used.

**Results:** 62 cases were gathered (32 boys and 30 girls). The average age when the reaction to cow’s milk formula took place was 4 months (3 days–14 months) and 43 % received some cow’s milk supplement during the lactation period; a first degree family history of atopy was revealed in 54 % of cases. The clinical signs were in 54 % of cases cutaneous. Prick-tests were performed on 87 % of the children and CMP-specific IgE in 45 % of them. 32 % had a Diagnostic elimination diet.

All patients adhered to strict avoidance of CMP and received an eHF with a good tolerance in 86 % of cases, goat’s milk used without medical advice induced urticaria or anaphylaxis and Camel’s milk was not tolerated. Reintroduction of CMP was conducted in hospital in 75 % of cases, the age of reintroduction varied from 12 months to 3 years. In 10 % of cases reintroduction was done at home by parents. CMPA remains persistent in 9 % of cases and 48 % of the patients in this study developed asthma and or allergic rhinitis.

**Conclusion:** CMPA is probably the most common food allergy in infants under 12 month in Algeria, CMPA testing should include other food allergens and aeroallergens. Home reintroduction of CMP and use of goat’s milk are not permitted. More studies on camel’s milk should be done. An early identification of, and intervention for, CMPA is essential to avoid vital complications.

### PP18

**WITHDRAWN**

*Clinical and Translational Allergy* 2016, **6(Suppl 1)**:PP18

### PP19 Cow’s milk and egg oral immunotherapy in children older than 5 years

####  Pablo Merida, Ana M^a^ Plaza, Juan Heber Castellanos, Adrianna Machinena, Montserrat Alvaro Lozano, Jaime Lozano, Olga Dominguez, Monica Piquer, Rosa Jimenez, M^a^ Teresa Giner

##### Pediatric Allergy Department, Hospital Sant Joan de Déu, Universitat de Barcelona, Barcelona, Spain

###### **Correspondence:** Adrianna Machinena

*Clinical and Translational Allergy* 2016, **6(Suppl 1)**:PP19

**Aim:** To report clinical features and outcomes of oral immunotherapy (OIT) in children with cow’s milk (CM) and egg allergy (EA).

**Methods:** Retrospective study of children with CM and EA who have undergone OIT with CM or raw egg from January 2011. Variables: age at oral food challenge (OFC), gender, atopic history, skin prick test (SPT), total and specific IgE (s-IgE) and OIT outcomes.

**Results:** 87 patients included, 39 with CMA and 48 with EA. 58.6 % boys, 67 % with atopic family history, 45 % had atopic dermatitis and 55.2 % had one or more food allergies.

*CM allergic children:* 69.2 % (27) had anaphylaxis at OFC before OIT, needing epinephrine 30.8 % (12). Mean CM and casein SPT and s-IgE were: 10.11 mm (3.5–19), 8.9 mm (3.5–17.5), 80.5 KU/L (1.34–688), and 80 KU/L (1–694), respectively. Mean time at build-up phase 8.7 months (2–33), with statistical significance for those with casein s-IgE >50 KU/L. At the maintenance phase only 1 child withdrew due to adverse events. The achieved dose range was 75–200 ml.

*EA children:* 39.6 % (19) had anaphylaxis at OFC before OIT, needing epinephrine 47.3 % (9). Mean egg-white, ovoalbumin, ovomucoid SPT and s-IgE were: 8.3 mm (0–13.5), 8.3 mm (0–14.5), 7.4 mm (0–13.5), 17.6 KU/L (0.35–101), 10.6 KU/L (0.35–101) and 12 KU/L (0.35–101), respectively. Mean time at the build-up phase 7.2 months (2–25). 5 children withdrew due to adverse events, 3 at build-up and 2 at maintenance phase. The achieved dose range was 15–30 ml of raw egg-white.

**Conclusion:** Most of the children (93 %) completed our OIT protocol for CMA and EA. The time to complete the build-up period was longer for children with casein s-IgE >50 KU/L. In EA, drop-outs due to adverse events were more frequent.

### PP20 Professionals’ awareness of management of Cow’s Milk Protein Allergy (CMPA) in North Wales Hospitals

#### Konstantinos Kakleas, Manohar Joishy, Wendmu Maskele, Huw R. Jenkins,

##### Ysbyty Gwynedd, Bangor and University Hospital of Wales, Cardiff, United Kingdom

###### **Correspondence:** Konstantinos Kakleas

*Clinical and Translational Allergy* 2016, **6(Suppl 1)**:PP20

**Background/Aims:** In the UK it is estimated that 2–2.5 % of infants suffer from CMPA, but the diagnosis can be easily missed and it is important to recognise this condition promptly and treat effectively. Our aim was to investigate whether pediatric doctors, working in Betsi Cadwaladr University Health Board (BCUHB) comprising of North Wales Hospitals, were aware of the CMPA guidelines and if they implemented these guidelines appropriately in clinical practice.

**Methods:** All pediatric doctors working in pediatric departments of BCUHB were asked to complete a specific questionnaire in order to determine their awareness on diagnosis and management of CMPA. Of the 50 distributed questionnaires, 40 were completed and returned to us.

**Results:** About 25 % of doctors were unaware of CMPA guidelines and 30 % stated they were not confident in managing this condition. Nearly 80 % of medical staff provided the right advice for CMPA in breast fed infants including duration of treatment and appropriately selected the hydrolysed formula for bottle fed infants. A good proportion of medical staff deviated from the guidelines regarding the management of CMPA in cases of anaphylaxis (52.5 %) and failure to thrive (FTT) (85 %).

**Conclusions:** Overall medical staff had a good understanding of CMPA guidelines. However nearly a quarter of staff was not aware of the existence of guidelines and another third stated they had not read them. In addition the majority deviated from guidelines in terms of FTT and anaphylaxis in CMPA. Therefore it is important to educate staff regarding the presence of guidelines and management of CMPA, as prompt and correct diagnosis of this condition will reduce the parental anxiety and will decrease the financial burden on the Health System.

### PP21

**WITHDRAWN**

*Clinical and Translational Allergy* 2016, **6(Suppl 1)**:PP21

### PP22 Anaphylaxis: the great unknown for teachers. Presentation of a protocol for schools

####  Mercedes Escarrer^1^, Agustín Madroñero^1^, Maria Teresa Guerra^2^, Juan Carlos Julia^3^, Juan Carlos Cerda^3^, Javier Contreras^4^, Eulalia Tauler^5^, Maria Jesus Vidorreta^3^, Ana Rojo^6^, Silvia Del Valle^5^

##### ^1^Clínica Juaneda, Palma de Mallorca, Spain; ^2^Cadiz, Spain; ^3^Valencia, Spain; ^4^Madrid, Spain; ^5^Barcelona, Spain; ^6^Granada, Spain

###### **Correspondence:** Mercedes Escarrer

*Clinical and Translational Allergy* 2016, **6(Suppl 1)**:PP22

**Background**: The cases of food allergies have doubled in the last 10 years and the number of hospitalisations caused by severe allergic reactions has increased seven fold in the same period.

**Objective**: To emphasise the importance of reaching an early diagnosis and appropriate treatment in order to prevent such serious incidents and improve patients’ quality of life.

**Methods**: In 2014 we made a questionnaire on 2481 Spanish teachers between November 2013 and January 2014, with the following questions:

- Do you know what anaphylaxis is?
- Are there any students in your class who have ever had an anaphylactic reaction?
- Would you know what to do if a student had an anaphylactic reaction in your school?
- Would you know how to administer the medication if a student had an anaphylactic reaction in your school?

**Fig. 2:**
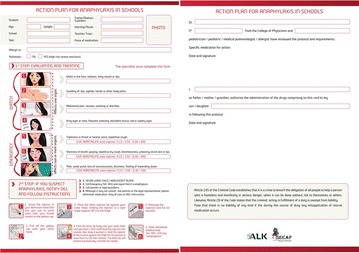
Protocol

**Results**: In the survey it became evident that knowledge about anaphylaxis was scarce, only 40.5 % reported knew what anaphylaxis was and only 11 % said they would know how to administer the auto-injector adrenaline (Fig. 2).

**Conclusion**: We present a protocol for teachers in case of anaphylaxis, where we explain simply the steps and how to use the auto-injector adrenaline with drawings.

### PP23 Challenges facing children with food allergies and their parents in out of school activity sectors

####  Niamh Flynn

##### University of Southampton, Dublin, Ireland

###### **Correspondence:** Niamh Flynn

*Clinical and Translational Allergy* 2016, **6(Suppl 1)**:PP23

**Background:** Food allergy (FA) affects up to 4–7 % of primary school children in Europe. Despite the rise in diagnosis, many community members are unaware that a severe allergic reaction, such as anaphylaxis, could result in death. Food allergy can often be confused with food intolerances leading to misunderstandings regarding severe allergic reactions. It can also have a significant effect on the quality of life (QoL) of the FA child and their parents in social interactions.

**Objective:** The focus of this research is in the area of extra-curricular activities that FA children participate in. No previous FA studies have looked at this before. Such a study is of significance in order to understand parental concerns and if necessary improve supports within the community.

**Methods:** A qualitative approach was used by conducting semi structured telephone interviews and analysed using thematic analysis.

**Results:** The interviews produced a number of key findings: food was regularly provided at one or more of the activities which the child engaged in, although primarily in team sports; parents tended to stay at the activity partly due to trust and also due to a sense of responsibility.

**Conclusion:** The main conclusion drawn from this study was that there is a lack of awareness and understanding of FA indicating that there is also no risk perceived by some coaches or instructors. Some sporting bodies were more proactive with regards training than others, but central to the risk of food allergen exposure is the education of the coaches and instructors in all activities where there is a potential for food to be provided. This research proffers engagement with coaches and instructors at community level to recognise that this is a real concern for parents of FA children and also to get governing sports bodies involved in developing FA and anaphylaxis policies.

### PP24 A review of food challenges at a Regional Irish Centre

####  Gary Foley, Carol Harmon, John Fitzsimons

##### Pediatric Assessment Unit, Our Lady of Lourdes Hospital, Drogheda, Ireland

###### **Correspondence:** Gary Foley

*Clinical and Translational Allergy* 2016, **6(Suppl 1)**:PP24

**Introduction:** Parents often overestimate the likelihood of their child having a food allergy.[1] Food challenges allow the health care professional to determine if a true food allergy exists. Food challenge audits such as this will help tailor the decision whether or not a food challenge is required in this subpopulation.

**Aims:**

1. To review the activity of food allergen challenges.
2. To determine food challenge results verses international standards.

**Methods:** Data of food challenges that occurred from 2012–2014 were reviewed and placed into the following categories; suspect allergen, type of challenge performed, IgE sample obtained, skin prick testing (SPT) performed, passed challenge, failed challenge, type of reaction if failed, treatment given if failed and follow up at 24 h.

**Exclusion Criteria:** Drug allergy challenges and food challenges with incomplete information were excluded.

**Results:** A total of 164 challenges were performed over the time period. 161 of these were food related. The main food allergens challenged were milk (N = 54), peanut (N = 47) and egg (N = 40). There was one case of a food challenge (wheat) with exercise. A total of 45 patients underwent SPT prior to the food challenge, 119 underwent IgE testing and 35 had both investigations. There were 119 (73 %) passed and 42 (27 %) failed food challenges. There was one case of anaphylaxis. The most common post challenge reaction noted was urticaria (N = 22). There were two incidents of respiratory reactions requiring Beta-2 agonists. One case of eczema flare at 18 h was documented.

**Conclusion:** A significant number of food challenges occurred without any prior investigation. There was a large discrepancy between the number of those who passed verses failed the food challenges. In time, the international standard of a near 50 % pass verses 50 % fail rate should be reached with more stringent criteria.[2] Until then limited resources will lend to higher than averages pass rates.

**References**

1. Venter C, et al. Prevalence of sensitization reported and objectivity assessed food hypersensitivity amongst 6-year old children: a population based study. Pediatr Allergy Immunol. 2006;17(5):356–63.
2. Food allergy in children and young people. NICE Guidelines 2011.

### PP25 The use of epinephrine in infants with anaphylaxis

####  Krasimira Baynova, Ávila Maria Del Robledo, Labella Marina

##### Department of Allergy, University Hospital “Virgen del Rocío”, Seville, Spain

###### **Correspondence:** Krasimira Baynova

*Clinical and Translational Allergy* 2016, **6(Suppl 1)**:PP25

**Background:** Anaphylaxis is a potentially life-threatening systemic hypersensitivity reaction and its first-choice treatment is Epinephrine given intramuscularly (IM). On discharge, all patients should be prescribed epinephrine auto injectors, and referred to an allergist. But do we use the epinephrine appropriately in case of anaphylaxis or do we skip it?

**Methods:** We studied retrospectively 41 children who performed anaphylactic reaction and who were admitted to the emergency department or were attended by emergency mobile units during a 9-years period (2006–2015) in a 700,000 population. We determined the prevalence of use of epinephrine as a first-choice treatment and the percentage of patients in whom epinephrine auto injector was prescribed when they were discharged.

**Results:** Only 22 % of the patients were treated with epinephrine IM when anaphylaxis was presented. Just 4.8 % of the patients received prescription of epinephrine auto injector when hospital/ED discharge. All patients were referred to an allergist.

**Conclusions:** The use of epinephrine as a first choice treatment is still not solid. The prescription of epinephrine auto injectors when patients are discharged from emergency departments is still an issue.

**Keywords:** anaphylaxis; emergency; infant; epinephrine.

### PP26

**WITHDRAWN**

*Clinical and Translational Allergy* 2016, **6(Suppl 1)**:PP26

### PP27

**WITHDRAWN**

*Clinical and Translational Allergy* 2016, **6(Suppl 1)**:PP27

### PP28 Mother’s psychological state predicts the expression of symptoms in food allergic children

####  Aaron Cortes^1^, Alicia Sciaraffia^1^, Angela Castillo^2^

##### ^1^Hospital Clinico Universidad de Chile, Santiago, Chile; ^2^Hospital Carlos Van Buren, Valparaiso, Chile

###### **Correspondence:** Aaron Cortes

*Clinical and Translational Allergy* 2016, **6(Suppl 1)**:PP28

**Background:** It has been established that child allergies, such as asthma and rhinitis, have a direct impact on carers (usually the mother), increasing their likelihood of psychological disorders and social network deterioration. It is hypothesised a significant interaction between mother’s psychological state and child’s food allergy (FA) symptoms; however, there is very little information on FA regarding this topic. Therefore, this study compared the relative odds of the occurrence of gastric symptoms in the food allergic child given exposure to maternal psychosocial factors.

**Methods:** Mother’s psychological state was evaluated and analysed against the occurrence of FA gastric symptoms in their children in a cross-sectional study involving 206 participants (mothers and children). Logistic regressions were used to determine whether mother’s psychological state is a risk factor for child’s gastric symptoms and to compare the magnitude of different psychological variables as risk factors for a specific gastric symptom occurrence.

**Results:** High levels of *anxiety* were found in 44 % of the participants, *depressive symptoms* on the 21.4 and 68 % had moderate or high *psychosocial impact* due to CFA. Low *perceived social support* was found in 21.4 % of the mothers. Higher *CFA*-*Related Impact (CFA*-*RI)* and *CFA*-*Related Social Impact (CFA*-*R SI)* in the mother increase the possibility for abdominal pain (OR = 2.04; p < .001) and diarrhoea (OR = 1.32; p = .05) in the child. The possibility for abdominal bloating in the child increases when the mother suffer from higher *anxiety* (OR = 4.45; p < .001), lower *perceived social support (PSS)* (OR = 3.17; p = .002) and *CFA*-*RI* (OR = 1.32; p < .05).

**Conclusions:** The psychological impact of caring a food allergic child and the perceived social support can predicts the occurrence of allergic symptoms in children. CFA is propounded as a process where biological, psychological and social variables have a relationship of mutual influence. Therefore, a comprehensive care strategy that considers the family perspective is proposed to achieve a more inclusive and integrative care of CFA focused on “families living with food allergy”.

### PP29 The correlation between sIgE towards tree nuts and birch pollen in a Danish Pediatric Allergy Clinic

####  Nanna Juel-Berg, Kirsten Skamstrup Hansen, Lars Kærgaard Poulsen

##### Allergy Clinic, Copenhagen University Hospital-Gentofte, Copenhagen, Denmark

###### **Correspondence:** Nanna Juel-Berg

*Clinical and Translational Allergy* 2016, **6(Suppl 1)**:PP29

**Background:** Tree nut allergy is a frequent medical problem. Some are allergic to tree nuts due to birch pollen cross reactivity whereas others have a primary tree nut allergy. There can be great regional variance in the serological profile and hence in clinical allergy. A pilot study suggested that hazelnuts, walnuts, pistachio nuts and cashew nuts were the most common nuts involved in allergic reactions in a Danish Pediatric population.

**Aim:** To investigate the correlation between sIgE towards four common tree nuts and the correlation between sIgE towards hazelnuts and birch pollen amongst patients in a Danish outpatient allergy clinic.

**Method:** Specific IgE in all patients born after January 1st 1991 with sensitization to tree nuts and birch pollen were sought in our sIgE (ImmunoCAP^®^) database.

GraphPad Prism 6 software was used for statistical analysis.

The interrelationship between sIgE was pictured in XY-plots and Spearman correlation was calculated for sIgE towards hazelnut and birch pollen and for sIgE towards hazelnut, walnut, pistachio and cashew measured in the same patients.

**Results:** The number of patients with sIgE >0.35 kU/l and median sIgE values:

- Hazelnut: n = 560, median 10.75 kU/l
- Cashew: n = 163, median 2.77 kU/l
- Pistachio: n = 182, median 2.55 kU/l
- Walnut: n = 164, median 3.85 kU/l
- Spearman correlation coefficient between:
- Hazelnut and birch: r_s_ = 0.7947
- Cashew and hazelnut: r_s_ = 0.2172
- Pistachio and hazelnut: r_s_ = 0.2854
- Walnut and hazelnut: r_s_ = 0.3132
- Walnut and cashew nut: r_s_ = 0.4696
- Pistachio nut and cashew nut: r_s_ = 0.8313
- Walnut and pistachio nut: r_s_ = 0.4648

**Conclusions:** Our findings show a strong relationship between sensitization towards cashew nut and pistachio nut, and hazelnut and birch pollen and a moderate relationship towards walnut and cashew nut, and walnut and pistachio nut. Results from the same population are underway to determine the clinical association of these findings.

### PP30 Food allergy in children: evaluation of parents’ use of online social media

####  Andreia Florina Nita^1,2^, Ioana Valentina Nenciu^1,2^, Adina Lazar^2^, Dumitru Oraseanu^1,2^

##### ^1^Carol Davila University of Medicine and Pharmacy, Bucharest, Romania; ^2^Grigore Alexandrescu Clinical Emergency Hospital for Children, Bucharest, Romania

###### **Correspondence:** Andreia Florina Nita

*Clinical and Translational Allergy* 2016, **6(Suppl 1)**:PP30

**Introduction:** Worldwide, 8 % of the children are diagnosed with food allergy, which means 2 children in every classroom. In the last 10 years food allergies among children have increased by 50 % (CDC). In light of this concerning facts, more and more parents have to cope with an allergic child, being responsible for risk assessment and management of their child’s condition. Nowadays, social media is a powerful tool that parents use to get informed. Despite this, little has been published about social media role in health issues.

**Objective:** To evaluate the content of communication in parents dedicated websites regarding topics on food allergy in children.

**Design:** We searched for non-medical online networking websites dedicated to parents, with topics and talks regarding food allergy in children and identified 19 online sites which we further evaluated in respect to content form (personal experience, information) and correctitude of information from a medical point of view.

**Results:** The number of answers to a topic was variable according to the popularity of the website, ranging from 8 to 215. However, the number of unique visualizations was exponentially higher, ranging from 3302 to 25.123.

Parents of children with food allergy either medically confirmed or suspected use social media tools to ask for information and share personal experiences. The hierarchy of most common reasons to use the online media is the following: (1) to describe an acute reaction and ask for opinions on management and causes (2) to get informed about food diet and treatment and find out others experience with specific treatment, including different brands, 3. Ask other parents to recommend a physician and/or a medical laboratory. Other important concerns raised by parents were the difficulties encountered regarding collectivity attendance due to impossibility of providing food specific diet. There were some medically inaccurate recommendations such as use of homeopathic treatment or inadequate definition of medical terms.

**Conclusions:** Online social media provides a place to share personal experiences, receive emotional support and get information on medical management, suggesting that this tool should be better managed for greater efficiency.

**Funding:** This paper was co-financed from the European Social Fund, through the Sectorial Operational Programme Human Resources Development 2007–2013, contract POSDRU/187/1.5/S/155463 “Supporting excellence in scientific interdisciplinary doctoral research in the economic, medical and social fields”, coordinator The Bucharest University of Economic Studies”.

### PP31 The impact of food allergy on quality of life: FAQLQ questionnaire

####  Rita Aguiar, Anabela Lopes, Maria J. Paes, Amélia S. Santos, M. A. Pereira-Barbosa

##### Hospital de Santa Maria-Centro Hospitalar Lisboa Norte, Lisbon, Portugal

###### **Correspondence:** Rita Aguiar

*Clinical and Translational Allergy* 2016, **6(Suppl 1)**:PP31

**Introduction:** Food allergy (FA) affects 8.5 % percent of children in Portugal. This study evaluates the impact of on quality of life (QoL).

**Methods:** We performed a study using the Food Allergy Quality of Life Questionnaire **FAQLQ.** The appropriate questionnaire will depend on the age of the patient. **FAQLQ-PF** (0–12 years) as perceived by the parent. **FAQLQ-CF** and **FAQLQ-TF** are self-administered tools that measure the impact of FA on children (8–12 years) and teenagers (13–17 years). We applied the questionnaire to patients under 18 years diagnosed with FA in the last 2 months.

Information on food allergens and demographic data was collected for all children.

**Results**: The questionnaire FAQLP-PF was applied to 13 parents of children under 12 years. The questionnaires FAQLP-CF and FAQLQ-TF were applied to 7 children and 9 teenagers, respectively.

The mean age of the food allergy population was 9 ± 5.2 (1–17 years). 16 patients (55.2 %) were male; 34.5 % had rhinitis, 27.6 % had atopic dermatitis and 13.8 % had asthma. 17 (58.6 %) were allergic to 1 or 2 foods; 12 (41.4 %) were allergic to 3 or more foods. The egg is the most common food allergen implicated in children (37.9 %), followed by milk proteins (31.09 %), shrimp (31.09 %), and fresh fruits (27.6 %).

The median score of the questionnaires were: FAQLQ-PF = 5.2; FAQLQ-CF = 4.9; FAQLQ-TF = 4.

The correlation between the severity of the reaction (Mueller classification) and the FAQLQ is 0.3 (p = 0.05). The correlation between the number of food and impairment in the QoL was not found.

**Conclusions:** The parents were the group most affected about the impact on quality of life (FAQLQ-PF 5.2) as they indicated that FA significantly impact meal preparation, impact on their level of stress, affected family social activities and affected their child’s school attendance. We found a positive but weak correlation between the severity of the reaction and the impairment in the quality of life (FAQLQ).

The results show that FA impairs the children’s quality of life in all age groups and also the quality of life of their families. We suggest the regular evaluation of the quality of life in the clinical management of children with FA.

### PP32 An unexpected cause of anaphylaxis: potato

####  Hatice Eke Gungor, Salih Uytun, Umit Murat Sahiner, Yasemin Altuner Torun

##### Kayseri Education and Research Hospital, Department of Pediatrics, Pediatric Allergy-Immunology Unit, Kayseri, Turkey

###### **Correspondence:** Hatice Eke Gungor

*Clinical and Translational Allergy* 2016, **6(Suppl 1)**:32

**Background:** Immediate reactions against contact to raw potato has been reported in adults with generally being in the form of an oral contact dermatitis or contact urticaria, but it may also manifest as rhinitis symptoms, wheezing or even anaphylaxis. Cooked or raw potato allergy has been rarely reported in children as some is being immediate and others being late reactions, and it usually results from ingestion.

**Objective:** We report two cases with a background of allergic diseases developed anaphylaxis one with cooked potato and the other one with raw potato.

**Methods:** We measured serum potato specific IgE and applied a skin prik test (SPT) with aeroallergens, food allergens and prick-to-prick test using raw and cooked potato.

**Results:** We found serum potato specific IgE 4.92 and 125 kU/L case 1 and case 2 respectively. We also demonstrated SPT positivity aganist raw potato and cooked potato case 1 and case 2 respectively.

**Conclusion:** We aimed to emphasize potato allergy, a rare entity, and to remind potential disorders that could develop with or after potato allergy.

### PP33 Is it clinical phenotype of allergic diseases determined by sensitisation to food?

####  Mirjana Zivanovic^1^, Marina Atanasković-Marković^2^

##### ^1^Special Hospital Sokobanja, Sokobanja, Serbia; ^2^University Children Hospital, Faculty of Medicine, Belgrade, Serbia

###### **Correspondence:** Mirjana Zivanovic

*Clinical and Translational Allergy* 2016, **6(Suppl 1)**:33

**Background:** Food allergy results from an atypical response of the mucosal immune system to orally consumed allergens. Relationship between the offending food and natural course of the disease is a little known and controversial.

**Aim:** To determine the relationship between the causative food and the clinical presentation of food allergy and other associated allergic diseases.

**Methods:** This is a review of patients who underwent skin prick testing, prick to prick testing, presence of food specific IgE and positive oral provocation test, in small number of patients.

**Results:** We were analysed 226 patients. There are ranged between 4 months and 7 years. The implicated food were as follows: CMP (24.79 %), egg (17.70 %), peanut (12.83 %), wheat (11.50 %), soy (10.18 %), tree nuts (7.96 %), fish (7.96 %), kiwi fruit (7.08 %). The median age of the children was 28 months, 52 % were boys and 48 % were girls. The frequency of atopic dermatitis was higher in children with egg allergy (72 %), compared to those with milk (28 %), or wheat (24 %), or peanut (49 %), (p < 0.001). Asthma was more frequently in children with isolated peanut allergy (65 %) than children with milk (26.2 %) and egg allergy (24.4 %), (p = 0.001). Allergic rhinitis was more frequent in children with isolated peanut allergy (41 %) compared to children with milk (4.2 %) and egg allergy (3.8 %), (p < 0.001). The frequency of pollen sensitization was higher in children with peanut allergy (64.4 %) compared to children with egg allergy (4.7 %), or milk allergy (4 %), (p < 0.001).

**Conclusion:** The type of culprit food may be important factor of clinical presentation and prognosis of food allergy and may be reason for expression other allergic conditions.

**Keywords:** allergy; children; food.

### PP34

**WITHDRAWN**

*Clinical and Translational Allergy* 2016, **6(Suppl 1)**:PP34

### PP35 Prescribing adrenaline auto-injectors in children in 2014: the data from regional pediatricians

####  Tina Vesel^1^, Mihaela Nahtigal^2^, Andreja Obermayer-Temlin^3^, Eva Šoster Križnik^3^, Mirjana Maslar^3^, Ruben Bizjak^4^, Marjeta Tomšič-Matic^5^, Sonja Posega-Devetak^6^, Maja Skerbinjek-Kavalar^7^, Mateja Predalič^8^, Tadej Avčin^1^

##### ^1^Department of Allergology, Rheumatology and Clinical Immunology, University Children’s Hospital, University Medical Center, Ljubljana, Slovenia; ^2^General Hospital in Slovenj Gradec, Slovenj Gradec, Slovenia; ^3^General Hospital in Celje, Celje, Slovenia; ^4^General Hospital in Šempeter, Šempeter, Slovenia; ^5^General Hospital in Trbovlje, Trbovlje, Slovenia; ^6^General Hospital in Izola, Izola, Slovenia; ^7^Allergologic Pediatric Ambulance, Maribor, Slovenia; ^8^General Hospital in Novo Mesto, Novo Mesto, Slovenia

###### **Correspondence:** Tina Vesel

*Clinical and Translational Allergy* 2016, **6(Suppl 1)**:PP35

**Introduction:** Previous data from the University Children’s Hospital in Ljubljana, Slovenia, showed that adrenaline auto-injector was prescribed to Slovenian children most frequently because of allergy to peanuts.

**Methods:** We prospectively collected data on interventions of immediate reactions and allergic follow-up of children which had adrenaline auto-injectors prescribed in year 2014 and were followed-up exclusively by regional paediatricians’ with special interest in paediatric allergy.

**Results:** 76 children (52 boys, 24 girls) were followed-up exclusively by regional pediatricians with special interest in paediatric allergy. 10.5 % were babies, 27.6 % 1 to 5 years old, 55.3 % 6 to 14 years old and 6.5 % 15 to 18 years old. Adrenaline auto-injector was prescribed in 63.1 % because of anaphylaxis and in 31.6 % because of urticaria and/or angioedema. In 92.1 % of children adrenaline auto-injector was prescribed because of food allergy, most frequently because of allergy to peanuts (44 children). 93.2 % of peanut allergic children had specific IgE antibodies to rArah h 2. Among food allergic children 7.2 % had multiple food allergies, 38.6 % asthma and 7.2 % suffered more than one immediate reaction. Other confirmed reasons for prescribing adrenaline auto-injector were insect sting (4 children) and inhalant allergens (1 child). In 5 % of children the cause of immediate reaction was unknown. 29.1 % of anaphylaxis was treated with adrenaline 43.7 % of children were admitted to hospital after anaphylaxis. For comparison, during 2014 adrenaline auto-injector was prescribed in 291 children during 2014 in Slovenia.

**Conclusions:** Demographic and clinical characteristics of children with prescribed adrenaline auto-injectors reported by regional paediatricians with special interest in pediatric allergy were similar to our previous data though children had fewer different food allergies.

### PP36 Who should have an adrenaline autoinjector? Adherence to the European and French guidelines among 121 allergists from the Allergy Vigilance Network

####  Guillaume Pouessel^1, 2, 3^, Etienne Beaudouin^2,4^, Anne M. Moneret-Vautrin^2,4^, Antoine Deschildre^2,3^, Allergy Vigilance Network^2^

##### ^1^Department of Pediatrics, Children’s Hospital, Roubaix, France; ^2^Allergy Vigilance Network, Vandoeuvre les Nancy, France; ^3^Pediatric Pulmonology and Allergy Department, Pôle Enfant, Hôpital Jeanne de Flandre, CHRU de Lille and Université Nord de France, Lille, France; ^4^Department of Internal Medicine, Immunology and Allergology, Jean Monnet Hospital, Epinal, France

###### **Correspondence:** Guillaume Pouessel

*Clinical and Translational Allergy* 2016, **6(Suppl 1)**:PP36

**Background:** According to the French[1] and the EAACI[2] anaphylaxis guidelines, patients with a previous anaphylaxis (apart drug allergy) or at particular risk of anaphylaxis should require prescription of adrenaline auto-injector (AAI). Our aim was to assess prescription practices of AAI by the French allergists from the Allergy Vigilance Network[3] (AVN) and to confront these to the European and French guidelines.

**Methods:** In January 2015, an electronic questionnaire was sent to the 299 allergists of the AVN.

**Results:** 121 questionnaires (40 %) were analyzed. According to the European guidelines, 77 % of the allergists were used to prescribe AAI for at least 5 of the 6 absolute indications in children, 90 % of them in adults, and about 50 % of them in all absolute indications in children and adults. According to the French guidelines, 81 % of the allergists were used to prescribe AAI for at least 4 of the 5 absolute indications in children and 90 % in adults, but only 33 % validated all the absolute indications in children, 28 % in adults. History of isolated generalized hive was not considered as an indication by a third of the allergists. 77 % of the allergists were used to prescribe AAI in children with co-existing unstable or moderate-to-severe asthma, 66 % in adults, even if it was not initially considered as an absolute indication in the French guidelines. Food allergy due to traces was an indication for 78 % of the allergists, and adolescence for 16 % of them. In all, there was no significant difference between children and adults. A second AAI was systematically prescribed by the allergists in 34 % of the patients and half of them were used to prescribe a second adrenaline auto-injector for at least one of the 6 suggested indications of the European guidelines. The main criteria for choosing the AAI device were: availability (56 %), ergonomics (38 %), availability of trainers (33 %), needle’s length (22 %).

**Conclusions:** Our findings may be useful to improve knowledge and adherence to the guidelines and to update French guidelines for prescribing AAI.

**References**

1. Commision tripartite de la SFA. Rev Fr Allergol Immunol Clin 2003.
2. Muraro, et al. Allergy 2014.
3. www.allergyvigilance.org.

### PP37 Anaphylaxis by Anacardium Occidentale

####  Marta Viñas^1^, Bartolomé Borja^2^, Nora Hernández^1^, Mª José Castillo^1^, Adriana Izquierdo^1^, Marcel Ibero^1^

##### ^1^Hospital de Terrassa, Terrassa, Spain; ^2^Bial Arístegui, Bilbao, Spain

###### **Correspondence:** Marta Viñas

*Clinical and Translational Allergy* 2016, **6(Suppl 1)**:PP37

**Introduction:** According to our casuistry, nuts are the leading cause of anaphylaxis in children aged from 0 to 14 years.

**Case report:** A 7 year old boy diagnosed in 2011 of extrinsic bronchial asthma due to sensitisation to dog dander/epithelium. In May 2014, after 5–10 min of eating 3 cashews, he presented dyspnea, dysphagia, sweating, and generalized urticaria. We performed him skin prick tests to panallergens (lipid transfer protein, polcalcina and profilin), to commercial extracts of nuts (peanut, hazelnut, almond, walnut, pistachio, pine nuts, sunflower seeds and chestnut) and natural extracts of fried and raw cashew and roasted and raw hazelnut, both degreased and without degreasing and prick by prick with cashew. Analytics with basal tryptase, total IgE and specific IgE by ImmunoCAP to peanut, hazelnut, walnut, cashew and pistachio, and component resolved diagnostics (CRD) with recombinant allergens. Finally, an oral challenge with hazelnuts was performed with good tolerance and SDS-PAGE immunoblotting with cashew. The results of all tests were completely negative. Tryptase baseline: 4.03 mg/L. Total IgE: 339.5 U/l. Specific IgE: hazelnut, peanut, walnut, cashew and pistachio <0.10 kU/L. CRD: rJug r 3, rAra h 2, rAra h 9 and rAra h 8 < 0.10 kU/L. The patient tolerated 20 g of hazelnuts and in the Immunoblotting SDS-PAGE one band corresponding to a protein of about 33 kDa was detected.

**Conclusions:** Basing on the results of the SDS-PAGE Immunoblotting, our patient was diagnosed of anaphylaxis by cashew nuts. The protein detected corresponds to an allergen described of cashew: Ana o 2, which is an 11S globulin reservation. In our patient we have forbidden eating cashew and pistachio, being of the same family (Anacardiaceae) and they have cross-reactivity, but we have allowed the intake of other nuts.

**Consent to publish**

Written informed consent for publication of this clinical details and/or clinical images was obtained from the patient/parent/guardian/relative of the patient. A copy of the consent form is available for review by the Editor of this journal.

### PP38 Anaphylaxis with honey in a child

####  S. Tolga Yavuz^1^, Ali Gungor^2^, Betul Buyuktiryaki^3^, Ozan Koc^2^, Can Naci Kocabas^3^, Faysal Gok^2^

##### ^1^Department of Pediatric Allergy, GATA School of Medicine, Ankara, Turkey; ^2^Department of Pediatrics, GATA School of Medicine, Ankara, Turkey; ^3^Department of Pediatric Allergy and Immunology, Ankara Child Health and Diseases Hematology-Oncology Training and Research Hospital, Ankara, Turkey

###### **Correspondence:** S. Tolga Yavuz

*Clinical and Translational Allergy* 2016, **6(Suppl 1)**:PP38

**Introduction:** Honey is a mixture of flower nectar, pollens, and components from bees. Honey allergy is a rare entity, however it may cause serious reactions in allergic individuals.

**Case report:** A 10-year-old boy admitted to our outpatient department suffering from allergic reactions after consumption of honey. He was under regular follow-up due to asthma and pollen-induced allergic rhinitis for 4 years. At different times, he presented with wheezing, swelling of the lips and eyelids within 5 min after ingestion of honey. His skin prick test with common aeroallergens revealed grass pollen and mold mix sensitivity and prick-to-prick tests with four different types of honey were found to be positive. Specific IgE to honey was also positive. Dietary elimination of honey was suggested and adrenaline auto-injectors were provided.

**Conclusion:** Along with the leading causes of food allergy in childhood such as cow’s milk, egg and peanuts, rare foods such as honey may cause allergic reactions in children. Increase of the awareness by the physicians and the families may help better identification of rare food allergies.

**Consent to publish**

Written informed consent for publication of this clinical details and/or clinical images was obtained from the patient/parent/guardian/relative of the patient. A copy of the consent form is available for review by the Editor of this journal.

### PP39 Evaluation of courses adopted to children on prevention, recognition and management of anaphylaxis

####  Tina Vesel^1^, Mihaela Nahtigal^2^

##### ^1^Department of Allergology, Rheumatology and Clinical Immunology, University Children’s Hospital, University Medical Center, Ljubljana, Slovenia; ^2^General Hospital in Slovenj Gradec, Slovenj Gradec, Slovenia

###### **Correspondence:** Tina Vesel

*Clinical and Translational Allergy* 2016, **6(Suppl 1)**:PP39

**Background:** Training children and teenagers to avoid, recognise and manage anaphylaxis is essential.

**Methods:** 95 food allergic children with prescribed adrenaline auto injectors were invited with their relatives and friends to attenuate courses on anaphylaxis management and families of 21 among them responded invitation. We have applied the questionnaire on avoiding, recognition and management of anaphylaxis to children before and after the 1.5 h long courses on anaphylaxis management.

**Results:** There were 26 participants (20 boys, 6 girls; age 10–18). 20 children had prescribed adrenaline auto injectors because of food allergy, four children were relatives of food allergic child and two children were colleagues of food allergic child. 96 % of children recognised correctly signs of anaphylaxis before and 100 % after the course. Regarding preventive measures of anaphylaxis, the correct position during anaphylaxis and the correct order of management during the anaphylaxis all children answered correctly. 88 % of children would give adrenaline by auto injector during anaphylaxis before the course and all of them after the course.

**Conclusions:** The course enhanced theoretical ability and the willingness of appropriate first-line management of anaphylaxis in children though children were already previously well prepared and motivated to act during anaphylaxis. Other modes of passing knowledge on anaphylaxis management beside courses during schooldays should be sought in order to educate more children.

### PP40 Symptomatic dust mites and shrimp allergy: three pediatric case reports

####  Filipa Almeida, Susana Lopes, Cristina Madureira, Tânia Lopes, Fernanda Carvalho

##### Centro Hospitalar do Médio Ave, Vila Nova de Fa, Portugal

###### **Correspondence:** Filipa Almeida

*Clinical and Translational Allergy* 2016, **6(Suppl 1)**:PP40

**Introduction:** It has been reported that many patients sensitized to inhaled dust mites are also sensitized to shellfish, due largely to the cross-reacting anti-tropomyosin (r Pen a1) IgE. That co-sensitization can also occur in the absence of cross-reactivity and clinical relevance may be different.

**Purpose and methods:** To characterize cases of allergy to shellfish and inhaled dust mites in children followed in outpatient Level II Hospital evaluating their sensitization profile and occurrence of any cross-reaction. Data were collected on shrimp, inhaled dust mites (*dermatophagoides pteronyssinus* and *dermatophagoides farinae*) and tropomyosin sensitization based on clinical history, skin prick tests (SPTs) (Leti^®^) or specific serum IgE (ImmunoCap^®^).

**Results:** Three cases were selected, all male. There was a family history of allergy in 2 cases. In those the allergy to shrimp was the first manifestation of atopic disease. The age of diagnosis of shrimp allergy varied between 8 and 10 years, and clinical presentations were: anaphylaxis, urticaria, angioedema and pharyngeal itching. All cases had allergic rhinitis to inhaled dust mites. One case also had asthma and co-sensitization to grass. All cases had co-sensitization with other food allergen (crustacean and mollusk). The specific serum IgE to shrimp and tropomyosin (r Pen a1) was positive in two cases (one case with a positive skin prick test to shrimp is waiting for the result). In 2 cases there was previous history of persistent rhinitis but the symptoms were not valued.

**Conclusion:** The authors present 3 cases of allergy to shrimp and inhaled dust mites with different clinical presentations. In all cases the rhinitis was the main atopic disease though often neglected. The presence of other food allergies to shellfish group is in favor of cross-reactivity obtained by the panallergen tropomyosin.

### PP41 Poor identification rates of nuts by high risk individuals: a call for improved education and support for families

####  Camille Heming^1*^, Emily Garrett^1*^, Adam Blackstock^1*^, Santanu Maity^2^, Rahul Chodhari^2^

##### ^1^Medical School, University College London, London, United Kingdom; ^2^The Royal Free NHS London Foundation Trust, London, United Kingdom

###### **Correspondence:** Camille Heming

*Co-first authors: Heming, Camille; Garrett, Emily; Blackstock, Adam.

*Clinical and Translational Allergy* 2016, **6(Suppl 1)**:PP41

**Background:** London is home to 170 nationalities and 300 spoken languages. Many allergic families have experienced difficulty implementing nut avoidance advice due to language barriers. This pilot investigates ability of allergic children, families and healthcare professionals to identify nuts to guide production of a targeted educational resource.

**Method:** 12 “nut playing cards” displaying large photos of common nuts were shown to families in a tertiary pediatric allergy clinic and to General Practitioners. Responses were recorded.

**Results:**

*Children and parents:*

- 95 participants aged 6–62 (15 different languages spoken).
- Only 1/5 participants correctly identified all nuts.
- Average nut identification rate—67 % in English-speaking families Vs 44 % in non-English speaking families.
- Only 30 % of subjects that were born outside the UK and did not speak English at home identified >6 nuts.
- Only 50 % of families speaking languages other English identified >1/3 of nuts.
- School-going age (5–18 years) identified fewest nuts correctly, averaging 5/12, compared to 9/12 in adults. Data is consistent with US findings summarised in 2006 and 2011 papers.
- Poor scoring correlated with lower socioeconomic classes.
- Only 30 % of subjects with a known nut allergy were able to correctly identify >6/12 nuts.

*Healthcare Professionals:*

**Fig. 3:**
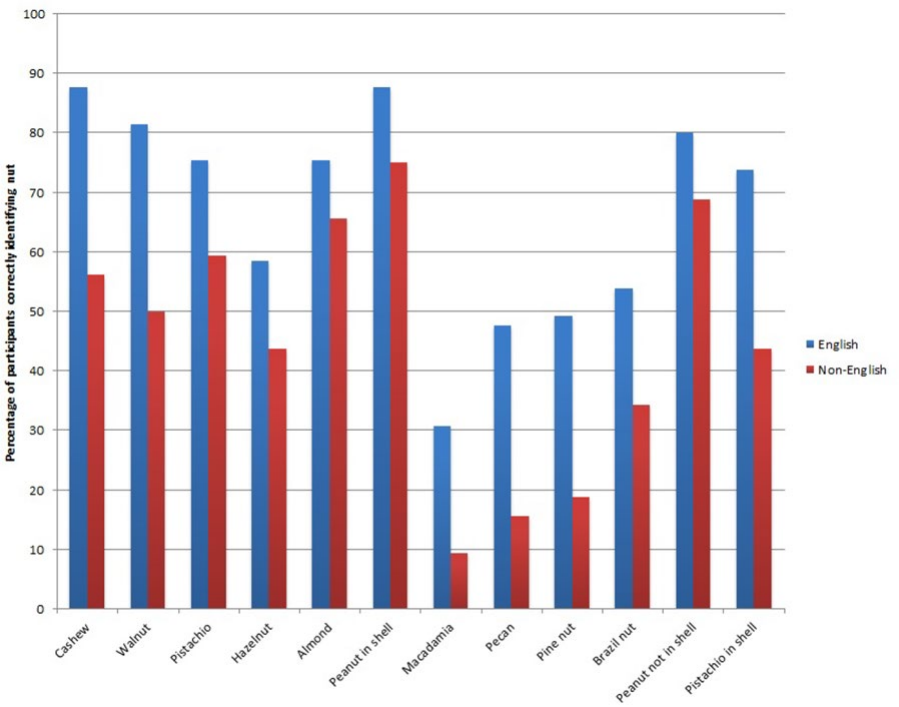
The effect of language spoken at home on ability to correctly identify nuts

- GPs identified an average of 10/12 nuts.
- 82 % of GPs felt that parents of children with nut allergies are most concerned about anaphylaxis and death.
- GPs think effective management of nut allergy is limited by language barriers, lack of education and misunderstanding of patients and health professionals, and time constraints (Fig. 3).

**Conclusion:**

- Visual identification was particularly unreliable in younger individuals, individuals of low socioeconomic class, or those who do not speak English at home.
- This pilot highlights an opportunity to develop multi-lingual educational resources such as online platforms and school programmes to support children and families from all backgrounds and ethnicities.

### PP42 DAFALL: database of food allergies in the Czech Republic

#### Simona Belohlavkova^1^, Eliska Kopelentova^2^, Petr Visek^3^, Ivana Setinova^4^, Ivana Svarcova^1^

##### ^1^Immuno-flow s.r.o., Na Homolce Hospital, Prague, Czech Republic; ^2^Kolin Hospital, Faculty Hospital Motol, Prague, Czech Republic; ^3^Allergology Litomysl, Litomysl, Czech Republic; ^4^Immunia, Prague, Czech Republic

###### **Correspondence:** Simona Belohlavkova

*Clinical and Translational Allergy* 2016, **6(Suppl 1)**:PP42

**Background:** Occurrence of food allergy has significantly risen in recent years and its prevalence is 6–8 % in children and 3–4 % in adults. There are some differences in incidence of types of food allergy in different areas. Most commonly implicated foods in childhood are milk, egg, nuts, wheat, soy, fish and shellfish worldwide. There are some differences in incidence of types of food allergy in different areas. Only limited data are known about the most common causes of food allergy in Czech Republic.

**Methods:** DAFALL-Datase of Food Allergies is an electronic registry founded in 2014 aiming to gather epidemiological data describing food allergy in the Czech Republic. Most common triggers of food reactions, threshold doses, processing of food allergens, laboratory test results including component resolved diagnosis, skin prick tests and food challenges results and also allergology history of the patients were evaluated. Special attention was devoted to patients with food anaphylaxis.

**Results:** During the first 5 months until April 2015, 250 patients were enrolled from more than 20 collaborating allergology clinics, 58 % children under age of 5 years, 24 % children aged 6–18 years and 18 % adults. In children under 1 year of age, cow’s milk is the most frequent food allergen 87 %). In 90 % of cases, first symptoms of milk allergy were recorded below age of 7 months and in 60 % of cases were noted in fully breast-fed infants. More than 50 % of milk reactions were non-IgE mediated, with no prove of any positivity in skin prick tests and/or specific IgE against milk. Most common triggers of allergy in children under age of 5 years were milk, egg, tree nuts, fruits and peanuts. In patients offer 6 years were fruits, tree nuts, peanuts and vegetables the most common triggers. In patients older than 6 years, significant allergens are also the seeds, mainly the sesame seed and poppy seed.

**Conclusion:** DAFALL is the first project in the Czech Republic describing relevant data on food allergy in the Czech population. Projected period for data collection is 3 years and we expect to enroll 1500–2000 patients from 30 collaborating centers.

### PP43 Serological cross-reactivity between grass and wheat is not only caused by profilins and CCDs

####  Sigrid Sjölander^1^, Nora Nilsson^2^; Malin Berthold^1^, Helena Ekoff^1^, Gunilla Hedlin^2^, Magnus Borres^1^, Caroline Nilsson^3^

##### ^1^Thermofisher Scientific, ImmunoDiagnostics, Uppsala, Sweden; ^2^Karolinska Institutet and Karolinska University Hospital and Center for Allergy Research, Stockholm, Sweden; ^3^Södersjukhuset, Karolinska Institutet and Sachs Children’s Hospital, Stockholm, Sweden

###### **Correspondence:** Sigrid Sjölander

*Clinical and Translational Allergy* 2016, **6(Suppl 1)**:PP43

**Background:** Sensitization to wheat is common among patients with grass pollen allergy but is not always associated with symptoms to wheat. It is often unclear whether the reactivity reflects a wheat specific sensitization or cross-reactivity with pollen allergens. Two cross-reacting allergens are known, profilins and cross-reacting carbohydrate determinants (CCDs).

**Aim:** To study the association between IgE sensitization to grass and wheat among wheat tolerant children with a diagnosis of grass pollen allergy.

**Materials and methods:** Seventy-two Swedish children (0–18 year) with a doctor’s diagnosis of grass pollen allergy, IgE antibody (IgE-ab) responses to grass and currently eating wheat were included. Serum analyses of IgE-ab to wheat, timothy and related components were performed. Inhibition with timothy and/or wheat extract was performed on sera displaying IgE responses to both wheat and timothy.

**Results:** All children had IgE-ab to timothy with a median level of 20 kU_A_/l, while 44 children were sensitized to wheat with a median level of 1,2 kU_A_/l. Inhibition with timothy extract was performed on 20 sera before analyzing the remaining IgE reactivity to wheat. In some sera the reactivity was completely inhibited while other sera, to different degrees, retained their IgE reactivity to wheat. Five children, displaying reactivity to both timothy and wheat, had IgE-ab binding to CCD (7 %) and 12 to timothy profiling, Phl p 12 (17 %). Inhibition of two sera with timothy extract the reduced the binding to CCD and wheat by >93 % and >86 %, respectively. Inhibition of three sera with wheat extract reduced the binding to Phl p 12 and timothy by >83 % and 7–20 %, respectively.

**Conclusions:** Sixty percent of the wheat tolerant children with grass pollen allergy were also sensitized to wheat, although the level of IgE-ab was significantly lower than that to grass. In the wheat sensitized children the reactivity to wheat could neither be entirely explained by cross-reactivity with timothy nor by cross-reactive responses to CCD or profilins. Thus, further cross-reacting allergens remains to be identified and/or co-sensitization to wheat needs to be considered.

### PP44 Oil body associated proteins in children with nuts allergy. Allergens to consider in IgE-mediated nuts allergy

####  Loreto González Domínguez^1^, Cristina Muñoz Archidona^2^, Ana Moreira Jorge^1^, Sergio Quevedo Teruel^1^, Teresa Bracamonte Bermejo^1^, Miriam Castillo Fernández^3^, Fernando Pineda de la Losa^3^, Luis Ángel Echeverría Zudaire^1^

##### ^1^Hospital Universitario Severo Ochoa, Madrid, Spain; ^2^Hospital de Villalba, Madrid, Spain; ^3^Application Department Diater, Madrid, Spain

###### **Correspondence:** Cristina Muñoz Archidona

*Clinical and Translational Allergy* 2016, **6(Suppl 1)**:PP44

**Background:** Oil body associated proteins (OAPs)’s role in nuts allergy is still poorly known. Conventional skin prick test (SPT) reagents determine mainly water soluble proteins (WPT) as well as it happens with allergen-specific IgE antibodies (sIgE), so OAPs are not well detected. We study the sensitization profile in children with suspected nuts allergy.

**Methods:** We evaluated children (median: 5 years old) with suspected allergies to nuts. WPT and OAPs of peanut, almond, hazelnut and walnut were determined through SPT and sIgE.

**Results:** Peanut suspected allergy: 31 patients. WPT’s SPT was positive in 74.2 % and OAPs SPT in 45.2 % of those patients. WPT-sIgE was positive (>0.35 kU/l) in 80 % (Median 2.29kU/l) and OAP-sIgE was positive (>0.1 kU/l) in 64.5 % (Median 0.66kU/l).

*Almond suspected allergy:* 9 patients. WPT-SPT positive: 33.3 %, OAPs-SPT positive: 66.7 %. WPT-sIgE positive: 62.5 % (Median 1.51kU/l), OAP-sIgE positive: 55.6 % (Median 0.11 kU/l).

*Hazelnut suspected allergy:* 23 patients. WPT-SPT positive: 87 %, OAPs-SPT positive: 52.2 %. WPT-sIgE positive: 90.9 % (Median 2.84kU/l), OAP-sIgE positive: 78.3 % (Median 0.27 kU/l).

*Walnut suspected allergy:* 30 patients. WPT-SPT positive: 70 %, OAPs-SPT positive: 60 %. WPT-sIgE positive: 86.7 % (Median 3.5kU/l), OAP-sIgE positive: 83.3 % (Median 0.61 kU/l).

They were a total of 69 patients. Of them, 50 had at least one OAPs-SPT positive and 39 were sensitized to more than 1 nut, being mainly involved the walnut (33 patients), followed by hazelnut (25 patients). No differences were found in reaction’s severity in terms of OAPs sensitization.

**Conclusions:** A considerable number of patients had any of OAPs tests positive (SPT or sIgE). Among these children, 78 % were sensitized to several nuts, being the walnut the most common. No relationship between sensitization to OAPs and reaction’s severity was found.

### PP45

**WITHDRAWN**

*Clinical and Translational Allergy* 2016, **6(Suppl 1)**:PP45

### PP46 Protective effect of helicobacter pylori infection against food allergy in children

####  Olga Vrani^1^, Antigone Mavroudi^1^, Maria Fotoulaki^2^, Maria Emporiadou^2^, Kleomenis Spiroglou^3^, Ioannis Xinias^1^

##### ^1^Aristotle University of Thessaloniki, Hippokrateio General Hospital, Thessaloniki, Greece; ^2^Aristotle University of Thessaloniki, Papageorgiou General Hospital, Thessaloniki, Greece; ^3^Aristotle University of Thessaloniki, Thessaloniki, Greece

###### **Correspondence:** Olga Vrani

*Clinical and Translational Allergy* 2016, **6(Suppl 1)**:PP46

The published version of this abstract can be found at [1].

**Reference**

1. Allergy. 2015;70(Suppl 101):614–49. http://onlinelibrary.wiley.com/doi/10.1111/all.12724/epdf.

### PP47 Anaphylaxis pathway: A road tryp-tase to success?

#### Helyeh A. Sadreddini, Mia Warnes, Donna Traves

##### Derbyshire Children’s Hospital, Royal Derby Hospital, Nottingham, United Kingdom

###### **Correspondence:** Helyeh A. Sadreddini

*Clinical and Translational Allergy* 2016, **6(Suppl 1)**:PP47

**Introduction:** Anaphylaxis is a life threatening condition with a UK incidence increasing by over 6 fold between 1992 and 2012. Our hospital provides an acute pediatric service to a population of over 110,000 children within Southern Derbyshire.

**Aims:** To assess the compliance of anaphylaxis management in children presenting to our Emergency Department against local and national guidance.

**Methods:** We retrospectively reviewed Emergency Department attendances over a 2-year period from April 2012-March 2014. Clinical records with a discharge code of “anaphylaxis” were examined by two independent doctors to ensure diagnosis consensus.

**Fig. 4:**
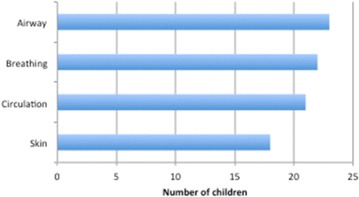
Completed documentation of features in an acute reaction

**Results:** A total of 24 cases were identified. Features of the acute reaction were well documented (Fig. 4). The circumstances around the reaction were not documented in 7 cases and the time of the onset not documented in 22. Nuts were a trigger in 2 children.

**Fig. 5:**
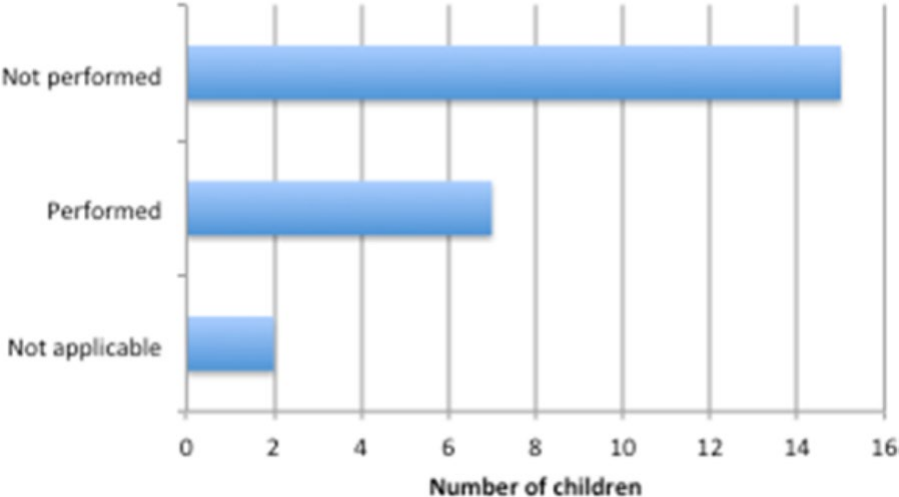
Initial pharmacological management initiated

**Fig. 6:**
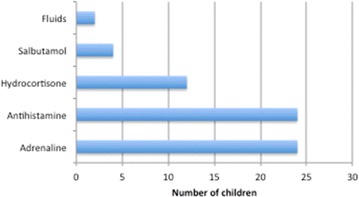
Initial serum mast cell tryptase measurements

Pharmacological management is illustrated in Fig. 5. An initial serum mast cell tryptase level was only taken in 4 of the children, no repeat samples were obtained (Fig. 6).

17 children were given an epipen of which 14 parents were trained in its use. 4 additional children were identified as requiring an epipen. 10 children received information about the signs and symptoms of an allergic reaction and what to do in this instance. No parents were advised about the risk of a biphasic reaction.

**Conclusions and discussions:** Although standards are high with regards to the acute management of anaphylaxis areas for improvement include documenting the specific circumstances of the reaction as well as the time of onset. This will help to identify likely triggers and improve allergen avoidance advice. Moreover this will help the attending doctor to decide when a serum mast cell tryptase level should be performed. This will assist in the longer term management of children presenting with anaphylaxis.

Overall there is good adherence to published guidance however simple measures including the introduction of a local anaphylaxis guideline, patient information leaflets and personal action plans will be implemented. We plan to re-audit in 2016.

## POSTER VIEWING SESSION 3: Miscellaneous (PP48–PP58)

### PP48 Surveillance study on safety of SLIT in pediatric population

#### Ivana Djuric-Filipovic, Zorica Zivkovic, Snežana Zivanovic, Gordana Kostić, Đorđe Filipovic

##### Faculty of Medical Science, Kragujevac, Serbia

###### **Correspondence:** Ivana Djuric-Filipovic

*Clinical and Translational Allergy* 2016, **6(Suppl 1)**:PP48

The published version of this abstract can be found at [1].

**Reference**

1. Allergy. 1999;54(10):1110–1113 (http://onlinelibrary.wiley.com/doi/10.1034/j.1398-9995.1999.00267.x/full).

### PP49 Efficacy and safety of mixed mite subcutaneous immunotherapy among allergic rhinitis patients in the Northeastern Thailand

####  Sawapon Sittisomwong^1^, Siripong Sittisomwong^2^

##### ^1^Allergy Unit, Khonkaen Ram Hospital, Khonkaen, Thailand; ^2^Department of Oral and Maxillofacial Surgery, KKU, Khonkaen, Thailand

###### **Correspondence:** Sawapon Sittisomwong

*Clinical and Translational Allergy* 2016, **6(Suppl 1)**:PP49

**Introduction:** Dust mites are the highest prevalence of allergen sensitization in the north-eastern Thailand. Subcutaneous specific immunotherapy (SCIT) is considered as a standard and effective treatment of respiratory allergies. However, there have been no data reported on clinical efficacy of mixed mite SCIT in the north-eastern of Thailand.

**Aim:** To evaluate the clinical efficacy of mixed *Dermatophagoides pterronyssinus (Der p)* and *Dermatophagoides farinae (Der f)* SCIT (ALK-Abellò) in allergic rhinitis (AR) patients visited Khonkaen Ram hospital.

**Method:** We performed a 3 years prospective descriptive study to evaluate the clinical efficacy of mixed *Der p* and *Der f* SCIT (ALK-Abellò) in AR patients visited Khonkaen Ram hospital. Fifteen patients (9 male and 6 female, age 5.33–37.33 years, median age = 14.75 years) with AR due to *Der p* and *Der f* were enrolled in the study. Main outcome of the study was the rhinoconjunctivitis symptom score (RSS). RSS was evaluated every year after SCIT in relation with the pre-treatment period in which patients suffered the highest symptomatic levels.

**Results:** The RSS was 11.26 ± 1.03 (mean ± SD) in the pre-treatment period and decreased to 6.13 ± 2.35 in the first year follow up period (p < 0.0001) and 1.93 ± 1.48 in the third year follow up period.

**Conclusion:** This study revealed that our mixed mite SCIT regimen decreased the RSS in patients with intermittent and persistent AR indicating the effectiveness of the north-eastern mixed mite SCIT regimen of Khonkaen Ram hospital.

### PP50 Effect of inhaled beclomethasone or placebo on brain stem activity in a patient chronically treated with steroids: preliminary report

####  Zygmunt Podolec^1^, Marcin Hartel^2^, Daria Panek^1^, Magdalena Podolec-Rubiś^1^, Tomasz Banasik^2^

##### ^1^Department of Psychoneuropharmacology, Centre for Research and Development MEDINET, Krakow, Poland; ^2^Department and Laboratory of Magnetic Resonance, Voxel SA, Krakow, Poland

###### **Correspondence:** Zygmunt Podolec

*Clinical and Translational Allergy* 2016, **6(Suppl 1)**:PP50

**Method:** The study was conducted in women [aged 37] treated with steroids because of asthma in order to assess the activity of the brainstem. The study was conducted with the fMRI reader [3T GE] after the administration of beclomethasone (BC) at 250, 500 and 1000 µg or placebo. BC and placebo were administered every 14 days. To ensure the deposition of aerosol in the central-(CAD) or peripheral-(PAD) airways, applied inhalation chamber integrated with a spirometer [PNEUMOlogic^®^ abcMED] was used. Data analysis was performed using FSL, ImageJ integrated program of anatomical atlases. The data were averaged over intervals of 5 min. The first 0–5 min interval taken as reference. Changing the strength of the signal was calculated in standard deviation units for each subsequent 5 min period.

**Fig. 7:**
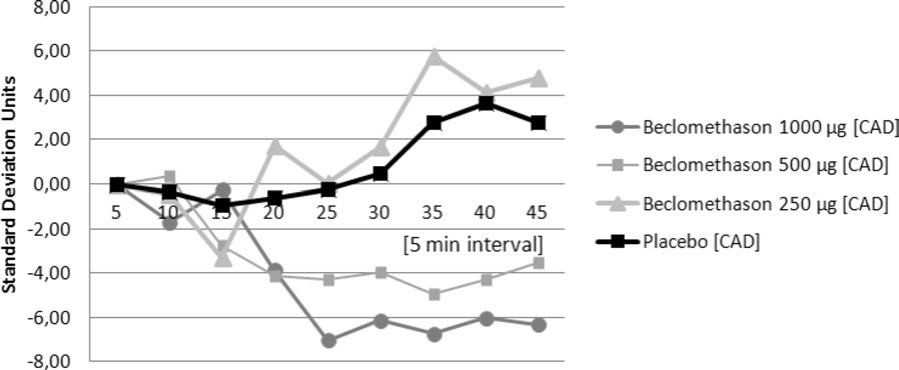
The effect of the dose of beclomethasone [BC] (Cortare TEVA) or placebo and aerosol deposition adventage in central airways [CAD] on the activity of the brain stem

**Results:** After inhalation, with a predominance of central deposition (CAD) placebo and 250 µg BC showed an increase in signal, whereas 500 and 1000 µg of BC was found dose-dependent decrease in the signal (Fig. 7).

**Discussion:** This is the first study to assess the impact of dose and space BC deposition on the function of selected brain structures. The study may be important for understanding the role of the knowledge of the brain and the need to harmonize the treatment of asthma with the brain.

### PP51 Sensitisation to aeroallergens in patients with allergic rhinitis, asthma and atopic dermatitis in Shiraz, Southwestern Iran

####  Elham Abbasi, Mozhgan Moghtaderi

##### Allergy Research Center, Shiraz University of Medical Sciences, Shiraz, Iran

###### **Correspondence:** Mozhgan Moghtaderi

*Clinical and Translational Allergy* 2016, **6(Suppl 1)**:PP51

**Background:** Aeroallergens as one of the most common cause of allergic disease derived from pollens, dust mites, fungi, cockroach and animals. The prevalence of aeroallergens is different in various areas. This study was designed to identify the frequency of sensitization to aeroallergens in patients with asthma, allergic rhinitis and atopic eczema in Shiraz, southwestern Iran.

**Methods:** Six hundred and fifty-six patients with allergy were included in this cross-sectional study from southwestern Iran during 2014. Sensitization to aeroallergens was assessed by skin prick test using a panel of common 15 aeroallergens in studied patients.

**Results:** A positive skin test to at least one of the applied allergens was seen in 74.5 % of our patients. The female to male ratio and mean age of the patients were 1.27 and 27.6 ± 14.7 years, respectively. Pollens were the most common type of aeroallergens (64.6 %), followed by dust mites (34.6 %), cockroach (30.6 %), molds and cat hair (each 16 %). Among pollens, the frequency of sensitization to weeds, grasses and trees was in turn.

**Conclusion:** The results of the present study revealed that pollens play a main sensitizing allergen in asthma, allergic rhinitis and eczema. This pattern was compatible with the results from studies carried out in this area.

### PP52 Referring a child for allergy test: how appropriate are we?

####  Phani Sanneerappa^1^, Alina Deliu^1^, Moosa Kutty^1^, Nagabathula Ramesh^2^

##### ^1^Letterkenny General Hospital, Letterkenny, Ireland; ^2^Midland Regional Hospital, Portlaoise, Ireland

###### **Correspondence:** Phani Sanneerappa

*Clinical and Translational Allergy* 2016, **6(Suppl 1)**:PP52

**Aim:** To study the epidemiology and appropriateness of referral of allergy tests performed in a peripheral hospital in Republic of Ireland

**Materials and methods:** Data was gathered retrospectively in all children up to 14 years of age, who had allergy tests (Total IgE and specific IgE) done between periods 1st January 2014 to 30th June 2014. Specific IgE values more than 0.35 were considered positive. Data was tabulated with variables of age, mode of delivery, associated asthma or eczema, family history of asthma or eczema, any anaphylaxis reaction and was NICE guidelines followed or not. Data was analysed and conclusions were drawn.

**Results:** 297 allergy tests were performed in the six month period. 154(51.8 %) of 297 had positive specific IgE values. 44.44 % had positive IgE. 5.8 % were performed under 1 year of age, 40.1 % performed between 1–5 years and 62.4 % performed over 5 years. 27 % born by section, 59.2 % by vaginal delivery and 13.8 % data was not available. 46.08 % had positive family history. 46.08 % has associated eczema and 55.68 % had associated asthma. 52.84 % followed NICE guidelines for performing the allergy test.

**Conclusion:** A vast number of children are subjected to allergy tests and only 52.84 % met NICE guidelines. As the cost of each test was Total IgE = €37.08, Basic RAST = €38.70, IgE and RAST = €75.78, Each individual separate request ranges from €4.30 to €27.54 (depending on allergen) on top of the €75.78, huge savings of about 22430.88 Euros could be saved per year. Appropriate training should be considered for all physicians who order allergy tests. By following NICE guidelines and performing alternate testing methods like skin prick tests, we can improve the health economics of Letterkenny hospital.

### PP53 EBV lymphoproliferative disease and cardiac lymphoma in a STK4 deficient patient

####  Roya Sherkat^1^, Mohammad Reza Sabri^2^, Bahar Dehghan^2^, Hamid Bigdelian^3^, Nahid Raeesi^2^, Mino Afshar^4^, Hamid Rahimi^2^, Christoph Klein^5^

##### ^1^Acquired Immunodeficiency Research Center, Isfahan University of Medical Sciences, Isfahan, Iran; ^2^Pediatric Department, Isfahan University of Medical Sciences, Isfahan, Iran; 3Heart Surgery Department, Isfahan University of Medical Sciences, Isfahan, Iran; ^4^Pathology Department, Isfahan University of Medical Science, Isfahan, Iran; ^5^Pediatric Department, Dr. von Hauner Children’s Hospital, Ludwig Maximilians-University, Munich, Germany

###### **Correspondence:** Roya Sherkat

*Clinical and Translational Allergy* 2016, **6(Suppl 1)**:PP53

**Introduction**: The infection of B-cells by EBV provokes a marked activation of immunoregulatory T-cells and requires restoration of immune homeostasis during convalescence. Therefore patients with immune defects resulting in impaired T-lymphocytes failed to destroy EBV-infected cells and may develop to sever lymphoproliferative disorders and lymphomas. STK4 plays an important role in activation of transcription factors involved in T cell homeostasis and response to chronic viral infections.

**Case**: We are presenting an eleven-year-old girl of consanguine family with chest pain and shortness of breath that has been diagnosed as STK4 deficiency, as a novel gene since the age of eight. She had history of neutropenia, recurrent skin abscesses, mouth ulcers, warts, molloscum contagiusum, herpetic lesions, recurrent fever, bacterial and fungal respiratory infections and positive family history of Stk4 deficiency in her aunt and uncle. She suffered from persistent fever and generalized lymphadenopathy which was reported as EBV-associated B-lymphoproliferative disorder in lymph node biopsy. Afterwards, she presented with cardiomegaly with no mediastinal involvement, pericardial effusion and cystic like echogenic masses on inter atrial septum which has been progressed. After few missed follow up visits, she has been admitted to the cardiology emergency with respiratory distress and syncope which required an emergency cardiac surgery. The excised specimen was positive for ICA, CD30, Ki67, EBV and EMA, suggesting the diagnosis of “T-cell non-Hodgkin’s Lymphoma”.

**Conclusion**: Primary cardiac lymphomas (PCL) account for about 1 % of the primary cardiac malignancies and 0.5 % of the extranodal lymphomas while disseminated lymphoma with cardiac involvement can occur in up to 20 % of lymphomas. PCL occurs more frequently in immunocompromised patients. There was no report of cardiac lymphoma associated with STK4 deficiency till now. Another study investigating 4 patients with STK4 deficiency, found development of EBV-associated lymphoproliferative syndrome and Hodgkin lymphoma in 2 and 1 patients respectively.

**Consent to publish**

Written informed consent for publication of this clinical details and/or clinical images was obtained from the patient/parent/guardian/relative of the patient. A copy of the consent form is available for review by the Editor of this journal.

### PP54 A case study: the effect of massive honeybees attack on various body parameters atopic girl including allergy

####  Mohemid Al-Jebouri

##### College of Medicine, University of Tikrit, Tikrit, Iraq

###### **Correspondence:** Mohemid Al-Jebouri

*Clinical and Translational Allergy* 2016, **6(Suppl 1)**:PP54

**Background:** The use of bee venom as a medicine is nothing new. The ancient Iraqi^′^s and Egyptians were the first known civilization to try bee venom. The emperor Charlemagne and Ivan the Terrible used bee venom to help alleviate sore joints and stiffness, according to “Medicinal Uses for Bee Venom” at Swedish.org, Today the medical uses of bee venom remain slightly clouded with evidence both for and against its medicinal benefits.

**Methods:** An 18 years old Iraqi girl who is atopic type was massively attacked by honeybees. She directly attended the nearest hospital suffering from severe allergy and asthma. Blood sample was drawn. Several biochemical, immunological and haematological tests were done for this patients. Urea, creatinine, transaminases, total bilirubin, alkaline phosphatase, uric acid, serum sodium, serum chloride and creatine kinase were carried out according to manufacturer instructions. Blood film were examined to differentiate different leukocytes. IgE, IgG.IL-I beta, IL-6 and TNF cytokines were assessed. Phospholipase A(2) was demonstrated.

**Results:** It was found that most of the biochemical parameters concerned were elevated. IgE, IgG, cytokines and phospholipase A(2) were increased. Eosinophils and basophils were elevated as well haemoglobin. It was shown that this girls was suffering from acute asthma and breathing difficulty. Skin and her eyes were red with oedema. The patient was recovered due to management by cortisone and antihistamine drugs as she is atopic type suffered from severe allergy of skin and asthma with very difficult breathing due to bee strings.

**Conclusions:** Bee stings could be very dangerous and fatal especially for the allergic individuals. The complications might involve different organs of the victim particularly the respiratory system, liver and kidneys.

**Consent to publish**

Written informed consent for publication of this clinical details and/or clinical images was obtained from the patient/parent/guardian/relative of the patient. A copy of the consent form is available for review by the Editor of this journal.

### PP55 The role of TLR9, NLRP3 and proIL-1β in activation of antiviral innate immunity

####  Oxana A. Svitich^1^, Daria O. Zubacheva^1^, Dmitrii A. Potemkin^1^, Ludmila V. Gankovskaya^2^, Vitalii V. Zverev^1^

##### ^1^Mechnikov Research Institute of Vaccines and Sera, Moscow, Russia; ^2^Russian National Research Medical University, Moscow, Russia

###### **Correspondence:** Oxana A. Svitich

*Clinical and Translational Allergy* 2016, **6(Suppl 1)**:PP55

**Background:** It is known, that during viral infection DNA or RNA molecules are detected in the cytoplasm of infected cells. The most important signaling pathways, taken part in the DDR (DNA damage response) are: cGAS-STING, Rad50-Card9. The result of activation of these and TLR signaling pathways is secretion of proinflammatory cytokines (IL-1β, IL-6, TNF), IFN, chemokines, AMP etc.

The aim is to investigate expression of genes of TLR9, NLRP3 and proIL-1β genes, which play role in detection of viral infection and activation of innate immunity.

**Methods:** In our work we used the cell culture Vero. In experiments of activation TLR9-mediated signaling pathways we used 48 h Vero cells and CpG-rich DNA antigens (Sintol, RF) in concentration 10, 1, 0.1 pg/ml. We use «Ribosorb» (ILS, RF), set for reverse transcription and PCR (Sintol, RF) for the detection of gene expression. To investigate the blocking of signalling pathways of innate immunity we use the siRNA-interference technology. The cells Vero were transfected by siRNA (Sintol, RF). We discover the CpG-rich DNA antigens-dependent expression of TLR9 increased in 46(10 pg/ml), 26 (1 pg/ml), 4 (0.1 pg/ml) times.

**Results:** The results in experiment with knockdown of components of signaling pathways of antiviral cytokine IL-1b demonstrated link between NLRP3 expression and IL-1b secretion. The transfection of NLRP3’s gene siRNA lead to decreasing of the level of NLRP3 expression in 1129 times and pro-IL-1b expression in 288 times.

**Conclusions**: So, CpG-rich DNA activates the TLR9 expression, that plays important role in viral DNA detection. The block of NLRP3 gene expression leads to decreasing of pro-IL-1b expression in 288 times. In future it is important to investigate more properly the mechanisms and signaling pathways of cell antiviral immunity.

### PP56 Overnight pulse oximetry, as a screening tool to diagnose obstructive sleep apnoea. How effective is it?

####  Phani Sanneerappa; Elaine OB Doyle; Paul Gallagher; Nagabathula Ramesh

##### Midland Regional Hospital, Portlaoise, Ireland

###### **Correspondence:** Phani Sanneerappa

*Clinical and Translational Allergy* 2016, **6(Suppl 1)**:PP56

**Background:** Overnight pulse oximetry is a one of the new investigative modalities performed in the home environment to diagnose Obstructive Sleep Apnoea (OSA). Research has demonstrated that home sleep tests could be an accurate method of identifying patients with OSA.

**Aim:** To study the efficacy of Overnight pulse oximetry as a screening and referring tool for obstructive sleep apnoea in a peripheral hospital in Republic of Ireland.

**Methods:** Children aged between 3 months and 14 years, clinically suspected to have moderate to severe obstructive sleep apnoea from June 2013 to June 2015 were included in the study. Acute Pediatric link nurse receiving a referral would educate the parents and the child regarding the procedure of overnight pulse oximetry. Pulse oximetry readings, an average from 3 consecutive nights were recorded. Results were interpreted from Working party on sleep physiology and respiratory control disorders in childhood September 2009. Appropriate referrals to tertiary centres were done either to otorhinolaryngology or pulmonologist.

**Results:** A total of 31 were referred for Overnight pulse oximetry. The study was performed on 28 (90.32 %). None of the children experienced difficulty in recording at home. In all cases the study report matched the clinical indicators of suspected apnoea. In 100 % of the cases, pulse oximetry was found to be positive and which displays the clinical appropriateness of referrals.

**Conclusion:** Overnight Pulse oximetry has proved be a definite screening tool to diagnose Obstructive sleep apnoea. As 100 % positive results are obtained, we feel more studies are needed to study the population with extended inclusion criteria. It has definitely helped to conduct appropriate referral to various specialities, thus improving the waiting time and decreased the inappropriateness in referrals.

### PP57 The presentation and management of acute urticaria and allergic reactions in children in a multi-ethnic, inner city Emergency Department (ED)

####  Sherine Dewlett, Kin Man, Minal Gandhi, James Pocock, Anna Gerrardhughes

##### Royal Free Hospital, London, United Kingdom

###### **Correspondence:** Sherine Dewlett

*Clinical and Translational Allergy* 2016, **6(Suppl 1)**:PP57

**Introduction:** Urticarial rash and allergic reactions are common symptoms in children presenting to the emergency department (ED) and vary geographically.

**Aim:** To describe the characteristics and management of children presenting to a London Teaching hospital ED with urticaria and allergic reactions to identify areas of focus for improvement and aid current guideline implementation.

**Method:** Retrospective review of electronic notes of 58 children (aged < 17 years), presenting to ED from March- June 2015 with diagnosis “allergic reaction”, “urticaria”, “urticarial rash” and “anaphylaxis”.

**Results:** 58 children over 3 m, 1.3 % of total children seen in the ED. Median age 5 years, M: 48 %, 34 % known allergy or atopy. 90 % first presentation, 4 (7 %) had anaphylaxis, 33 (56 %) had urticaria alone. Suspected triggers Food: 19 (32 %) (commonest nuts), Idiopathic 14 (24 %), Virus 12 (21 %), Drugs 12 (21 %) (mainly antibiotics), Other 1(2 %).

*Anaphylaxis management*: Adrenaline Auto-injector (AAI) not used, no written plans and no AAI on discharge but allergy follow up arranged.

*Drugs given*: Antihistamine 72 %, Steroids 22 % (variable indication).

Follow up arranged in most cases of suspected food allergy 13/19, few of drug allergy 1/12.

**Limitations:** The study is limited by size and by specific search criteria probably leading to an underestimate of incidence of allergic presentations.

**Conclusion:** Urticaria and allergic reactions account for at least 1.3 % of children seen in the ED. Anaphylaxis accounted for 7 % of these. Food especially nuts were the commonest trigger. Antibiotics are the commonest drugs implicated; however over reporting due to intercurrent infections also being a trigger may occur. Few of these are referred for formal testing with implications for future management of infection. Management is poor with respect to variable use of steroids, use and prescription of AAI and written action plans. Future educational interventions and guideline implementation in the ED will target these issues.

### PP58 Food allergens responsible for delayed-type sensitisation in atopy patch test in children diagnosed with autism spectrum disorder

####  Jolanta Wasilewska, Maciej Kaczmarski, Dariusz Lebensztejn

##### Department of Pediatrics, Gastroenterology and Allergology, Medical University of Bialystok, Bialystok, Poland

###### **Correspondence:** Jolanta Wasilewska

*Clinical and Translational Allergy* 2016, **6(Suppl 1)**:PP58

**Introduction:** Casein-free and gluten-free (CFGF) diet has been considered a potentially beneficial dietetic intervention of children diagnosed with autism spectrum disorder (ASD). IgE mediated cow’s milk allergy and wheat allergy have not been found to be more common in children with autism as compared to the general population. In the current study, we evaluated delayed-type sensitization to cow’s milk, wheat, gliadin and other common food allergens in children diagnosed with ASD.

**Materials and methods:** Atopy Patch Tests with food allergens were performed in 103 children, including 47 children diagnosed with autism (median 4.7 years), 29 with IgE mediated food allergy (median 4.6 years) and in 27 children without IgE food allergy (median 4.2 years). Patients of the Consultant Allergy Clinic of Children’s University Hospital in Białystok, aged 2–10 years, were included in the study between April 2009 and May 2012. A Chi square test was used for analysis.

**Results:** Delayed skin response to gliadin was found in 11 of 47 (23.4 %) children with autism, in 1 of 29 (3.45 %) children with food allergy and in 0 of 27 (0.0 %) children without IgE food allergy (p = 0.003). Delayed skin response to cow’s milk was noted in 3 of 47 (6.38 %) children with autism, in 3 of 29 (10.34 %) with food allergy and in 2 of 27 (7.41 %) children with abdominal symptoms (p = 0.831). No difference was observed in the study group between soya (p = 0.882), cocoa (p = 0. 850), rice (p = 0.746), barley (p = 0.637), peanuts (p = 0.589), corn (p = 0.527), oat (p = 0.187), sesame (p = 0.195), wheat (p = 0.183) and yolk (p = 0.071).

**Conclusion:** Delayed-type sensitization to gliadin found in children diagnosed with autism may be considered in future research as a potential mechanism involved in multi-symptomatic clinical manifestation accompanying autism.

**Funding:** Project funded by the Ministry of Science and Higher Education No. N N407 53 1538.

## POSTER VIEWING SESSION 4: Asthma—Rhinitis (PP59–PP87)

### PP59 Systematic review of incense as a trigger factor for asthma

####  Chandramani Thuraisingham, Davendralingam Sinniah

##### International Medical University Clinical School Seremban, Seremban, Malaysia

###### **Correspondence:** Chandramani Thuraisingham

*Clinical and Translational Allergy* 2016, **6(Suppl 1)**:PP59

**Introduction:** Asthma is a multifactorial disease with many well-known triggers. One suspected environmental trigger factor (TF) is incense smoke that is generated during religious ceremonies by the traditional custom of burning incense at places of worship, and also in homes to eliminate odours and freshen the air. In view of this widespread practice by billions, it is imperative to determine if this suspected link between incense smoke and asthma is evidence-based.

**Study objective:** Null hypothesis: Incense smoke is a TF for asthma. Alternative hypothesis: Incense smoke is not a TF for asthma.

**Methodology:** A search of the medical literature published in the English language from 1960 up to 2014 using PubMed, TRIP Database, Cochrane Library Ebsco Host, Google Scholar and bibliographies of retrieved articles, was carried out to look for objective evidence that support, incriminate, question or refute the role of incense as a potential trigger for asthma. The articles reviewed: 1. Epidemiological association between inhalation of incense and asthma. 2. Association between genetic susceptibility, inhalation of incense smoke and asthma, and, 3. Evidence incriminating incense smoke as a trigger for inflammation in human lung biopsy material and/or tissue culture of respiratory mucosal cells of experimental animals.

**Results:** 1–2. Control studies show evidence based epidemiological and genetic relationship between incense smoke and asthma. 3. Animal and human lung cell studies show that exposure to incense smoke causes inflammation of mucosal cells lining the airways and pneumocytes; findings that are the hallmarks of asthma.

**Conclusion:** There is strong evidence incriminating incense smoke as a trigger factor for asthma.

**Take home message:** Incense is a trigger for asthma.

### PP60 Increased risks of mood and anxiety disorders in children with asthma

####  Yue Chen, Xiaomei Mei

##### School of Epidemiology, Pubic Health and Preventive Medicine, University of Ottawa. Ottawa, Canada

###### **Correspondence:** Yue Chen

*Clinical and Translational Allergy* 2016, **6(Suppl 1)**:PP60

**Background:** Asthma and mental health problems are common among youth and young adults. Their relationships exist, but study results are not entirely consistent.

**Objective:** The study was to determine the associations of mood and anxiety disorders with asthma in Canadian children.

**Methods:** The analysis was based on data from the Canadian Community Health Survey conducted in 2011–2012, and included 10,396 Canadian children aged 12–17 years. The survey used a multistage stratified sampling design, and a questionnaire covered the information on mood disorder, anxiety disorder and asthma.

**Results:** In Canadian children aged 12–17 years, the prevalence was 12.2 % for asthma (11.6 weighted to the population), 3.2 % for mood disorder (2.7 % weighted to the population) and 5.2 % for anxiety disorder (4.9 % weighted to the population), respectively. Logistic regression analysis was used to determine the associations of mood and anxiety disorders with asthma after taking account for sex, age, body mass index, leisure time physical activity, smoking, and social economic status. Children with asthma had increased risks of mood disorder (adjusted odds ratio (OR): 2.07, 95 % confidence interval (CI): 1.57, 2.71) and anxiety disorder (adjusted OR: 1.75; 95 % CI: 1.40, 2.20).

**Conclusion:** Children with asthma had increased risks of mood disorder and anxiety disorder in Canadian children.

### PP61

**WITHDRAWN**

*Clinical and Translational Allergy* 2016, **6(Suppl 1)**:PP61

### PP62 Asthma Control Test (ACT) and Pediatric Asthma Quality of Life Questionnaire (PAQLQ) association in children

####  Sebnem Ozdogan, Pinar Karadeniz, Durdugul Ayyildiz-Emecen, Ummuhan Oncul

##### Şişli Etfal Research and Training Hospital, Istanbul, Turkey

###### **Correspondence:** Sebnem Ozdogan

*Clinical and Translational Allergy* 2016, **6(Suppl 1)**:PP62

**Objective:** In this study we aim to evaluate the association of Paediatric Asthma Quality of Life Questionnaire (PAQLQ) with Asthma Control Test (ACT) in children with persistent asthma.

**Material and method:** Children aged 7–17 years with poorly controlled persistent asthma were involved. At first visit, ACT and PAQLQ were applied to and pulmonary function test (spirometry) was performed in all subjects. Daily Inhaler therapy were initiated based on asthma severity according to international asthma guidelines. Patients were reevaluted following 6 week inhaler therapy and ACT, PAQLQ and PFT (Pulmonary function test) were performed.

**Results:** Out of 77 patients, 35 (45 %) were female. The mean age was 11.62 ± 2.35 years. Following 6 weeks daily inhaler therapy, the ACT scores, PAQLQ scores and all the parameters of PFT except FEV1/FVC were significantly increased (p < 0.05). There was a significant correlation between ACT scores and PAQLQ scores (r: < 0.5, p:0.001). The most asthma-related restricted activities were running (85.7 %), soccer (42.9 %), and climbing stairs (40.3 %) subsequently.

**Conclusion:** There is a strong correlation between ACT and PAQLQ.

**Keywords:** Asthma; children; pediatric asthma quality of life questionnaire; Asthma Control Test.

### PP63 Seasonal and gender variations in vitamin D levels in children with asthma and its association with pulmonary function tests

####  Sebnem Ozdogan, Gizem Sari, Sabanur Cavdar

##### Şişli Etfal Research and Training Hospital, Istanbul, Turkey

###### **Correspondence:** Sebnem Ozdogan

*Clinical and Translational Allergy* 2016, **6(Suppl 1)**:PP63

**Purpose:** There is no consensus on the association between vitamin D and asthma. In the last few years, there have been few studies showing the association between Vitamin D levels and asthma. We aimed to examine seasonal variation in vitamin D status in children with asthma and its association with pulmonary function tests.

**Methods:** We recruited children 8–17 years old diagnosed with asthma. Vitamin D levels (serum hydroxy vitamin D3) were obtained and pulmonary function tests were performed both in winter months (Jan, Feb, and March) and at the end of summer (August, September and October). The seasonal variation of vitamin D levels and lung function were examined.

**Results:** 56 children with asthma (M: 26, F:30, mean age: 11.9 ± 1.97) were recruited. The mean vitamin D level in winter was 13.36 ± 6.31 ng/ml. There was no significant correlation between vitamin D levels and lung function. The mean vitamin D level at the end of summer was increased to 22.89 ± 7.83 ng/ml. It was increased 74.6 % in male, 163.7 % in female. There was no correlation between vitamin D levels and lung function performed at the end of summer.

**Conclusion:** There is a seasonal and gender variation in vitamin D status in asthmatic children. Vitamin D levels do not correlate with lung function.

### PP64 Defining treatment response in childhood asthma: rationale and design of the Pharmacogenomics in the Childhood Asthma (PiCA) consortium

####  Niloufar Farzan^1^, Susanne J. Vijverberg^1^, Colin J. Palmer^2^, Kelan G. Tantisira^3^, Anke-Hilse Maitland-van der Zee^1^ on behalf of the PiCA consortium

##### ^1^Division of Pharmacoepidemiology & Clinical Pharmacology, Utrecht Institute for Pharmaceutical Sciences, Utrecht University, Utrecht, The Netherlands; ^2^Population Pharmacogenetics Group, Biomedical Research institute, University of Dundee, Ninewells Hospital and Medical School, Dundee, United Kingdom; ^3^Channing Division of Network Medicine, Department of Medicine, Brigham and Women’s Hospital and Harvard Medical School, Boston, MA, USA

###### **Correspondence:** Niloufar Farzan

*Clinical and Translational Allergy* 2016, **6(Suppl 1)**:PP64

**Background:** *Genetic variation* may partly explain heterogeneity *in* response *to asthma treatment.* Recently the Pharmacogenomics **i**n **C**hildhood **A**sthma (PiCA) consortium was initiated to unite pediatric asthma cohorts, (high risk) birth cohorts and clinical studies and to perform large-scale pharmacogenomics studies.

**Aims:** To describe the characteristics of the patients included in PiCA and to assess treatment response definitions within this consortium.

**Methods:** An online survey was send to the 22 studies included in the PiCA consortium. The survey included questions about data collection, patient characteristics and outcome phenotypes.

**Results:** In total, 14,809 children/young adults (up to 23 years) from 11 different countries are enrolled in the PiCA consortium. Preliminary analysis shows that half of the asthmatic patients (55 %) are male. PiCA is ethnically diverse: approximately 52 % participants are Caucasians, 21 % Asians, 18 % Latinos and 9 % African-Americans. PiCA currently includes data of 1775 severe asthmatics. Furthermore, PiCA currently includes 14,179 short acting β_2_-adrenergic agonists (SABA) users, 11,314 ICS users, 1977 leukotriene modifiers (LTMs) users and 1964 users of long acting β_2_-adrenergic agonists (LABA). Most studies had information regarding exacerbations despite treatment (95 % of the studies), lung function measurements (85 % of the studies) and asthma symptoms despite treatment (80 % of the studies).

**Discussion:** The children within the PiCA consortium are a good reflection of the global heterogeneous pediatric asthma population. Different outcome measures reflect different dimensions of asthma. Therefore, by classifying three outcome measures using data that are widely available within the PiCA consortium, we will identify non-responders to asthma maintenance treatment.

### PP65 Prevalence of asthma and allergic disease in patients with inflammatory disease compared to celiac disease

####  Fatma Yavuzyilmaz, Sebnem Ozdogan, Nafiye Urganci, Merve Usta

##### Department of Pediatrics, Şişli Hamidiye Etfal Research and Training Hospital, Istanbul,

###### **Correspondence:** Fatma Yavuzyilmaz

*Clinical and Translational Allergy* 2016, **6(Suppl 1)**:PP65

**Objective:** Increased incidence of atopy and asthma has been reported in autoimmune disease such as celiac diseases and inflammatory bowel disease (IBD). In this study we aimed to investigate the prevalence of asthma and allergic disease in IBD compared to celiac disease.

**Materials and methods:** The validated ISAAC questionnaire in Turkish was applied to all patients with celiac disease and IBD.

**Results:** A total of 153 patients (70 with IBD and 83 with celiac disease) were involved. 52.9 % of patients were female and the mean age was 13.21 ± 3.80 years. The prevalence of wheeze was 26.6 % at any time (24.3 % in IBD and 28.9 % in celiac disease), and 18.2 % for the last 12 months (17.1 % IBD, 19.3 % celiac disease). The prevalence of allergic rhinitis was 3/.5 % at any time (41.4 % in IBD and 33.7 % in celiac disease).

**Conclusion:** The prevalence of asthma and allergic disease were similar in both children with IBD and celiac disease.

### PP66 A severe case with cystic fibrosis (CF) asthma

####  Mehmet Hoxha, Maksim Basho

##### UHC, Tirana, Albania

###### **Correspondence:** Mehmet Hoxha

*Clinical and Translational Allergy* 2016, **6(Suppl 1)**:PP66

Severe, difficult-to-treat asthma still constitutes a diagnostic and therapeutic problem. The diagnosis of asthma can be difficult, especially in preschool children. It is difficult to determine which wheeze is related with asthma or cystic fibrosis lung disease. A personal history of atopy, and a family history of atopy are probably the most useful guides of asthma.

**Case report:** A 7 years old boy was appeared in our consulting service. He had severe episodes of breathless, productive cough, wheezes frequently, nasal obstruction, several viral illnesses per year, etc. He had patches of dry skin on elbows and cheeks. Nasal polyps noted. The child was diagnosed with atopic bronchial asthma and he was uncontrolled patient, despite optimal conventional therapy and specific immunotherapy for mites. He was followed up in an allergy clinic assigned as “treatment resistant asthma patient”.

Clinical and laboratory evaluation were performed: blood tests, biological tests, induced sputum cell profile for eosinophils and neutrophils, ECP concentration, skin prick test and serum radioallergosorbent tests for the common aeroallergens, serum total IgE, sweat test (CF). We evaluated the level of nitric oxide in exhaled and nasal air. We performed standard spirometric tests and body plethysmography which resulted obstructive pattern with pulmonary hyperinflation and increased airways resistance. High resolution CT scanning was very useful, it reveals extensive small airways disease manifest by distal air trapping due to fixed obliterative bronchiolitis. Based on clinical and functional examinations we concluded the diagnosis of CF asthma, and we stopped specific immunotherapy.

**Conclusion:** The diagnosis of “CF asthma” is problematic and it is difficult to determine which patients have a combination of CF and asthma and which have asthma like symptoms caused by inflammation of CF lung. We know what have in common CF and asthma, but we have to consider the differences between them based on: genetic basis, single gene versus many, tissue and organ affected, airway inflammation, different treatments, antibiotics and nutrition, and which is more important the different progression of disease.

**Consent to publish**

Written informed consent for publication of this clinical details and/or clinical images was obtained from the patient/parent/guardian/relative of the patient. A copy of the consent form is available for review by the Editor of this journal.

### PP67 Severe asthma exacerbation complicated with pneumothorax in a child with uncontrolled asthma due to poor treatment compliance

####  Ioana Valentina Nenciu, Andreia Florina Nita, Adina Lazar, Alexandru Ulmeanu, Carmen Zapucioiu, Dumitru Oraseanu

##### Carol Davila University of Medicine and Pharmacy, Bucharest, Romania

###### **Correspondence:** Ioana Valentina Nenciu

*Clinical and Translational Allergy* 2016, **6(Suppl 1)**:PP67

**Background:** Asthma, the most common chronic disease of childhood and one of the leading causes of morbidity in children worldwide is currently affecting more than 14 % of world`s children (The Global Asthma Report 2014). The relationship between asthma and pneumothorax still needs to be established since several reports emphasize that pneumothorax is an uncommon complication of asthma (Porpodis K, Zarogoulidis P, Spyratos D, et al. Pneumothorax and asthma. *Journal of Thoracic Disease*. 2014;6(Suppl 1):S152-S161. doi:10.3978/j.issn.2072-1439.2014.03.05).

**Case report:** We report a case of a 9-year-old-girl with asthma, in treatment with Salmeterol and Fluticasone proprionate, with poor adherence to treatment, who presents to the Emergency Department with cough, wheezing and respiratory distress. The episode began 4 days earlier and it had been accompanied by a runny nose. She received symptomatic treatment but she has become more short of breath prior to coming to the ED.

On physical examination, she was afebrile, with pale teguments, cyanosis of nail beds and lips, in moderate respiratory distress, with flaring of the nostrils, rapid, shallow breathing and intercostal retractions. The vitals include a respiratory frequence of 40 rate/min, a heart rate of 150 beats/minute and pulse oximetry of 95 % on room air. The remainder of the examination is unremarkable.

Therapy is initiated with Ampicillin and Sulbactam, Methylprednisolone, Aminophylline, Albuterol, Fluticasone propionate. Over the next 24 h, her condition worsens, with frequent desaturation periods until 88 % while being under oxygen therapy, therefore she is transferred to the Pediatric Intensive Care Unit where she develops apical right sided pneumothorax. The further evolution is favorable, with dismissal after 2 weeks.

**Conclusion:** This case illustrates several issues related to management of a patient with an acute exacerbation of asthma complicated with pneumothorax in a child with poor adherence to treatment due to undisciplined parents.

**Consent to publish**

Written informed consent for publication of this clinical details and/or clinical images was obtained from the patient/parent/guardian/relative of the patient. A copy of the consent form is available for review by the Editor of this journal.

### PP68 Evaluation of the Pediatric Quality of Life inventory (PedsQL) asthma module among low income asthmatic children and adolescents in Sao Paolo, Brazil

####  Gustavo F. Wandalsen, Fernanda Monteiro, Dirceu Solé

##### Federal University of São Paulo, São Paulo, Brazil

###### **Correspondence:** Gustavo F. Wandalsen

*Clinical and Translational Allergy* 2016, **6(Suppl 1)**:PP68

**Rational:** PedsQL 3.0 Asthma Module is an instrument developed to measure the impact of asthma on health-related quality of life (HRQOL) in children and adolescents.

**Objective:** To evaluate the Portuguese version (Brazilian culture) of PedsQL Asthma Module among low income asthmatic children and adolescents.

**Methods:** PesdsQL Asthma Module was applied to 200 asthmatic children and adolescents (2–18 years) living in São Paulo, Brazil, and to their parents. HRQOL was also evaluated by PAQLQ and asthma control was measured by ACT or C-ACT.

**Results:** Moderate correlation was observed between children (PedsQL Child) and parents (PedsQL Parent) mean scores (r:0.67). PedsQL Child-Parent correlation was stronger among young children (5–7 years; r:0.76) and weaker among teens (13–18 years; r:0.54). Among the four questionnaire subscales, symptoms subscale showed the highest child-parent correlation (r:0.77) and communication subscale the lowest (r:0.46). Moderate correlation was found between mean PedsQL Child and mean PedsQL Parent scores and PAQLQ (r:0.66 and 0.64, respectively). Mean symptoms and worry subscale scores were higher among males (68 vs. 59 and 100 vs. 83, respectively; p < 0.05). No significant correlation was noted between mean PedsQL Child and mean PedsQL Parent scores and BMI, asthma duration and maternal education. The presence of atopic dermatitis and allergic rhinitis did not alter mean PedsQL scores. Progressive reduction in mean PedsQL Child and mean PedsQL Parent scores was noted with GINA treatment steps (PedsOL Child: 85 in GINA step 1 to 63 in GINA step 5).

**Conclusions:** The Portuguese PedsQL Asthma Module version showed to be a valid instrument among low income children and adolescents. In this group, we observed higher symptoms and communication subscale scores among males. Asthma severity was the main factor associated with impaired HRQOL.

### PP69 Early initiation of specific immunotherapy in asthma patients leads to higher benefits

####  Blerta Lame, Eris Mesonjesi, Arjeta Sherri

##### Allergology Department, University of Medicine, Tirana, Albania

###### **Correspondence:** Blerta Lame

*Clinical and Translational Allergy* 2016, **6(Suppl 1)**:PP69

**Introduction:** During the everyday practice patients with allergic asthma and allergic rhinitis are treated with inhaled corticosteroids, long-acting beta agonists, oral antihistaminic, specific immunotherapy (SIT). Specific Immunotherapy is indicated in patients with respiratory allergies (pollen, dust mite, mold) and Hymenoptera allergy. Specific immunotherapy, in addition to relieving symptoms, fights the causes of allergy.

**Case report:** We report a case of a 6 year-old girl, who suffered of frequent respiratory problems since she was born, manifested with cough (especially during the night), nocturnal dispnoae, wheezing, sneezing and runny nose. The little girl was diagnosed with allergic asthma and allergic rhinitis at the age of 3. In April 2013, skin prick tests were performed and resulted positive to house dust mites, cat and dog. During these years the doctors treated her with antibiotics, ketotifen, antihistaminics, inhaled corticosteroids, leukotriene receptor antagonists and nasal corticosteroids. Despite the regular use of controller medication she suffered several times a year of asthma exacerbations, where short-courses of oral corticosteroids were needed. In August 2013, we decided to start the subcutan immunotherapy with D. Pteronyssinus and D. Farinae. After 1 year of immunotherapy, last winter she had less severe asthma symptoms. She had no need for the rescue medication. The overall patient‘s conditions are improved.

**Conclusion:** Early initiation of Specific Immunotherapy can alleviate the symptoms of allergic rhinitis and alters positively the immune response. Allergen specific immunotherapy is the only treatment with immunomodulatory effect and capability to change natural course of allergic diseases.

**Consent to publish**

Written informed consent for publication of this clinical details and/or clinical images was obtained from the patient/parent/guardian/relative of the patient. A copy of the consent form is available for review by the Editor of this journal.

### PP70 Treatment resistant asthma and rhinosinusitis with recurrent pulmonary infections. Is it primary ciliary dyskinesia?

####  Alkerta Ibranji^1^, Laert Gjati^2^, Gjustina Loloci^3^, Ardii Bardhi^4^

##### ^1^Allergy and Clinical Immunology Department, “At Luigji Monti” Polyclinic, “Our Lady of Good Counsel” University, Tirana, Albania; ^2^Pulmonary Disease Department, University Hospital for Lung Disease, Tirana, Albania; ^3^Allergy and Clinical Immunology Department, Faculty of Medicine, Tirana University, Tirana, Albania; ^4^Imagery Department, “At Luigji Monti” Polyclinic, “Our Lady of Good Counsel” University, Tirana, Albania

###### **Correspondence:** Alkerta Ibranji

*Clinical and Translational Allergy* 2016, **6(Suppl 1)**:PP70

**Background:** Primary ciliary dyskinesia (PCD) is a relatively rare condition mainly inherited as an autosomal recessive with other inheritance patterns still under study. The incidence of PCD varies from 1:17,000 and 1–35,000 live births. Clinical symptoms in this pathology are; neonatal respiratory distress, recurrent lower respiratory tract infection, chronic rhino-sinusitis, bronchiectasis and visceral mirror image reported in approximately 50 % of cases. The prognosis is good, but if it is not managed properly, it leads to high morbidity levels. PCD, even though very rare, enters the differential diagnosis of atypical asthma and unusually severe upper and lower airway chronic diseases.

**Method:** We present the case of a 14 years old female asthmatic patient with chronic cough and rhinosinusitis resistant to treatments with local and inhaled steroids and bronchodilatators as well as oral antibiotics. She referred a neonatal respiratory distress and rhinitis from the early childhood and difficult to expectorate chronic productive cough and nasal yellowish discharge during the last 3 years with recurrent pulmonary infections, despite the aggressive asthma treatment.

**Results:** Auscultation evidenced labile cough-related ronchi and rales. Normal blood count and biochemical results found, negative skin prick test and sIgE for food and inhalant allergens. Thoracic- abdominal CT findings: situs inversus of liver, spleen and stomach, without dextrocardia or bronchiectasia. ORL investigation confirmed pan-sinusitis. Audiometri tests were normal. Respiratory function test showed a mild restriction. Sweat test resulted negative. Genetic testing and other specific diagnostic tools such as nasal nitric oxide, saccharin test, ciliary beat frequency and pattern on light microscopy are not available in Albania, so she was referred to a specialized clinic abroad.

**Conclusion:** A difficult to treat asthma with chronic rhinosinusitis is not a very rare scenario in allergist’s office. Difficult to expectorate sputum in asthmatic children with persistent rhinosinusitis, are important symptoms to consider PCD in the differential diagnosis. Despite the lack of the right diagnostic tools, thoracic-abdominal CT is very suggestive, even though half of the PCD patients have situs inversus. A multidisciplinary management and follow-up is essential in order to prevent the development of bronchiectasis and other morbidities.

**Consent to publish**

Written informed consent for publication of this clinical details and/or clinical images was obtained from the patient/parent/guardian/relative of the patient. A copy of the consent form is available for review by the Editor of this journal.

### PP71 The comparison of sensitisation to animal allergens in children- and adult- onset patients with asthma

####  Behnam Moghtaderi, Shirin Farjadian, Dorna Eghtedari

##### Shiraz University of Medical Sciences, Shiraz, Iran

###### **Correspondence:** Behnam Moghtaderi

*Clinical and Translational Allergy* 2016, **6(Suppl 1)**:PP71

**Background:** With higher exposure to animal during the life, the risk of sensitisation to animals may get more. The purpose of this study was the comparison of sensitisation to animal allergens in children with asthma from those with adult-onset asthma.

**Methods:** This cross-sectional study included 100 children and 100 adults with asthma as well as 100 healthy individuals with no history of asthma and atopy. Skin tests were performed in patients and controls to allergens using a panel of 15 animal allergens.

**Results:** The rate of sensitisation to animal allergens was 33 % in children with asthma, 39 % in patients with adult- onset asthma compared to 10 % in the control group. Children with asthma were most commonly sensitised to dog (10 %), hamster (8 %) and cat (7 %). Among patients with adult-onset asthma, the most common sensitisations were to dog (19 %), canary (14 %) and cat and goat (each 7 %). The frequency of sensitisation to animal allergens was not significantly different between children- and adult-onset patients with asthma.

**Conclusions:** We observed that sensitization to dog and canary was higher in adult-onset than children with asthma. Efforts to improve conditions at the public buildings to reduce the load of airborne allergens are also potentially helpful.

### PP72 Characterisation of children less than five years with wheezing episodes in Cali, Colombia

####  Manuela Olaya, Laura Del Mar Vasquez, Luis Fernando Ramirez, Carlos Daniel Serrano

##### Fundacion Valle del Lili, Cali, Colombia

###### **Correspondence:** Manuela Olaya

*Clinical and Translational Allergy* 2016, **6(Suppl 1)**:PP72

**Introduction:** Wheezing has been for a long time a frequent cause for consultation in pediatric hospitals. The aim of this study was to characterize children under 5 years old with wheezing episodes in the outpatient setting of a high complexity institution in Cali, Colombia.

**Methods:** A prospective study was conducted between April 2014 and March 2015. Patients from 0 to 5 years old with at least one wheezing episode were evaluated and studied for possible allergy disease. A complete medical record was taken, including possible risk/protective factors. Sensitization was determined by prick test for aeroallergens and food when we consider necessarily. We pursued correlation between risk/protection factors and asthma/wheezing.

**Results:** A preliminary analysis included 157 patients. 94 (59 %) were male. Mean age was 34.6 months old. 73 (47 %) received exclusive breastfeeding for 6 months. 25 (15.9 %) were in presence of smoking environment. 56 (35.7 %) had bronchiolitis in the first year of life. 109 (69.4 %) had history of atopia in first degree. In 94 patients (60 %), the skin prick test was performed. 42 (44.6 %) were positive for aeroallergens, of which the main sensitization was to *D. pteronyssinus* in 28 patients (67 %), *D farinae* in 16 (38 %), animal dander in 4 (9.5 %) and grass pollen in 2 (4.5 %). 64 patients (40.7 %) had rhinitis, 51 (32.4 %) had atopic dermatitis, 9 (5.7 %) had food allergy, and 2 (1.7 %) had urticaria. There was no correlation between protective/risk factors and the presence of asthma/wheezing.

**Conclusions:** This is the first prospective cohort in our country, in which clinical and epidemiologic data of wheezing children less than five years old are described. We did not find any correlation among different risk/protective factors and the presence of the disease.

### PP73 Evaluation of the patients with recurrent croup

####  Belgin Usta Guc^1^, Suna Asilsoy^1^, Fulya Ozer^2,3^

##### ^1^Department of Pediatric Allergy and Immunology, Faculty of Medicine, Adana Education and Research Hospital, Başkent University, Adana, Turkey; ^2^Department of Otolaryngology, Faculty of Medicine, Adana Education and Research Hospital, Başkent University, Adana, Turkey; ^3^Department of Head and Neck Surgery, Faculty of Medicine, Adana Education and Research Hospital, Başkent University, Adana, Turkey

###### **Correspondence:** Belgin Usta Guc

*Clinical and Translational Allergy* 2016, **6(Suppl 1)**:PP73

The published version of this abstract can be found at [1].

**Reference**

1. Allergy. 2011;66(Suppl 94):482–642. http://onlinelibrary.wiley.com/doi/10.1111/j.1398-9995.2011.02608.x/epdf.

### PP74 Obesity in adolescence compromising the asthma control

####  Guergana Petrova, Sylvia Shopova, Vera Papochieva, Snezhina Lazova, Dimitrinka Miteva, Penka Perenovska

##### Pediatric Clinic, University Hospital “Alexandrovska”, Sofia, Bulgaria

###### **Correspondence:** Guergana Petrova

*Clinical and Translational Allergy* 2016, **6(Suppl 1)**:PP74

**Background:** Childhood asthma and obesity are significant public health problems, affecting more children with each passing year. Excess weight and obesity in children may be fuelling the “asthma epidemic” faced in many developed countries in the latest years. After an excessive weight gain one seemingly “well-controlled” asthma, turns out to be “uncontrolled” despite the increase in therapy.

**Clinical cases:** We present a case of 13-year old boy with asthma since infancy, well controlled till 2 years ago with inhaled corticosteroid. Last 2 years the boy gained over 40 kilos and despite his combined therapy he has very poor asthma control for this period.

The second case is a 17-year old girl with bronchial asthma, poorly controlled despite high dose of combined corticosteroids as a controller medication. For the last 4 years she starts to show protest behavior towards therapy, that’s modifying in the course of psychological maturation-denial of the medicines, unhealthy and hazardous life styles. She gained over 30 kilos and at the age of 16-years depression was diagnosed, and was pharmacological and psychological therapy.

**Conclusion:** A modern team-work approach with respiratory/allergy specialist, nutritionist and psychology/psychiatry specialist sometimes is the best way to manage teenagers with difficult to treat asthma and obesity.

**Consent to publish**

Written informed consent for publication of this clinical details and/or clinical images was obtained from the patient/parent/guardian/relative of the patient. A copy of the consent form is available for review by the Editor of this journal.

### PP75 Sleep behavior in children with persistent allergic rhinitis

####  Gustavo F. Wandalsen, Jessica Loekmanwidjaja, Márcia Mallozi, Dirceu Solé

##### Federal University of São Paulo, São Paulo, Brazil

###### **Correspondence:** Gustavo F. Wandalsen

*Clinical and Translational Allergy* 2016, **6(Suppl 1)**:PP75

**Objectives:** To evaluate sleep disorders in children (4–10 years) with persistent allergic rhinitis (PAR) and its relation with rhinitis severity.

**Methods:** Parents from 90 PAR children followed in a reference clinic answered the Children Sleep Habits Questionnaire (CSHQ). This one-week retrospective questionnaire is composed by 33 questions and divided in 7 subscales. Total nasal symptom score (TNSS, range 0–12) and total extra nasal symptom score (TENSS, range 0–16) were recorded as well as nasal peak inspiratory flow (NPIF).

**Results:** Mean group age was 7.9 years. All of them were in regular use of nasal steroids or oral antihistamines. 74 % had concomitant asthma which was not controlled in 33 %. Mean CSHQ total score was 54 ± 8. No significant correlation was observed between CSHQ total scores and TNSS, TENSS and NPIF. Significant correlation was observed between CSHQ sleep duration subscale and NPIF (r: 0.25), between CSHQ parasomnias subscale and TENSS (r: 0.25), and between CSHQ sleep-disordered breathing subscale and TNSS (r: 0.32) and TENSS (r: 0.27). The presence of asthma or uncontrolled asthma did not change CSHQ total scores.

**Conclusions:** CSHQ total scores showed no correlation with rhinitis symptom scores. Some questionnaire subscales, however, could identify specific sleep disorders possibly related to allergic rhinitis severity.

### PP76 Randomised trial of the safety of MP29-02* compared with fluticasone propionate nasal spray in children aged ≥4 years to <12 years with allergic rhinitis

####  William Berger^1^, Ulrich Wahn^2^, Paul Ratner^3^, Daniel Soteres^4^

##### ^1^Allergy and Asthma Associates of Southern California, Mission Viejo CA, USA; ^2^Department of Pediatrics, Division of Pneumonology, Immunology and Intensive Care Medicine incl. Rescue Center, Charité University Hospital, Berlin, Germany; ^3^Sylvana Research Associates, San Antonio TX, USA; ^4^Allergy and Asthma Associates, Colorado Springs CO, USA

###### **Correspondence:** Ulrich Wahn

*Clinical and Translational Allergy* 2016, **6(Suppl 1)**:PP76

**Background:** MP29-02* (a novel intranasal formulation of azelastine hydrochloride (AZE) and fluticasone propionate (FP)) is approved for use in patients aged 12 years or older with moderate/severe allergic rhinitis (AR). The objective of this study was to evaluate the safety of MP29-02* compared to FP, administered as 1 spray per nostril twice daily, in pediatric AR subjects ≥4 to <12 years.

**Methods:** This was a randomized, open-label, 3-month study in patients (≥4–12 years). Qualified subjects had a history of AR, were in good health, and had no evidence of nasal mucosal erosion, nasal ulceration, nasal septum perforation, or any significant nasal disease. Subjects were randomized in a 3:1 ratio to MP29-02* (n = 304) or FP (n = 101) and were stratified by age as follows: (i) ≥4 years to <6 years; (ii) ≥6 to <9 years; and (iii) ≥9 to <12 years. Safety was assessed by subject and/or caregiver-reported adverse events (AEs), nasal examinations, vital signs, and laboratory assessments.

**Results:** Overall, 94 % of subjects treated with MP29-02* and 92 % treated with FP completed the study. Completion rates were similar in each age strata. The percentage of subjects with AEs was comparable between treatment groups and across the age strata. The most frequently reported AEs (i.e. ≥4 % in either group) with MP29-02* and FP, respectively, were: epistaxis (10 and 9 %), headache (7 and 3 %), cough (4 and 3 %) and diarrhoea (1 and 4 %). The discontinuation rate due to AEs was 2 % with MP29-02* and 4 % with FP. Laboratory parameters showed no meaningful increase in mean or median values in either treatment group. Improvements in nasal examination findings were observed in both treatment groups and in each age stratum and there were no findings of nasal mucosal ulceration or septal perforation. The two treatment groups were comparable for mean changes in vital sign measurements regardless of age stratification.

**Conclusion:** MP29-02* and FP were well-tolerated during this 3-month study when administered as 1 spray per nostril twice daily in pediatric subjects ≥4 to <12 years with AR.

*Dymista

### PP77 Safety and tolerability evaluation of bilastine 10 mg in children from 2 to 11 years of age with allergic rhinoconjunctivitis or urticaria

####  Zoltán Novák^1^, Anahí Yáñez^2^, Kiss Ildikó^3^, Piotr Kuna^4^, Miguel Tortajada^5^, Román Valiente^6^, the Bilastine Pediatric Safety Study Group

##### ^1^Aranyklinika Egészségügyi és Innovációs Kft., Szeged, Hungary; ^2^INAER-Investigaciones en Alergia y Enfermedades Respiratorias, Buenos Aires, Argentina; ^3^Children Department, Zala Megyei Kórház, Zalaegerszeg, Hungary; ^4^Allergy and Lung Diseases, SPZOZ Uniwersytecki Szpital, Lodz, Poland; ^5^Departamento de Pediatría, Hospital Universitario Dr. Peset, Valencia, Spain; ^6^Clinical Research Department, FAES FARMA S. A., Leioa, Bizkaia, Spain

###### **Correspondence:** Zoltán Novák

*Clinical and Translational Allergy* 2016, **6(Suppl 1)**:PP77

**Objective:** The main objective of this study was to assess safety and tolerability of bilastine, antihistamine marketed for adults and adolescents with pediatric development ongoing, in a 10 mg once daily dose for children aged 2–11 years with allergic rhinoconjunctivitis or chronic urticaria.

**Methods:** Multicenter, double-blind, randomized, placebo-controlled, parallel group study in 504 boys and girls aged 2–11 with either allergic rhinoconjunctivitis (AR) or chronic urticaria (CU). The primary endpoint was the occurrence of TEAEs (Treatment Emergent Adverse Events) throughout the 12 week treatment period to the last non treatment safety follow-up visit at week 16.

**Results:** The study has demonstrated the primary hypothesis of non-inferiority for Bilastine 10 mg with respect to placebo (p = 0.015). The proportion of children without TEAEs was 31.5 % (82 patients) in the Bilastine 10 mg group and 32.5 % (81 patients in the placebo group, showing a mean difference of 0.99 with a 95 % confidence interval ranging from −9.10 to 7.10. No statistically significant differences between treatment groups were found regarding the TEAEs and the related TEAEs reported (5.8 % in bilastine group and 8.0 % in placebo group). The most frequently reported TEAEs were headache, reported by 11.5 % patients in the Bilastine group and 10.4 % in the placebo group, followed by cough, reported by 23 and 22 patients in the Bilastine and placebo groups. Both Bilastine 10 mg and placebo showed a slight decrease in the somnolence and sedation as assessed with the Pediatric Sleep Questionnaire, with no statistically significant differences between groups.

**Conclusions:** Based on the study results, the incidence of TEAEs of Bilastine 10 mg in children 2–11 years old was similar to placebo. It confirms that Bilastine 10 mg once daily as a good and safe antihistamine for children with allergic rhinoconjunctivitis or urticaria.

### PP78 Sensitisation to Alternaria alternata: Is it a risk factor for severe rhinitis?

####  Susana Lopes, Filipa Almeida, Tânia Lopes, Cristina Madureira, José Oliveira, Fernanda Carvalho

##### Centro Hospitalar do Médio Ave, Vila Nova de Fa, Portugal

###### **Correspondence:** Susana Lopes

*Clinical and Translational Allergy* 2016, **6(Suppl 1)**:PP78

**Introduction and aim:** Allergic rhinitis (AR) is a worldwide condition with a lifetime prevalence ranging from 10 to 30 %. In some pediatric studies, sensitization to Alternaria alternata (AA) had been associated with more persistent and severe asthma in adulthood. Fungal sensitization is also found in allergic rhinitis without asthma, but there are no studies showing AA as a risk factor for more severe disease. Authors aim to clinically characterize a group of patients with AR without asthma, compare those sensitized and non-sensitized to AA and evaluate if sensitization to AA was a risk factor to rhinitis severity and co-morbidities.

**Materials and methods:** Retrospective study involving pediatric patients with AR without asthma admitted at our outpatient clinic in 2014. Clinical files were reviewed. ARIA classification was used and AA sensitization was assessed by skin prick test.

**Results:** 168 participants, 64.9 % boys, with a median age of 12 years old. A family history of atopic disease was found in 49.4 and 16.9 % were sensitized to AA. According to ARIA classification, 66.1 % presented with intermittent symptoms and 67.9 % with mild AR. Conjunctivitis and atopic dermatitis were found in 25.6 and 14.9 %, respectively. Among AA-sensitized patients, 6 % were monosensitised, 39.4 % were mainly sensitized to mites, 21.2 % to grasses and 15.2 % to both. Any significant differences were found between AA-sensitized and no-sensitized patients concerning to disease severity using ARIA classification, previous month use of anti-histaminic, nasal glucocorticoid or montelukast or immunotherapy. There were also no differences in co-morbidities rates, namely conjunctivitis, atopic dermatitis, sinusitis, nasal poliposis or upper-airway surgical interventions.

**Conclusions:** The prevalence of AA sensitization in AR without rhinitis was similar to previous studies and the prevalence of monosensitised patients was lower. This study failed to demonstrate an association between AA-sensitization and AR severity.

### PP79 Validation of the Patient Benefit Index (PBI) for the assessment of patient-related outcomes in allergic rhinitis in children

####  Julia Feuerhahn^1^, Christine Blome^1^, Meike Hadler^2^, Efstrathios Karagiannis^2^, Anna Langenbruch^1^, Matthias Augustin^1^

##### ^1^University Medical Center Hamburg-Eppendorf, Hamburg, Germany; ^2^Stallergenes GmbH, Kamp-Lintfort, Germany

###### **Correspondence:** Efstrathios Karagiannis

*Clinical and Translational Allergy* 2016, **6(Suppl 1)**:PP79

**Introduction and objectives:** Allergic rhinitis (AR) is a high-prevalence disease in children and adolescents in Western countries. We developed a specific version of the Patient Benefit Index (PBI) questionnaire for the assessment of patient-relevant treatment needs and benefits in children and adolescents with AR. The objective was to validate the PBI in patients receiving allergen immunotherapy (AIT), which is currently the only causal treatment of AR.

**Methods:** The questionnaire “PBI-AR-K” was developed by an expert panel including pediatric patients with AR. It consists of two questionnaires with 19 items each. In the first part, patients rate treatment goal importance; in the second part, they rate the extent to which the goals have been fulfilled by therapy. The PBI-AR-K was validated in a non-interventional, longitudinal, multi-center study. Children (5–12 years) and adolescents (13–17 years) with grass pollen-induced AR were treated with the 5-grass pollen tablet and followed up from the first exposure of sublingual AIT until the end of the first treatment year.

**Results:** Data of 246 children and 135 adolescents (35 %/44 % female) under AIT with the 5-grass pollen tablet were evaluated. The feasibility of the PBI was supported by a low rate (0.8 %/0.7 %) of patients with >25 % missing item pairs, the threshold for score calculation. However, many did not complete one or both questionnaires (33 %/34 %). All subscales, developed on the basis of factor analyses, were internally consistent (Cronbach’s alpha ≥ 0.8). The PBI and most subscales correlated highly significantly (p < 0.001) with patients’ and physician’s judgments on treatment benefit and with changes in frequency of rhinitis symptoms. Mean PBI was 2.6 in children, 2.5 in adolescents (0 “no benefit” to 4 “maximum benefit”). Applying a threshold of 1, 95 % of the children and 91 % of the adolescents gained a relevant benefit from sublingual AIT treatment with the 5-grass pollen tablet.

**Conclusion:** The PBI-AR-K is a valid, reliable and suitable instrument for the assessment of patient-reported benefit in the treatment of pediatric AR. Treatment with the 5-grass tablet showed a considerable patient benefit.

### PP80 Efficacy of sublingual tablet of house dust mite allergen extracts in adolescents with house dust mite-associated allergic rhinitis

####  Michel Roux^1^, Shinji Kakudo^2^, Efstrathios Karagiannis^3^, Robert K. Zeldin^1^

##### ^1^Stallergenes, Antony, France; ^2^Shionogi & Co. Ltd, Osaka, Japan; ^3^Stallergenes Germany GmbH, Kamp-Lintfort, Germany

###### **Correspondence:** Efstrathios Karagiannis

*Clinical and Translational Allergy* 2016, **6(Suppl 1)**:80

**Background:** Allergen immunotherapy with house dust mite (HDM) sublingual tablet has proven efficacious and safe in subjects with allergic rhinitis (AR). Here we report efficacy results in the subset of adolescents with HDM-associated AR in a Phase III rhinitis study.

**Methods:** In a DBPC study conducted in Japan, adults and adolescents (12–64 years) with HDM-associated AR confirmed by a positive HDM-specific IgE test and a positive response to a nasal provocation test were randomised 1:1:1 to receive HDM tablet at 500IR or 300IR, or placebo once daily for 1 year. The primary efficacy endpoint was the Average Adjusted Symptom Score (AASS, an Average Rhinitis Symptom Score adjusted for rescue medication use; scale 0–15) over the last 8 weeks of treatment. This criterion was analysed using an analysis of covariance.

**Results:** Of the 968 subjects randomised, 181 were adolescents aged 12–17 years (500IR = 61, 300IR = 60, Placebo = 60) and 171 of these were included in the efficacy analysis set. In this subset, statistically significant differences between 500IR (p = 0.0001) and 300IR (p < 0.0001) compared to placebo were observed on the primary efficacy endpoint. Over the last 8 weeks of treatment, the AASS Least Squares mean differences vs. placebo were −1.88 (CI_95%_ [−2.84, −0.93]) and −2.04 (CI_95%_ [−3.01, −1.08]) for 500IR and 300IR respectively, corresponding to relative reductions vs. placebo of −24.8 % for 500IR and −26.9 % for 300IR. These results were consistent to those obtained in the overall population.

**Conclusion:** In a Phase III study of subjects with HDM-associated allergic rhinitis, treatment with 500IR and 300IR sublingual tablets of HDM allergen extracts administered for 1 year was efficacious in the subset of adolescents.

**Conflict of interest disclosure:** Presenting author is an employee of Stallergenes.

### PP81 Lung function improvement in a child treated with omalizumab for bronchial asthma

#### Anna Sokolova^1^, Tiago Milheiro Silva^2^

##### ^1^PediatricsDepartment, Hospital Professor Doutor Fernando Fonseca, Amadora, Portugal; ^2^PediatricsDepartment, Hospital de Dona Estefania, Lisbon, Portugal

###### **Correspondence:** Anna Sokolova

*Clinical and Translational Allergy* 2016, **6(Suppl 1)**:PP81

**Background:** Omalizumab is a recombinant humanized monoclonal antibody that binds to IgE molecules, forming biologically inert complexes. It is indicated as add-on therapy to improve asthma control in children aged ≥6 years.

**Objective:** Clinical case report.

**Methods and results:** The authors present the case of a 15-year old boy with a 10 year history of asthma. Despite optimized treatment with high dose inhaled corticosteroids, long acting bronchodilator and leukotriene antagonist, he continued to present symptoms consistent with severe persistent asthma with frequent visits to the emergency department and oral corticosteroid treatment. Skin testing was positive for house-dust mites and total serum IgE levels ranged from 244 to 597 UI/mL. Lung function assay’s showed FEV1 levels of 52 % of the predicted value. At the same time, asthma control tests (ACT) scores ranged from 6 to 9. He was started on Omalizumab 225 mg monthly and after 16 weeks of therapy there was a marked clinical improvement, without the need of systemic corticosteroid rescue therapy since the beginning of the therapy, improved ACT scores (≥20) and it was possible to reduce the required dose of inhaled corticosteroids. We also noticed a marked improvement in lung function, with FEV1 levels of 88 % of the predicted after 16 weeks of treatment.

**Conclusion:** Besides the clear effect of Omalizumab in reducing exacerbations and the required dose of inhaledcorticosteroids with no need of oral corticosteroidtreatment, the authors point to the fact that it may also improve lung function. Further assessments are needed to study this effect.

**Consent to publish**

Written informed consent for publication of this clinical details and/or clinical images was obtained from the patient/parent/guardian/relative of the patient. A copy of the consent form is available for review by the Editor of this journal.

### PP82 How to treat a child suffering from asthma, allergic rhinitis, allergy to peanuts and diabetes at the same time?

####  Snezana S. Zivanovic, Vesna Cvetkovic, Ivana Nikolic, Sonja J. Zivanovic,

##### Children’s Hospital, Clinical Center Nis, Faculty of Medicine, University of Nis, Nis, Serbia

###### **Correspondence:** Snezana S. Zivanovic

*Clinical and Translational Allergy* 2016, **6(Suppl 1)**:PP82

**Background:** Disruption of immune tolerance in early childhood causes different diseases such as allergic and autoimmune diseases.

**Objective**: We report the case of boy, aged 11, suffering from diabetes, with high atopical status and difficult-to-control asthma.

**Methods and results**: The patient has diabetes mellitus type 1 since the age of 4 and had been treated with different insulin regimens. He has been hospitalised more than 14 times due to suboptimal diabetes control. Two years after establishing the diagnosis of diabetes, at the age of 6, viral upper respiratory infection triggered wheezing symptoms. Eczema is diagnosed in 7, while allergic rhinitis in 8 years of age. The patient’s older brother has a diagnosis of asthma. Four months after the initial wheezing episode, 4 recurrent episodes of wheezing occurred in the springtime, thus leading to establishing the diagnosis of allergic asthma. He presented a high atopical status: total IgE level of 12 300 IU/ml and allergen-specific IgE tests to birch pollen, Hordeum murinum, timothy grass, meadow grass, meadow foxtail, couch grass, orchard grass >100, (class VI), ambrosia 3.08, house dust mites 5, 41, peanuts >100 (VI), soya 93.4 (VI), egg whites 30.7; wheat 9.17; milk 3.07 IU/ml; and peripheral eosinophilia 12 %. Therapy started with low doses of inhaled corticosteroids (ICS). However, there was a major problem of adherence to the prescribed treatment. In the autumn the same year, the patient experienced 3 wheezing episodes as well as clinical manifestations of allergy to peanuts in the form of rash and angioedema. Spirometry test detected no pulmonary ventilation defect. The following year, partially controlled asthma was further complicated by rhinitis symptoms. The treatment with montelukast combined with low dose ICS was prolonged.

**Conclusion**: Our patient suffers from Th1 autoimmune disease (Diabetes mellitus) combined with Th2 mediated allergic diseases (asthma, rhinitis, eczema) which is commonly found in literature, yet rarely seen in medical practice. Being difficult to treat and put under control, having chronic course, affecting negatively both the patient and his family in a sense of quality of life, these conditions represent a major challenge to doctors.

**Consent to publish**

Written informed consent for publication of this clinical details and/or clinical images was obtained from the patient/parent/guardian/relative of the patient. A copy of the consent form is available for review by the Editor of this journal.

### PP83 Nitric oxide in exhaled air in the relationship of the degree of sensitisation to aeroallergens

####  Snezana S. Zivanovic^1^, Ljiljana Saranac^1^, Ivana Nikolic^1^, Sonja J. Zivanovic^1^, Zorica Zivkovic^2^

##### ^1^Children’s Hospital, Clinical Center Nis, Faculty of Medicine, University of Nis, Nis, Serbia; ^2^Children’s Hospital for Lung Diseases and Tuberculosis, Medical Center, Nis, Serbia

###### **Correspondence:** Snezana S. Zivanovic

*Clinical and Translational Allergy* 2016, **6(Suppl 1)**:PP83

**Aim:** The examination of nitric oxide in exhaled air in children suffering from asthma and to establish the relation with the degree of sensitization to aeroallergens.

**Materials and methods:** The examination included fifty-two children (aged 12.40 ± 2.35 years), twenty-eight male (53.85 %) and twenty-four female (46.15 %), with the average length of suffering from asthma 8.33 ± 3.93 years. FeNO was measured by using the online method, the NIOX MINO device. The degree of sensitization to aeroallergens was determined by skin prick testing and assessed using the atopic index. The atopic index was indicated by the number of positive prick tests (AI 0—a negative prick test, AI 1—a positive number of prick tests <2 allergens, AI 2—a positive prick test <4 allergens and AI 3—a positive prick test ≥5 or more allergens).

**Results:** The average value of FeNO in exhaled air of children suffering from stable allergic asthma was 43.92·35.63 ppb, and after a 4 week anti-inflammatory treatment it decreased to 34.92·32.04 ppb (p < 0.05). In relation to AI (AI I, AI II i AI III), the level of FeNO in exhaled air was 41.00 vs. 40.69 vs 50.88 ppb, in the given order without statistically significant difference. The highest values of FeNO in exhaled air were present in children suffering from a mixed type of sensitisation (sensitisation to seasonal and common environmental allergens), 56.85 ppb (Me 48.50) in comparison to sensitisation to seasonal allergens 15.29 ppb (Me 12) and indoor allergens 32.22 ppb (Me 26). Allergic rhinitis, the duration of asthma and the gender were not significantly related to the values of FeNO in exhaled breath, while significant was the negative correlation between the body mass index and FeNO, r = −0.43 (p˂0.01).

**Conclusion:** Children suffering from allergic asthma possess increased values of nitric oxide in exhaled air, which is a useful indicator of daily dosage adjustment in patients treated with anti-inflammatory drugs.

### PP84 Clinical basis of diagnostic errors in pediatric asthma

####  Zoia Nesterenko

##### Saint-Petersburg State Medical University, Saint-Petersburg, Russia

###### **Correspondence:** Zoia Nesterenko

*Clinical and Translational Allergy* 2016, **6(Suppl 1)**:PP84

**Background:** Current diagnosis of pediatric asthma can’t include all its clinical variants and results to be a complex task.

**Aim:** To analyze the most common diagnostic errors in pediatric asthma.

**Materials and methods:** 82 patients aged 4–18 years were observed. Mild asthma was in 20.7 % of patients, moderate—in 79.3 %.

**Results:** In 37.1 % of patients community-acquired pneumonia (CAP) was diagnosed, caused in 51.6 % of cases by Mycoplasma pneumonia (MP), in 29 %—by Cytomegalovirus (CMV), and provoked asthma exacerbation in them (r_xy_ = 0.9). In 62.2 % of cases CAP recurrent episodes occurred (CAP). In 64.7 % of patients somatic pathology of osteoarticular, cardiovascular, gastrointestinal systems, besides respiratory, was observed, that resulted in variety of non-respiratory complains with difficulties in asthma diagnosis. In the first 2 years of life asthma diagnosis and adequate therapy was provided in 22 % of children, in 3–5 years since asthma onset it was revealed in 75.6 % of patients, after 5 years of disease manifestation—in 2.4 %. In the first 2 years of asthma manifestations its symptoms were assessed as “frequent acute respiratory infections“(ARI) in 55 % of children, in 44.4 %—mild heart abnormalities (MHA) were detected. 38.7 % of children with asthma diagnosed in 3–5 years since its onset were observed with frequent ARI, 6.5 %—with recurrent bronchitis (RB), 17.7 %—with recurrent CAP, 16.1 %—with MHA, 9.7 %—with autonomic dysfunction, 11.3 %—with gastroesophageal reflux (GER) and frequent ARI. Absence of asthma controller therapy led to development of complications: pulmonary hypertension (PH)—in 32.9 % of patients; pulmonary fibrosis (PF)—in 40.7 % of asthmatic patients with CAP; emphysematous bullae (EB)—in 45.5 % of them.

**Conclusions:**

1. Somatic pathology of 3–4 systems in asthmatic patients caused variety of complains and delayed asthma diagnosis with development of complications at diagnosis since 2 years of its onset.
2. Close relationship between asthma exacerbation and recurrent CAP caused by MP, CMV that led to development of PH, PF, EB is revealed.
3. Thorough examination of patients with frequent ARI is important to exclude asthma.

### PP85

**WITHDRAWN**

*Clinical and Translational Allergy* 2016, **6(Suppl 1)**:PP85

### PP86 Childhood asthma control in Serbia and organised Asthma Educational Intervention (AEI)

####  Snezana Radic^1^, Branislava Milenkovic^2^, Spomenka Smiljanic^1^, Milka Micic-Stanijevic^1^, Olivera Calovic^1^

##### ^1^KBC Dr. Dragisa Misovic, Children’s Hospital for Respiratory Diseases, Belgrade, Serbia; ^2^Clinic for Pulmonary Diseases, Clinical Centre of Serbia, Belgrade, Serbia

###### **Correspondence:** Snezana Radic

*Clinical and Translational Allergy* 2016, **6(Suppl 1)**:PP86

**Background:** To try to investigate how is childhood asthma controlled in Serbia and is Asthma educational intervention (AEI) on children and parents/caregivers effective in asthma control improvement.

**Materials and methods:** 20.042 children from 28 primary schools (6–15 years old) in Belgrade, Serbia, were investigated and invited for the asthma education. According to questionnaire, we separated 707 children already diagnosed as asthma (prevalence rate 8.61 %), however, only 527 children and parents/caregivers completed AEI (education and 2 control check-ups 6 and 12 months later). For the assessment of asthma control, children and parents/caregivers completed the Asthma Control Test before the education (ACT1) and at the control check-ups (ACT2, ACT3). For children 7–11 years old, we put together answers of children and parents; children older than 12 answered solely. The sum >19 points represented good control of asthma, while sum ≤19 represented poor control of the illness.

**Results:** There were 321 (60.9 %) of boys and 206 (39.1 %) of girls (mean age 10.7; the first wheezing episode at 2.3 and diagnose of asthma at 3.3 years; 4.5 ± 4.6 asthma exacerbations, 2.2 ± 2.8 nights disturbed by asthma exacerbation, 1.0 ± 2.6 hospitalizations, and 8.9 ± 5.4 school days lost 12 months before the study). At least one positive skin prick test had 385 children (73.1 %). There were 296 (56.2 %) children 7–11 years old, mean ACT1 was 20.3 ± 5.3, 130 children had ACT1 ≤ 19 (43.9 %); after the AEI, control of asthma statistically improved (ACT1114 children had ACT1 ≤ 19 (49.4 %); after the AEI, control of asthma statistically improved ACT1).

**Conclusions:** Childhood asthma is not successfully controlled in Serbia, as in many other countries. There is a space for intervention. Education of children and parents/caregivers about asthma is valuable tool for asthma control improvement.

### PP87 Experience from a group of adolescents with severe allergic asthma treated with Omalizumab

####  Anne Marie Bro Hofbauer, Lone Agertoft

##### Hans Christian Andersen Children’s Hospital, Odense University Hospital, Odense, Denmark

###### **Correspondence:** Lone Agertoft

*Clinical and Translational Allergy* 2016, **6(Suppl 1)**:PP87

**Background:** Omalizumab, a subcutaneous anti IgE monoclonal antibody, is shown to be effective in severe allergic asthma.

**Aim:** To evaluate the effect of group based education and structured dialogue among adolescents being treated with omalizumab in enhancing knowledge and insight in the asthma disease, treatment, social behaviour and experience.

**Method:** The adolescents were invited to share their experience after treatment with omalizumab. A group of 4 (13–16 years of age) participated some hours one afternoon. They received education about asthma and treatment using question and answer cards and they were informed about effect and adverse effects of omalizumab. The dialogue included questions about life before and after omalizumab treatment, relation to family and friends, ability and lack of ability to perform physical activities and dreams of the future. One week later they completed a telephone interview based on questions handed out at the session.

**Results:** All 4 adolescents experienced a positive change in everyday life and ability to manage the asthma disease. Furthermore, they all experienced an enhanced physical ability and appreciated the knowledge about omalizumab and asthma. In the interview they reflected on their own disease and physical ability, and they were satisfied by knowing that all in the group had the same thoughts and feelings. Besides one in the group, who in many years had struggled with the ability to perform sports, found out, that her expectation was unrealistic.

**Conclusion:** Adolescents with a chronic disease seems to have a beneficial effect being together in a group sharing thoughts, emotions and knowledge about their disease and treatment.

## THEMATIC POSTER SESSION 1: Prevention and Treatment—Epidemiology (TP01–TP18)

### TP01 A cost effective primary school asthma education program: pilot study from inner London schools

####  Lucy Everson^1^, Jessica Kearney^1^, Jonny Coppel^1^, Simon Braithwaite^1^, Rahul Chodhari^2^

##### ^1^University College London, London, United Kingdom; ^2^The Royal Free NHS London Foundation Trust, London, United Kingdom

###### **Correspondence:** Rahul Chodhari

*Clinical and Translational Allergy* 2016, **6(Suppl 1)**:TP01

**Introduction:** There are on average 2 children with asthma in every classroom.

It is thought that as many as 75 % of deaths from asthma are preventable.

Building on children’s curiosity in the classroom about asthma is a possible method of increasing awareness of the condition and its treatment, with the overall aim of reducing hospital admissions and deaths.

**Methods:**

- UCL medical students who were trained in asthma education visited 14 schools in inner city London.
- 1443 children between the ages of 7 and 13 took part.
- The children completed a quiz before and after a 15 min presentation on asthma. The presentation and the quiz questions addressed treatment, emergencies, triggers, misconceptions and basic physiology.
- The average total score (out of 13) and marks for each question were calculated and the pre and post presentation test results compared.

**Results:**

- The average pre-presentation score was 37 % and the average post presentation score was 83 %.
- The most poorly answered pre-presentation questions were those about asthma treatment and triggers.
- Knowledge in all areas of the quiz was improved on average after the presentation. The greatest areas of improvement were how to deal with an asthma emergency and misconceptions about asthma.
- Participating medical students gave positive feedback.

**Conclusion:**

**Fig. 8:**
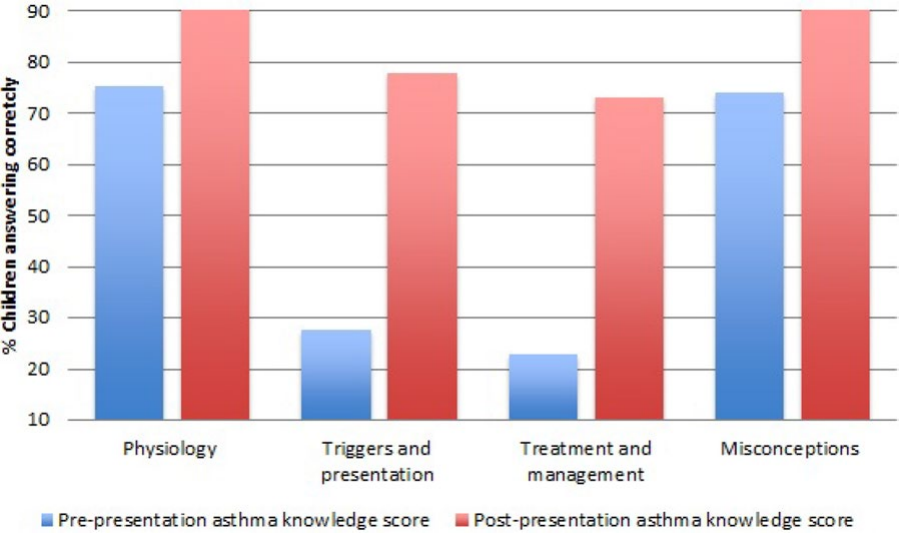
Asthma-scores

- Medical student run education sessions provide a cost-effective and simple method of teaching school children about asthma.
- The improvement in quiz scores before and after the presentation demonstrates the efficacy of the programme.
- Expanding the education programme across the high prevalence asthma areas may help reduce hospital admissions due to asthma (Fig. 8).

### TP02 The prevalence of allergic diseases among 14–15 years old adolescents in two Danish birth cohorts 14 years apart

####  Elisabeth S. Christiansen^1^, Henrik Fomsgaard Kjaer^2^, Esben Eller^1^, Charlotte G. Mørtz^1^, Susanne Halken^2^

##### ^1^Department of Dermatology and Allergy Center, Odense University Hospital, University of Southern Denmark, Odense, Denmark; ^2^Hans Christian Andersen Children’s Hospital, Odense University Hospital, University of Southern Denmark, Odense, Denmark

###### **Correspondence:** Elisabeth S. Christiansen

*Clinical and Translational Allergy* 2016, **6(Suppl 1)**:TP02

**Background:** The prevalence of allergic diseases has increased during the second half of the 20th century in children although the prevalence of asthma seems to have reached a plateau. Using data from two unselected prospective population-based birth cohorts from Odense, Denmark, the Odense85 cohort and the DARC (Danish Allergy Research Center) cohort we aimed to compare the 12 months prevalence of allergic diseases in adolescence over a period of 14 years.

**Methods:** The 15 years follow-up of the Odense85 cohort (n = 276) born in 1985 was carried out in 2000 and the 14 years follow-up of the DARC cohort (n = 562) born 1998–1999 was carried out in 2013–2014. In both cohorts the participant underwent questionnaire-based interviews, physical examinations, skin prick tests, specific IgE testing and spirometry. The diagnostic criteria of asthma, atopic dermatitis and rhinoconjunctivitis were the same in both cohorts.

**Results:** Follow-up rates in both cohorts were high, 78 % (215/276) and 66 % (372/562) respectively. At the 15 years follow-up of the Odense85 cohort the 12 months prevalence of asthma, atopic dermatitis, rhinoconjunctivitis and any allergic diseases was 10.2, 9.5, 16.7 and 28.4 % and at the 14 years follow-up of the DARC cohort the 12 month prevalence of the same diseases were 12.4, 7.3, 25.5, 38.4 % respectively.

**Conclusion:** The prevalence of rhinoconjunctivitis has significantly increased from 2000 to 2014 in Odense, Denmark.

### TP03 Does pattern of sensitisation to phleum pratense change with age? Is it different in children with allergic rhinitis or asthma?

####  Cristina Román India^1^, Ana Moreira Jorge^1^, Loreto González Domínguez^1^, Cristina Muñoz Archidona^2^, Sergio Quevedo Teruel^1^, Teresa Bracamonte Bermejo^1^, Juana Jiménez Jiménez^1^, Luis Echeverría Zudaire^1^

##### ^1^Hospital Universitario Severo Ochoa, Madrid, Spain; ^2^Hospital de Villalba, Madrid, Spain

###### **Correspondence:** Ana Moreira Jorge

*Clinical and Translational Allergy* 2016, **6(Suppl 1)**:TP03

**Background:** Grass pollen is one of the most common seasonal allergens that cause allergic symptoms in children. Many patients with allergic rhinitis, with or without asthma (AR, AR·A, A) are allergic to Timothy grass (*Phleum pratense*). Molecular characterization of Phleum pratense, has revealed different allergen components: rPhl p 1 and rPhl p 5 as species specific, while rPhl p12 (profilin) and the rPhl p7 (polcalcine) are the principal Cross-Reactive Components.

**Patient and methods:** We included a total of 178 children diagnosed AR·A and grass pollen allergy (SPT and IgE results). We evaluated: The frequency of sensitization to allergenic molecules of Phleum pratense and olive pollen (rPhlp1, rPhlp5, rPhlp7, rPhlp12). The correlation of sensitization to major allergens and panallergens and its variation according to age and level of IgE and the presence of AR·A and other pollen concomitant SPT sensitization.

**Results:** Mean age was 9.5 years. 87.64 % had AR, AR + A in 71.5 % and A 86.51 %. rPhlp1, rPhlp5, rPhlp7, rPhlp12 IgE were found in 82.6, 39.88, 6.1 and 10.11 % We performed an age distribution of patients in 3 groups. 1: ≤7 years-old 2:7–12 years-old 3: ≥12 years old. Group 1: Phlp1, 5, 7, 12: 76.8, 39.3, 1.78, 0 %; Group 2: Phlp1, 5, 7, 12: 80.9, 39.7, 7.35, 11.76 %; Group 3: Phlp1, 5, 7, 12: 84.5, 37.9, 8.62, 10.34 %; Older children were more polysensitized (SPT to Olive 88.2 % > weeds 74 % > cupressus 58.2 %). Asthma reacting to rPhlp5 was 41.6 % (ns).

**Conclusion:** rPhlp1 is the most relevant sensitizing allergen in our population, at all ages, while rPhlp5, rPhlp12 and rPhlp7 increase according to age. No differences were found in sensitization to rPhlp5, rPhlp7, rPhlp12 and their IgE levels, by groups. We didn’t find a correlation between rPhlp5 and asthma.

### TP04 Practicalities of prevention of peanut allergy: modelling a national response to LEAP

####  Cathal O’Connor, Jonathan Hourihane

##### University College Cork, Cork, Ireland

###### **Correspondence:** Cathal O’Connor

*Clinical and Translational Allergy* 2016, **6(Suppl 1)**:TP04

**Introduction:** Severe eczema is an early marker for peanut allergy. The LEAP study demonstrated a successful absolute reduction in development of peanut allergy in high-risk infants if peanut was introduced between 4 and 11 months of age in children with severe eczema or egg allergy.

**Aims:** To establish the number of children who would require screening, intervention, and monitoring, if the LEAP protocol were to be implemented in Ireland, based on a cut-off of SCORAD > 40; and to quantify the potential reduction in peanut allergy.

**Methods:** The Central Statistics Office provided data regarding births in Ireland for 2013. There were 68,684 births in Ireland in 2013. The BASELINE birth cohort provided data regarding prevalence and severity of eczema in Irish infants. 18.7 % of Irish children have eczema at six months, of whom 13.5 % (2.4 % overall) had severe eczema (SCORAD > 40).

**Results:** 1647 children (SCORAD > 40) would require initial screening.

175 would be excluded from peanut consumption due to a strongly positive skin prick test (SPT ≥ 3 mm), 225 would have a minimally positive SPT (1–2 mm), and 1247 would have a negative SPT (0 mm). Of the minimally positive SPT group, 55 cases of peanut allergy would be avoided with peanut consumption (24.7 % absolute reduction). Of the negative SPT group, 147 cases of peanut allergy would be avoided with peanut consumption (11.8 % absolute reduction). The total number of peanut allergies that could be avoided in Ireland per annum would be 202.

**Conclusions:** If the LEAP protocol could be fully implemented nationally, it would substantially decrease the incidence of peanut allergy in Ireland. Major new resources for staff, training and facilities would be needed to identify, treat and monitor children during such a public health intervention.

### TP05 Comparison of the influence of sunflower seed oil and skin care lotion on the skin barrier function of newborns: a randomised controlled trial

####  Varvara Kanti^1^, Lena Lünnemann^1^, Günther Malise^1^, Laine Ludriksone^1^, Andrea Stroux^1−2^, Wolfgang Henrich^3^; Michael Abu-Dakn^4^, Ulrike Blume-Peytavi^1^, Natalie Garcia Bartels^1^

##### ^1^Department for Dermatology and Allergy, Clinical Research Center for Hair and Skin Science, Charité-Universitätsmedizin Berlin, Berlin, Germany; ^2^Department of Medical Statistics and Clinical Epidemiology, Charité-Universitätsmedizin Berlin, Berlin, Germany; ^3^Department of Obstetrics, Charité Universitätsmedizin Berlin, Berlin, Germany; ^4^Department of Obstetrics, St. Joseph Clinic Berlin Tempelhof, Berlin, Germany

###### **Correspondence:** Varvara Kanti

*Clinical and Translational Allergy* 2016, **6(Suppl 1)**:TP05

**Background:** Skin care practices influence skin barrier function during the first weeks of life. The application of sunflower seed oil for neonatal skin care has been investigated in previous studies, with controversial findings, varying from lowering mortality rate to decelerating skin barrier maturation in preterm infants.

**Objective:** To investigate the effect of sunflower seed oil compared to a commercially available skin care lotion on the skin barrier function of healthy full-term newborns using standardized, objective, non-invasive methods.

**Methods:** In a prospective, randomized clinical study, 50 healthy full-term newborns aged ≤96 h were randomly assigned to two groups receiving three times per week: topical baby lotion application (group L, n = 22) and topical sunflower seed oil application (group SSO, n = 24). Skin barrier function was evaluated in three anatomical areas (front, abdomen, upper leg) by non-invasive assessment of transepidermal water loss (TEWL), stratum corneum hydration (SCH), sebum and skin-pH at inclusion and after 5 weeks. Clinical skin condition was assessed using the neonatal skin condition score.

**Results:** Both groups showed a significant decrease of skin-pH (p = 0.001) and an increase of SCH (p = 0.001) in all measured anatomical areas after 5 weeks compared to baseline. A significant decrease of TEWL was observed on the forearm in both groups (p = 0.015). TEWL decreased significantly on the leg in group L (p = 0.002) and on the abdomen in group SSO (p = 0.018). Skin condition remained stable.

**Conclusions:** The use of sunflower seed oil showed comparable results to the use of an infant specific emollient in healthy term neonates. Both skin care regimes seem to maintain postnatal maturation of skin barrier function and skin condition.

### TP06 The effect of daily skin care on skin barrier properties in infants with dry skin and risk for atopic dermatitis

####  Varvara Kanti^1^, Lena Lünnemann^1^, Laine Ludriksone^1^, Marianne Schario^1^, Andrea Stroux^1,2^, Ulrike Blume-Peytavi^1^, Natalie Garcia Bartels^1^

##### ^1^Department for Dermatology and Allergy, Clinical Research Center for Hair and Skin Science, Charité-Universitätsmedizin Berlin, Berlin, Germany; ^2^Department of Medical Statistics and Clinical Epidemiology, Charité-Universitätsmedizin Berlin, Berlin, Germany

###### **Correspondence:** Varvara Kanti

*Clinical and Translational Allergy* 2016, **6(Suppl 1)**:TP06

**Background/objectives:** In infants with atopic predisposition, topical emollients may play a pivotal role in improving the skin barrier function and reducing the likelihood of the onset of atopic dermatitis. We aimed to evaluate the effect of daily emollient use on the skin barrier function in infants with dry skin and at risk for developing atopic dermatitis.

**Methods:** A monocentric, prospective open-label clinical study was performed in 25 infants with clinically dry skin and atopic predisposition (Erlangen Atopic Score ≥4). The included infants received a daily combined topical skin care regimen containing ice plant (Mesembryanthemum crystallinum L.) pressed juice over 16 weeks: intensive ice plant cream on the face, ice plant body care lotion on the body and both products on the forearm. Transepidermal water loss (TEWL), stratum corneum hydration (SCH), skin surface pH (pH) and sebum were assessed on the forehead, upper leg and forearm at inclusion (baseline) and at weeks 4, 12 and 16. Skin condition was evaluated using the SCORAD index.

**Results:** In all anatomic regions, SCH increased until week 12 (p = 0.026). TEWL at all investigational sites and pH and sebum on the forearm and leg remained stable between baseline and week 16. On the forehead, pH (p = 0.013) and sebum (p = 0.003) decreased until week 16. SCORAD decreased significantly during the study (p = 0.035).

**Conclusion:** The daily application of a plant-based moisturizing lotion and cream showed an improvement of skin barrier function and clinical skin condition in infants with dry skin and atopic predisposition.

### TP07 Change in sum total aeroallergen skin prick test wheal diameters at 6 months predicts which children will respond to subcutaneous immunotherapy by three years

####  Thorsten Stanley, Nicolien Brandenbarg

##### University of Otago Wellington, Wellington, New Zealand

###### **Correspondence:** Thorsten Stanley

*Clinical and Translational Allergy* 2016, **6(Suppl 1)**:TP07

**Introduction:** Immunotherapy is a proven treatment for allergic rhinoconjunctivitis and moderate asthma in children, but it has proved difficult to predict who will respond, leading to years of therapy for sometimes no benefit. In a small study presented 4 years ago [EAACI Istanbul 2011] we showed serial exhaled nitric oxide levels did not predict response to therapy but change in sum total wheal diameters from a standard range of skin test aeroallergens (house dust mite, dog, cat, grass pollen mix, tree pollen mix and Aspergillus fumigatus (Hollister Stier Ltd Spokane USA) appeared to discriminate responders from non-responders (Ong, S Stanley TV Allergy 66 Suppl.94, 2011 (211-2) We wished to review this observation using a much larger cohort of sixty pediatric patients (age 5–17 years) who had completed subcutaneous immunotherapy (Alustal, Stallergenes, France) for these indications between 2003 and 2015.

**Method:** Retrospective case note review using recently published EAACI consensus guidelines on scoring rhinoconjunctivitis severity to judge response to therapy (Pfaar O et al. Allergy 69: 854–867; 2014).

**Results:** The sum total aeroallergen skin prick test wheal diameters reduced by a mean of 2.24 mm after 6 months maintenance treatment in the children who responded to immunotherapy by 3 years [highly beneficial, or significant improvement] but increased by a mean of 8.3 mm in the non-responders [no benefit, or worse after treatment] in the same time frame. None of the non-responders registered a reduced skin prick wheal diameter (p < 0.015). Interestingly skin test diameters diminished even for allergens for which the child had not received immunotherapy raising the possibility of a class effect of immune suppression for immunotherapy such as has been noted for Timothy Grass with sublingual immunotherapy (Grazax, Alk-Abello A/S Denmark). Reduction in cat allergy after dust mite immunotherapy has recently also been reported.

**Conclusions:** A reduction in sum total wheal diameter after 6 months maintenance immunotherapy appears to predict a successful outcome at 3 years. Alternative therapy should be considered for those children without such a response. It may be possible to develop a multipotent vaccine that allows children to be treated for multiple allergens with only one vaccine.

### TP08 Are mobile apps regarding adrenaline auto-injectors accessed by adolescents for support and education in the community?

####  Alia Boardman, Gary McGreevy, Emily Rodger, Katherine Knight, Victoria Timms, Trisha Taylor, Gemma Scanlan, Roisin Fitzsimons

##### GSTT, London, United Kingdom

###### **Correspondence:** Alia Boardman

*Clinical and Translational Allergy* 2016, **6(Suppl 1)**:TP08

**Objectives:** The teenage years are the transitional stage from childhood to adulthood which heralds physical and psychological changes and issues relating to independence. We wanted to examine risk taking behaviour in this group and evaluate if young people are appropriately educated in the management of allergic reactions. In addition to traditional resources such as health care professionals, there are many virtual support mechanisms available such as included mobile apps and websites which are also available to fulfil this purpose.

**Method:** We surveyed young people who attended outpatient allergy clinics as well as those attending for day case procedures. We aimed to find out what resources they accessed to supplement the teaching they received regarding management of allergic reactions and use of their Adrenaline Auto-Injectors (AAI). The inclusion criteria were: young person aged 12 years and over, who has been prescribed an AAI.

**Results:** Early data suggests that none of the adolescents who completed the questionnaire were aware of an iPhone or Android mobile App which provides education and support relating to the leading brands of AAI.

**Conclusion:** Young people are a group known to engage in risk taking behaviour, therefore any opportunities should be utilised to provide education and offer support. Reasons such as a suitable time, lack of anonymity, appearing to lack knowledge, have all been identified as barriers which prevent young people from accessing traditional support services such as health care professionals. Mobile Apps and websites, in this technical savvy group seem the most likely solution. However, for these to be effectively accessed, it is vital that these services are well publicized, available, easily accessible and friendly. This would allow patient education and empower young people to manage any allergic reactions.

### TP09

**WITHDRAWN**

*Clinical and Translational Allergy* 2016, **6(Suppl 1)**:TP09

### TP10 Prevention of early atopic dermatitis among low-atopy-risk infants by immunoactive prebiotics is not sustained after the first year of life

####  Grüber Christoph^1^, Ulrich Wahn^1^, Margriet van Stuivenberg^2^, Fabio Mosca^3^, Guido Moro^4^, Gaetano Chirico^5^, Christian P. Braegger^6^, Joseph Riedler^7^, Yalcin Yavuz^8^, Günther Boehm^9^

##### ^1^Charité - Universitätsmedizin Berlin, Berlin, Germany; ^2^UMC, Beatrix Children’s Hospital, Groningen, The Netherlands; ^3^Fondazione IRCCS ‘‘Ca’Granda’’ Ospedale Maggiore, Milan, Italy; ^4^University of Milan, Milan, Italy; ^5^Spedali Civili, Brescia, Italy; ^6^University Children’s Hospital, Zurich, Switzerland; ^7^Schwarzach Hospital, Salzburg, Austria; ^8^Danone Research, Utrecht, The Netherlands; ^9^Private practice, Leipzig, Germany

###### **Correspondence:** Grüber Christoph

*Clinical and Translational Allergy* 2016, **6(Suppl 1)**:TP10

The published version of this abstract can be found at [1].

**Reference**

1. Allergy. 2015;70(Suppl 101):280–289. http://onlinelibrary.wiley.com/doi/10.1111/all.12718/epdf.

### TP11

**WITHDRAWN**

*Clinical and Translational Allergy* 2016, **6(Suppl 1)**:TP11

### TP12

**WITHDRAWN**

*Clinical and Translational Allergy* 2016, **6(Suppl 1)**:TP12

### TP13 Treatment with Omalizumab in a 16-year-old Caucasian girl with refractory solar urticaria

####  Stefania Arasi, Giuseppe Crisafulli, Lucia Caminiti, Federica Porcaro, Giovanni Battista Pajno

##### Department of Pediatrics, Allergy Unit, University of Messina, Messina, Italy

###### Correspondence: Stefania Arasi

*Clinical and Translational Allergy* 2016, **6(Suppl 1)**:TP13

**Case report:** Omalizumab is an anti-IgE antibody, approved for severe asthma and chronic idiopathic urticaria. Solar urticaria (SU) is a rare disease, hypothesized to be IgE-mediated, often unresponsive to conventional treatment, burdened by the risk of anaphylactic shock.

We present the case of a 16-year-old Caucasian girl with an 18-month history of severe SU unresponsive to several conventional treatments, dramatically improved after omalizumab.

This adolescent, with a poorly disturbing allergic rhinitis to Graminacee and Alternaria, had a quality of life seriously compromised by SU. After about 15 min from also minimal sunlight exposure, she presented erythema, intense itch, swelling and hives in the sun-exposed areas with complete resolution after about 30 min from the avoidance of sun exposure. Phototest was positive for UVA (>5 J/cm^2^) and UVB (>1.2 J/cm^2^). Negative all tests for investigating other causes of urticaria and photodermatosis. After treatment failure of conventional therapies (triple combination of antihistamines with high-grade sun blockers and association with anti-leukotriene) the patient has been subjected to omalizumab. On the basis of her initial serum IgE level (moderately elevated, 228 IU/mL) and weight (57.7 kg), a calculated dose of 450 mg every 2 weeks of omalizumab was performed. Therapeutic response has been excellent and persistent since the second administration. After 6 months we tried to reduce dose progressively in line with the coming up indication for chronic spontaneous urticaria up to suspension. Now, at 4 month since omalizumab wash-out, SU is still in remission clinically. Phototest is negative both for UVA and UVB. The patient is going on follow-up.

**Conclusions:** This case reports a successful treatment of SU with omalizumab. At this time, it is an experimental treatment but our case could contribute to a new therapeutic scenario for SU and throw light on the potential pharmacologic mechanism of omalizumab in this disease. In fact, it is conceivable that omalizumab, depleting IgE, attenuates the multiple effects of IgE to maintain and enhance mast cells activities, reducing mast-cells-depending inflammatory mechanisms. Further studies are needed.

**Consent to publish**

Written informed consent for publication of this clinical details and/or clinical images was obtained from the patient/parent/guardian/relative of the patient. A copy of the consent form is available for review by the Editor of this journal.

### TP14 Ultra-pure soft water ameliorates skin conditions of adult and child patients with atopic dermatitis

####  Akane Tanaka^1^, Yaei Togawa^2^, Kumiko Oida^1^, Naotomo Kambe^2^, Peter Arkwright^3^, Yosuke Amagai^1^, Naoki Shimojo^2^, Yasunori Sato^4^, Hiroyuki Mochizuki^5^, Hyosun Jang^1^, Saori Ishizaka^1^, Hiroshi Matsuda^1^

##### ^1^Tokyo University of Agriculture and Technology, Tokyo, Japan; ^2^Graduate School of Medicine, Chiba University, Chiba, Japan; ^3^University of Manchester, Manchester, United Kingdom; ^4^Chiba University Hospital, Chiba, Japan; ^5^School of Medicine, Tokai University, Tokyo, Japan

###### **Correspondence:** Akane Tanaka

*Clinical and Translational Allergy* 2016, **6(Suppl 1)**:TP14

**Background:** Water hardness is determined by minerals such as Ca^2+^ and Mg^2+^ that form insoluble precipitates with fatty acid salts including in soap, termed metallic soap. Metallic soap irritates the skin, inducing pruritus and dryness, and thus exacerbating the dermatitis.

**Objective:** The present study investigated the effect of ultra-pure soft water (UPSW) lacking Ca^2+^ and Mg^2+^ on the skin in patients with atopic dermatitis (AD).

**Methods:** A pilot study was conducted and the clinical benefits of UPSW were assessed in adult patients with mild AD. We also performed a randomized, double-blind, placebo-controlled, crossover pilot study to evaluate clinical improvements in skin barrier functions of UPSW in child patients with mild to moderate AD. Finally we tried to find out effects of metallic soap on allergic responses in the animal model of AD, NC/Tnd mice.

**Results:** After 4 weeks of showering with UPSW, the skin of patients with mild AD improved, with a reduction in dryness and itching. In child patients, the eczema area and severity index score showed tendency to improve, though out-in skin transparency and Visual Analog Scale for pruritus and satisfaction with therapy were improved with statistical significance. Application of metallic soap to the skin of tape-stripped NC/Tnd mice induced an allergic inflammatory response with itch and elevation of plasma IgE.

**Conclusion:** UPSW treatment reduced symptoms of AD in adult and child patients. Application of metallic soap onto the barrier disrupted skin induced allergic inflammation in mice. Since UPSW abrogates the formation of metallic soap, washing with UPSW may be beneficial for subjects with skin trouble.

### TP15 Potential adjuvant effect of immunomodulator to improve specific immunotherapy in asthmatic child

####  Wisnu Barlianto, Ery Olivianto, H.M.S. Chandra Kusuma

##### ^1^Department of Pediatrics, Faculty of Medicine, University of Brawijaya, Saiful Anwar Hospital, Malang, Indonesia

###### **Correspondence:** Wisnu Barlianto

*Clinical and Translational Allergy* 2016, **6(Suppl 1)**:TP15

**Background:** Immunotherapy has been proven effective in asthmatic therapy by modulate immune response. The usage of immunomodulators, *Nigella sativa* and probiotics, also has positive effects on allergic disease. Combined immunomodulator and immunotheraphy had shown no improvement in clinical or immunological parameters during building-up phase. The aim of this study is to evaluate the adjuvant effect of immunomodulators on maintenance phase of immunotherapy.

**Method:** Thirty-one mild-asthmatic children were divided into four groups: immunotherapy only, in combination with *Nigella sativa,* with probiotics, and with both *Nigella sativa* and probiotics. Treatment was given for 56 weeks. Th2 (CD4^+^IL-4), Th17 (CD4^+^IL-17^+^), and Treg ((CD4^+^CD25^+^Foxp3^+^), were measured using flowcytometri. Clinical improvement was assessed by Asthma Control Test (ACT). All of the parameters are analyzed with ANOVA test to compare the mean of differences.

**Result:** There was a decreasing trend in the number of Th2 and Th17 after the administration adjuvant immunomodulators compared with the immunotherapy-only group, although it was not statistically significant. These phenomena did not reveal in building-up phase. The number of Treg was likely to be increased in both building-up and maintenance phase. ACT score indicated a significant improvement after immunomodulator administration.

**Conclusion:** Immunomodulator can make improvement in maintenance phase of specific immunotherapy in clinical asthmatic children.

### TP16 How can Component Resolved Diagnosis (CRD) influence in Specific Immunotherapy (SIT) prescription, in a Spanish children population

####  Ana Moreira Jorge^1^, Cristina Román India^1^, Loreto González Domínguez^1^, Cristina Muñoz Archidona^2^, Juana Jiménez Jiménez^1^, Teresa Bracamonte Bermejo^1^, Sergio Quevedo Teruel^1^, Luis Echeverría Zudaire^1^

##### ^1^Hospital Universitario Severo Ochoa, Madrid, Spain; ^2^Hospital de Villalba, Madrid, Spain

###### **Correspondence:** Ana Moreira Jorge

*Clinical and Translational Allergy* 2016, **6(Suppl 1)**:TP16

**Background:** The most important pollens that induce seasonal allergic rhinitis or asthma in pediatrics population in Spain, are grass and olive. Both pollination is overlapped and it may complicate the recognition of real causing agent for an appropriate SIT prescription, even worse if cross reactivity due to common panallergens is present.

**Objective:** Determinate how knowledge of the sensitization pattern to major grass pollen allergens species specific of Phleum pratense (rPhlp1 and rPhlp5) and Olive (Ole e1) might influence SIT prescription.

**Patients and methods:** A total of 155 children with seasonal rhinitis with or without asthma with a positive SPT to Phleum pratense and olive test were included. Determination of serum IgE to Phleum, Olive, rPhlp1, rPhlp5 and Ole e1 was performed. Different positive cut-off points values to IgE were considered, in order to include both grass and olive pollen or not, in a theoretical composition of SIT, pre and post CRD results.

**Results:** A decrease in the number of children reacting to rPhlp1 + rPhlp5 + Olee1 with higher cut off levels of IgE was observed, opposite to an increased of those only positive to Ole e1, Phlp1 or Phlp5. A discordance in the composition of SIT selected before and after the knowledge of CRD, was observed (prior based on clinical data and SPT positive to Phleum and Olive) in 28.4 % of patients (Kappa index: 0.27 p < 0.05). 34.20 % and 54.2 % of children would have been prescribed a different composition of SIT based on cut off point of 0.7 KU/L and 25 % IgEOlee1 level of the total value of rPhlp1.

**Conclusion:** Accuracy of CRD improves SIT prescription in grass and olive polysensitizated patients, changing its composition up to 50 % respect those based only in positive SPT. It is still difficult to establish an appropriate sera IgE positive threshold in case of polysensitization.

### TP17 Mitochondrial dysfunction in food allergy: effects of *L. rhamnosus* GG in a mice model of peanut allergy

####  Rosita Aitoro^1^, Mariapia Mollica^2^, Roberto Berni Canani^1,3^, Giovanna Trinchese^2^, Elena Alfano^2^, Antonio Amoroso^1^, Lorella Paparo^1^, Francesco Amato^1^, Claudio Pirozzi^4^, Antonio Calignano^4^, Rosaria Meli^4^

##### ^1^Department of Translational Medical Science, University of Naples “Federico II”, Naples, Italy; ^2^Department of Biology, University of Naples “Federico II”, Naples, Italy; ^3^CEINGE Advanced Biotechnologies, University of Naples “Federico II”, Naples, Italy; ^4^Department of Pharmacy, University of Naples “Federico II”, Naples, Italy

###### **Correspondence:** Lorella Paparo

*Clinical and Translational Allergy* 2016, **6(Suppl 1)**:TP17

**Objectives and study:** Preliminary findings suggest that mitochondrial dysfunction (MD) could play a role in the pathogenesis of allergic diseases. We aimed to see whether if MD is also present in food allergy, and if it could be modulated by a nutritional intervention with an extensively hydrolyzed casein formula containing the probiotic *L. rhamnosus* GG (LGG).

**Methods:** 4-week-old female C3H/HeOuJ mice were sensitized by oral route with five weekly doses of peanut extracts (6 mg) plus cholera toxin (10 μg) as adjuvant in the presence or absence of a 14-day pre-treatment with an extensively hydrolyzed casein formula containing LGG (EHCF + LGG). Liver mitochondrial respiration rates were evaluated polarographically in isolated mitochondria in the presence of succinate (substrate FAD dependent) or palmitoyl-L-carnitine (fatty acid oxidation) using the Clark electrode, soon after oral food challenge. The carnitine-palmitoyl-transferase (CPT) (rate limiting enzyme of the mitochondrial fatty acid oxidation) and aconitase (oxidative stress marker) activities were measured spectrophotometrically. H_2_O_2_ yield was assayed by following the linear increase in fluorescence (ex 312 nm and em 420 nm) due to the oxidation of homovanillic acid in the presence of horseradish peroxidase.

**Results:** Peanut sensitized mice showed a lower state 3 respiration rate in presence of succinate and decreased fatty acid oxidation than controls (−36 %, p < .05). No difference in CPT activity was observed between these two groups. An increased oxidative stress in sensitized group was proven by inactivation of aconitase activity (−25 %, p < .05) and higher H_2_O_2_ yield (+52 %, p < .05). Pre-treatment with EHCF + LGG induced an improvement of mitochondrial function (+85 %, p < .05) and redox state (−57 %, p < .05), compared to sensitized group. No changes on CPT activity was observed in mice receiving EHCF + LGG.

**Conclusion:** Peanut allergy is characterized by mitochondrial dysfunction and increased oxidative stress. EHCF + LGG efficiently prevents both effects.

### TP18 Prediction of atopic diseases in childhood: elevated blood eosinophils in infancy in a high risk birth cohort

####  Siri Rossberg^1^, Kerstin Gerhold^1^, Kurt Zimmermann^2^, Mohammad Zaino^3^, Thomas Geske^4^, Eckard Hamelmann^5,6^, Susanne Lau^1^

##### ^1^Charité University Hospital, Berlin, Germany; ^2^SymbioPharm GmbH, Herborn, Germany; ^3^Biostatistics, Leipzig, Germany; ^4^TG Medical Services, Berlin, Germany; ^5^Children’s Hospital, Children’s Center Bethel, EvKB, Bielefeld, Germany; ^6^Allergy Center Ruhr, Ruhr-University Bochum, Bochum, Germany

###### **Correspondence:** Siri Rossberg

*Clinical and Translational Allergy* 2016, **6(Suppl 1)**:TP18

The published version of this abstract can be found at [1].

**Reference**

1. Clin Transl Allergy. 2014;4(Suppl 1):P6. http://ctajournal.biomedcentral.com/articles/10.1186/2045-7022-4-S1-P6.

## THEMATIC POSTER SESSION 2: Food allergy—Anaphylaxis (TP19–TP38)

### TP19

**WITHDRAWN**

*Clinical and Translational Allergy* 2016, **6(Suppl 1)**:TP19

### TP20

**WITHDRAWN**

*Clinical and Translational Allergy* 2016, **6(Suppl 1)**:TP20

### TP21 Double-blind provocation tests in non-IgE mediated cow’s milk allergy and the occurrence of placebo reactions

####  Sarah Bogovic, Jochem van den Berg, Chantal Janssen

##### Atrium Medisch Centrum, Heerlen, The Netherlands

###### **Correspondence:** Sarah Bogovic

*Clinical and Translational Allergy* 2016, **6(Suppl 1)**:TP21

**Background:** Double blind provocation tests (DBPTs) are considered the gold diagnostic standard in food allergy. Our cohort of children suspected of cow’s milk allergy (CMA) predominantly showed non-IgE mediated symptoms. Previously published data on incidence of placebo reactions were on patients with Type I food allergic reactions. We hypothesized that the non-specific and subjective nature of symptoms leads to a higher percentage of placebo reactions, especially with placebo administered at the first day. Our aim was to analyze the occurrence of placebo reactions in DBPTs in non-IgE mediated CMA and examine its effect on validity of the supposed gold standard.

**Methods:** Retrospective analysis of data from a single center cohort, prospectively included between July 2011 and August 2014, was performed.

**Results:** 374 DBPTs were performed and resulted in 168 (44.9 %) positive, 197 (52.7 %) negative and 9 inconclusive tests. In the negative outcome group 56/197 children showed placebo reactions, these reactions occurred significantly more often when placebo was administered on the first day. None of these reactions mimicked IgE mediated response. In the positive test group there was no difference in administration of placebo on first or second test day. There was no correlation between order of administration and test result. In 153/197 cases with a negative test cow’s milk was successfully reintroduced.

**Conclusions:** The occurrence of placebo reactions in negative tests (28 %) is higher than previously published. Placebo reactions occurred more often with placebo administered on the first day of testing. The delayed nature of non-IgE symptoms determines an important role for parent observed complaints at home. Therefore DBPTs should be strongly recommended in patients with delayed type of complaints. A seemingly positive reaction, however convincing, should not lead to deviation from blinding. If performed correctly the higher percentage of placebo reactions does not affect validity of DBPT.

### TP22 Gradual introduction of baked egg (BE) in egg allergic patients under 2 years old

####  Angela Claver

##### Servicio Alergia, Hospital Universitario Quirón Dexeus, Barcelona, Spain

###### **Correspondence:** Angela Claver

*Clinical and Translational Allergy* 2016, **6(Suppl 1)**:TP22

**Background:** Our previous results with BE suggest a good tolerance with safety. Also, the regular consumption of baking goods seems to accelerate the tolerance to regular egg.

**Methods:** 25 children were treated with a progressive introduction of BE into their diets. 21 patients had a history of immediate reaction and 4 were sensitized to egg with egg white (EW) sIgE >50 KU/L. All 25 underwent an open food challenge (OFC) with BE performed in 3 different days. Day one: OFC with cookies (brand containing egg). Tolerant patients incorporated cookies and food containing egg traces into their diet. A second challenge with home-breaded chicken was performed a week later. 15 days later they were challenged with a serving size of home-made cake containing 3 eggs. After each steps, regular consumption (cookies, breaded foods and baked goods) was advised for tolerant kids. 24 h mobile contact was given for possible reactions. All children continued regular ingestion and were periodically controlled. Factors including SPT and sIgE levels were used to determine a subsequent challenge with less-heated-egg.

**Results:** All patients tolerated cookies (2–4). 1/25 presented mild anaphylaxis (MA) during OFC with breaded chicken (exercise cofactor); 1 week after, he tolerated it. During next step (cake), a 14 months old girl presented a MA but she continued regular intake up to tolerated omelet. All the remaining patients tolerated BE without reactions. 23/25 patients were successfully challenged with hard-boiled egg, 22/25 were challenged with omelet (19 without problems and 2 with cutaneous symptoms and 1 MA). 12 patients (48 %) came to raw egg ingest. The home phone contact allowed us check only mild symptoms in few patients.

**Comments:** BE is well tolerated and safe even from the diagnosis moment. Thus, dietary avoidance may not be necessary improving the quality of life and minimizing risks in our patients.

### TP23 Randomised controlled trial of SOTI with raw hen’s egg in children with persistent egg allergy I: Safety and efficacy of daily vs. weekly protocols of induction

####  M^a^ Flor Martin-Muñoz^1^, C. Martorell^2^, M. T. Belver^1^, E. Alonso Lebrero^3^, L. Zapatero^3^, V. Fuentes^3^, M. Piqué^4^, A. Plaza^4^, C. Muñoz^5^, A. Martorell^2^, Cristina Blasco^6^, B. Villa^6^, C. Gómez^7^, S. Nevot^7^, J. M. García^8^, L. Echeverria^9^

##### ^1^Hospital La Paz, Madrid, Spain; ^2^General Hospital, Valencia, Spain; ^3^Hospital Gregorio Marañón, Madrid, Spain; ^4^Hospital San Juan de Dios, Barcelona, Spain; ^5^Hospital Carlos Haya, Málaga, Spain; ^6^Hospital Vall Hebrón, Barcelona, Spain; ^7^Hospital Fundación Althaia San Juan de Dios, Barcelona, Spain; ^8^Hospital de Cruces, Bilbao, Spain; ^9^Hospital Severo Ochoa Leganés, Madrid, Spain

###### **Correspondence:** M^a^ Flor Martin-Muñoz

*Clinical and Translational Allergy* 2016, **6(Suppl 1)**:TP23

**Background:** The best, safest and most effective clinical protocol of egg SOTI has not been established. Our aim was to compare 2 egg desensitization protocols to improve efficiency, safety and easy to perform SOTI.

**Methods:** 101 children (5–9 years old) with egg allergy proved by DBCCP were randomized to 25 controls, to follow an egg free diet, and 76 to follow SOTI with pasteurized white egg (PWE) during 1 year. The initial dose-escalation phase started with a water solution (1/1000) 1 ml, doubling dose every 30 min until allergic symptoms appeared (threshold dose). Then, patients followed 2 different protocols of build-up phase: I (5 % daily increases at home and 30 % weekly in the hospital) *vs.* II (30 % weekly increases in the hospital) until reaching the final programmed dose of 30 ml (3.3 g protein). At T12 tolerance was assessed in control group by a DBCCP with PWE. Then, patients in group C with demonstrated persistent egg allergy followed SOTI if parents and they want. We value adverse reactions and analyse the associated factors to safety and efficacy.

**Results:** At T12, 3 control patients and 9 of SOTI group had withdrawn; 2/22 (18 %) of control group passed the egg DBCCP and were considered to be tolerant vs 64/76 (84 %) desensitized of SOTI group (p < 0.001). Then, 10 patients of control group decided follow SOTI. Finally, twenty-six children had followed pattern I and 60 pattern II of desensitization, 12 did not reach total desensitization (3.84 % protocol I *vs* 15.62 % protocol II); 25/26 I protocol *vs* 49/60 II protocol reached 30 ml in 70.12 ± 29.045 I *vs* 149.80 ± 101.365 days pattern II) (p < 0.001). Dropouts and adverse reactions were associated to protocol II, allergic asthma and atopic dermatitis (p < 0.05).

**Conclusions:** A daily protocol of induction with minor increases is more effective and safer.

### TP24 Randomised controlled trial of SOTI with raw hen’s egg in children with persistent egg allergy II: a randomised controlled trial to study a safer, more effective and easy to perform maintenance (daily vs. every two days) pattern of egg SOTI

####  M^a^ Flor Martin-Muñoz^1^, C. Martorell^2^, M. T. Belver^1^, E. Alonso Lebrero^3^, L. Zapatero^3^, V. Fuentes^3^, M. Piqué^4^, A. Plaza^4^, C. Muñoz^5^, A. Martorell^2^, Cristina Blasco^6^, B. Villa^6^, C. Gómez^7^, S. Nevot^7^, J. M. García^8^, L. Echeverria^9^

##### ^1^Hospital La Paz, Madrid, Spain; ^2^General Hospital, Valencia, Spain; ^3^Hospital Gregorio Marañón, Madrid, Spain; ^4^Hospital San Juan de Dios, Barcelona, Spain; ^5^Hospital Carlos Haya, Málaga, Spain; ^6^Hospital Vall Hebrón, Barcelona, Spain; ^7^Hospital Fundación Althaia San Juan de Dios, Barcelona, Spain; ^8^Hospital de Cruces, Bilbao, Spain; ^9^Hospital Severo Ochoa Leganés, Madrid, Spain

###### **Correspondence:** M^a^ Flor Martin-Muñoz

*Clinical and Translational Allergy* 2016, **6(Suppl 1)**:TP24

**Background:** There are described some different maintenance patterns of SOTI, but has not been established what are better. Our aim was investigate the more effective, safer and reasonably easy to perform maintenance (daily *vs.* every two days) pattern.

**Methods:** 101 children (5–11 years of age) with persistent egg allergy proved by DBCCP were randomized to 25 controls to follow an egg free diet or 76 to follow SOTI with pasteurized white egg (PWE) during 1 year, 38 group A (GA) and 38 group B (GB). Once children with SOTI reached the final programmed dose of 30 ml (3.3 g protein), followed 2 different maintenance patterns: GA 30 ml per day and GB 30 ml every 2 day. At 12 months (T12), and 6 months after discontinuing treatment and following diet with egg (T18), a DBCCP with PWE was carried out. We assessed the influence of maintenance patterns and the consumption of egg on the efficacy and safety of SOTI.

**Results:** At T12 96 % patients that reached desensitization tolerated cooked egg; 31/32 (96.8 %) AG, 31/32 (98.8 %) BG, 8/10 (80 %) CG.

At T18, 72/74 (98.6 %) patients that reached total desensitization eat cooked egg without problems (93.9 % AG, 86.8 % BG and 80.0 % CG). At this time 54/74 (72.9 %) tolerated 3.3 g protein of PWE in the DBCCP (27/29 patients in AG *vs* 22/27 in BG and 5/8 in CG (p < 0.01). Egg consumption during T12-T18 was related to PWE tolerance in DBCCP (p < 0.01). There were no differences in adverse reactions of A *vs* B maintenance protocol.

**Conclusion:** A daily is a more effective than an every two days protocol of maintenance and did not related to adverse reactions.

### TP25 Determining the safety of baked egg home reintroduction for children with mild egg allergy

####  Brenda DeWitt^1^, Judith Holloway^2^, Donald Hodge^1^

##### ^1^Leeds Children’s Hospital, Leeds, United Kingdom; ^2^University of Southampton, Southampton, United Kingdom

###### **Correspondence:** Brenda DeWitt

*Clinical and Translational Allergy* 2016, **6(Suppl 1)**:TP25

**Background:** Egg allergy is one of the most common food allergies in children, but many children with IgE-mediated egg allergy can tolerate baked egg in their diet. The British Society of Allergy and Clinical Immunology (BSACI) guidelines recommend home introduction of baked egg for children with mild egg allergy. Home reintroduction is controversial, as anaphylaxis to baked egg in children with mild egg allergy has been observed.

**Objectives:** To determine whether the BSACI guidelines for home reintroduction of baked egg to children with mild egg allergy could be safely implemented at the Leeds Children’s Hospital.

**Methods:** A retrospective cohort case note analysis of baked egg challenges over an 18-month period was conducted. Challenge outcome, severity of reactions, co-morbidities and markers of atopic disease were assessed to see if challenge outcome could be predicted. Subgroup analysis was performed for low and high-risk subjects.

**Main Results:** Case notes of 91 children who had a baked egg OFC were reviewed. 21 subjects had a positive challenge and 70 subjects had a negative challenge resulting in 77 % of the participants successfully completing the baked egg food challenge. Symptoms experienced during a positive challenge were predominantly cutaneous and gastrointestinal. No subject had respiratory difficulties or anaphylaxis to baked egg.

Age (p = 0.021), asthma (p = 0.002), eczema (p = 0.048), peanut allergy (p = 0.006) and total IgE (p = 0.001) were found to be significant in relation to a positive outcome. Subgroup analysis further supported significance of peanut allergy and age in relation to positive challenge. High-risk subjects, identified as egg allergic with asthma or complex allergy, were more likely to have a positive challenge (p = 0.007).

**Conclusions:** BSACI recommendations for home introduction of baked egg are suggested to be safe. However, given concerns about safety and reported instances of anaphylaxis to baked egg, supervised challenged are recommended.

### TP26 Demographics, investigations and patterns of sensitisation in children with oral allergy syndrome in a London Teaching Hospital

#### Sian Ludman^1^, Merhdad Jafari-Mamaghani^2^, Rosemary Ebling^3^, Adam T. Fox^4^, Gideon Lack^4^, George Du Toit^4^

##### ^1^St. Mary’s Hospital, London, United Kingdom; ^2^ThermoFisher Scientific, Uppsala, Sweden; ^3^Immunology Laboratory, King’s College Hospital, London, United Kingdom; ^4^St. Thomas’ Hospital, London, United Kingdom

###### **Correspondence:** Sian Ludman

*Clinical and Translational Allergy* 2016, **6(Suppl 1)**:TP26

The published version of this abstract can be found at [1].

**Reference**

- Ludman S, Jafari-Mamaghani M, Ebling R, Fox AT, Lack G, Du Toit G. Pollen food syndrome amongst children with seasonal allergic rhinitis attending allergy clinic. Pediatr Allergy Immunol. 2016;27:134–140.

### TP27 Airborne peanut challenge in children: allergic reactions are rare

####  Sofia Lovén Björkman, Caroline Nilsson, Natalia Ballardini

##### Sachs Children and Youth Hospital, Stockholm, Sweden

###### **Correspondence:** Sofia Lovén Björkman

*Clinical and Translational Allergy* 2016, **6(Suppl 1)**:TP27

**Background:** Allergic reactions to food allergens commonly occur after oral ingestion, although reactions after inhalation have also been reported, which causes fear in many patients.

**Aim:** To investigate airborne reactions for peanuts in children.

**Methods:** Eighty-four children were referred to the Children and Youth Hospital in Stockholm for an airborne peanut challenge 2008–2014. The median age was 10 years (range 2–18). At the airborne peanut challenge the children were exposed to 300 g of peanuts in a bowl for 30 min in a small room and observed two hours after.

**Results:** Among the 84 children only 2 (2.4 %) reacted at challenge. A 12 year old boy with IgE level 58 kU/L to peanut and 37 kU/L to Ara h 2, reacted with rhinoconjunctivitis only. A 10 year old girl with IgE level 700 kU/l to peanut and 115kU/l to Ara h 2, reacted with itching of the mouth and rhinoconjunctivitis. The boy had asthma but did not develop any asthmatic symptom during the challenge. Neither patient required any medical treatment during or after the challenge. The median IgE level in all children was 100 kU/l (range 0.1–3000 kU/l) to peanut and 55 kU/l (range 0.1–810) to Ara h 2. There was no significant difference for the median IgE level to peanut (379 and 100 kU/l respectively) or to Ara h 2 (76 and 55 kU/l respectively) between the group of patients with objective allergic reactions from the group of patients without reactions.

**Conclusion:** Very few patients reacted at airborne peanut challenge and the reactions observed were mild. Neither an asthma diagnosis nor high levels of IgE to peanut or Arah2 could predict reactions. Allergic reactions to airborne peanut allergens exist but are rare.

### TP28 The nutty question on Pediatric Wards: to be or “nut” to be?

####  Supriyo Basu, Jenny Hallet, Jyothi Srinivas

##### Milton Keynes University Hospital NHS Foundation Trust, Milton Keynes, United Kingdom

###### **Correspondence:** Supriyo Basu

*Clinical and Translational Allergy* 2016, **6(Suppl 1)**:TP28

The published version of this abstract can be found at [1].

**Reference**

1. Abstracts of the 2015 Annual Meeting 4–6 September 2015 Telford International Centre UK. Clin Exp Allergy. 45:1876–1913. doi:10.1111/cea.12656.

### TP29

**WITHDRAWN**

*Clinical and Translational Allergy* 2016, **6(Suppl 1)**:TP29

### TP30

**WITHDRAWN**

*Clinical and Translational Allergy* 2016, **6(Suppl 1)**:TP30

### TP31 Allergy education in nursery schools

####  Hazel Stringer, Nicola Jay

##### Sheffield Children’s Hospital, Sheffield, United Kingdom

###### **Correspondence:** Hazel Stringer

*Clinical and Translational Allergy* 2016, **6(Suppl 1)**:TP31

**Objective:** We wished to determine the number of children currently in nurseries in Sheffield with food allergy, how the nurseries manage the children’s allergies, who provides staff training and is the training adequate.

**Methods:** We achieved this by asking 5 questions which included the number the children in nursery with food allergies with or without an adrenaline auto-injector (AAI), number of staff trained in allergy treatment and use of an AAI and the training provider.

**Results:** Ninety-eight (98) questionnaires were returned with a return rate of 60 %.

Most nurseries had at least one child with a food allergy and only 13 % didn’t have any child with a food allergy. Just over half of nurseries had less than 5 children with food allergies, a quarter of nurseries had children with an AAI and no nursery had more than 3 children with one. In about one-third of nurseries all staff had some form of allergy training, 22 % had no staff trained and 8 % were unsure. Nearly three quarters of nurseries had some staff trained to use an AAI but 20 % had no staff trained and 6 % were unsure.

A variety of training providers were used with about a quarter using NHS or school nurses, a third using first aid or online training only, and another third had no training or were unsure where to access training.

**Conclusions:** It is essential that nursery staff are fully trained in the care of children with food allergies. Education plays a key role in the management of allergies in the nursery setting. The results of our questionnaire show that the quality of allergy education for nurseries in Sheffield is inconsistent and immeasurable. Standardising the delivery of allergy education will help to ensure the safety of these preschool children.

### TP32 Food allergy in the first year of life

####  Tânia Lopes, Cristina Madureira, Filipa Almeida, Susana Lopes, Paula Fonseca, Clara Vieira, Fernanda Carvalho

##### Centro Hospitalar do Médio Ave, Santo Tirso, Portugal

###### **Correspondence:** Tânia Lopes

*Clinical and Translational Allergy* 2016, **6(Suppl 1)**:TP32

**Introduction:** Food allergy is an adverse health effect arising from a specific immune response to a given food. This immune response can be IgE-mediated or non IgE-mediated. Apart from the nine major food allergens, any food can trigger an allergic response.

The aim of this study is to draw attention to less frequent food allergy cases that forced a large degree of suspicion.

**Methods:** Five infants with less frequent food allergy followed in outpatients of our Hospital were selected. The medical files were reviewed and the following variables were evaluated: gender, age, personal and family history of atopy, food allergen and allergic manifestations, specific and total IgE, skin prick test, oral food challenge and tolerance, co-morbidities and others allergies.

**Results:** Five infants were selected, three males. The mean age at onset of symptoms was five months. The implied foods were: potato, corn, wheat and banana. In two cases (wheat, corn) the initial manifestation was anaphylaxis and in other three (potato, corn, banana) vomiting and prostration. In the latter group research revealed a non IgE-mediated allergic reaction, most likely food protein that induced enterocolitis syndrome. In these cases food challenge carried to confirm the diagnosis.

**Conclusions:** Any food can induce a severe allergic reaction such as anaphylaxis. In the first year of life with food diversification it is necessary to be aware of any symptoms related to food intake.

Through a careful food history, physical examination and interpretation of specific tests, the food allergen can be found. In all our cases, the food allergen was mixed with other food which led to the performance of several tests and food challenges for its identification.

Food avoidance is the only treatment so it’s important to alert the medical community that any food can induce allergy preventing delay in diagnosis.

### TP33 Prevalence and geographic distribution of oral allergy syndrome in Italian children: a multicenter study

####  Carla Mastrorilli^1^, Carlo Caffarelli^1^, Riccardo Asero^2^, Salvatore Tripodi^3^, Arianna Dondi^4^, Gianpaolo Ricci^5^, Carlotta Povesi Dascola^1^, Elisabetta Calamelli^5^, Francesca Cipriani^5^, Andrea Di Rienzo Businco^6^, Annamaria Bianchi^7^, Paolo Candelotti^8^, Tullio Frediani^9^, Carmen Verga^10^, Paolo Maria Matricardi^11^

##### ^1^Pediatric Department, Unit of Allergy and Immunology in Evolutive Age, Clinical and Experimental Medicine, University of Parma, Parma, Italy; ^2^Allergology Service, San Carlo Clinic, Paderno Dugnano, Italy; ^3^Pediatric Department and Pediatric Allergology Unit, Sandro Pertini Hospital, Rome, Italy; ^4^Pediatric Unit, Department for Mother and Child, Ramazzini Hospital, Carpi, Italy; ^5^Pediatric Unit, Department of Medical and Surgical Sciences, University of Bologna, Bologna, Italy; ^6^Centro per la Prevenzione, Diagnosi e Cura delle Malattie Allergiche e Otorinolaringoiatriche, Rome, Italy; ^7^Operative Complex Unit of Pediatrics and Neonatal Patology, Mazzoni Hospital, Ascoli Piceno, Italy; ^8^Pediatric Unit, Mazzoni Hospital, Ascoli Piceno, Italy; ^9^Pediatric Department, La Sapienza University, Rome, Italy; ^10^Azienda Sanitaria Locale Salerno, Salerno, Italy; ^11^Department of Pediatric Pneumology and Immunology, Charité Medical University, Berlin, Germany

###### **Correspondence:** Carla Mastrorilli

*Clinical and Translational Allergy* 2016, **6(Suppl 1)**:TP33

**Background:** Oral allergy syndrome (OAS) is a common adverse reaction to the ingestion of pollen-related foods in patients with pollen-induced rhinoconjunctivitis.

**Objectives:** To investigate prevalence, risk factors and clinical relevance of oral allergy syndrome in a large cohort of Italian children living in different geographic areas.

**Methods:** This cross-sectional study assessed 1271 children, aged 4–18 years, with pollen-related seasonal allergic rhinoconjunctivitis, who were enrolled by 16 pediatric outpatient clinics throughout Italy. Clinical data were assessed using a standardized questionnaire. Skin prick tests (SPTs) with commercial pollen and food extracts as well as profilin-enriched extract of date palm pollen were performed. Total and specific IgE to major pollen and food related allergenic molecules as well as to the pan-allergens Phl p 12 (profilin), Bet v 1 (PR-10 protein) and Pru p 3 (lipid transfer protein) were tested by ImmunoCAP FEIA.

**Results:** Oral allergy syndrome was observed in 300 patients (23.6 %), with a higher frequency in Northern Italy. The most commonly reported foods causing OAS were kiwi (38.0 %), peach (28.3 %), apple (24.0 %), hazelnut (22.0 %) and peanut (20.3 %). Symptoms were associated with female gender, at least one atopic parent, an OAS-affected mother and longer duration of allergic rhinoconjunctivitis. Other comorbidities (asthma, anaphylaxis, urticaria and/or angioedema, atopic dermatitis, gastrointestinal symptoms) as well as positive SPT to date palm profilin, were more frequently observed in children with OAS. More than 76 % of the patients with OAS produced specific IgE to at least one group of pan-allergens. Children with OAS had an increased risk for IgE sensitization to pan-allergens, respectively 32 % were sensitized to Phl p 12, 36 % to Bet v 1 and 43 % to Pru p 3.

**Conclusions:** Oral allergy syndrome in pollen-allergic children is more common than previously reported. Geographic differences were described with a higher frequency in Northern Italy. Children reporting OAS showed significantly longer rhinoconjunctivitis duration. Most of the patients with OAS sensitized to at least one group of pan-allergens.

### TP34 Are common standardised allergen extracts used in skin test enough in the diagnosis of nuts allergy?

####  Cristina Muñoz Archidona^1^, Loreto González Domínguez^2^, Ana Moreira Jorge^2^, Sergio Quevedo Teruel^2^, Teresa Bracamonte Bermejo^2^, Miriam Castillo Fernández^3^, Fernando Pineda de la Losa^3^, Luis Ángel Echeverría Zudaire^2^

##### ^1^Hospital de Villalba, Madrid, Spain; ^2^Hospital Universitario Severo Ochoa, Madrid, Spain; ^3^Application Department Diater, Madrid, Spain

###### **Correspondence:** Cristina Muñoz Archidona

*Clinical and Translational Allergy* 2016, **6(Suppl 1)**:TP34

**Background:** The use of standardized allergen extracts in skin prick tests (SPT) is in general safe and allows and effective diagnosis and treatment of allergic disease. However, in extraction procedures used for nuts, defatting steps are included. As a result, water soluble proteins (WPT) are present in diagnostic reagents whereas many oil body associated proteins (OAPs) are absent, so a number of nut-allergic patients could not be correctly diagnosed.

**Methods:** We evaluated 61 children with a convincing history of peanut or walnut ingestion related symptoms. WPT and OAPs of peanut and walnut were determined through SPT and allergen-specific IgE antibodies (sIgE). An SPT was considered positive if the ratio to histamine wheal was ≥0.5. We want to determine how many patients with WPT-SPT negative, showed an OAPs-SPT positive.

**Results:** Peanut: 8 patients had a WPT-SPT negative. One of them presented an OAPs-SPT positive. Walnut: 9 children with WPT-SPT negative. 55.5 % of them (5 patients) showed an OAPs-SPT positive. Patients with WPT-SPT negative and OAPs-SPT positive: 66.6 % had asthma and 100 % family history of atopy. Age of symptom’s onset: 29 months (median). Reactions occurred within 30 min after ingestion (1 patient after walnut contact). 83.3 % presented skin symptoms, 33.3 % respiratory and 50 % digestive symptoms. Reaction’s severity: 4 mild, 2 moderate. No differences were found in reaction’s severity depending on OAPs sensitization. 50 % were sensitized to other nut (2 hazelnut and 1 cashew nut). Total IgE: 78.8kU/l (median). Case with peanut allergy: peanut-WPT-sIgE 0.81kU/l, peanut-OAP-sIgE 0.76kU/l. Walnut-allergy patients: walnut-WPT-sIgE 3.8kU/l, walnut-OAP-sIgE 1kU/l.

**Conclusions:** The deffating procedures performed in obtaining standardized reagents may be the cause of false negative SPT in patients with nut allergy. We consider very important to develop adequate allergen extracts to improve nuts allergy diagnosis.

### TP35 Evaluation of IgE sensitisation in children with allergic proctocolitis and its relationship to atopic dermatitis

####  Despina Mermiri, Paraskevi Korovessi, Skevi Tiliakou, Evaggelia Tavoulari, Kalliopi-Maria Moraiti, Fotini Giannoula, Athina Papadopoulou

##### Allergology and Respiratory Unit, Penteli’s Children Hospital, Athens, Greece

###### **Correspondence:** Paraskevi Korovessi

*Clinical and Translational Allergy* 2016, **6(Suppl 1)**:TP35

**Background:** Allergic Proctocolitis (AP) is a disease of early infancy that represents the benign end of, non-IgE-mediated to Cow’s Milk Protein (CMP), allergy spectrum. The aim of the present study was to evaluate IgE sensitisation to CMP in infants with AP, its relation to the natural course of the disease and the presence of atopic dermatitis (AD).

**Methods:** We retrospectively reviewed the medical records of 73 infants (60.3 % boys, mean age 2.3 ± 1.84 months, 84 % exclusively breastfed), consistent with AP, in a 2 year period. IgE sensitisation (SPTs, CAP), history of AD and results of open food challenges (OFC) to milk were recorded.

**Results:** Positive CMP SPTs were found in 11 infants (15 %) out of which 45 % were also sensitised to other food proteins (egg, fish, wheat). Co- morbidities in the form of AD and gastroesophageal reflux (GER) where found in 27 and 14 % respectively. IgE sensitisation was significantly associated with the presence of AD (p = 0.009), whereas there was no relation between IgE sensitisation neither with breastfeeding nor with GER.

During re-introduction of CMP, 2 OFC turned out positive with symptoms related to IgE-mediated milk allergy (2.7 %) both children had high levels of sensitization.

**Conclusion:** Although AP is classically considered a non IgE mediated allergy to CMP, a significant percentage of infants can present with IgE sensitization. The sensitization is related to AD either genuinely or false positively in the context of barrier dysfunction.

Allergic work-up is important before milk re-introduction in infants with AP as there is a small but existent risk of IgE-mediated reaction. Further studies are needed to clarify the role of IgE sensitization in determining the natural history of AP.

### TP36 Food allergy in children: are we managing them appropriately in the Emergency Department?

#### Wan Jean Tee, Samir Deiratany, Raymond Seedhoo, Roisin McNamara, Ike Okafor

##### Children University Hospital, Temple Street, Dublin, Ireland

###### **Correspondence:** Wan Jean Tee

*Clinical and Translational Allergy* 2016, **6(Suppl 1)**:TP36

**Background:** Poor management of food allergy in children during their first presentation resulted in multiple re-presentation to the emergency department (ED).

**Aim:** To evaluate the management and diagnosis of acute food allergic reaction presenting to a busy Pediatric ED, Dublin, Ireland.  

**Methods:** 221 patients presented to Temple Street Children University Hospital, from 1st January 2013–31st December 2013 with a diagnosis of allergy. Data were collected using a designated audit tool by extracting information from our Electronic Patient System (Symphony).

**Results:** 61 patients were diagnosed with food allergy from 1st January 2013-31st December 2013. Average age of presentation was 45 months old. 39.3 % of patients presented with food allergy had background history of eczema, asthma or allergic rhinitis. 45 (73.77 %) patients were newly diagnosed, while 16 (26.22 %) patients were known to have specific food allergy. Only 1(1.63 %) patient presented with an anaphylactic reaction. The rest of the patients presented with facial swelling (49.1 %), urticarial rash (36.0 %), facial swelling and rash (9.8 %), wheeze (1.6 %), bloody diarrhoea (1.6 %) and lump in a throat (1.6 %). Oral anti-histamines were given to 68.9 % (n = 42) of the patients with acute allergic reaction while 22.9 % (n = 14) received a combination of oral anti histamine and oral steroids. 51 (83.6 %) patients were kept for observation more than 2 h. Only 14 patients (31.1 %) were referred for a further consultation with an allergist or general pediatrician. One patient was referred to the dietician.

**Discussion:** Children with suspected with food allergy reaction in ED should be referred for further tests and specialised management. However, only a third of them were referred to allergist or general pediatrician. There was also lack of information-giving to parents and dietician follow up. Appropriate management prevents re-presentation and improved the quality of care for children with food allergy.

### TP37 Importance of oil body associated allergenic proteins in nuts suspected allergy children

####  Loreto González Domínguez^1^, Ana Moreira Jorge^1^, Cristina Muñoz Archidona^2^, Teresa Bracamonte Bermejo^1^, Sergio Quevedo Teruel^1^, Fernando Pineda de la Losa^3^, Miriam Castillo Fernández^3^, Luis Ángel Echeverría Zudaire^1^

##### ^1^Hospital Universitario Severo Ochoa, Madrid, Spain; ^2^Hospital de Villalba, Madrid, Spain; ^3^Application Department Diater, Madrid, Spain

###### **Correspondence:** Loreto González Domínguez

*Clinical and Translational Allergy* 2016, **6(Suppl 1)**:TP37

**Background:** It has defined Oil body-associated proteins (OAPs) as allergenic proteins found in peanut, almond, walnut and hazelnut. The aim of this study is to show a pattern of IgE recognition to OAPs in a pediatric population with suspected allergy to these nuts.

**Methods:** A total of 90 children were selected. Skin prick test (SPT) and specific IgE, both to OAPs and water soluble proteins (WSP), to peanut, almond, walnut and hazelnut were tested. OAPs and WSP were analyzed by SDS PAGE under reducing conditions. Immuno-BLOT with the pooled sera was done and biotiniled OAPs from almond, hazelnut, peanut, walnut were coupled to Streptavidin-InmunoCAP solid phase.

**Fig. 9:**
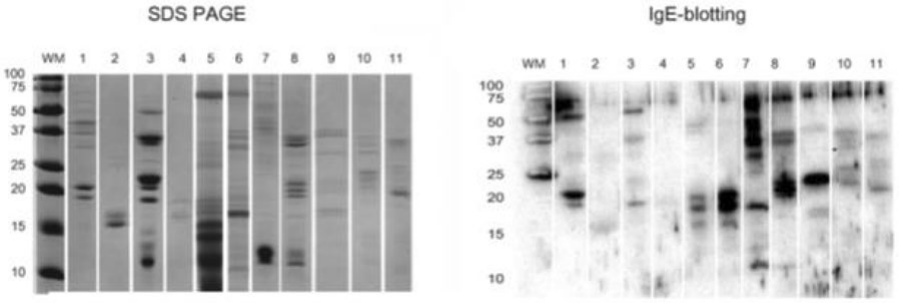
.

**Results:** Positive SPT OAPs: peanut 23.3 %, almond 8.9 %, walnut 45.6 %, hazelnut 25.6 %. OAPs and WSP from the sources described were mainly detected around 15–25 kDa, but WSP around 50 kDa from almond, hazelnut, peanut and WSP around 30 to 65 kDa from walnut. Specific IgE from OAPs were positive for peanut 61 patients (67 %), almond 34 (37 %), walnut 58 (64 %) and hazelnut 47(52 %), considering IgE cut off point >0.1 KU/L.). Proteins around 15–25 kDa were recognized in OAPs and WSP, but WSP around 50 kDa from almond, hazelnut, peanut and WSP around 30–65 kDa from walnut (Fig. 9).

**Conclusions:** More than 50 % of children with suspected nuts allergy are sensitized to OAPs. Our data show that peanut and walnut, are the most implicated. Some of the proteins around 15–25 kDa recognized in OAPs, may correspond to oleosin. These potential allergenic sources should be included in commercial SPT reagents.

### TP38 Practical application of basophil activation test in children with food allergy

####  Ekaterina Khaleva^1^, Gennady Novic^1^, Natalia Bychkova^2^

##### ^1^Saint Petersburg State Pediatric Medical University, St. Petersburg, Russia; ^2^Nikiforov Russian Center of Emergency and Radiation Medicine, St. Petersburg, Russia

###### **Correspondence:** Ekaterina Khaleva 

*Clinical and Translational Allergy* 2016, **6(Suppl 1)**:TP38

The published version of this abstract can be found at [1].

**Reference**

1. Poster Session Group II. Allergy. 2014;69:326–453. doi: 10.1111/all.12477.

## THEMATIC POSTER SESSION 3: Asthma (TP39–TP57)

### TP39 Effect of corticosteroid therapy upon serum magnesium level in chronic asthmatic children

#### Amany Abd Al-Aziz, Amany Fatouh, Ayat Motawie, Eman El Bostany, Amr Ibrahim

##### National Research Centre, Cairo, Egypt

###### **Correspondence:** Amany Abd Al-Aziz

*Clinical and Translational Allergy* 2016, **6(Suppl 1)**:TP39

The published version of this abstract can be found at [1].

**Reference**

1. Int J Food Saf, Nutrition and Public Health (IJFSNPH). 2009;2(2).

### TP40 ADAM33 in Bulgarian children with asthma

####  Guergana Petrova^1^, Dimitrinka Miteva^1^, Snezhina Lazova^1^, Penka Perenovska^1^, Sylvia Andonova^2^, Alexey Savov^2^

##### ^1^Pediatric Cilinic, University Hospital, Sofia, Bulgaria; ^2^National Genetic Laboratory, Obstetric and Genecology Hospital “Maichin dom”, Sofia, Bulgaria

###### **Correspondence:** Guergana Petrova

*Clinical and Translational Allergy* 2016, **6(Suppl 1)**:TP40

The published version of this abstract can be found at [1].

**Reference**

1. Allergy. 2015; 70(Suppl. 101):290–392. http://onlinelibrary.wiley.com/doi/10.1111/all.12719/epdf.

### TP41

**WITHDRAWN**

*Clinical and Translational Allergy* 2016, **6(Suppl 1)**:TP41

### TP42 The impact of vitamin D serum levels in asthma and allergic rhinitis

####  Maria Zoto^1^, Marialena Kyriakakou^2^, Paraskevi Xepapadaki^2^, Nikolaos G. Papadopoulos^2^

##### ^1^Hygeia Hospital, Tirana, Albania; ^2^Allergy Unit, 2nd Pediatric Clinic, University of Athens, Athens, Greece

###### **Correspondence:** Maria Zoto

*Clinical and Translational Allergy* 2016, **6(Suppl 1)**:TP42

**Background:** The relationship between vitamin D status and asthma has been the subject of several studies in the last decade. The aim of the present study was to investigate the relationship between serum vitamin D levels and indices of asthma control, asthma severity and quality of life in children with asthma and comorbidities.

**Methods:** The study included 29 children who were diagnosed with asthma according to the GINA criteria and who did not receive immunotherapy for their allergy. Serum 25-hydroxyvitamin D [25(OH) D] levels were measured in blood samples collected at first enrollment in summer and in reevaluation 6 months later. Subjects were categorized into deficient (<20 ng/ml), insufficient (21–30 ng/ml) and sufficient (>30 ng/m). The asthma control, asthma severity and quality of life were evaluated using the ACT (asthma control test), GINA criteria and PedsQL (pediatric quality of life questionnaire). Atopy biomarkers and spirometry were also measured for analysis.

**Results:** 6.9 % percent of subjects were deficient and 27.6 % insufficient in serum vitamin D at baseline. The study revealed a highly decrease in vitamin D levels from 38.4 ng/ml to 22.1 ng/ml in the winter, (p < 0.001). None of the children had vitamin D sufficiency during winter. Asthma severity and asthma quality of life showed no significant association with serum 25(OH) D levels. We found an association between asthma control and vitamin D levels (p = 0.0665) in summer. Furthermore, there is an association between 25(OH) D and severity of comorbit rhinitis (p = 0.03).

**Conclusions:** Vitamin D deficiency and insufficiency are common in asthmatic children. Lower serum levels are prevalent in winter season. Vitamin D status is associated with asthma control and severity of rhinitis.

### TP43 Life-threatening, first reported, paradoxical bronchospasm after nebulised Salbutamol in a 10 year old child

####  Paraskevi Korovessi^1^, Mariza Vassilopoulou^2^, Athina Balaska^3^, Lambros Banos^3^, Stavroula Kostaridou^3^, Despina Mermiri^1^

##### ^1^Allergology and Respiratory Unit, Penteli’s Children Hospital, Athens, Greece; ^2^Intensive Care Unit, Penteli’s Children Hospital, Athens, Greece; ^3^Pediatric Department, Penteli’s Children Hospital, Athens, Greece

###### **Correspondence:** Paraskevi Korovessi

*Clinical and Translational Allergy* 2016, **6(Suppl 1)**:TP43

**Introduction:** Paradoxical bronchospasm describes an unexpected, rare and under recognised, adverse life threatening reaction of bronchoconstriction following β_2_ adrenergic receptor agonist inhalation. Several previous case reports have been described in adults, but to our knowledge, none in children.

**Case report:** A 10-year-old boy, with a history of intermittent asthma and sensitisation to dust mites was admitted for an asthma exacerbation. Following an initial improvement, he developed a sudden unexplained severe cyanotic episode with loss of consciousness and bradycardia a few seconds after salbutamol neb. He was admitted to ICU where prednisolone, aminophylline and nebulised salbutamol were administered. He was discharged on a Formoterol/budesonide inh. combination. One month later, during a scheduled visit, a spirometry test with bronchodilation was performed. A few second after salbutamol inhalation, a sharp drop of FEV1 was noted (from 96 to 35 % of predicted value) while the boy developed severe respiratory distress and ultimately silent chest and cyanosis. He was transferred to ICU and treated with oxygen, nebulised ipratropium, systematic corticosteroids with gradual clinical improvement. The patient’s respiratory distress resolved over 20 min and completely normalised within 2 h.

Both events were attributed to paradoxical bronchospasm following salbutamol. In the past, when the patient had tolerated salbutamol on several occasions, there has always been a co-administration of systematic steroid treatment, that may have had a protective effect. It is also worth noting that there is a family history of similar adverse bronchoconstrictive reactions to salbutamol.

**Conclusion:** Salbutamol is one of the most common and effective rescue bronchodilator medications used to treat asthma. Yet, paradoxical life threatening bronchospasm, is a well described adverse effect in adults. While a massive influx of eosinophils has been proposed, the exact mechanism remains unknown. A high index of awareness of this adverse effect can be life saving to the patient.

**Consent to publish**

Written informed consent for publication of this clinical details and/or clinical images was obtained from the patient/parent/guardian/relative of the patient. A copy of the consent form is available for review by the Editor of this journal.

### TP44

**WITHDRAWN**

*Clinical and Translational Allergy* 2016, **6(Suppl 1)**:TP44

### TP45Asthma symptoms in children with treatment for allergic rhinoconjunctivitis

####  Jorien Wartna, Arthur M. Bohnen, Gijs Elshout, David H. J. Pols, Patrick J. E. Bindels

##### Erasmus MC, Rotterdam, The Netherlands

###### **Correspondence:** Jorien Wartna

*Clinical and Translational Allergy* 2016, **6(Suppl 1)**:TP45

**Background:** Children with co-existing physician-diagnosed asthma and allergic rhinoconjunctivitis (AR) have worse asthma outcomes than those with asthma only. Proper treatment of AR might benefit the lower airways. It is unclear how INCS or oral antihistamine use can contribute to more controlled asthma in children with both conditions.

**Methods:** In a single-blind RCT with three parallel treatment groups the effectiveness of hay fever treatment in children was studied using intranasal corticosteroids or oral antihistamines. All patients had AR and some concomitant asthma. A daily online (AR and asthma) symptom diary was completed during the hay fever season. Patients with concomitant asthma also received an asthma specific quality of life questionnaire and recorded their asthma control. In all patients the primary outcome is the comparison between AR symptom scores and asthma symptom scores. Furthermore, in patients with concomitant asthma the secondary outcome is asthma control and asthma specific quality of life.

**Results:** General practitioners (n = 155) invited 4858 children, 419 of which responded positively. Finally 150 children and adolescents were randomized, of which 23 with concomitant asthma. Of those patients, 70 % used any type of asthma medication in the last month. At baseline, most patients had their asthma under control. Analyses on mean daily AR symptoms score and mean daily asthma symptom score will be performed. As well as asthma control and asthma specific quality of life in patients with concomitant asthma.

**Conclusion:** In this study we were able to provide explorative data on the effect of AR control on asthma symptoms and specific asthma outcomes. The results will provide more insight for the theory that better controlled AR will lead to better controlled asthma. It also will give insight in the asthma symptoms in patients with AR but no co-existing physician-diagnosed asthma during the hay fever season.

### TP46 Atopy increased the risk of developing exercise-induced bronchoconstriction in young athletes

####  Sven F. Seys^1^, Ellen Dilissen^1^, Sarah Van der Eycken^1^, An-Sofie Schelpe^1^, Gudrun Marijsse^1^, Thierry Troosters^2^, Vincent Vanbelle^3^, Sven Aertgeerts^4^, Jan L. Ceuppens^1^, Lieven J. Dupont^2^, Koen Peers^5^, Dominique M. Bullens^6^

##### ^1^Laboratory of Clinical Immunology, KU Leuven, Leuven, Belgium; ^2^Laboratory of Pneumology, KU Leuven, Leuven, Belgium; ^3^Flemish Swimming Federation, Belgium; ^4^ Academic Centre for General Practitioners, KU Leuven, Leuven, Belgium; ^5^Sport Medical Advice Centre, UZ Leuven, Leuven, Belgium; ^6^Laboratory of Pediatric Immunology, KU Leuven, Leuven, Belgium

###### **Correspondence:** Sven F. Seys

*Clinical and Translational Allergy* 2016, **6(Suppl 1)**:TP46

**Introduction:** Exercise-induced bronchoconstriction (EIB) is more common in athletes compared to the general population. The eucapnic voluntary hyperventilation test is the gold standard (IOC-MC) to detect EIB. We previously demonstrated the presence of epithelial damage and increased damage associated molecular patterns in elite athletes compared to control individuals [1].

**Methods:** Young athletes (basketball (n = 13), football (n = 20), swimming (n = 12)) were recruited at the start of their elite sports career (12–14 years). In some of the individuals (75 %), a second or third EVH test after 1 and 2 years. Eight age-matched controls were also recruited. Eucapnic voluntary hyperventilation test was performed according to ATS guidelines in all subjects as previously described [2]. Serum Clara Cell protein 16 (CC16) was measured by ELISA as a marker of airway epithelial damage.

**Results:** At time of first evaluation, 3/13 basketball players, 4/20 football players, 5/12 swimmers and 1/8 controls met criteria for EIB (fall in FEV_1_ >10 % after EVH test). The maximal fall in FEV_1_ after the EVH test was significantly lower in atopic compared to non-atopic athletes both after 1 and 2 years but not at time of first analysis. Atopic athletes furthermore showed significantly decreased maximal fall in FEV_1_ after one year, whereas this was unchanged in non-atopic athletes. Serum CC16 was significantly increased in athletes compared to controls at time of first analysis.

**Conclusion:** EIB is detected in young athletes, especially in swimmers. The degree of EIB response in young athletes was associated with atopy. Signs of epithelial damage were already found in young athletes at the start of their elite sports career. Follow up of the development of EIB is advised in young athletes, performing sports at elite level, especially in those with atopy.

**References**

1. Seys SF, Hox V, Van Gerven L, Dilissen E, Marijsse G, Peeters E, e.a. Damage-associated molecular pattern and innate cytokine release in the airways of competitive swimmers. Allergy. 2015;70(2):187–94.
2. Parsons JP, Hallstrand TS, Mastronarde JG, Kaminsky DA, Rundell KW, Hull JH, e.a. An official American Thoracic Society clinical practice guideline: exercise-induced bronchoconstriction. Am J Respir Crit Care Med. 2013;187(9):1016–27

### TP47 The effect of higher BMI on risk for asthma and treatment outcome in overweight and obese children

####  Ivana Banic, Sandra Bulat Lokas, Jelena Zivkovic, Boro Nogalo, Iva Mrkic Kobal, Davor Plavec, Mirjana Turkalj

##### Children's Hospital Srebrnjak, Zagreb, Croatia

###### **Correspondence:** Ivana Banic

*Clinical and Translational Allergy* 2016, **6(Suppl 1)**:TP47

**Introduction:** Asthma and obesity have a considerable impact on public health with obesity being a risk factor for asthma. Obesity can reduce pulmonary compliance, lung volumes and the ventilation-perfusion relationship.

**Aims and objectives:** To assess the effect of higher BMI on risk for asthma, airway inflammation and treatment outcomes in asthmatic children.

**Methods:** Of 2000 children (healthy and asthmatics), a cohort of 475 children with asthma was recruited. They underwent physical examination, basic anthropometric measurements, blood sampling and lung function tests. We clinically assessed their health status and treatment outcome at the point of diagnosis, after 6 months and after 12 months.

**Results:** Participants were categorized into 4 groups according to BMI percentile: underweight, normal, overweight and obese. Increased body weight was more prevalent in male participants, both overweight and obese, than in female. Baseline levels of hsCRP were elevated both in overweight and obese participants, compared to children with normal BMI. When treatment success was assessed by changes in airway inflammation after 6 months, increased FeNO levels were more frequent in inadequate and bad responders, compared to children with good response to treatment. The risk for asthma in all 2000 children was higher in overweight participants compared to children with normal BMI, but not in obese.

**Conclusions:** The effect of obesity appears to be insufficient in the development of asthma alone. Increased BMI (overweight) increases the risk for asthma and obesity rather increases the level of airway and systemic inflammation and potentially affects the level of disease control and response to asthma treatment.

### TP48

**WITHDRAWN**

*Clinical and Translational Allergy* 2016, **6(Suppl 1)**:TP48

### TP49

**WITHDRAWN**

*Clinical and Translational Allergy* 2016, **6(Suppl 1)**:TP49

### TP50

**WITHDRAWN**

*Clinical and Translational Allergy* 2016, **6(Suppl 1)**:TP50

### TP51

**WITHDRAWN**

*Clinical and Translational Allergy* 2016, **6(Suppl 1)**:TP51

### TP52 The impact of a multidisciplinary project intended to change the culture of nebulisers towards pressurised metered dose inhalers

####  Georgeta Oliveira^1^, Katharine Pike^2^, Alda Melo^3^, Tomás Amélia^3^, José Carlos Cidrais Rodrigues^1^, Cristina Serrano^1^, José Manuel Lopes dos Santos^1^, Carla Lopes^4^

##### ^1^Department of Women, Child and Youth, Local Health Unit of Matosinhos, Matosinhos, Portugal; ^2^Respiratory, Critical Care and Anaesthesia Section, University College London, Institute of Child Health, London, United Kingdom; ^3^Grouping of Matosinhos Health Centers, Local Health Unit of Matosinhos, Matosinhos, Portugal; ^4^Department of Clinical Epidemiology, Predictive Medicine and Public Health, School of Medicine, University of Porto, Porto, Portugal

###### **Correspondence:** Georgeta Oliveira

*Clinical and Translational Allergy* 2016, **6(Suppl 1)**:TP52

**Background:** Despite all recommendations supporting the use of pressurised metered dose inhalers with spacers (pMDI + S) for acute asthma treatment, there is still a considerable lack of its use of in Portugal not only within the emergency departments (ED) but also the home setting.

**Objectives:** The aim of this study was to evaluate the impact of the Optimisation of Inhaler Therapy in Children Project (OITCP) upon the use of pMDI + S at the ED and at home, considering the perspectives of children with asthma/wheezing, their parents/caregivers and health professionals.

**Methods:** Parents/caregivers (n = 325) and health professionals (n = 220) included in the OITCP were asked to complete questionnaires. Data related to respiratory/asthma control, device prescriptions and costs associated with this project were also analysed.

**Results:** Ninety-two (28 %) caregivers and 163 (74 %) health professionals responded to the questionnaires. Most parents/caregivers recognise the benefits of pMDI + S for asthma/wheezing treatment and more than 80 % expressed a preference for their use either at home or the ED. Similarly, most healthcare professionals recognise the advantages of these devices and the merits of the OITCP in changing their opinions and attitudes. Nearly 55 % of children surveyed had their asthma controlled, 4 % were hospitalised due to asthma/wheezing and 24 % had at least one hospital ED visit in the previous 12 months. Accounting for costs, the OITCP led to substantial savings to ULSM (over 30,000€) at no additional expense for families.

**Conclusions:** This study showed that the OITCP had a positive impact on both parents/caregivers and health professionals and was successful in encouraging use of pMDI + S at ED and at home.

### TP53

**WITHDRAWN**

*Clinical and Translational Allergy* 2016, **6(Suppl 1)**:TP53

### TP54

**WITHDRAWN**

*Clinical and Translational Allergy* 2016, **6(Suppl 1)**:TP54

### TP55

**WITHDRAWN**

*Clinical and Translational Allergy* 2016, **6(Suppl 1)**:TP55

### TP56 Increased asthma control in patients with severe persistent allergic asthma after 12 month of nightly temperature controlled laminar airflow (TLA)

####  Eckard Hamelmann^1,2^, Uwe Schauer^2,3^, Karl-Christian Bergmann^4^

##### ^1^Children’s Hospital, Children’s Center Bethel, EvKB, Bielefeld, Germany; ^2^Allergy Center Ruhr, Ruhr-University Bochum, Bochum, Germany; ^3^Children’s Hospital, St. Joseph Hospital, Bochum, Germany; ^4^Allergy Center Charité, Charité University Medicine, Berlin, Germany

###### **Correspondence:** Eckard Hamelmann

*Clinical and Translational Allergy* 2016, **6(Suppl 1)**:TP56

The published version of this abstract can be found at [1].

**Reference**

1. Eur Clin Respir J. 2015;2:28531—doi:10.3402/ecrj.v2.28531.

### TP57

**WITHDRAWN**

*Clinical and Translational Allergy* 2016, **6(Suppl 1)**:TP57

## THEMATIC POSTER SESSION 4: Drug allergy—Dermatology (TP58–TP77)

### TP58 Should we proceed directly to provocation challenges to diagnose drug allergy? Our experience says yes

#### Luis Moral, Teresa Toral, Nuria Marco, Beléns García Avilés, Mª Jesús Fuentes, Jesús Garde, Cristina Montahud, Javier Perona, Mª José Forniés,

##### Hospital General Universitario de Alicante, Alicante, Spain

###### **Correspondence:** Luis Moral

*Clinical and Translational Allergy* 2016, **6(Suppl 1)**:TP58

**Background:** True beta-lactam antibiotic hypersensitivity (BLAH) is very rare in childhood. The predictive values of skin and in vitro test are unknown. Oral provocation tests (OPT) are generally safely performed. We report our experience in the evaluation of children with suspected BLAH.

**Methods:** We reviewed patients attended for suspected BLAH in the allied pediatric allergy units of 6 close hospitals from 2010 to 2014. We collected data related to patients, past episodes, allergic workup and results. We analyzed the trends of in vitro and skin tests by means of Pearson’s correlation coefficient. We compared the characteristics of patients diagnosed of BLAH with those with a negative OPT by means of Fisher’s exact test.

**Results:** 668 patients. From 2010 to 2014, specific IgE test orders decreased from 45 to 27 % of patients (p < 0.001) and skin tests from 52 to 41 % (p = 0.003). OPT were performed in 89 % of patients (594 patients). BLAH was diagnosed in 27 patients (4 %): 26 by OPT results and 1 with a history of anaphylaxis and positive specific IgE and skin tests. BLAH was ruled out in 86 % of patients and 10 % did not conclude the study. All 26 patients with a positive OPT showed early or delayed skin signs (exanthema, urticaria, angioedema…) but none had severe or anaphylactic reactions. BLAH was more commonly diagnosed in patients with a history of more than one episode (9 vs. 3 %, P = 0.024), with a history of anaphylaxis (40 vs. 4 %, P = 0.017) and with cephalosporins (9 vs. 4 %, P = 0.086).

**Conclusions:** OPT is an easy and safe procedure to definitively diagnose or rule out BLAH in children. The role of skin and in vitro tests in pediatric patients is been questioned and needs clarification.

### TP59 Anaphylaxis to 13-valent pneumococcal vaccine

####  Esozia Arroabarren^1^, Marta Anda^1^, Maria Luisa Sanz^2^, Maria Teresa Lizaso^1^, Candida Arregui^1^

##### ^1^Complejo Hospitalario de Navarra, Pamplona, Spain; ^2^University Clinic of Navarra, Pamplona, Spain

###### **Correspondence:** Esozia Arroabarren

*Clinical and Translational Allergy* 2016, **6(Suppl 1)**:TP59

The incidence of severe allergic reactions after vaccination ranges from 0.5 to 1/100.000 cases.

A 12-month old infant was referred to our Consult after an anaphylaxis (urticaria and wheezing) appearing 1 h after receiving simultaneously the 1st dose of MMR (mumps, measles and rubella), 3rd dose of meningococcal C and the 4th dose of 13-valent pneumococcal vaccines (PCV13).

Intradermal tests showed positive results with PCV13. Meningococcal and MMR tested negative. PCV13 comprises 13 capsular *Streptococcus pneumoniae* polysaccharide serotypes conjugated to a non-toxic diphtheria toxin mutant (cross-reactive material [CRM(197)]). Current vaccination schedules recommend 4 doses of DTP (Diphtheria-tetanus toxoid-pertussis) within the first 2 years and a booster dose at 6. Therefore, skin tests were performed with 2 DTP variants, authorized for children of different ages: DTP1 (Composition: at least 30 international units [IU] of diphtheria, 40 IU of tetanus, and 25mcg of pertussis toxoids; authorized for infants) and DTP2 (Composition: at least 2 IU of diphtheria, 20 IU of tetanus, plus 8mcg of pertussis toxoids; authorized for children older than 4 years) with positive results for both.

Basophil activation tests (BAT) performed with PCV13, 23-valent pneumococcal vaccine and both DTP vaccines showed positive results with PCV13 and both DTP vaccines. Two controls tested negative.

Further tetanus and pertussis doses have been suspended since there are no mono-component vaccines available in Spain. Reevaluation of DTP sensitization has been offered in a few years since there have been reports of DTP hypersensitivity resolution.

This is the first report of anaphylaxis after PCV13 administration and the first time its carrier protein, CRM (197), is identified as its cause, demonstrated by BAT and skin tests results.

Carrier proteins are used to enhance infants’ immune response against capsulated bacteria. They should be considered in the assessment of hypersensitivity reactions to combined and/or conjugated vaccines.

**Consent to publish**

Written informed consent for publication of this clinical details and/or clinical images was obtained from the patient/parent/guardian/relative of the patient. A copy of the consent form is available for review by the Editor of this journal.

### TP60 Intrapartum antibiotic exposure for treatment of group B streptococcus was not associated with the development of penicillin allergy in children

####  Sara May, Martha Hartz, Avni Joshi, Miguel A. Park

##### Mayo Clinic, Rochester NY, USA

###### **Correspondence:** Miguel A. Park

*Clinical and Translational Allergy* 2016, **6(Suppl 1)**:TP60

**Background:** Group B streptococcus (GBS) is the leading infectious cause of neonatal morbidity and mortality in the Unites States. Intrapartum antibiotic delivery to GBS positive mothers is performed for prevention of neonatal infection with penicillin being the drug of choice. Previous studies have noted an increased risk of atopic diseases associated with intrapartum antibiotic exposure. This study sought to determine if intrapartum exposure to penicillin for GBS increased the likelihood of penicillin allergy in children.

**Methods:** Retrospective, chart review was performed on patients from a birth cohort. Birth cohort included children born in 2007 at a local tertiary care hospital with local addresses. GBS status of mother, intrapartum antibiotic exposure, delivery mode and birth order was collected and analyzed.

**Results:** We identified 927 children with 812 included in the cohort. Eighty (10 %) of the children had a reported penicillin allergy, most were Caucasian (79 %) males (61 %). Intrapartum exposure to penicillin (OR 0.84, CI 0.45–1.57, p = 0.59), amoxicillin or ampicillin (OR 0.22, CI 0.01–2.71, p = 0.29) did not increase the risk of penicillin allergy in children. The timing of antibiotic therapy (OR 1.00, CI 0.53–1.88, p = 0.99), GBS status of mother, mode of delivery or birth order did not affect the risk of penicillin allergy in the offspring.

**Discussion:** To our knowledge, this is the first study to evaluate intrapartum exposure to penicillin for GBS treatment and the subsequent development of penicillin allergy in the child. However, our study was limited as it was a retrospective, chart review of a birth cohort from a tertiary care facility with children lost to follow up.

**Conclusions:** In contrast to other atopic diseases, intrapartum antibiotic exposure as well as mode of delivery is not associated with the development of penicillin allergy in children. Parents and obstetricians should be reassured when using Penicillin for prevention of neonatal GBS.

### TP61 Evaluation of suspected drug hypersensitivity reactions in 169 children referred to the General Hospital

####  Sonja Posega Devetak^1^, Tina Vesel^2^, Anja Koren Jeverica^2^, Tadej Avčin^2^

##### ^1^General and Teaching Hospital Izola, Izola, Slovenia; ^2^University Children’s Hospital, Ljubljana, Slovenia

###### **Correspondence:** Sonja Posega Devetak

*Clinical and Translational Allergy* 2016, **6(Suppl 1)**:TP61

**Background:** The aim of our study was to evaluate the diagnostic work-up of children referred to a general hospital due to suspected drug hypersensitivity reactions (DHR), with the emphasis on children with immediate or severe non-immediate DHR, who were also referred to the University children’s hospital Ljubljana.

**Methods:** We retrospectively analysed medical documentation of 169 children who were referred to the General Hospital Izola due to a suspected DHR from 2009 to 2014. 125 children (73.9 %) had mild DHR due to which oral provocation test (OPT) were planned/made. All OPT in this group were negative except one. For further analysis were selected 44 children (16 female, 28 male, aged 1–9 years, mean 2.5 years) with clinical manifestation suspicious of immediate or severe non-immediate DHR that were further evaluated at the University Children’s Hospital Ljubljana.

**Results:** 17/44 (39 %) children were treated with penicillin G, one (2 %) with penicillin V, 17 (39 %) with amoxicillin, five (11 %) with amoxicillin and clavulanic acid, two with cefuroxime (5 %) and one (2 %) with ceftriaxone. Clinical manifestations of DHR were immediate urticaria (21), immediate urticaria with angioedema (seven), anaphylaxis (three), maculopapular exanthema in first two hours (three), maculopapular exanthema with additional alert signs (four), vasculitis (four), fixed drug erythema (one) and erythema multiforme (one). Beta lactam allergy was confirmed in seven children (4 penicillin G, 3 amoxicillin with clavulanic acid) by skin testing (ST). Five children were solely allergic to penicillin and two also to cephalosporins and meropenem. All children had negative specific IgE to betalactams, two children had also positive basophil activation test results. ST was inconclusive in two patients due to collapse and dermographism. Eight patients have not come for further investigations yet.

**Conclusion:** Drug hypersensitivity was confirmed in 4.7 % of referred children, more often when immediate or severe non-immediate DHR were suspected (in 15.9 %).

### TP62 Drug provocation testing: experience of a tertiary hospital

####  Leonor Castro, Carolina Gouveia, Ana Carvalho Marques, Antonio Jorge Cabral

##### Hospital Central do Funchal, Funchal, Portugal

###### **Correspondence:** Antonio Jorge Cabral

*Clinical and Translational Allergy* 2016, **6(Suppl 1)**:TP62

**Introduction:** All drugs have the potential to cause adverse reactions, although not all are allergic reactions. Drug allergy can be defined as any reaction caused by a drug, clinically compatible with an immunological mechanism. This study aims to understand the pediatric population with suspected drug allergy, followed in a specialized consultation.

**Methods:** This is an observational, cross-sectional, descriptive study. The study included children followed in a pediatric drug allergy consultation, from January 2012 to April 2015. The collection of demographic, clinical and laboratory data was obtained from the clinical records.

**Results:** In the studied period, 83 children were followed, of which 57 (69 %) were submitted to the oral provocation test (OPT) and 26 (31 %) are currently on a waiting list. In total, 80 OPT were performed, of which 26 % were re-provocations and 7 % were for alternative drugs. Drug allergy was confirmed in 8 patients. Even though the most suspected drugs were amoxicillin (75 %) and ibuprofen (18 %), only 4 children showed a positive OPT to amoxicillin (7 %) and 2 to ibuprofen (4 %). The most common clinical manifestations were cutaneous (urticaria and/or angioedema), in 91 % of children. In 2 patients the reaction was understood as being an anaphylactic reaction, but only in one of them the allergy was confirmed. Half of the patients with confirmed drug allergy were tested to an alternative drug, all of which were negative.

**Conclusion:** Our results are consistent with other studies in which penicillin appears as the most frequently identified drug with confirmed allergy in only 7 % of the patients. Studies show that only 10 % of the population believed to be allergic to penicillin is truly allergic. Children with unconfirmed allergies risk greater exposure to broad-spectrum antibiotics, with all its implications, as well as sub-optimal therapy and should be promptly oriented to a specialized consultation for diagnosis.

### TP63 Perioperative anaphylaxis: a growing concern in pediatric population

####  Luis Amaral^1^, Fabrícia Carolino^1^, Eunice Castro^1^, Madalena Passos^2^, Josefina R. Cernadas^1^

##### ^1^Serviço de Imunoalergologia, Centro Hospitalar de São João E.P.E., Porto, Portugal; ^2^Serviço de Anestesiologia, Centro Hospitalar de São João E.P.E., Porto, Portugal

###### **Correspondence:** Luis Amaral 

*Clinical and Translational Allergy* 2016, **6(Suppl 1)**:TP63

**Background:** Serious adverse events are unusual during surgeries. Allergic reactions are among the major factors that contribute to perioperative morbidity and mortality. The literature on perioperative anaphylaxis is scarce, especially in the pediatric population.

**Aim:** To characterize pediatric patients with adverse allergic perioperative outcomes and to examine the most commonly involved agents.

**Methods:** We reviewed the medical records of patients ≤18-years-old with a perioperative anaphylaxis history referred to our drug allergy unit, between January 2009 and April 2015. The culprit drugs were assessed by positivity of intradermal skin tests (IDT) with nonirritant concentrations, in accordance to the published guidelines. All patients were screened for latex allergy by skin prick test (SPT) with commercial extract and specific IgE (sIgE) assay.

**Results:** Data of 10 patients (6 male, median age [minimum–maximum] 10 [1–17] were collected; 7 were atopic (6 allergic rhinitis; 4 asthma and 1 atopic eczema). In 2 patients, the SPT and the sIgE to latex were positive; nonetheless, in these cases, we also performed IDT to the used drugs which were all negative. Two patients had a positive IDT with a 1/100 dilution of cis-atracurium; 1 to neostigmine (1/100); 1 to fentanyl (1/100); 1 to ropivacaine (1/1000); 1 to midazolam (1/10) and 1 cefazolin (1/10). In 1 patient, latex screening and all the IDT to drugs involved during the surgery were negative and so it wasn’t possible to relate the reaction to any of the drugs used.

**Comments:** Perioperative anaphylaxis is becoming more common and thus recognition and evaluation by anesthesiologists in concert with allergists is of paramount importance. As other studies suggest, the presence of allergic disease seems to be positively associated to perioperative anaphylaxis. According to previous studies, neuromuscular blocking agents and latex were the main causes of IgE-mediated perioperative reactions. In this study, other pharmacologic groups were implicated including a local anesthetic (ropivacaine), which is rarely described as a cause of anaphylaxis.

### TP64 Raising awareness of hypersensitivity to non-steroidal anti-inflammatory drugs in the pediatric age

####  Fabrícia Carolino, Luís Amaral, Eunice Dias de Castro, Josefina R. Cernadas

##### Serviço de Imunoalergologia, Centro Hospitalar São João E.P.E., Porto, Portugal

###### **Correspondence:** Fabrícia Carolino

*Clinical and Translational Allergy* 2016, **6(Suppl 1)**:TP64

**Background:** Non-steroidal anti-inflammatory drugs (NSAIDs) are a common cause of hypersensitivity (HS) reactions and they are widely prescribed for paediatric use. Few studies have specifically addressed NSAID-HS in this age group.

**Aim:** To review cases of children and adolescents with suspected NSAID-HS.

**Methods:** Medical records’ review of 37 patients under 18 years of age, studied in the Drug Allergy Division of our Department for suspected NSAID-HS, during a 3 year period. Reactions were classified according to Kowalsky et al. (2013).

**Results:** Patients’ median age was 11.0 years (interquartile range 6.5–15.5; min–max 1–17) with a similar gender distribution (54.1 % female); 43.2 % were atopic (81.3 % with single NSAIDs reactivity).

Reactions to a single NSAID were reported by 30 (81.1 %) of all patients (30.0 % with more than one episode), and ibuprofen was the most common incriminated drug (83.3 %). In this group, the reactions were acute (until 24 h) in 83.3 %, the most frequent clinical manifestation was urticaria and/or angioedema (76.7 %) and 10 % presented anaphylaxis; diagnostic oral provocation test (OPT) was performed in 76.7 %.

Of the 7 (18.9 %) patients with reactions to multiple NSAIDs, 71.4 % presented with urticaria and/or angioedema and none with anaphylaxis.

In the total sample, NSAID-HS was confirmed in 4 of the 26 (15.4 %) patients that underwent diagnostic OPT:

- three with acute urticaria to ibuprofen (2 reacted with the total cumulative dose; all tolerated paracetamol and one performed safely OPT with the alternative drug nimesulide);
- one with acute urticaria to paracetamol (elicited by the age-recommended full dose; the child had a reported tolerance to ibuprofen and no alternative drug was tested);
- all reactions were managed in an outpatient basis, with antihistamine/corticosteroid treatment.

All 14 patients that performed OPT with an alternative NSAID (85.7 % with drugs more selectively inhibiting COX-2) tolerated the tested drug, including the 3 cases of anaphylaxis (to ibuprofen).

**Discussion:** NSAID-HS was confirmed only in 15.4 % of those who performed diagnostic OPT. Alternative drugs were safely tested, even in patients with more severe index reaction.

### TP65 Perioperative anaphylaxis in young children: how to confirm the suspicion

####  Josefina R. Cernadas^1^, Fabrícia Carolino^1^, Luís Amaral^1^, Fernando Pineda^2^, Armanda Gomes^3^

##### ^1^Serviço de Imunoalergologia, Centro Hospitalar São João EPE, Porto, Portugal; ^2^Application Department, DIATER, Madrid, Spain; ^3^Serviço de Anestesiologia, Centro Hospitalar São João EPE, Porto, Portugal

###### **Correspondence:** Fabrícia Carolino

*Clinical and Translational Allergy* 2016, **6(Suppl 1)**:TP65

**Background:** Perioperative anaphylactic reactions are immediate, potentially life-threatening, hypersensitive reactions. Recognition by anesthesiologists and allergic study by allergists is of outmost importance. The most frequent causing agents are neuromuscular blocking agents (NMBAs), latex and antibiotics with latex being the first cause in pediatric age.

**Aim:** The authors describe two cases of anaphylaxis to ropivacaine and rocuronium in a 10 and 11 years old, both males, and discuss in vivo and in vitro diagnosis work up.

**Case 1:** Ten year old boy, submitted to a circumcision surgery, with immediate symptoms and signs of severe anaphylaxis coincident with ropivacaine administration. Tryptase level by the time of reaction was 15.60 mcg/l (<11 mcg/l).

Latex allergy was first excluded by negative skin prick tests (SPT) and specific IgE (sIgE). Intradermal test (IDT) with ropivacaine 2 mg/ml at a dilution of 1/1000 was clearly positive. Tests to lidocaine, without vasoconstrictor were doubtful.

As both drugs were from the same local anesthetic group, a subcutaneous provocation test with procaine was performed in the presence of an anesthesiologist, without any reaction.

**Case 2:** Eleven year old boy reacted with generalized urticaria and bronchospasm during an appendicectomy. Latex allergy was excluded as in the previous case.

Because many drugs were used (propofol, fentanyl, tramadol and rocuronium), SPT and IDT tests were performed to each one, according to the guidelines, with a clear positivity to rocuronium. As alternatives to the culprit, we tested vecuronium and suxamethonium with negative results.

The authors discuss that specific IgE, basophil activation tests (BAT) to the culprit drugs or passive sensitization by histamine release test (HRT) are expected to be performed to confirm the mechanism of the reaction.

**Comments:** Any suspected hypersensitive reaction during anesthesia must be extensively investigated by pre and post-operative tests, independent of patient’s age.

The authors discuss a complete in vivo and in vitro diagnosis work up in pediatric age especially in young children where the IDT cannot be feasible.

**Consent to publish**

Written informed consent for publication of this clinical details and/or clinical images was obtained from the patient/parent/guardian/relative of the patient. A copy of the consent form is available for review by the Editor of this journal.

### TP66 A case study of a child suspected to be penicillin allergic-digging deeper

####  Katherine Knight, Roisin Fitzsimons, Helen Brough

##### St. Thomas’ Hospital, London, United Kingdom

###### **Correspondence:** Katherine Knight

*Clinical and Translational Allergy* 2016, **6(Suppl 1)**:TP66

**Objectives:** Many children in the UK are suspected to be penicillin allergic. Having the incorrect label of a penicillin allergy can have implications for life long health care. As per the BSACI beta lactam allergy guidelines, it is recommended that drug provocation testing (DPT) be used as a first line diagnostic procedure in children with mild adverse reactions. Often a child will return to the unit for further DPT to establish a safe alternative, to treat further infections. This case study discusses the importance of correct identification and DPT of suspected drugs to arrive at an accurate diagnosis.

**Methods:** A 3 year old boy presented to the allergy outpatients in Jan 2014 with a history of multiple food allergies. During the allergy consultation it was noted he had reacted to Amoxicillin syrup with immediate and widespread itching whilst being treated for an ear infection. He had previously tolerated oral Amoxicillin for treatment of a chest infection. The patient was switched to oral Azithromycin by his GP and reacted with an urticarial rash and eye swelling. Incremental, oral DPT to Amoxicillin and Azithromycin were planned with further DPT if needed to establish a suitable antibiotic alternative.

**Results:** The patient had a positive oral DPT to Amoxicillin syrup and Azithromycin syrup resulting in immediate urticaria and delayed eczema flare. An oral cefuroxime axetil syrup DPT resulted in a delayed eczema flare. A reaction to an excipient of the syrup preparations was suspected. After involvement from the medicines information team, the patient had a successful DPT to Amoxicillin tablets in July 2014 which did not contain any of the suspected excipients.

**Conclusions:** Children who experience allergic reactions to multiple types of antibiotics require consideration of whether an excipient of the antibiotics is the cause.

**Consent to publish**

Written informed consent for publication of this clinical details and/or clinical images was obtained from the patient/parent/guardian/relative of the patient. A copy of the consent form is available for review by the Editor of this journal.

### TP67 Prevalence, characteristics and risk factors of hypersensitivity reactions to antibiotics in patients with cystic fibrosis

####  Jobst Röhmel, Carsten Schwarz, Anne Mehl, Philippe Stock, Doris Staab

##### Charité Universitätsmedizin Berlin, Berlin, Germany

###### **Correspondence:** Jobst Röhmel

*Clinical and Translational Allergy* 2016, **6(Suppl 1)**:TP67

**Objectives:** Hypersensitivity reactions to parenteral administered antibiotics (HRPA) are a substantial problem in managing pulmonary disease in Cystic Fibrosis (CF), especially in advanced CF. This group of patients requires a life long antibiotic treatment with extremely high cumulative doses compared to other patients. In our daily routine we observed a growing number of hypersensitivities. Therefore we conducted this observational study to assess HRPA’s impact on the daily clinical work with CF, as well as its nature, frequency and predisposing risk factors.

**Methods:** By reviewing medical records and conducting interviews, age, sex, FEV1, ΔF508genotype, onset and duration of *pseudomonal* colonisation, allergy history (including IgE serum levels, past ABPA and results of screening tests for inhalative aeroallergens), parenteral antibiotic exposure and HRPA (timing, symptoms and treatment) were recorded. Included were all pediatric and adult patients at our centre with >3 intravenous antibiotic treatment courses.

**Results:** Of 100 patients included in the study, 60 had ≥1 HRPA. Overall, 3205 antibiotic courses with 185 HRPA were ascertained. 15 % of HRPA met the criteria for anaphylaxis. Symptoms were mostly dermal (53 %). 81 % of all and 80 % of anaphylactic HRPA occurred during days 14. Approximately 10 % of all treatment courses with cefepime and piperacillin/tazobactam caused HRPA. The number of years with pseudomonal colonisation and the cumulative annual exposure of the given antibiotic were significant risk factors for HRPA in our patient cohort.

**Conclusions:** Our results demonstrate that HRPA with a prevalence of 60 % are very relevant. During days 14of antibiotic Treatment courses patients might be at elevated risk to experience HRPA. HRPA appear to be drug specific and to be dependent on cumulative annual drug exposure of the given drug. High cumulative dose of the same antibiotic over a short period of time may lead to a higher risk of HRPA than the same dose over a longer period of time. Is this an argument to change the therapeutic regimen more often? Diagnostic algorithms for this CF specific problem should be developed further. Besides recent publications about drug specific lymphocytes in patients with CF, we believe that further elucidation of HRPA’s immunological mechanisms is needed.

### TP68 Antibiotic drug hypersensitivity in cystic fibrosis: A pilot study using cellular allergy tests for diagnostics

####  Jobst Röhmel, Carsten Schwarz, Christine Seib, Doris Staab, Philippe Stock

##### Charité Universitätsmedizin Berlin, Berlin, Germany

###### **Correspondence:** Jobst Röhmel

*Clinical and Translational Allergy* 2016, **6(Suppl 1)**:TP68

**Objectives:** Hypersensitivity reactions to parenteral antibiotics (HRPA) are a major problem in the management of patients with CF. These patients require lifelong treatment with antibiotics in high cumulative doses. Allergy testing methods such as specific IgE in serum are not available for antipseudomonal antibiotics. Skin tests seem to have limited value. This pilot study was carried out to assess the diagnostic value of in vitro allergy testing methods for HRPA and the underlying immune reactions.

**Methods:** Patients with CF and a documented hypersensitivity to ≥1 parenteral antibiotic were recruited. The suggested antibiotic had never been prescribed after the initial reaction. For a total of 13 patients, basophil activation tests (BAT) and lymphocyte transformation tests (LTT) for each suspected antibiotic were performed (piperacillin/tazobactam (pip/taz), ceftazidime, meropenem and others). BAT with an activation index (AI) of >2.5 and LTT with a stimulation index (SI) >2 were considered positive.

**Results:** Two positive BAT (each for pip/taz, AI = 2.7 and 3.1) and seven positive LTT in five patients were ascertained (pip/taz SI = 76.6 and 2.2; cefepime SI = 10.5, 3.0 and 2.4; sulbactam SI = 8.7; ceftazidime SI = 2.1).

**Conclusions:** The pilot study showed mostly for LTT positive results. This may indicate a T-cellular genesis of HRPA that is consistent with the lack of association to atopy. These results could be an argument for this method as a diagnostic tool for HRPA. The sample size was small though and clinical trials with re-exponation of the negative tested patients are pending. Therefore, no clear recommendations can be applied for the routine clinical setting.

### TP69 Oral antibiotics challenges in children

####  Anita Critchlow, Alyson Barber, Nicola Jay

##### Sheffield Children’s Hospital, Sheffield, United Kingdom

###### **Correspondence:** Nicola Jay

*Clinical and Translational Allergy* 2016, **6(Suppl 1)**:TP69

**Introduction:** Children are often labeled as allergic to antibiotics without good evidence. We have previously shown that many children will pass an antibiotic challenge under such circumstances. Consequently we altered the protocol used during an antibiotic challenge to reduce the doses given and time needed. The hope being that this will eventually be used in primary care.

**Method:** Children were initially seen in general allergy clinic with either an urticarial rash or maculopapular rash developing during a treatment course of oral antibiotics. All of these children had negative SPT and RAST to the antibiotic of concern or by default Amoxicillin. Sixty-one children underwent the new protocol for antibiotic challenge from January to June 2015. This comprised three doses given 20 min apart at 1/10th dose, 4/5th dose and half for the final dose. Children where then observed for an hour and discharged if well to complete a 5 day course of antibiotics at home.

**Results:** No child failed the escalating challenge protocol in hospital however three developed rashes during the continuation phase at home. The antibiotic challenged was either Amoxicillin (27), or Penicillin (11), Macrolide (14), Cephalosporins (5) or other (4). All of the rashes developed in children challenged to either Amoxicillin or Co-Amoxiclav.

**Conclusion:** Oral antibiotic challenges are safe in children even using a shorter protocol. In theory reducing the protocol further and re-evaluating may allow more children access to the service, thus enabling de-labelling. We may even be able to perform challenges without allergy tests having been done. Public Health relies on rationale use of antibiotics and the current state of labelling antibiotic allergy in children is irrational.

### TP70 Hypersensitivity reaction to vancomycin: a new successful desensitization protocol

####  Belen Delavalle, Teresa Garriga, Blanca Vilá, Cristina Blasco

##### Department of Pneumology, Allergy and Cystic Fibrosis, Pediatric Allergy Unit, Vall d’Hebron University Hospital, Barcelona, Spain

###### **Correspondence:** Belen Delavalle

*Clinical and Translational Allergy* 2016, **6(Suppl 1)**:TP70

**Introduction:** Hypersensitivity reactions to vancomycin can occur through two different mechanisms. Although both reactions involve mast cell activation, vancomycin-induced anaphylactic reactions are mediated by immunoglobulin E (IgE), whereas anaphylactoid reactions (i.e. Red Man Syndrome) are not. In some infections, vancomycin is the antibiotic of choice. Therefore, in IgE mediated reactions, vancomycin desensitization can be a reasonable option and be performed successfully in most cases.

**Case report:** A 5-year-old boy, with congenital hydrocephalus and double carrier ventricular peritoneal shunt valve, was admitted to our hospital for valve dysfunction and infection. The cerebrospinal fluid culture was positive for *Staphylococcus epidermidis.* The patient required valve cystoperitoneal replacement. Few minutes before the surgery, vancomycin was administered intravenously. Immediately, he suffered generalized urticaria, pruritus, tachycardia (170 beats/min), hypotension (62/24 mmHg) and severe respiratory distress. Patient was treated with antihistamines, corticoesteroids and adrenaline with clinical improvement. Serum tryptase was measured at 1, 2 and 6 h following onset of the episode and at basal conditions. As the patient had experienced an anaphylactic shock due to vancomycin, linezolid treatment was started without clinical improvement. For this reason, a vancomycin desensitization protocol was designed. Dosing was administered at intervals of one hour, in the following increasing doses: 0.05, 0.5, 1, 5, 10, 20, 30, 50, 60 and 80 mg. Premedication with dexchlorpheniramine was administered.

**Allergy test:** Laboratory data: Total serum IgE 80.2 KU/L. Tryptase concentration at 1 h: 19.6 mcg/l, at 2 h: 15.2 mcg/l, at 6 h: 10.7 mcg/l. Baseline tryptase 3.39 mcg/l. Skin test (prick and intradermal) to vancomycin were negative.

**Conclusions:** We report a new successful vancomycin desensitization protocol in a pediatric patient who experienced an anaphylactic shock. Vancomycin desensitization should be considered for severe reactions to vancomycin when substitution for another antibiotic is not feasible.

**Consent to publish**

Written informed consent for publication of this clinical details and/or clinical images was obtained from the patient/parent/guardian/relative of the patient. A copy of the consent form is available for review by the Editor of this journal.

### TP71

**WITHDRAWN**

*Clinical and Translational Allergy* 2016, **6(Suppl 1)**:TP71

### TP72 Clinical phenotypes according to FLG gene loss of function mutations in children with atopic dermatitis

####  Francesca Cipriani^1^, Annalisa Astolfi^2^, Costanza Di Chiara^1^, Elisabetta Calamelli^1^, Iria Neri^3^, Annalisa Patrizi^3^, Gianpaolo Ricci^1^

##### ^1^Pediatric Unit, Department of Medical and Surgical Sciences, University of Bologna, Bologna, Italy; ^2^“Giorgio Prodi” Cancer Research Center, University of Bologna, Bologna, Italy; ^3^Dermatology Unit, Department of Experimental, Diagnostic and Specialty Medicine, University of Bologna, Bologna, Italy

###### **Correspondence:** Francesca Cipriani

*Clinical and Translational Allergy* 2016, **6(Suppl 1)**:**72**

**Background:** Recent evidences showed an association between FLG gene mutations and other allergic diseases in children with atopic dermatitis (AD) [1–3]. The frequency of mutations varies widely among different population [4].

**Methods:** We enrolled Italian children suffering from AD aged 6 months–18 years. Patients underwent clinical evaluation, skin prick test (SPT) and blood sampling for determination of total and specific IgE and to analyse FLG gene. Purified PCR products were sequenced on both strands using a Big Dye Terminator v1.1 Cycle Sequencing kit (Applied Biosystems) and run on an ABI 3730 Genetic Analyzer (Applied Biosystem).

**Results:** 223 patients with AD were recruited from June 2011 to June 2014. 57.8 % of them were males, the mean age was 6.1 years. The prevalence of asthma was 26.5 %, rhinoconjunctivitis (RC) 48.4 % and food allergy (FA) 42.0 %. We detected heterozygous FLG gene null mutations in 16 patients (7.2 %): R501X in 14 cases, 2282del4 in 2 cases. By comparing FLG null carriers vs. *wild type* patients, we didn’t found differences in gender (males: 43.8 vs. 58.9 %), family history of atopy (68.8 vs. 58.5 %), severity of AD (mild 37.5 vs. 71 %, moderate 50 vs. 22.7 %, severe 12.5 vs. 6.3 %). The mean age at onset of AD was 3.9 months among FLG null carriers and 12.7 months among *wild type* patients (p = 0.015), without differences in the remission rate (37.5 vs. 37.9 %). The prevalence of asthma (12.5 vs. 37.1 %) and RC (50 vs. 47.3 %) didn’t differ between FLG null carriers and *wild type* FLG patients, while the prevalence of FA was higher in FLG null carriers (75 vs. 39.1 %, p = 0.007). Total IgE levels and IgE sensitization to airborne allergens wasn’t different among the two groups, while IgE sensitization to food allergens was higher in FLG null carriers (75 vs. 42.5 %, p = 0.017).

**Conclusions:** Our data confirmed the low frequency of FLG mutation among Italian children with AD. The presence of FLG null mutations was related to an earlier age at onset of AD, to a higher prevalence of IgE sensitization to food allergens and FA.

**References**

1. Palmer CAN, et al. Nat Genet. 2006.
2. Venkataraman D, et al. JACI. 2014.
3. Brough HA, et al. JACI 2014.
4. Cascella, et al. J Invest Dermatol 2011.

### TP73

**WITHDRAWN**

*Clinical and Translational Allergy* 2016, **6(Suppl 1)**:TP73

### TP74 Urticaria in children: clinical and epidemiological features

####  Katerina Neskorodova, Asya Kudryavtseva

##### I.M. Sechenov First Moscow State Medical University, Moscow, Russia

###### **Correspondence:** Katerina Neskorodova

*Clinical and Translational Allergy* 2016, **6(Suppl 1)**:TP74

**Background:** Ubiquitous growth of allergic diseases and a limited number of publications dedicated to epidemiology of urticaria in children determine significance of the study.

**Objective:** To assess clinical and epidemiological features of acute and chronic urticaria in pediatric population.

**Materials and methods:** 63 children aged 0–16 years with clinical signs of urticaria were included in an observational, cross-sectional study. 45 of them had acute, 18 had chronic urticaria. M:F ratio was 1:1.

**Results:** The mean age at diagnosis of acute urticaria was 6.2 years. Isolated appearance of wheals was observed in 27 %, angioedema in 13 %, combination of two symptoms -in 60 %. Causative factors were identified in 69 % of cases. Common triggers were: foods (42 %), medication use (22.6 %), exposure to pollen (16 %), insect stings (9.7 %), cosmetics (9.7 %). 47 % of patients have a history of allergic diseases. Laboratory studies revealed high IgE in 65 % and high eosinophil count in 18 %. Most patients were treated with sAH (55.6 %), others with nsAH. 42 % of patients received at least one injection of corticosteroids.

The mean age at diagnosis of chronic urticaria was 8.7 years, the mean disease duration was 17 mths (max 48). Patients were classified as having chronic spontaneous urticaria (94 %) and physical urticaria (6 %). Isolated appearance of wheals was observed in 53 %, together with angioedema—in 47 %. Causative factors were identified in 28 % of cases and included: helminthic infection, foods and insect stings. Common comorbidities were: allergic diseases (41 %), *H. pylori* infection (35 %), thyroiditis (16.6 %). Exacerbation with emotional stress was reported by 17.6 % of patients. High IgE level was detected in 47 %, allergen-specific IgE tests were positive in 25 %. In 82.3 % for treatment were used nsAH. The efficiency of standard doses was 83 %. In 27.8 % of cases patients received short parenteral courses of corticosteroids.

**Conclusions:** The mean age of patients with chronic urticaria is higher, than with acute urticaria (p < 0.05). The etiologic agent is more likely to be identified in acute urticaria. In most cases chronic urticaria can be effective treated with standard doses of nsAH.

### TP75

**WITHDRAWN**

*Clinical and Translational Allergy* 2016, **6(Suppl 1)**:TP75

### TP76 Acute urticaria at the Pediatrics Emergency Department: is it allergy?

####  Esozia Arroabarren, Jorge Alvarez, Marta Anda, Miriam Palacios, Marta Martinez-Merino, Ibone Vaquero

##### Complejo Hospitalario de Navarra, Pamplona, Spain

###### **Correspondence:** Esozia Arroabarren

*Clinical and Translational Allergy* 2016, **6(Suppl 1)**:TP76

**Objective:** Acute urticaria (AU) is a frequent complaint in Pediatric Emergency Departments (PED). We searched for clinical data during the acute episode that may enable us to inform the parents about its origin, assessing a cohort of children attended at our PED and referred to Allergy Consults.

**Methods:** Retrospective review of PED charts of children attended for AU at both the PED and Allergy Consults (2009–2013). According to the allergy work-up they were included as allergy-induced urticaria (AIU) or non-allergic urticaria (NAU). We analysed: symptoms before urticaria onset, identification of triggers in the chart, atopy, fever and management of the episode (Chi Square). Patients’ ages and duration of symptoms at PED admission were also assessed (Mann–Whitney).

**Results:** We reviewed 44 charts (AIU Group: 22 patients; NAU Group: 22). There were significant differences in the age of the patients (median age in AIU Group: 2.2 years (3 months–14 years) vs. 6.5 years (1–15) in NAU Group; p = 0.035), previous atopic diseases (45.5 % in AIU vs. 5 % in NAU; P = 0.002), symptoms before the urticaria onset (27.2 % in AIU vs. 65.6 % in NAU; p = 0.013) and in the identification of triggers (foods in 81.8 % and drugs in 18.2 % of AIU vs. none in 71.3 % of NAU cases). No differences were detected in: coexisting fever (27.3 % in both; p = 0.632), pharmacological treatment: 81.8 % in AIU vs. 72.7 % in NAU (p = 0.951) or the duration of symptoms at PED arrival (median of 2 h (1–3 h) in AIU Group vs. 6 h (1–72 h) in NAU Group (p = 0.316).

**Conclusions:** Patients’ age, the identification of specific triggers in the anamnesis, presence of symptoms previous to the AU onset and coexisting atopy may be useful data in predicting whether an AU may be allergy related. However, the presence of fever and the duration of symptoms at PED arrival were not useful in our sample.

### TP77

**WITHDRAWN**

*Clinical and Translational Allergy* 2016, **6(Suppl 1)**:TP77

